# 90‐90‐90 by 2020? Estimation and projection of the adult HIV epidemic and ART programme in Zimbabwe – 2017 to 2020

**DOI:** 10.1002/jia2.25205

**Published:** 2018-11-22

**Authors:** Loveleen Bansi‐Matharu, Valentina Cambiano, Tsitsi Apollo, Raymond Yekeye, Jeffrey Dirawo, Sithembile Musemburi, Calum Davey, Sue Napierala, Elizabeth Fearon, Amon Mpofu, Owen Mugurungi, James R Hargreaves, Frances M Cowan, Andrew N Phillips

**Affiliations:** ^1^ Institute for Global Health UCL London UK; ^2^ Department of HIV/AIDS and STIs Ministry of Health and Childcare Harare Zimbabwe; ^3^ Zimbabwe National AIDS Council Harare Zimbabwe; ^4^ Centre for Sexual Health HIV/AIDS Research Zimbabwe Harare Zimbabwe; ^5^ Faculty of Public Health and Policy London School of Hygiene and Tropical Medicine (LSHTM) London UK; ^6^ RTI International San Francisco CA USA; ^7^ Department of Social and Environmental Health Research London School of Hygiene & Tropical Medicine London UK; ^8^ TB and AIDS Unit Zimbabwe Ministry of Health Harare Zimbabwe; ^9^ Department of International Public Health Liverpool School of Tropical Medicine Liverpool UK

**Keywords:** HIV care continuum, public health, testing, treatment, viral suppression

## Abstract

**Introduction:**

The 90‐90‐90 targets set by the United Nations aspire to 73% of people living with HIV (PLHIV) being virally suppressed by 2020. Using the HIV Synthesis Model, we aim to mimic the epidemic in Zimbabwe and make projections to assess whether Zimbabwe is on track to meet the 90‐90‐90 targets and assess whether recently proposed UNAIDS HIV transition metrics are likely to be met.

**Methods:**

We used an approximate Bayesian computation approach to identify model parameter values which result in model outputs consistent with observed data, evaluated using a calibration score. These parameter values were then used to make projections to 2020 to compare with the 90‐90‐90 targets and other key indicators. We also calculated HIV transition metrics proposed by UNAIDS (percentage reduction in new HIV infections and AIDS‐related mortality from 2010 to 2020, absolute rate of new infections and AIDS‐related mortality, incidence–mortality ratio and incidence–prevalence ratios).

**Results:**

After calibration, there was general agreement between modelled and observed data. The median predicted outcomes in 2020 were: proportion of PLHIV (aged 15 to 65) diagnosed 0.91 (90% uncertainty range 0.87, 0.94) (0.84 men, 0.95 women); of those diagnosed, proportion on treatment 0.92 (0.90, 0.93); of those receiving treatment, proportion with viral suppression 0.86 (0.81, 0.91). This results in 72% of PLHIV having viral suppression in 2020. We estimated a percentage reduction of 36.5% (13.7% increase to 67.4% reduction) in new infections from 2010 to 2020, and of 30.4% (9.7% increase to 56.6% reduction) in AIDS‐related mortality (UNAIDS target 75%). The modelled absolute rates of HIV incidence and AIDS‐related mortality in 2020 were 5.48 (2.26, 9.24) and 1.93 (1.31, 2.71) per 1000 person‐years respectively. The modelled incidence–mortality ratio and incidence–prevalence ratios in 2020 were 1.05 (0.46, 1.66) and 0.009 (0.004, 0.013) respectively.

**Conclusions:**

Our model was able to produce outputs that are simultaneously consistent with an array of observed data and predicted that while the 90‐90‐90 targets are within reach in Zimbabwe, increased efforts are required in diagnosing men in particular. Calculation of the HIV transition metrics suggest increased efforts are needed to bring the HIV epidemic under control.

## 1 Introduction

In 2014, the United Nations Programme on HIV and AIDS introduced the 90‐90‐90 targets by 2020, 90% of people living with HIV (PLHIV) will be diagnosed, of those diagnosed 90% of people will be receiving antiretroviral treatment and among those receiving treatment, 90% will be virally suppressed 1. While it is arguable whether the 90‐90‐90 targets should be a primary target for a healthcare system, in so far as they do not distort rational resource allocation between healthcare options in countries, such targets can have a galvanizing effect and much progress has been made to meet them. However, there is still room for further advancement. In Zimbabwe, a 2016 population‐based survey (Zimbabwe Population‐Based HIV Impact Assessment (ZIMPHIA)) suggests that a quarter of PLHIV were not aware of their HIV status in 2016 2, although there is likely to be some under‐reporting of HIV diagnosis 3. Of those reporting having been diagnosed, 86.8% self‐reported use of antiretroviral therapy (ART) and, among these people, 86.5% had viral suppression 2. The 90‐90‐90 target amounts to 73% of PLHIV achieving viral suppression by 2020. In ZIMPHIA, the corresponding estimate was 60.4% 2.

Prevalence rates (henceforth referred to as ‘prevalence’) and incidence estimates are crucial to understand the current status of the HIV epidemic and, in particular, to identify whether trends suggest an improving epidemic, as was intended to be achieved by the 2020 Joint United Nations Programme on HIV/AIDS (UNAIDS) targets. Estimates from UNAIDS suggests that adult HIV prevalence and HIV incidence in Zimbabwe have decreased considerably since 1998 4, although for HIV prevalence, the level now appears to be stable at around 14% in 2016, as reported by nationally representative surveys (DHS 2015/6 (13.8%), ZIMPHIA 2016 (14.0%)) 2, 5. Estimates based on the Spectrum model also suggest that HIV incidence has continued to fall since 1998 4 and, in 2017, the ZIMPHIA reported an HIV incidence of 0.45 per 100 person‐years among 15 to 64 year olds.

A recent report has outlined HIV transition metrics which can be considered in defining “epidemic control” and therefore aid understanding of the current HIV epidemic 6. It would be useful to see the results of these measures in the context of Zimbabwe, in order to have a more robust understanding of the current epidemic.

Using the HIV Synthesis Model 7, we here aim to mimic the epidemic in Zimbabwe and compare outputs from this individual‐based model to a range of observed data and describe recent trends in the HIV epidemic. We also project future trends in key indicators to 2020 with the aim of assessing whether Zimbabwe is on track to meet the 90‐90‐90 targets by 2020. Finally, we calculate the HIV transition metrics to assess the extent to which they appear to be consistent with the 90‐90‐90 targets.

## 2 Methods

We used an individual‐based model of HIV transmission, progression and the effect of ART in the adult population in Zimbabwe which has been previously described 7. In brief, each time the model programme is run it simulates values of variables for the number of short‐term condomless sex partners, the presence of a long‐term condomless sex partner, HIV testing, HIV acquisition and, additionally, in PLHIV, viral load, CD4 count, use of specific ART drugs, adherence to ART, resistance to specific drugs and risk of HIV‐related death, each updated in three‐month time steps from 1989.

For the purposes of this paper, the model was calibrated to data from Zimbabwe. An approximate Bayesian computation 8 approach was used; this involves sampling model parameter values from various possible values, represented by prior distributions (see Data S2, Table 1) and assessing how closely the model outputs concur with observed data on the real‐life HIV epidemic and ART programme in Zimbabwe. Observed data were obtained from: ZIMPHIA 2, DHS 5, Global AIDS Response Progress Reports 9, WHO Resistance Report 10, Zimbabwe Ministry of Health 11, 12, published papers 13, 14 and UN Reports 15.

**Table 1:** Comparison of latest available observed and modelled data for components of the original c‐score

Components included in the original calibration score	Observed data			Modelled estimate across runs Median (90% range)
	Source	Year of comparison	Estimate (95% CI) %	
Prevalence – men 15 to 49	ZIMPHIA 2	2016.25	0.11 (0.10, 0.12)	0.10 (0.08, 0.13)
Prevalence – women 15 to 49	ZIMPHIA 2	2016.25	0.17 (0.16, 0.17)	0.16 (0.13, 0.21)
Prevalence – men 15 to 24	DHS 5	2015.75	0.03	0.02 (0.01, 0.04)
Prevalence – women 15 to 24	DHS 5	2015.75	0.07	0.05 (0.02, 0.08)
Prevalence – FSWs 15 to 65	Cowan 2017 13	2013.75	0.58 (0.43, 0.79)	0.47 (0.26, 0.72)
Incidence per 100 person‐years – men 15 to 49	ZIMPHIA 2	2016.25	0.28 (0.06, 0.50)	0.50 (0.20, 0.96)
Incidence per 100 person‐years – women 15 to 49	ZIMPHIA 2	2016.25	0.67 (0.37, 0.97)	0.76 (0.27, 1.53)
Number of HIV tests – all 15 to 49	GARCPR 9	2016.5	22,01,246	23,59,360 (17,06,740, 30,28,040)
Proportion diagnosed of those with HIV – men 15 to 64	ZIMPHIA 2	2016.25	0.697	0.77 (0.70, 0.84)
Proportion diagnosed of those with HIV – women 15 to 64	ZIMPHIA 2	2016.25	0.771	0.92 (0.87, 0.96)
Number on ART^3^ – men 15 to 64	MoH 11	2015.75	3,01,650	2,70,100 (2,09,875, 3,38,720)
Number on ART^3^ – women 15 to 64	MoH 11	2015.75	5,16,557	5,23,775 (4,08,435, 6,44,590)
Number on 2nd line – all 15 to 64	MoH 12	2014.75	12,696	14,965 (0, 36,135)
Proportion suppressed of those with HIV – men 15 to 64	ZIMPHIA 2	2016.25	0.53 (0.51, 0.58)	0.57 (0.48, 0.64)
Proportion suppressed of those with HIV – women 15 to 64	ZIMPHIA 2	2016.25	0.64 (0.62, 0.67)	0.67 (0.60, 0.71)
Proportion with resistance at ART initiation – all 15 to 64	WHO Resistance report^a^^,^ b 10	2010.5	0.09 (0.03, 0.22)	0.04 (0, 0.10)
Number of FSW 18 to 49	Cowan^14^	2017.25	73,270^b^	1,27,385 (60,225, 2,00,020)
Number of pregnancies 15 to 49	UN 15	2013.5	3,98,474	4,58,440 (2,10,240, 7,65,040)
Other variables of interest				
Proportion suppressed of those on ART – men 15 to 64	ZIMPHIA 2	2016.25	0.84	0.88 (0.80, 0.94)
Proportion suppressed of those on ART – women 15 to 64	ZIMPHIA 2	2016.25	0.88	0.86 (0.81, 0.92)
Proportion with resistance at ART initiation – all 15 to 64	WHO Resistance report 28	2016.5	0.11 (0.07, 0.16)	0.07 (0, 0.18)
Number of circumcisions per year	GARCPR 9	2015.5	1,06,286	1,16,800 (89,060, 1,44,540)

ART, antiretroviral therapy; FSW, female sex workers; ZIMPHIA, Zimbabwe Population‐Based HIV Impact Assessment.

^a^WHO Resistance Report 2017 was published after completion of the calibration process hence was not included in the calibration score; ^b^see “Description of Model Calibration” document for FSW calculation.

### 2.1 Calibration score

Our approach was to derive a calibration score that conveys the average proportionate deviance of the modelled outputs from the observed data over a range of data items. Details of the calibration, including the number of data items included in the original calibration score are given in Data S2.

The aim of the calibration process was to find 500 parameter sets that yielded an overall calibration score of <0.30. Model outputs for all items included in the score and other outputs of interest were compared with observed data for runs in which the score was <0.3.

In the second phase of the analyses, we made projections to 2020 to describe trends in prevalence, incidence, proportion diagnosed, proportion on treatment, proportion virally suppressed and proportion with resistance. Trends in the number of PLHIV, number of new infections per years, number of new diagnoses per year and number of AIDS deaths per year were also described. We reported medians and 90% ranges, calculated by taking the 10th and 90th centile across the range of runs used. To make these projections, each time the model was run one of the 500 parameter sets that had a calibration score below 0.30 was selected at random and used to replace the original random probability distributions for these model parameters. Each parameter set had an equal probability of being chosen. We ran the model 1500 times and again only selected runs for which the calibration score was <0.3. This latter criterion was not fulfilled for all runs, despite using the 500 selected parameter sets due to stochastic variation.

We considered three alternative methods of calculating the calibration score. This was to explore the implications of favouring different data items within the score; the rationale for each alternative score is described in Data S3. When describing the trends between 2017 and 2020, we assumed no change in national ART regimens and a constant rate of testing (which entailed 20% men and 30% women tested in the last year in 2017 (see Data S2: Description of Model Calibration, page 32)). We also assumed all people were eligible for ART at HIV diagnosis during this time, as per current guidelines 16. Finally, we assumed that eligibility for, and rate of, circumcision remained unchanged (all men aged 15 to 49 were eligible for circumcision determined by age‐specific probability of being circumcised, if not previously diagnosed with HIV). Details of specific rates can be found in Data S1 (Material: Calibration to Zimbabwe, Interventions and populations, page 43). We do not explicitly model transactional sex within the model. However, we hypothesize that both the number and epidemic trends among women with greater than three condomless sex partners in the model will be similar to the number and epidemic trends among women engaged in sex work in Zimbabwe. We are aware of the complexities in explicitly modelling sex work but feel our approach allows a useful comparison between modelled phenomena and observed data.

Finally, we calculated the percentage reduction in new HIV infections and AIDS‐related mortality from 2010 to 2020, the absolute rate of new infections and AIDS‐related mortality, the incidence–mortality ratio and incidence–prevalence ratios. These outputs were compared to the thresholds in the recently published UNAIDS document 6 focussing on defining HIV transition metrics which can be used to consider whether the HIV epidemic is under control. In addition, we calculated four other outputs of interest: total number of adults living with HIV; total number of new infections per year; total number of new diagnoses per year; and the total number of AIDS‐related deaths per year.

## 3 Results

Of the 500 runs that had a c‐score of <0.3, the median c‐score was 0.27 (90% range: 0.22, 0.29).

Comparison of the modelled data items using the original score with the latest available observed data is shown in Table 1. The modelled estimates for incidence of HIV (median over 500 runs: 0.50/100 person‐years (90% range: 0.20, 0.96) for men and 0.76/100 person‐years (0.27, 1.53) for women) and proportion of HIV‐positive people diagnosed (0.77 (90% range: 0.70, 0.84) for men and 0.92 (0.87, 0.96) for women) were higher than the observed data (0.28/100 person‐years for men, 0.67/100 person‐years for women and 0.70 for men and 0.77 for women respectively 2). However, these modelled values were a result of a good fit of all other items in the score and the extent to which the observed data are underestimates is unclear. The modelled estimate for number of female sex workers (FSWs) was also higher than the observed data, though the observed data did fall within the 90% modelled range. Table 1 also shows selected comparisons between other data items of interest and, in particular, the proportion with viral suppression of those on ART. Again, there is a good concordance between observed data and model outputs; 84% of males have viral load suppression while on ART according to the ZIMPHIA survey, compared to 88% in our model outputs and the respective proportions for females are 88% (observed) and 86% (modelled). Further comparisons with a broad range of data items are given in the Description of Model Calibration document.

**Figure 1:**
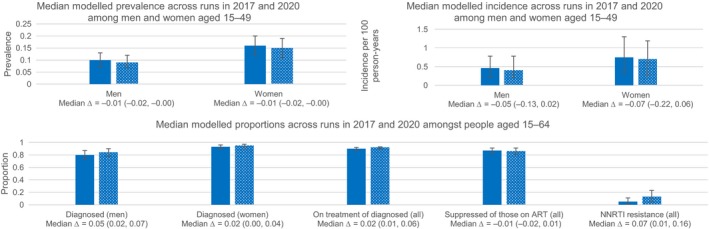
Median (90% range) modelled projections at 2017 and 2020

In the second phase of the analyses, we projected forward to 2020 and analysed results at 2017 and at 2020 (Figure 1). We predict little change in the prevalence of HIV from 2017 to 2020, though predict incidence to fall by a mean of 0.06/100 person‐years, albeit with substantial uncertainty in this trend (90% range: −0.16, 0.03). With continuation of current testing rates, the overall predicted proportion of PLHIV diagnosed by 2020 is estimated to be 0.91 (90% range: 0.87, 0.94). We predict a mean 0.05 absolute increase (90% range: 0.02, 0.07) in the proportion of men diagnosed and a 0.02 absolute increase (90% range: 0.00, 0.04) in the proportion of women diagnosed, resulting in an overall proportion of 0.84 (90% range: 0.78, 0.90) of men diagnosed with HIV and 0.95 (90% range: 0.91, 0.97) of women diagnosed in 2020. The proportion of HIV‐positive people diagnosed with HIV receiving ART is expected to increase by 0.02 (90% range: 0.01, 0.06), resulting in 0.92 (90% range: 0.90, 0.93) of diagnosed people on treatment in 2020. Of those receiving ART, there is minimal predicted change in the proportion of people with viral suppression. The overall proportion of people with viral suppression in 2020 is predicted to be 0.86 (90% range: 0.81, 0.91); 0.87 (90% range: 0.78, 0.92) of men and 0.86 (90% range: 0.82, 0.91) of women had viral load suppression in 2020. Of those aged 15 to 49 living with HIV (undiagnosed as well as diagnosed), 0.37 (90% range: 0.31, 0.43) had unsuppressed viral load in 2017, compared to a predicted 0.30 (90% range: 0.25, 0.36) in 2020. The proportion of people with non‐nucleoside reverse transcriptase inhibitors (NNRTI) resistance at time of starting ART is expected to increase markedly in three years time, from 0.05 (90% range: 0.01, 0.13) in 2017 to 0.13 (90% range: 0.05, 0.23) in 2020; a change of 0.07 (0.01, 0.16). Modelled estimates in 2017 and in 2020 using the three alternative calculations were in line with those calculated using the original score and are shown in Data S3.

There was little difference in the projected number of PLHIV: 1,130,770 (90% range: 881,840, 1,394,665) in 2017 and 1,130,305 (90% range: 875,303, 1,431,397) in 2020; or the number of new infections per year: 41,610 (90% range: 18,615, 67,890) in 2017 and 40,515 (90% range: 17,155, 66,795) in 2020. The number of new diagnoses per year was 58,400 (90% range: 32,120, 90,520) in 2017 and 43,800 (90% range: 22,165, 70,611) in 2020. The number of AIDS‐related deaths per year was similar (17,520 (90% range: 8760, 29,200) in 2017 and (18,051 (90% range: 11,680, 25,882); change of 0 (95% range: −8893, 78310).

Finally, we calculated a modelled percentage reduction in new infections from 2010 to 2020 of 36.5% (90% range: 13.7% increase to 67.4% reduction), and in AIDS‐related mortality of 30.4% (90% range: 9.7% increase to 56.6% reduction. The interim impact target for both these measures agreed by the United Nations Assembly was a 75% reduction. The absolute rates of modelled incidence and AIDS‐related mortality in 2020 were 5.48 (90% range: 2.26, 9.24) and 1.93 (1.31, 2.71) per 1000 person‐years respectively. The modelled incidence–mortality ratio and incidence–prevalence ratios in 2020 were 1.05 (90% range: 0.46, 1.66) and 0.009 (90% range: 0.004, 0.013) respectively.

## 4 Discussion

Using an individual‐based model and a range of observed data in Zimbabwe, we were able to mimic the current HIV epidemic and project future trends in key indicators, including the 90‐90‐90 targets. The UNAIDS 90‐90‐90 targets in 2020 are within reach according to our projected trends, given increased efforts in diagnosing men in particular. While we predict 90.7% of adults with HIV overall will be diagnosed in 2020, the respective estimates for men and women are 84% and 95%. We estimate that 92% of people diagnosed will be receiving treatment, and of these, 86% will be virally suppressed (although may potentially be biased due to people under‐reporting treatment). Ninety per cent is within our uncertainty range; and continued commitment to the Extended Zimbabwe National HIV and AIDS Strategic Plan 17 may increase this estimate further.

The United Nations Assembly have suggested a target of a 75% reduction in new infections and AIDS‐related deaths between 2010 and 2020. In Zimbabwe, the percentage reduction for both these modelled outputs was <30%, with wide variability around the estimates suggesting that we cannot rule out a percentage increase. While this transition metric does not take into account the achievements made prior to 2010, it does suggest that there is still considerable work to be done to reduce the number of new infections and AIDS‐related deaths. The modelled absolute rates of HIV incidence and AIDS‐related deaths in 2020 were both above 1/1000 person‐years. Rates below 1/1000 person‐years have been used to define elimination of HIV 18, and UNAIDS have also suggested that rates below 1/1000 person‐years could be relevant in higher incident and prevalence settings 6. Potential issues with our projected incidence measure are discussed later in this section and so though it is considerably higher than 1/1000 person‐years our model may not reflect the true incidence. Our modelled incidence–mortality ratio was 1.05. UNAIDS do state a benchmark of one for this transition metric; however, this is only relevant with a measure of low mortality among PLHIV or high ART coverage, defined as >81% of PLHIV (P Ghys, personal communication). Zimbabwe, according to the ZIMPHIA estimates, does not currently meet the 81% ART coverage. However, using estimates from our model, we predict 84% ART coverage and in this case, the incidence–mortality ratio implies that there are more new infections than deaths per year. Hence, it is likely that, despite the reduction in prevalence, the number of PLHIV is on the rise, due to population growth. The modelled incidence–prevalence ratio takes into account duration of life after HIV acquisition. Our projected output was below the target suggested, of 0.03, based on an average survival of people with HIV of 33 years after acquisition. This transition metric suggests that the total number of PLHIV will eventually fall, and hence, the epidemic is in a “state of control.” This interpretation is not in line with the interpretation of the three metrics prior to this (percentage reduction in new infections/AIDS‐related deaths, the absolute rates of new infections/AIDS‐related mortality and the incidence–mortality ratio) which suggest that increased efforts are needed to bring the HIV epidemic under control. This also suggests that even if the 90‐90‐90 targets were achieved, the HIV transition metrics are unlikely to all be met, and hence, there may be a lack of consistency between the two sets of targets, certainly in the short term.

Our modelled estimate of prevalence of HIV in women who had greater than three condomless sex partners in the past three month period was lower than observed data among FSWs 13. This may be due to differences in the make‐up of these two groups: for example, our modelled estimate for the number of FSWs was markedly higher than the empirical estimate on the number of FSWs, which itself is difficult to accurately estimate. To reflect this uncertainty, we allowed substantial variability over model runs in sexual behaviour parameter values which determine the number of women with greater than three condomless sex partners within the model.

When projecting forward to 2020 using the original calibration score, HIV incidence was projected to drop by 0.06 per 100 person‐years. Given the uncertainty around the incidence estimate, the degree of decrease is difficult to accurately quantify. We project that by 2020, the proportion of people diagnosed with HIV will increase, particularly in men, assuming the rate of testing remains at the current level. This may be due to a lower proportion of men diagnosed in 2017 compared to women, and hence, there is more scope for this estimate to increase. It is hoped that these estimates may increase even further, in line with increased efforts in testing for HIV in men, within for example the circumcision programme currently underway in Zimbabwe 19 and with the planned rollout of HIV self‐testing which is likely to attract a significant proportion of men (TA, Zimbabwe MoH, personal communication). The proportion of people with NNRTI resistance at ART initiation is also projected to increase by 9% to an overall 15% by 2020. This is of particular concern in this setting, given the limited access and availability of alternative first line regimens. Adherence counselling and efforts to increase testing have, and are currently taking place but continued effort in these areas is needed to ensure transmitted drug resistance does not become an issue that is unable to be effectively addressed. WHO guidelines have changed the national first line ART regimen in new initiators to be dolutegravir, rather than efavirenz, containing in countries with pre‐ART NNRTI resistance >10% 20, and it is predicted this change would be both effective and cost‐effective 21. The Zimbabwe treatment guidelines have not yet incorporated this change 16. Recent data suggesting a potential risk of neural tube defects in women who are on dolutegravir at the time of conception create doubt over the timing and nature of dolutegravir introduction 22.

Generally, the estimates obtained using the alternative calibration scores are in line with our original calibration score, suggesting that the weights and data items used in our original score are robust. This implies that if the assumptions hold (in terms of testing, number of circumcisions, number of people receiving treatment, etc.) our projections to 2020 are likely to be in line with observed data in the future or may even be an underestimation of the potential benefits, particularly since there are ongoing efforts to improve the status of the HIV epidemic in Zimbabwe.

When comparing observed and modelled outputs, we found a good fit across the majority of components included in the score. We did however see a higher predicted HIV incidence (within the 95% observed CI for women but not for men) and a higher predicted proportion diagnosed compared to those reported in ZIMPHIA. There is particular uncertainty over the current incidence since the only data source to inform this is the recent ZIMPHIA estimate 2, which was calculated using the recent infection algorithm 23. There are concerns of using a single method to estimate incidence 24; hence, the ZIMPHIA estimate should be interpreted with caution. In contrast, the prevalence estimates are informed by three DHS surveys as well as ZIMPHIA 5, 25, 26, and we did indeed see a good fit when comparing our modelled prevalence data to observed data. Our estimate for proportion of PLHIV who have been diagnosed is higher than that seen in ZIMPHIA 2, which was the only data source available for this item. Our modelled estimate was a result of a good agreement of all other components of the score. Furthermore, the results from ZIMPHIA on HIV status are based on self‐reported data and hence must be interpreted with caution. It is highly likely that not all people diagnosed with HIV report as having been diagnosed when asked; this has been demonstrated in other surveys 3 and if this is the case, it would result in an underestimation in the proportion diagnosed.

## 5 Conclusions

It is essential to have reliable observed data in order to calibrate models well to specific settings and hence accurately replicate past trends in order to predict future trends. While our score proved to be robust, there are some data items that are both difficult to model and obtain reliable data on. Surveys like the DHS and ZIMPHIA are invaluable in terms of the information they provide and are a good indication of whether treatment programmes are working. However, it is likely that some of the outputs reported in these surveys, for example, the proportion of people diagnosed with HIV, and the proportion of people receiving treatment are underestimated due to stigma surrounding a HIV diagnosis and hence failure to disclose status. Further data collection, perhaps in the form of complete healthcare provider records, together with commitment to the Monitoring and Evaluation Plan as outlined in the National Strategic Plan 17 would be necessary to fully understand the impact of HIV in Zimbabwe and work towards achieving and surpassing the 90‐90‐90 UNAIDS target. Considerable efforts are needed to reach the benchmarks suggested by the UN when calculating the HIV transition metrics, and in particular, to achieve the recommended 75% reduction in the number of new HIV infections. It is important to recognize that while continued efforts in testing are likely to reduce the number of people with undiagnosed HIV, the proportion of these people that are hard to reach is likely to increase and hence increased resources may be required for outreach testing to maintain current testing rates. Furthermore, while continued efforts are needed to diagnose and treat those with HIV, resources also need to be used to maximize prevention of HIV 27, provided that the use of such resources is cost‐effective.

## Competing Interest

Frances Cowan received a donation of Truvada from Gilead Health Sciences as the Principal Investigator for a PrEP Demonstration Project incorporated in the SAPPH‐IRE trial.

## Authors’ Contribution

All authors have read and approved the final manuscript. LBM performed the analyses and wrote the paper. VC and AP designed the research question, extensively reviewed the analyses and the paper. TA, RY, JD, SM, CD, SN, EF, AM, OM, JRH and FC reviewed the paper.

## Supporting information


**Data S1.** Calibration to Zimbabwe.

**Data S2.** Description of model calibration.

**Data S3.** Alternative methods of calculating the calibration score.
