# Supplementary material for: 90‐90‐90 by 2020? Estimation and projection of the adult HIV epidemic and ART programme in Zimbabwe – 2017 to 2020
Source: J Int AIDS Soc. 2018 Nov 22;21(11):e25205. doi: 10.1002/jia2.25205 (PMC6250855; doi:10.1002/jia2.25205)
Supplement: Supplementary file 1 — Data S1. Calibration to Zimbabwe. [file JIA2-21-e25205-s001.docx]

**S1: Supplementary Material: Calibration to Zimbabwe**

**Dec 2017**

Contents

[1. Demographic model 2](#_Toc525313261)

[2. Sexual behaviour and risk of HIV acquisition 4](#_Toc525313262)

[3. Natural history of HIV infection 15](#_Toc525313263)

[4. HIV testing and diagnosis of HIV infection 17](#_Toc525313264)

[5. Modelling the effect of ART 19](#_Toc525313265)

[6. Emergence of specific resistance mutations and their effect on drug activity 39](#_Toc525313266)

[7. Interventions and populations 43](#_Toc525313267)

[8. Risk of clinical disease and death in HIV infected people 45](#_Toc525313268)

# Demographic model

**General population death rates and determination of age in 1989**

The model runs to from 1989 (*as this is reasonably close to when the first heterosexual AIDS cases were reported in South Africa (1987) (Van der Vliet, 2004) and Zimbabwe (1985) (Dehne, Tropical Doctor 1992)* to 2039 (although in our results we concentrate on a significantly shorter period), with variables updated in 3 month periods. Each run of the simulation program creates 100,000 simulated people who will be age 15 or above at some point between 1989 and 2039, of whom approximately 50,000 are alive and age over 15 at any one point in time. In order to scale up from the simulated population to the actual population in Zimbabwe we use a scale factor of 365.

In the absence of data on death rates considered reliable for Zimbabwe, age specific death rates for uninfected people are based on death rates in South Africa in 1997 (Table 1.1) – before the significant impact of HIV-related deaths. These rates were modified by a factor (randomly assigned as either 1-fold, 1.5-fold or 2-fold) with 33.3% probability ) in order to mimic the population pyramid in Zimbabwe (Table 1.2)

Table 1.1 Age specific death rates (per year)

| Age group | Annual death rate | Age group | Annual death rate |
| --- | --- | --- | --- |
| Males |  | Females |  |
| 15 – 19 | 0.00400 | 15 – 19 | 0.03000 |
| 20 – 24 | 0.00640 | 20 – 24 | 0.00560 |
| 25 – 29 | 0.01160 | 25 – 29 | 0.00800 |
| 30 – 34 | 0.01500 | 30 – 34 | 0.00800 |
| 35 – 39 | 0.01600 | 35 – 39 | 0.00840 |
| 40 – 44 | 0.02000 | 40 – 44 | 0.01100 |
| 45 – 49 | 0.02400 | 45 – 49 | 0.01500 |
| 50 – 54 | 0.03800 | 50 – 54 | 0.02200 |
| 55 – 59 | 0.05000 | 55 – 59 | 0.03000 |
| 60 – 64 | 0.07000 | 60 – 64 | 0.04200 |
| 65 – 69 | 0.09000 | 65 – 69 | 0.06000 |
| 70 – 74 | 0.11000 | 70 – 74 | 0.07600 |
| 75 – 79 | 0.13000 | 75 – 79 | 0.10000 |
| 80 – 84 | 0.20000 | 80 – 84 | 0.14000 |
| >85 | 0.80000 | >85 | 0.30000 |

Table 1.2 Observed and modelled population size by age in 2017.

|  | Male | | Female | |
| --- | --- | --- | --- | --- |
|  | Observed | Model | Observed | Model |
| 15 – 24 years | 1.56m | 1.56m | 1.54m | 1.54m |
| 25 – 54 years | 2.58m | 2.25m | 2.35m | 2.66m |
| 55 – 64 years | 0.19m | 0.22m | 0.33m | 0.41m |
| 65 years and over | 0.19m | 0.13m | 0.31m | 0.37m |

Observed data from CIA world factbook (2017) <https://www.cia.gov/library/publications/the-world-factbook/geos/zi.html>

The initial age distribution for both males and females is determined on the basis of the distribution in Table 1.3.

Table 1.3 Distribution of ages of simulated individuals in 1989

| Age group | Probability of being in age group in 1989 |
| --- | --- |
| -65 to -56 | 0.150 |
| -55 to -46 | 0.130 |
| -45 to -36 | 0.120 |
| -35 to -26 | 0.110 |
| -25 to -16 | 0.100 |
| -15 to -6 | 0.090 |
| -5 to 4 | 0.080 |
| 5 to 14 | 0.0650 |
| 15 to 24 | 0.048 |
| 25 to 34 | 0.040 |
| 35 to 44 | 0.030 |
| 45 to 54 | 0.021 |
| 55 to 64 | 0.016 |

This distribution is chosen such that in the absence of HIV, given the death rates above, the population size increases over time. Thus around 72% of simulated people have an age below 15 in 1989. The only variable that is modelled and updated up to reaching the age of 15 (when becoming potentially sexually active) is age itself. The “youngest” person in 1989 is age -35 (i.e. will be born in 2024 and reach age 15 in 2039, when the modelled period ends.

# Sexual behaviour and risk of HIV acquisition

Here we describe the approach to modelling sexual behaviour and HIV acquisition. The basic approach is summarized in Figure 2.1. The parameter values related to sexual behaviour were chosen such that they lead to a modelled HIV prevalence level over time as observed (see *Description of Model Calibration* document). Sexual behaviour is characterized by two variables representing, respectively, the number of short term condomless sex partners and whether the person has a current long term condomless *sex* partners in the 3 month period. The status of long term partners is tracked over time (i.e. if they are infected, diagnosed, on ART). Short term partners are not tracked over time, in that if a person has a short term partner in time period t who is infected with HIV, this is independent of the probability that any short term partner in time t+1 is infected with HIV.

Figure 2.1
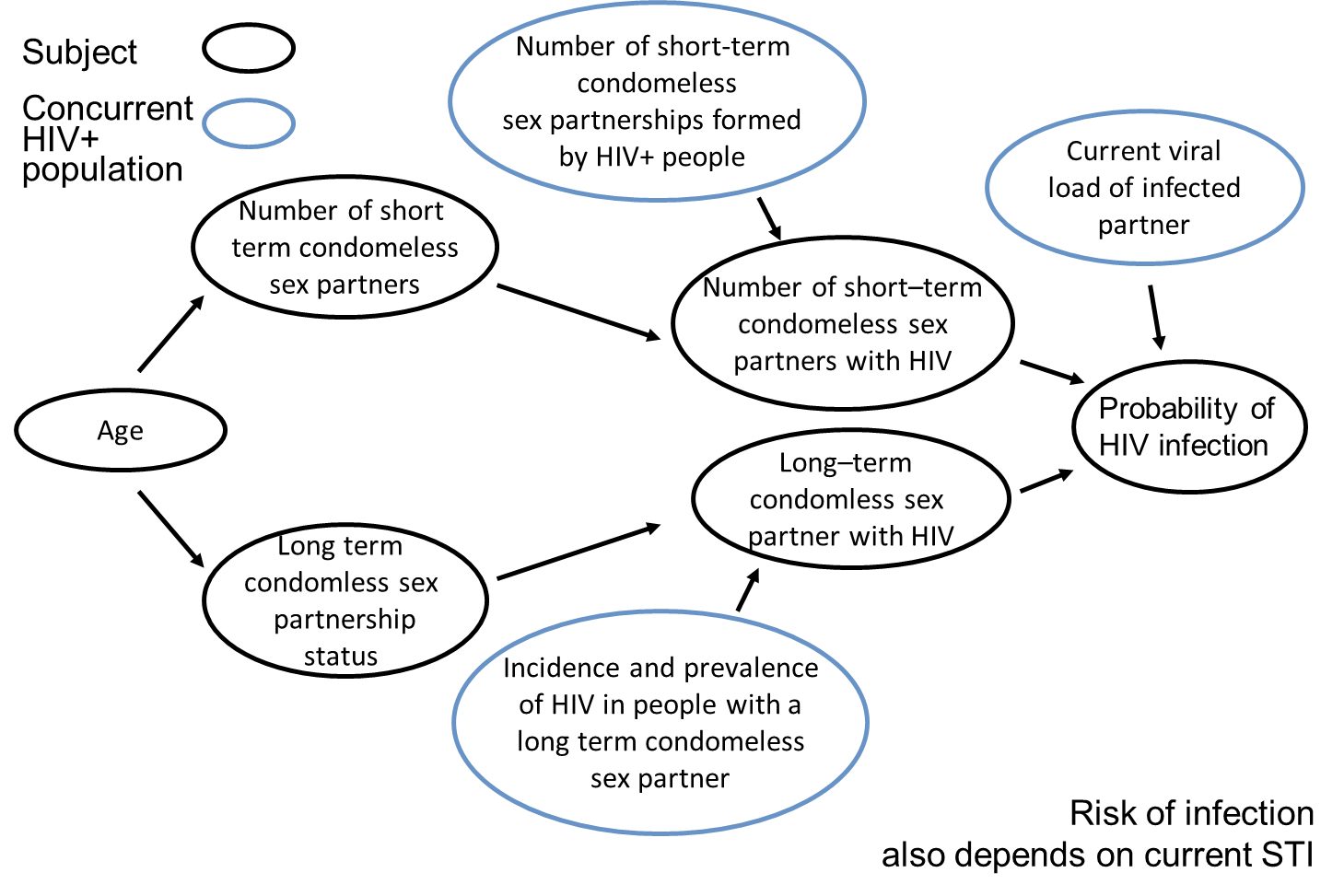
 Summary of modelling of sexual behaviour and HIV acquisition

**Determination of number of short term (condomless sex) partners at period t**

Numbers of short term partners in a given period was generated at random, according to which of four sexual behaviour groups the person was in for this period. Changes in the sexual behaviour group from t-1 to t were determined by transition probabilities between 4 groups: (i) no short term condomless partners in 3 month period, (ii) 1 short term partner, (iii) medium number of short term partners, and (iv) high number of short term partners. Transition probabilities $p_{gija}$ of moving from partner group i at t-1 to partner group j at t are given by

$p_{\mathrm{gija}}=\frac{f_{\mathrm{gij}}}{\left( f_{gi1}+\sum_{j=2}^{4} \left( f_{\mathrm{gij}}\cdot r_{\mathrm{ga}} \right) \right)} \text{ for }j=1$ $p_{gija}=\frac{f_{gij}\times r_{ga}}{\left( f_{gi1}+\sum_{j=2}^{4} \left( f_{gij}\cdot r_{ga} \right) \right)}$

where g = 0,1 for males, females, respectively, and a = 1-10 for age groups 15-, 20-, 25-, 30-, 35-, 40-, 45-, 50-, 55-, 60-, respectively. Values of $f_{gij}$ and $r_{ga}$ are given in Table 2.1 and Table 2.2, and if j=1 then $r_{ga}$=1.

Values of $r_{ga}$ are modified at time t by a factor 0.2 if the subject has a current AIDS defining disease and by a factor *ch_risk_diag_newp* (determined by sampling from a Beta distribution [Beta (12, 2)]), informed by (Fonner et al 2012) if the subject is diagnosed with HIV (sqrt(*ch_risk_diag_newp* from 6 months after diagnosis). In addition, there is a person-fixed modification factor (*p_rred_p =* Uniform(0.1, 0.5)). For a proportion *p_rred_p* of men and 1.5 x *p_rred_p* of women, values of $r_{ga}$ are modified by a factor 0.1, to reflect the fact that a proportion of people experience only very low sexual risk activity in their life.

Actual transitions between groups were determined by random sampling. For the first two groups, the number of partners in the period is given (i.e. no short term partners, 1 short term partner, respectively). When a person was in the medium short term partners group the number of partners was determined by sampling from a Uniform (3, 10) distribution (*highsa*), where median *highsa* = 6.5. When in the high short term partners group the number of partners was determined by sampling from a Poisson (2) distribution and multiplied by the parameter *swn, uniformly distributed (Uniform (4, 25) (median= 14.5*).

Table 2.1 Values of $\boldsymbol{f}_{\boldsymbol{gij}}$ (values determining probability of transitioning between short term partner risk behaviour groups)

|  | **Short term partners group in period t** | | | |
| --- | --- | --- | --- | --- |
| **Short term partners group in period t-1** | 0 | 1 | Medium (Uniform (3, 10)*) | High (Poisson mean 2 x *swn**) |
| Males |  |  |  |  |
| 0 | 0.89 | 0.08 | 0.03 | 0.00 |
| 1 | 0.80 | 0.15 | 0.05 | 0.00 |
| Medium | 0.35 | 0.27 | 0.38 | 0.00 |
| High | 0.20 | 0.30 | 0.50 | 0.00 |
| Females |  |  |  |  |
| 0 | 0.93 | 0.05 | 0.02 | 0.00020 |
| 1 | 0.86 | 0.11 | 0.03 | 0.00035 |
| Medium | 0.54 | 0.08 | 0.38 | 0.0007 |
| High | 0.02 | 0.02 | 0.06 | 0.900 |

* *highsa* = median 6.5, *swn* = median 14.5

Table 2.2 Values of $\boldsymbol{r}_{\boldsymbol{ga}}$ (factor determining relative level of sexual risk activity)

| **Age group (a=1,10)** | **Males (g=1)** | **Females (g=2)** |
| --- | --- | --- |
| 15- | 0.60 | 1.80 |
| 20- | 0.60 | 1.80 |
| 25- | 1.00 | 1.00 |
| 30- | 0.80 | 0.80 |
| 35- | 0.65 | 0.50 |
| 40- | 0.50 | 0.35 |
| 45- | 0.40 | 0.10 |
| 50- | 0.35 | 0.05 |
| 55- | 0.25 | 0.04 |
| 60- | 0.15 | 0.02 |

**Determination of having a long term (condomless sex) partner at period t**

Note that only condomless sex partnerships are modelled. Thus if a person has a long term partner but condoms are used on all occasions of sexual intercourse then this is not counted as having a long term condomless sex partner.

At each period, people with no current long term partner have age-dependent probabilities of having a new long term partner and these are dependent on parameter *eprate*, Log normal (ln 0.10, 0.25) and given by: age 15-24, p= *eprate*; age 25-34, p= *eprate*; age 35-44, p= *eprate/2*; age 45-54, p= *eprate/3*; age 55-64, p= *eprate/5*

At the time a long term partnership is started, it is classified into 3 duration groups, each with a different tendency to endure. The percent of people in each group is dependent on age and is shown in Table 2.3.

At time period, t, for people with a long term partner, the probability of the condomless sex partnership continuing is (1-(0.25 / *ch_risk_beh_ep*)) if duration category is 1, is (1-(0.05 / *ch_risk_beh_ep*)) if duration category is 2, and (1-(0.02 / *ch_risk_beh_ep*)) if duration category is 3, where *ch_risk_beh_ep* is a parameter conveying the population level change in sexual behaviour with long term partners that occurs in 1995 (*ch_risk_beh_ep* = 1-((caldate{t}-1995)*ych_risk_beh_ep between 1995 and 2000, depicting a gradual linear decline over this period and = 1-((2000-1995)*ych_risk_beh_ep after the year 2000)*.* Further, this probability is reduced by a factor *ch_risk_diag* in the 3 month period after a partner’s diagnosis, if a partner has HIV and is diagnosed. (*ch_risk_diag* = Beta (12,2), median 0.87).

Note also that levels of sexual behaviour, in terms of numbers of short term partners and the probability of a long term partner are essentially determined by the levels of such sexual behaviour required in order to produce an epidemic as described, given rates of transmission with condomless sex partners. Sexual behaviour tends to be under-reported particularly in women and higher levels of behaviour have to be assumed both to be consistent with levels of risk behaviour reported in men, and to generate an epidemic of the proportions observed (e.g. Gregson 20022, Johnson 2009). Nonetheless, reported sexual behaviour, particularly in terms of differences by age in males and females have been referred to (e.g. Zimbabwe DHS 2004, Zimbabwe DHS 2011).

Table 2.3 Percent of newly formed long term partnerships classified into each of three duration groups, each of which has a different tendency to endure (higher class, more durable).

| **Age** | **1** | **2** | **3** |
| --- | --- | --- | --- |
| 15-44 | 30% | 30% | 40% |
| 45-54 | 30% | 50% | 20% |
| 55-64 | 30% | 70% | 0% |

**Population level change in sexual behaviour**

There is assumed to be a general average reduction in condomless sex after 1995, reflecting the reductions observed over the period from around this date (Gregson 2010, Halperin 2011).

**Determination of number of short term (condomless sex) partners who are HIV infected at time t**

For each short term partner that a subject has at time t, the probability that the partner is infected is calculated. This is dependent on the prevalence of HIV in those of the opposite gender, taking consideration of age mixing. If the subject is of gender g and age group a, then for each short term partner the first step is to determine by sampling at random, the age group of the short term partner, $a^{\text{newp }}$(in fact, for simplicity, all short term partners at time t are assumed to be in this same age group). The gender and age mixing probabilities used are given by values in Table 2.4.

Table 2.4 Sexual mixing by age and gender. The proportion of short term partnerships formed by men in age group a_m_ which are with females of age group a_f_ and the proportion of short term partnerships formed by females in age group a_f_ which are with men of age group a_m_.

|  | **Female age groups (a_f_)** | | | | |
| --- | --- | --- | --- | --- | --- |
| **Male age groups (a_m_)** | 15-24 | 25-34 | 35-44 | 45-54 | 55-65 |
| 15-24 | 0.865 | 0.11 | 0.025 | 0.00 | 0.00 |
| 25-34 | 0.47 | 0.43 | 0.10 | 0.00 | 0.00 |
| 35-44 | 0.30 | 0.50 | 0.20 | 0.00 | 0.00 |
| 45-54 | 0.43 | 0.30 | 0.23 | 0.03 | 0.01 |
| 55-64 | 0.18 | 0.18 | 0.27 | 0.27 | 0.10 |

|  | **Male age groups (a_m_)** | | | | |
| --- | --- | --- | --- | --- | --- |
| **Female age groups (a_r_)** | 15-24 | 25-34 | 35-44 | 45-54 | 55-65 |
| 15-24 | 0.43 | 0.34 | 0.12 | 0.10 | 0.01 |
| 25-34 | 0.09 | 0.49 | 0.30 | 0.10 | 0.02 |
| 35-44 | 0.03 | 0.25 | 0.34 | 0.25 | 0.13 |
| 45-54 | 0.00 | 0.00 | 0.05 | 0.25 | 0.70 |
| 55-64 | 0.00 | 0.00 | 0.00 | 0.10 | 0.90 |

Then, for the given partner (of gender 1-g and age group *a^newp^*), the risk that the partner is infected is then given by

$$h_{gat}=\frac{\sum_{a^{\text{newp}},(g-1)} L_{(t-1)}^{\text{inf}}}{\sum_{a^{\text{newp}},(g-1)} L_{(t-1)}}$$

where $L_{(t-1)}^{\text{inf}}$ is the total number of infected short term partners at time (t-1), and $L_{(t-1)}$ is the total number of short term partners at time t-1. The numerator is therefore the total number of infected short term partnerships of the opposite gender in age group *a^newp^_._*

Since we assume that all short term partners at time t are in this same age group, the total number of infected short term partners that the subject has at time t, $L_{t}^{\text{inf}}$, is then given by

$$L_{t}^{\text{inf}}=\text{Min}\left( \text{Poisson}\left( h_{t}\cdot L_{t} \right),L_{t} \right)$$

The distribution of numbers of partners by age and gender is shown in Table I.

**Determination of probability that a long term partner is HIV infected at time t**

$E_{t}^{\text{inf}}$indicates whether the subject has a long term (condomless sex) partner who is infected ($E_{t}^{\text{inf}}=1$ if infected, else $E_{t}^{\text{inf}}=0$). A long term partner at time t can be infected either because (i) a new long term partnership has been formed and the partner was already infected, (ii) because a long term partner at t-1, which has remained a long term partner at time t, has become infected, or (iii) because an infected long term partner has remained as a long term partner.

For (i):

$E_{t}^{\text{inf}}=1\text{ if }L_{(t-1)}^{\text{inf}}\geq1$ (i.e. if the subject had a short term partner at time t-1 who was infected then it is assumed that the new long term partner is infected)

For (ii):

The probability that a long term partner of a subject of age group a and gender g becomes infected is derived from the HIV incidence at t-1 for age group a (i.e. the same age group) and gender 1-g, $i_{a(1-g)(t-1)}$ among the sexually active population, either with a long term partner or at least one short term partner (which is given by the number of subjects newly infected in age group at time *t-1* divided by the number of HIV-uninfected subjects in age group at *t-1*, who had condomless relationships, either long or short term)

$$\left\{ \begin{aligned} E_{t}^{\text{inf}}=1, &U<i_{a\left( 1-g \right)\left( t-1 \right)} \text{where }U \text{randomly sampled from }Uniform(0,1) \\ E_{t}^{\text{inf}}=0, &\text{otherwise} \end{aligned} \right.$$

In order to maintain balance, for each gender, between the number of uninfected people with a long term partner who is infected, and the number of infected people with a long term partner who is uninfected, this incidence $i_{a(1-g)(t-1)}$ is modified at time t dependent on the degree of balance at time t-1.

For (iii):

If $E_{\left( t-1 \right)}^{\text{inf}}=1\text{ and }E_{t}\geq1 \text{then assign }E_{t}^{\text{inf}}=1$

**Determination of the risk of infection from a short term partner**

For each HIV infected short term partner of a subject of gender g and age group a the viral load group, v, of the partner is obtained by sampling from the viral load distribution of those of the opposite gender. Thus we sample from Uniform(0,1), where the probability of the partner having viral load in group v is given by

$$\frac{\sum_{v} L_{(t-1)}^{\text{inf}}}{\sum L_{(t-1)}^{\text{inf}}}$$

where the numerator is the total number of short-term partnerships had by infected people in viral load group v and the denominator is the total number of short-term partnerships had by infected people (in any viral load group).

Viral load groups are:

(1) < 2.7 log cps/mL

(2) 2.7-3.7 log cps/mL

(3) 3.7-4.7 log cps/mL

(4) 4.7-5.7 log cps/mL

(5) > 5.7 log cps/mL

(6) primary infection.

Once the viral load group, v, of the infected partner is determined, the probability, t_v_, of the subject being infected by the partner is then given according to: t_1_ = Normal (*tr_rate_undetec_vl*,0.000025^2^), t_2_ = Normal (0.01,0.0025^2^), t_3_ = Normal (0.03,0.0075^2^), t_4_ = Normal (0.06,0.015^2^), t_5_ = Normal (0.1,0.025^2^), t_6_ = Normal (*tr_rate_primary*,0.075^2^). These are based on Hollingsworth et al (2008) and are the rates for a long term partner. The transmission rate for a short term partner is multipled by fold_tr_newp (0.3) due to the assumed lower number of sex acts. These probabilities are increased by *fold_change_w*, log Normally distributed (ln 1.5, 0.3) (median = 1.5) for female subjects aged > 20, by fold_change_yw for female subjects aged < 20, and by *fold_change_sti*, log normally distributed (ln 3.0, 0.3) (median = 3.0) if the person has an existing STI (risk of a new STI in any one three month period is given by the number of short term condomless partners / 20 (or 1 if > 20 short term partners)) (Cohen et al 1998, Nicolosia 1994).

We assume that super-infection can occur (i.e. a person can be reinfected with HIV with consequent risk of acquiring new mutations).

Realization of whether the subject is infected by each short term partner is determined by sampling from Uniform (0,1).

**Determination of the risk of infection from a long term partner**

Infected long term partners at time t are classified by whether they are in primary infection (if infection occurred at t-1), whether they are diagnosed with HIV, whether they are on ART, and whether their current viral load is < 2.7 cps/mL or not. The proportion of long term partners with HIV who have HIV diagnosed at time t, $p_{t}^{\text{e,diag}}$, is determined with reference to the difference, $d_{(t-1)}^{\text{e,diag}}$, in the proportion of subjects with HIV who are diagnosed, $\frac{T_{(t-1)}^{\text{diag}}}{T_{(t-1)}^{\text{inf}}}$ and $p_{(t-1)}^{\text{e,diag}}$;

i.e. $d_{(t-1)}^{\text{e,diag}}=\frac{T_{(t-1)}^{\text{diag}}}{T_{(t-1)}^{\text{inf}}}-p_{(t-1)}^{\text{e,diag}}$

where $T_{(t-1)}^{\text{diag}}$ is the total number of subjects diagnosed with HIV at time t-1and $T_{(t-1)}^{\text{inf}}$ is the total number of subjects with HIV (diagnosed and undiagnosed) at time t-1.

The number of long-term partners is altered according to the current degree to which the number of diagnosed existing partners (*epdiag*) corresponds to the proportion diagnosed (*p_diag*) in the subjects, by gender.

For each gender separately,

if 0 < $d_{(t-1)}^{\text{e,diag}}$ then j=0

if 0.05 > $d_{(t-1)}^{\text{e,diag}}$ > 0 then j= $p_{(t-1)}^{\text{e,diag}}$/5

if 0.10 > $d_{(t-1)}^{\text{e,diag}}$ > 0.05 then j= $p_{(t-1)}^{\text{e,diag}}$/2

if $d_{(t-1)}^{\text{e,diag}}$ > 0.10 then j= $p_{(t-1)}^{\text{e,diag}}$

if ${ep}_{(t-1)}^{\text{diag}}$ ne **1** then ${ep}_{(t)}^{\text{diag}}$=**0**

s=uniform(**0**)

if s < j then ${ep}_{(t)}^{\text{diag}}$=**1**;

a=uniform(**0)**

if s < **0.9** then ${ep}_{(t)}^{\text{diag}}$ = mr_epdiag{t};

if s >=**0.9** and a < j then ${ep}_{(t)}^{\text{diag}}$=**1**;

where ${ep}_{(t)}^{\text{diag}}$is the total number of long term partners diagnosed at time t.

The proportion of those diagnosed who are on ART, and the proportion of those on ART who have viral load < 2.7 log cps/mL are determined in a similar manner. In this way the proportions diagnosed with HIV, on ART, and with current viral load is < 2.7 log cps/mL are kept similar for the long term partners as in the simulated subjects themselves.

Risk of infection from a long term infected partner is determined by Normal (*tr_rate_primary*, 0.075^2^) if the existing partner is in primary infection (ie. infected at t-1), Normal (*tr_rate_undetec_vl*, 0.000025^2^) if the existing partner has viral load < 2.7 log cps/mL, and Normal (0.05, 0.0125^2^) otherwise.

**Transmitted resistance: overview**

The modelling of transmission of drug resistance is summarized in Figure 2.2. The presence or not of resistance mutations does not influence the risk of transmission (i.e. virus with resistance mutations present is assumed equally transmissible as virus without such mutations, for a given viral load). Resistance is modelled in terms of the presence or absence of mutations specific to the drugs in use. Distinction is made for each mutation as to whether it is only present in minority virus (if the patient has a mutation present but has stopped drugs that select for that mutation), so the mutation is assumed not transmissible, or if it is present in majority virus, and hence the mutation is assumed transmissible. The probability that resistance mutations present in majority virus of the source partner are transmitted to the newly infected person is dependent on the specific mutation. Once a resistance mutation is transmitted to the new host it is assumed to have a certain probability of being lost from majority virus over time (Castro 2013). Even after being lost from majority virus, it is assumed to remain in minority virus and is selected back as majority virus if an antiretroviral drug selecting for that mutation is initiated. We also consider the possibility of a person who is already infected become super-infected, including with drug resistant HIV (Smith 2005), although there is assumed to be at most a 20% chance that a person super-infected by a person with HIV resistance then has virus with those resistance mutations as a result.

Figure 2.2 Overview of modelling of transmission of drug resistance
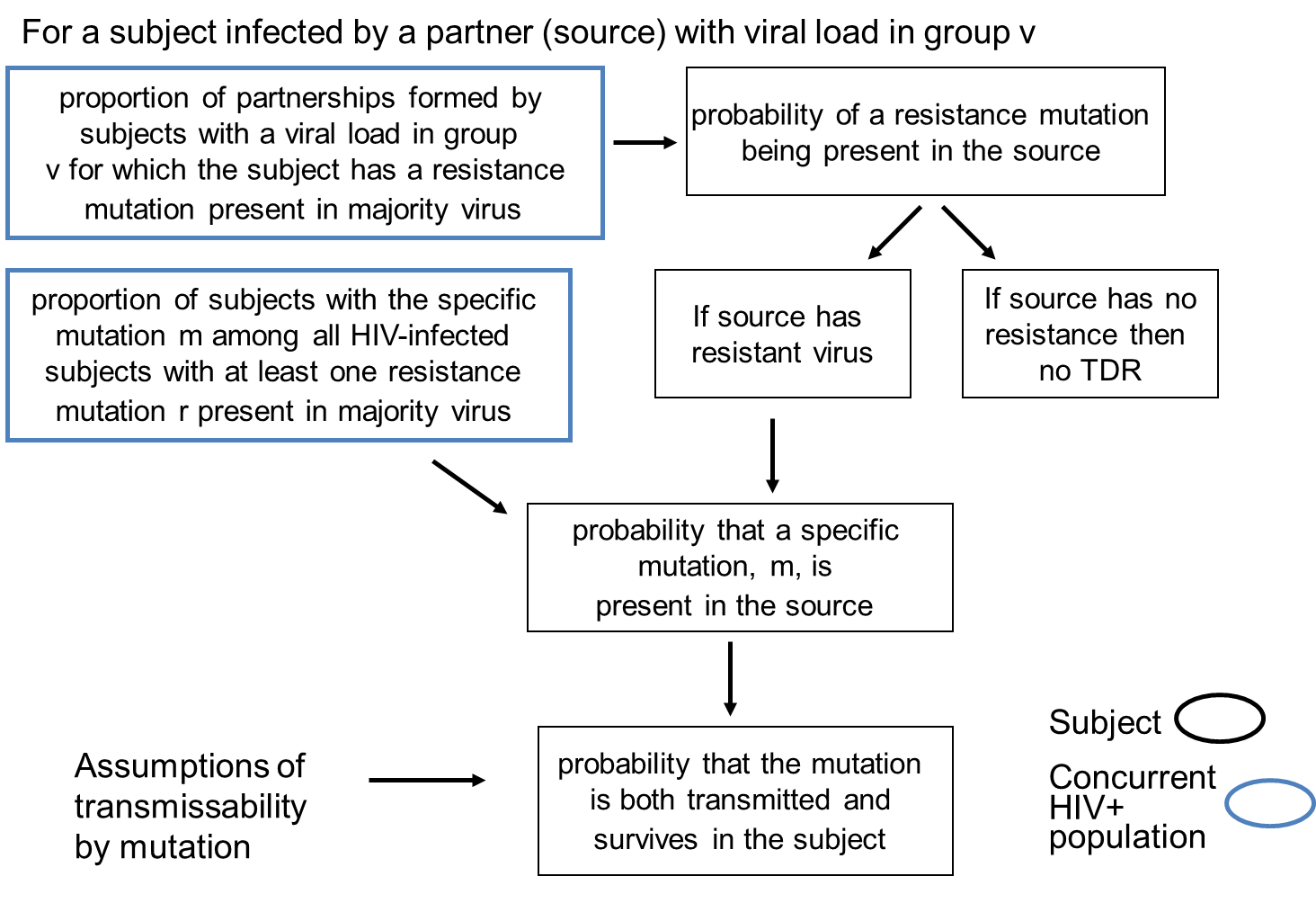


**Transmitted resistance: details**

The viral load group of the person who infected the subject is known, as indicated above. For a subject infected by a person in viral load group v the probability of a resistance mutation being present in the infected person is given by

$$\frac{\sum_{v, \text{ and mutation present}} L_{(t-1)}^{\text{inf}}}{\sum_{v} L_{(t-1)}^{\text{inf}}}$$

where $\sum_{v, \text{and mutation present}}$is the sum over all partnerships had by HIV-infected people in viral load group v for whom a resistance mutation is present in majority virus and $\sum_{v}$is the sum over all HIV-infected subjects in viral load group v. Again, realization of whether the subject is infected by a person with at least one resistance mutation in majority virus is determined by sampling from Uniform(0,1).

For subjects infected from a source partner with a resistance mutation, the probability that a specific mutation, m, is present in the source is given by

$$\frac{\sum_{\text{mutation }m\text{ present }} L_{(t-1)}^{\text{inf}}}{\sum_{\text{mutation present }v} L_{(t-1)}^{\text{inf}}}$$

Where $\sum_{\text{mutation }m \text{present}}$is the sum over all HIV-infected subjects with mutation m present in majority virus and $\sum_{\text{mutation present}}$is the sum over all HIV-infected subjects with at least one resistance mutation in majority virus.

If a given resistance mutation, m, is present in the source partner, the probability that the mutation is both transmitted and survives in the subject (i.e. that its presence will affect future response to drugs for which the mutation confers reduced sensitivity) is shown in Table 2.5.

Table 2.5 Table of probabilities that for a given mutation present in the source partner the mutation is both transmitted and survives in the subject. (based on evidence from studies comparing distribution of resistance mutations between treated and antiretroviral naïve populations; (e.g. Corvasce et al 2006, Turner et al 2004) and modelling of HIV in MSM in the UK (Phillips PLOS ONE 2013).

| Mutation in source partner | Probability that mutation is transmitted and survives in subject |
| --- | --- |
| M184V | 0.20 |
| K65R | 0.20 |
| L74V | 0.50 |
| Q151M | 0.50 |
| Thymidine analogue mutations (TAMS) | 0.50 |
| NNRTI mutations (K103N, G190A, Y181C) | 1 - (0.20*res_trans_factor) |
| PI mutations | 0.50 |

We consider uncertainty in the extent to which transmitted NNRTI resistance mutations are effectively immediately lost (even from minority virus) by sampling from a distribution for parameter *res_trans_factor* (= 0.50/0.75/1.00 for one third of the population), informed by fitting of a model of HIV in MSM to UK data (Phillips et al PLOS ONE 2013).

**Loss from majority virus of transmitted mutations**

There is a probability per 3 months of loss of persistence of transmitted mutations from majority virus to minority virus (same for each mutation) *rate_loss_persistence, uniformly distributed* (0.005, 0.02) (median=0.013), again informed by fitting of a model of HIV in MSM to UK data (Phillips et al PLOS ONE 2013).

**Model outputs relating to sexual behaviour and transmission**

Table 2.6 shows the proportion of people with at least one (at least two, at least 10, at least 50) condomless sex partner in the past year by gender and calendar year. Table 2.7 shows the proportion of new infections that have been acquired from a person in primary HIV infection by year, and the proportion of new infections that have been acquired from a long term partner by year. Table 2.8 shows the proportion of people with at least one (at least two) condomless sex partner in the past year by HIV status and year using modal values for parameters.

**Table 2.6 Sexual risk behaviour in 1990 and 2000 (after behaviour change [phased in from 1995 onwards]): proportion of people with at least one (at least two, at least 10, at least 50) condomless sex partner(s) in the past year by gender**

| 1990 (start of epidemic) | % with > 1 (>2; ­> 10; >50) condomless sex partners (short or long term) in past year | |
| --- | --- | --- |
| Age-group | Males | Females |
| 15- | 59% (12%;5%;0%) | 58% (11%;8%;3%) |
| 25- | 72% (20%;9%;0%) | 67% (7%;5%;2%) |
| 35- | 67% (14%;6%;0%) | 62% (3%;3%;1%) |
| 45- | 62% (9%;3%;0%) | 58% (1%;1%;0%) |
| 55- | 57% (4%;1%;0%) | 54% (0%;0%;0%) |

| 2000 | % with > 1 (>2; ­> 10; >50) condomless sex partners (short or long term) in past year | |
| --- | --- | --- |
| Age-group | Males | Females |
| 15- | 45% (6%;2%;0%) | 45% (7%;6%;2%) |
| 25- | 65% (11%;4%;0%) | 62% (6%;5%;2%) |
| 35- | 55% (7%;3%;0%) | 49% (3%;2%;1%) |
| 45- | 44% (4%;1%;0%) | 37% (0%;0%;0%) |
| 55- | 31% (1%;1%;0%) | 26% (0%;0%;0%) |

**Table 2.8: Proportion of people with at least one (at least two) condomless sex partner (including**

**long term partner) in the past year by HIV status and year using modal values for parameters.**

|  | 1990 | 1995 | 2000 | 2005 | 2010 | 2015 |
| --- | --- | --- | --- | --- | --- | --- |
| HIV+ | 98% (80%) | 82% (35%) | 63% (16%) | 48% (9%) | 43% (9%) | 39% (7%) |
| HIV+ diagnosed | - | - | 59% (16%) | 44% (8%) | 38% (6%) | 35% (5%) |
| HIV - | 86% 64%) | 71% (38%) | 46% (18%) | 46% (19%) | 49% (21%) | 59 (32%) |

# Natural history of HIV infection

Figure 3.1 gives an overview of the modelling of HIV natural history. The model of the natural history of HIV and the effect of antiretroviral therapy has been derived previously and compared with a range of observed data (see Phillips et al Lancet 2008, AIDS 2011, Nakagawa et al 2012, 2015 and associated supplementary material). Below we set out the structure of the model and explain what parameters represent.

Figure 3.1: Overview of modelling of natural history of HIV infection.


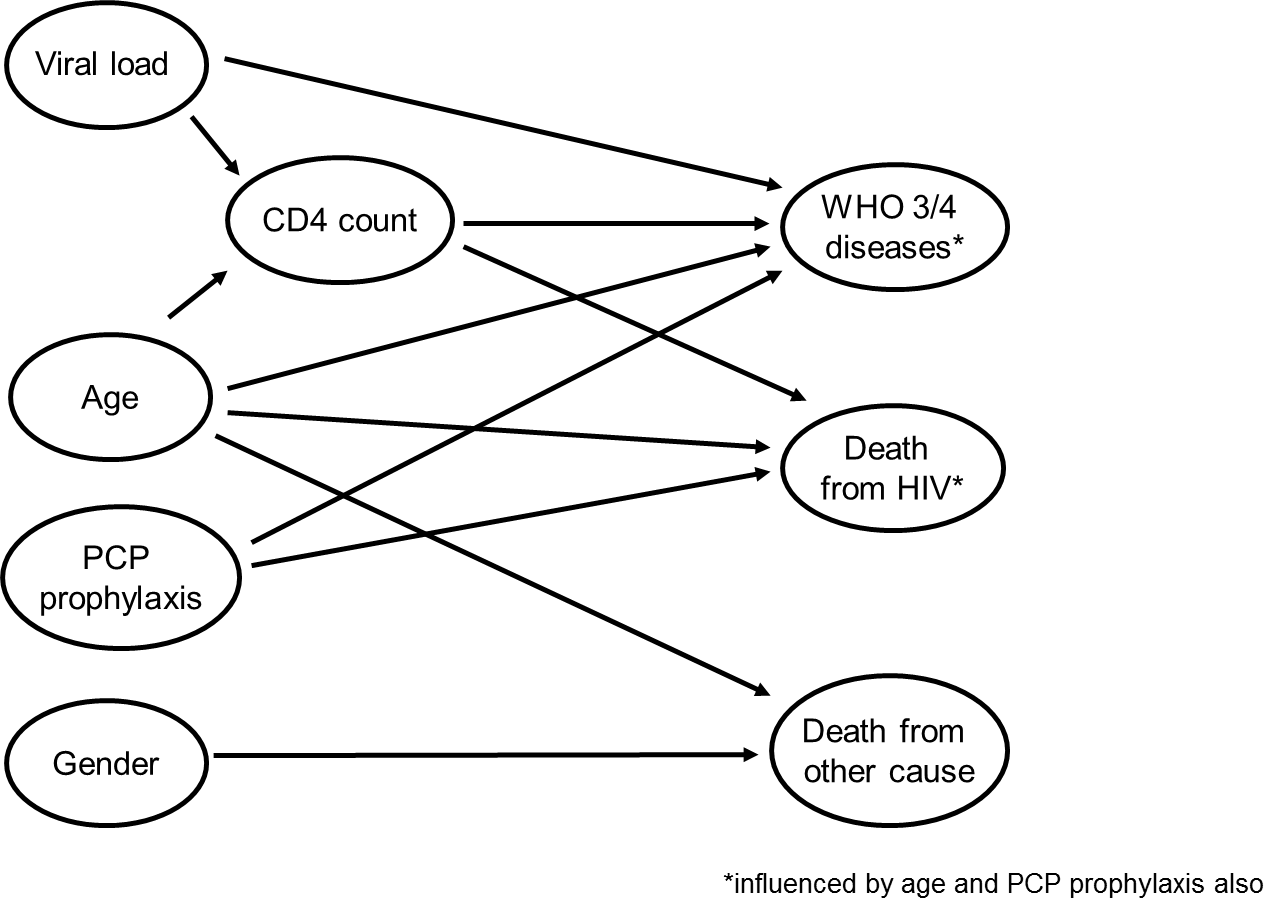


**Determination of changes in viral load and CD4 count**

Initial log_10_ viral load (V_set_) is dependent on age and is sampled from Normal (4.075, 0.5^2^) + ((age{t}-35)*0.005). This distribution is slightly lower for females (V_set_ – 0.2).

This viral load (V_set_) is assumed to be that reached after primary infection. It is not used to determine the risk of transmission in primary infection itself.

Initial CD4 count, modelled on the square root scale, is partially dependent on initial viral load and given by

Square root CD4 count = *mean_sqrtcd4_inf* (= 27.5) - (1.5 x V_set_) + Normal(0,2^2^) – ((age – 35) x 0.05)

Initial virus is assumed to be R5-tropic. Shift to presence of X4 virus is assumed to depend on viral load. Probability of a shift per 3 months is given by 10^v^ x 0.0000004, where v is the current log_10_ viral load.

Viral load change (vc) from period t-1 to period t (i.e. in 3 months) is given by

Vc(t-1( = (gx x 0.02275 + Normal(0, 0.05^2^) + ((age(t-1) - 35) x 0.00075)

gx=1 viral load at t (v(t)) = v(t-1)+ vc(t-1)

CD4 count changes from period t-1 to t are dependent on the current viral load (i.e. viral load at time t-1) and are given by sampling from a Normal distribution with standard deviation *sd_cd4 (=1.2)* and mean *fx* (= 1.0 * exp(normal(0)*0.20) ) times the values as follows

| Viral load at t-1 | Change in square root CD4 count (per 3 mths) |
| --- | --- |
| <3.0 | +0.000 |
| 3-0 | +0.022 |
| 3.5- | +0.085 |
| 4.0- | -0.400 |
| 4.5- | -0.400 |
| 5.0- | -0.850 |
| 5.5- | -1.300 |
| 6.0- | -1.750 |

The change additionally is affected by the current age as follows:

People with X4 virus present experience an additional change in square root CD4 count of -0.25.

These estimates were derived based on consideration of evidence from natural history studies (Pantazis 2005, Sabin JAIDS 2000, Hubert J-B 2000, O'Brien 1998, Henrard 1995, Lyles 2000, Touloumi 2004, Mellors 1997, Koot 1993) and were selected in conjunction with other relevant parameter values to provide a good fit to the incubation period distribution. Differences that have been found in initial viral load by sex, age and risk group are not currently incorporated in the model.

Table 3.1: Incubation period by age. Kaplan-Meier percent with WHO 4 Event. Compare with Darby et al 1996.

| Age at infection | Years from infection | | | | | |
| --- | --- | --- | --- | --- | --- | --- |
| 15- | 0.6% | 4% | 14% | 50% | 75% | 89% |
| 25- | 1.1% | 2% | 23% | 67% | 88% | 97% |
| 35- | 2.1% | 13% | 34% | 82% | 97% | 100% |
| 45- | 3.7% | 21% | 54% | 93% | 100% | 100% |
| 55 | 1.4% | 24% | 59% | 96% | 100% | 100% |

# HIV testing and diagnosis of HIV infection

HIV testing was assumed introduced in symptomatic people in 1997 and in the general population in 2000. At the time of general testing, we assumed a proportion of the population were resistant to be tested for HIV unless symptomatic (*hard_reach=1*). This proportion was defined by two Uniform distributions, p_hard_reach_m= Uniform (0.25, 0.45) and p_hard_reach_w=Uniform (0.15, 0.35) for men and women respectively (median 35% men, 25% women). In the model this group has no possibility of getting tested for HIV unless symptomatic. Limited data are available to inform this parameter (proxy variables are the proportion who reported never being tested for HIV and, more precisely, the proportion who refuse HIV testing), nevertheless we considered it important to take this into account, given the evidence that not everyone accepts HIV testing for various reasons. The level of acceptability of provider initiated HIV testing and counselling (PITC) in resource limited settings is extremely variable from levels of 99%, observed in inpatients in Uganda (Wanyenze 2011) to 31% among outpatients in South Africa (Bassett 2007). Among pregnant women the level of acceptability of PITC seems to be higher, varying from 76 to 99.9% (Hensen 2012), while the estimated acceptability of home-based counselling and testing has been estimated in a meta-analysis to be 83% (Sabapathy 2012). This variability seems to be related mainly to the quality of the intervention delivered and calendar time. Acceptability seems to have increased over time due to the reduction in stigma and higher availability of ART, therefore we thought it was reasonable to assume a decline in the proportion resistant to be tested for HIV down to 5% in 2010.

For the remainder of the population (non-resistant to HIV testing), gender and age-specific rates of HIV testing (for the 1st time and for repeat testing) since 1996 were assumed, and multiplied by a factor 0.00007*base_test_incr (log Normal (ln 0, 0.6) to reflect the level of testing observed in the DHS (DHS Zimbabwe). Pregnant women experience an additional probability of being tested in ANC, which increases over calendar year until 2017, and thereafter remains the same as that in 2017 (DHS Zimbabwe). The probability of attending an ANC is defined as

*prob_anc* = 0 + ((*caldate{t}-date_start_testanc*)^2^)**rate_testanc_inc*;

where *date_start_testanc* is set at 1994 and is the date at which testing in ANCs began, *caldate{t}* refers to the current time period and *rate_testanc_inc* is the rate of testing at an ANC, with probability Beta (3, 900) (median 0.003 per 3 month period). Prob_anc is multiplied by a factor to assume lower attendance of ANC for older women up to 2012. After 2012 prob_anc is not dependent on age. All women who attend an ANC are tested for HIV if not previously diagnosed. A second test is also performed for all women after birth to capture the assumption that women are tested twice during pregnancy.

For people who have never had condomless sex, a 3-fold reduction in the rate of testing was assumed. This has been observed in the Zimbabwe DHS (Zimbabwe DHS 2010/2011) where people who reported never having had sex (whether with or without condom) are less likely to test for HIV.

People with acute symptoms (WHO stage 4, 3 or active TB) are assumed to have a higher chance of testing for HIV in that 3 month period and a higher chance of being linked to care once diagnosed. The probability of testing as a result of symptoms in 1997 was 0.1, 0.1 and 0.05, respectively, for people with a WHO stage 4 event, tuberculosis and WHO stage 3 event. This was multiplied in each 3 month period by a factor uniformly distributed (1.05, 1.075) to give the probability of testing as a result of symptoms in each 3 month period. These values were truncated at 0.8.

In this application of the model it is assumed HIV self-testing is not available.

Testing for symptomatic people who do not have HIV, but with symptoms suggesting HIV may also take place with 0.5% probability. Estimates of the number of people being tested due to symptoms are shown in the *Description of Model Calibration* document

Finally, if the chances of circumcision are realised amongst men aged over 15, a HIV test is performed. Circumcision only occurs if the results of this test are negative (see section “Circumcision”).

Modelled and observed gender and age-specific proportions of ever being tested for HIV and tested for HIV in the last year are given in the *Description of Model Calibration* document.

# Modelling the effect of ART

The structure of the relationship between ART adherence, viral load, development of resistance, CD4 count and risk of death is modelled is illustrated in Figure 5.1 below. The adherence level - the determination of which is described in detail below - influences the risk of acquisition of new mutations as well as having a direct effect on the viral load and CD4 count. Acquisition of resistance mutations impacts on the number of fully active drugs in the current regimen. This, in turn, is a further determinant of the risk of new mutations arising. Failure of the current line of ART is determined by CD4 count or viral load or clinical disease, depending on the monitoring strategy, and this triggers a switch to the next line of ART (if assumed available, and often with a delay), which leads to the number of active drugs again returning to 3 or more if on boosted PIs. The following sections provide further details, including how adherence levels are determined and how they influence the viral load, risk of resistance and the CD4 count. We also explain the modelling of ART interruption and loss to follow-up. We provide references to papers that have been used to inform the approach. It should be noted though that parameter values used in the model are rarely extracted directly from any one paper, they are values that are arrived at based on their ability to generally reproduce outputs that are consistent with observed estimates, as illustrated below.

Figure 5.1 Overview of the modelling of the effect of ART, highlighting the role of adherence.

**
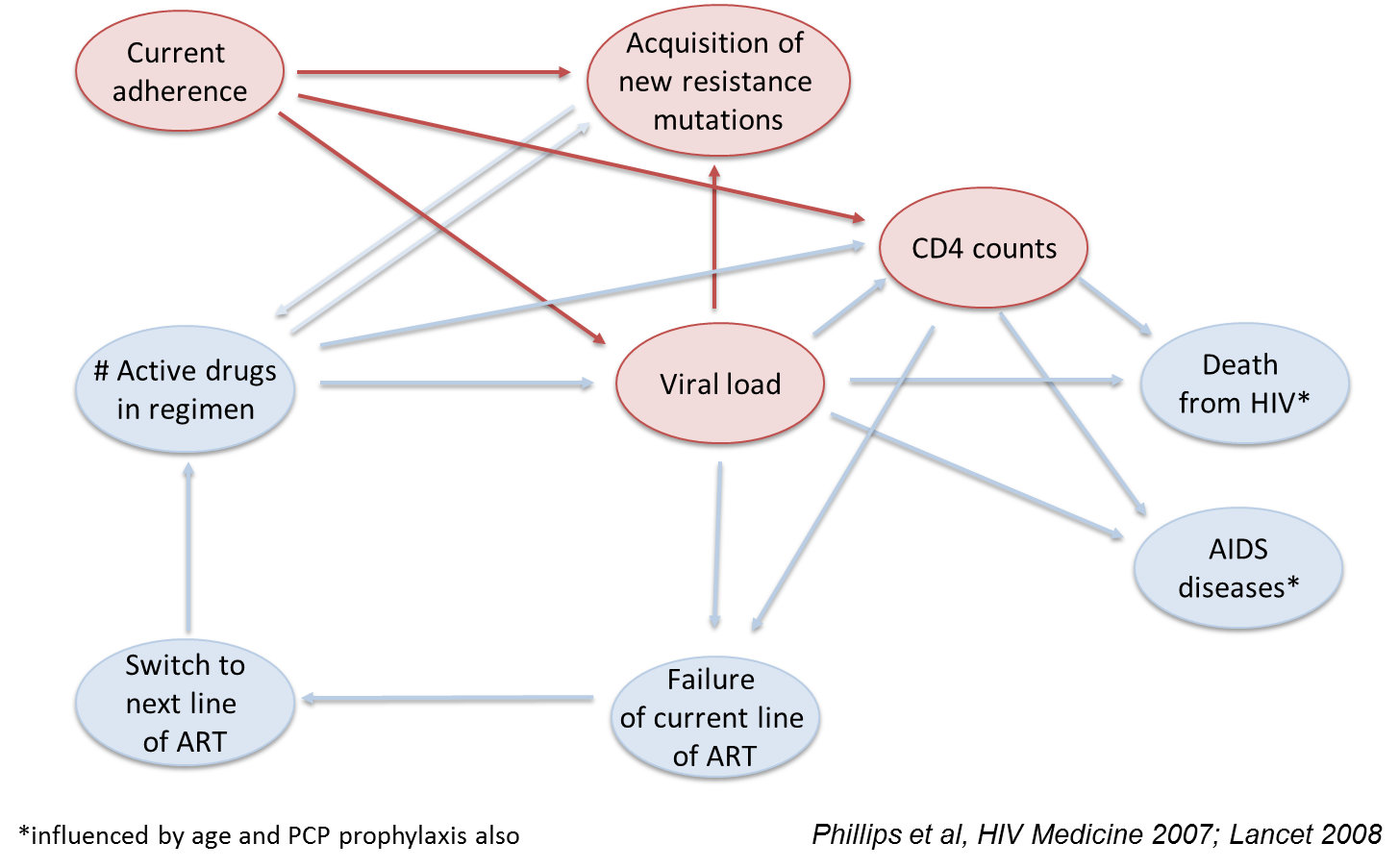
**

**Initiation of ART**

It is assumed ART became available in 2004. Eligibility for ART initiation in people diagnosed with HIV before 2003 is determined by the development of a WHO 4 or TB event. From 2004 to 2010, eligibility for ART initiation is determined by a measured CD4 count < 200 (in the last year) or the development of a WHO 4 event (at time t-1); from 2011 onwards, is determined by a CD4 count <350 (measured in the last year) or a WHO 4 event (at time t-1)). From 2015 onwards, eligibility for ART initiation is determined by a CD4 count < 500 (measured in the last year), the development of a WHO 4 event at time t-1, of TB disease in the last 6 months, or pregnancy (option B+). From 2017 onwards, all people diagnosed with HIV are eligible for treatment. For people that are eligible to be initiated on treatment the probability that ART initiation occurs is determined by sampling from a Uniform (0,1) distribution and determined whether this is below 0.6.

From 2003 onwards, we assume that CD4 counts are monitored at 6 monthly intervals for those who are in pre-ART care. Viral load monitoring is assumed at 5% per 3 month period after 2016.5. Once a person is under the viral load monitoring policy, it is assumed they will stay under this policy.

**Switch to second line after failure of first line ART**

Whichever the criterion for the need to switch to second line ART is determined, the probability of switching per 3 month period after the criterion is met is *pr_switch*_line (randomly distributed Beta (2, 100), median =0.02)). The switch rate is likely to vary substantially by setting (Fox 2012; Johnston 2012). In several settings, including Zimbabwe, the proportion of people who have started second line ART is consistent with a value for *pr_switch*_line of below 0.1 (e.g. Lesotho, Malawi) (personal communications Zimbabwe MoHCC; Government of Malawi Ministry of Health, Integrated HIV Program Report, Oct-Dec 2014).

**Adherence pattern**

The model specifies a current adherence level (i.e. for the current 3 month period) for people on ART. Given that the model updates in 3 month time periods the adherence level in a given 3 month period has to effectively be considered as the average adherence over the period. The determination of this is described below. Interruption of ART for periods of duration 3 months is considered separately (and explained in subsequent sections below).

Consistent with evidence that people tend to have different tendencies to adhere (Cambiano 2010a;Carrieri 2001;El-Khatib 2011;Genberg 2012;Glass 2010;Kleeberger 2004;Lazo 2007;Levine 2005;Mannheimer 2002;Meresse 2014;Osterberg 2005), adherence is modelled using two components. Each patient has a certain greater or lesser tendency to adhere (*adhav,* measured on a scale of 0-100%) but their actual adherence in a given period varies over time, both at random and according to the presence of symptoms (with drug toxicity or presence of WHO stage 4 disease leading to a decrease in adherence) and there is an effect of a tendency for increasing adherence with age. Adherence in a given 3 month period (*adh{t}*) is measured on a scale of 0 to 100%. *adhvar* is the standard deviation representing the within-person period-to-period variability over time. Thus, adherence at any one period (*adh{t}*) is determined as follows (although with modifications explained below):- adh(t) = *adhav* + Normal(0,*adhva*r^2^). The distribution of the values of *adhav* and *adhvar* is specified as follows and as illustrated in Figure 5.2.

5% probability *adhav* = 50% *adhvar* = 0.2

10% probability *adhav* = 80% *adhvar* = 0.2

65% probability *adhav* = 90% *adhvar*  = 0.05

20% probability *adhav* = 95% *adhvar* = 0.05

Figure 5.2
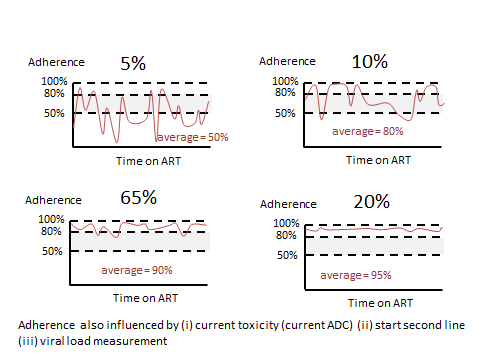
 Illustration of adherence pattern assumptions. 5% of the population have the adherence as shown in the top left, 10% as shown in the top right, etc. While adherence is generally high in the majority of people on ART (hence the high proportion of people on ART with viral suppression), most probably experience at least some periods of poorer adherence (e.g. see (Muyingo 2008)).

This distribution of adherence is primarily determined by the adherence levels required for the model outputs to mimic observed data. This includes data on rates of resistance development and virologic failure and also data on the proportion of patients at first virologic failure who have no resistance mutations present (Bangsberg 2004;Bangsberg 2006b;Hamers 2011;Hassan 2014;Hoffmann 2014;Kobin 2011;Li 2014;Mackie 2010;Mannheimer 2002;Meresse 2014;Rosenblum 2009;Tran 2014;Usitalo 2014;von, V 2013). It is clear from such data in more recent years that the great majority of patients who started ART with 3 or more drugs are sufficiently adherent that virologic failure rates are low (and so resistance accumulation is also likely to be low) (El-Khatib 2011;Johannessen 2009).

Comparisons between model outputs and data from the literature in Figure 5.3 -Figure 5.9 help to illustrate the extent to which the model captures various aspects of virologic responses to ART.

Figure 5.3 Risk of virologic failure while on ART according to adherence level


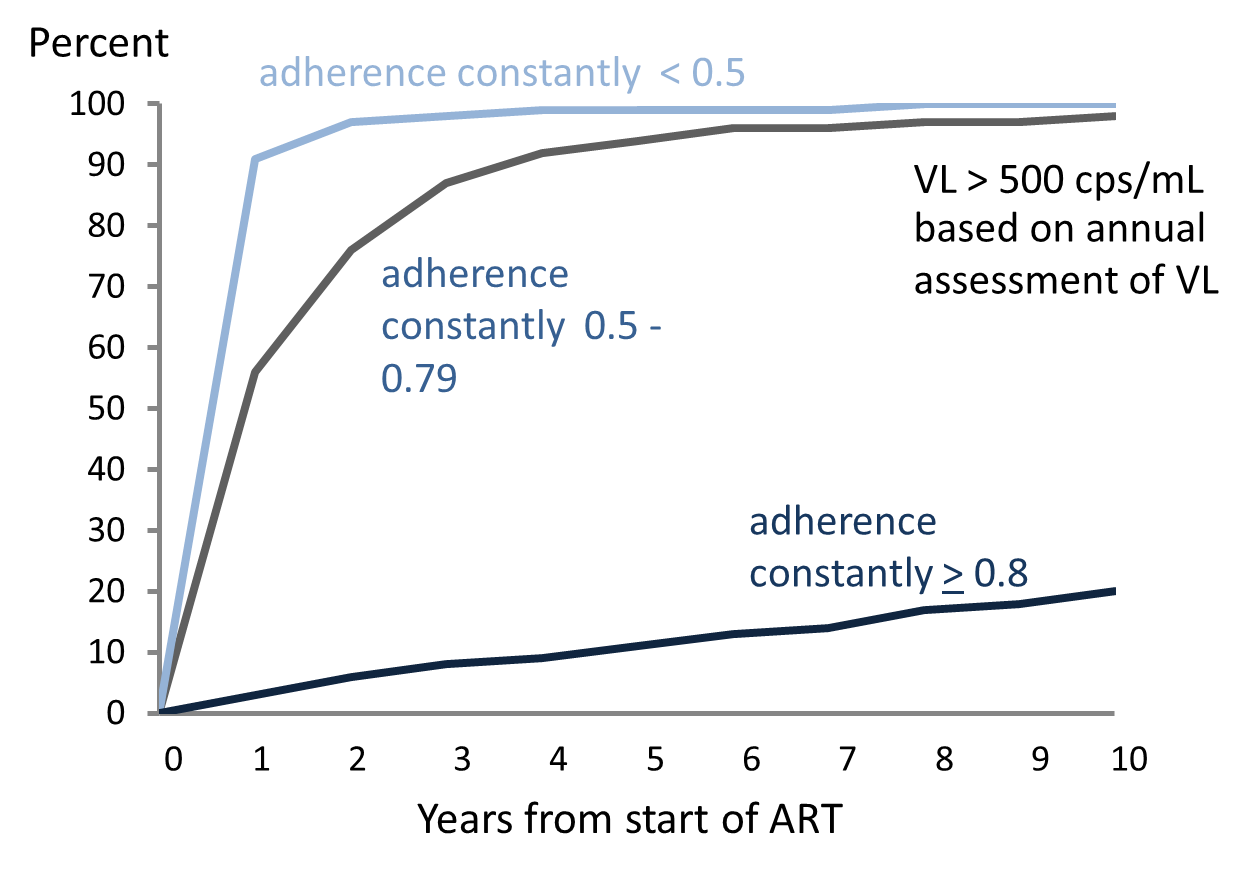


Figure 5.4 Risk of NNRTI resistance with virologic failure while on ART, according to adherence level


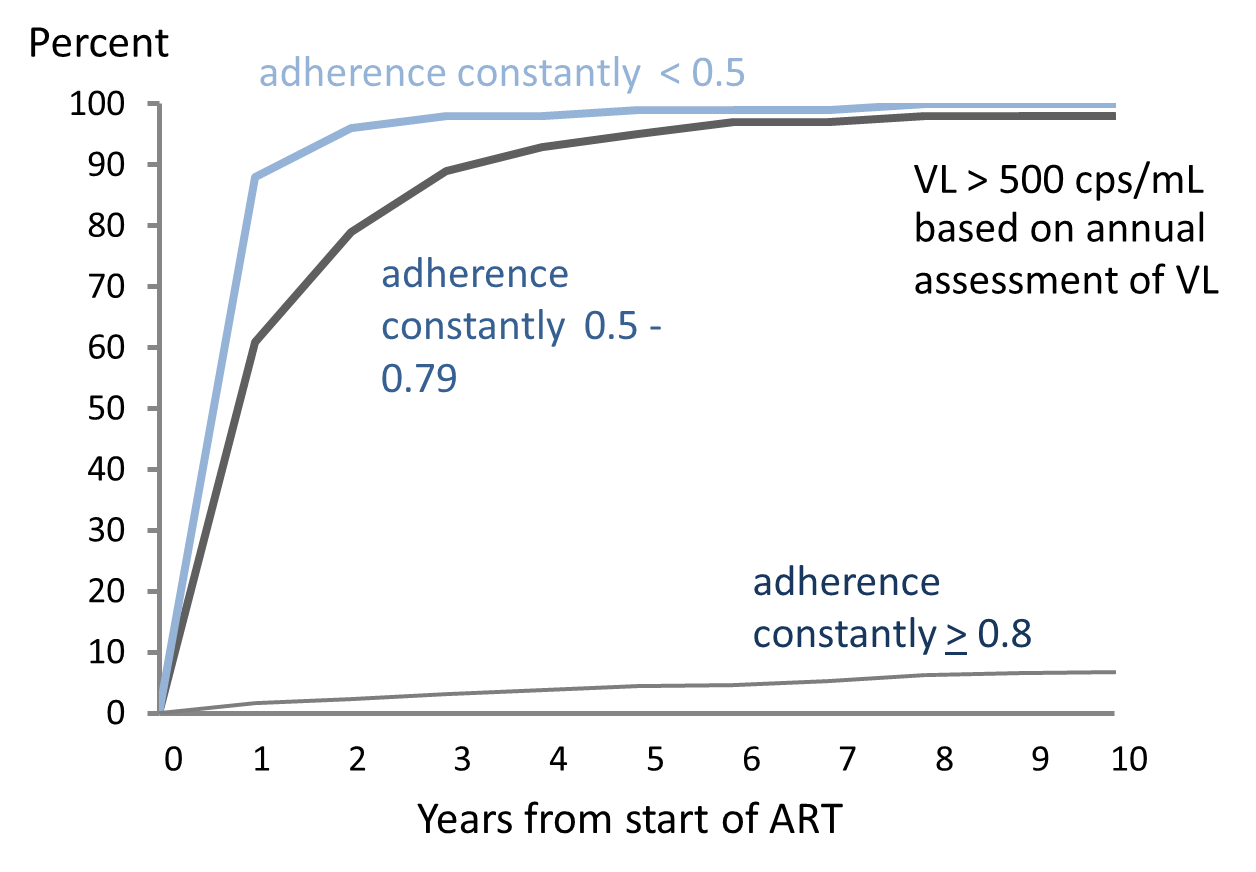


The distribution of adherence over the first year of ART has been compared with data from a large programme in Zambia (see Figure 5.5; (Chi 2009)). The degree to which outputs on viral load at one year from start of ART is shown in Figure 5.6. These are reconstructed outcomes for all people who have initiated ART in Zimbabwe (the overall mean CD4 count at initiation is 145 /mm^3^). Figure 5.7 and Figure 5.8 compare Kaplan Meier estimates of time to virologic failure and resistance, respectively, between the model and observed data, in the latter case from the UK due to the lack of data from sub-Saharan Africa. Figure 5.9 illustrates the proportion of people with resistance (amongst those on ART with non-suppressed viral load) and corresponds to estimates from the large WHO resistance surveillance.

Figure 5.5 Distribution of average adherence level over first year of ART (for those on ART at 1 year).


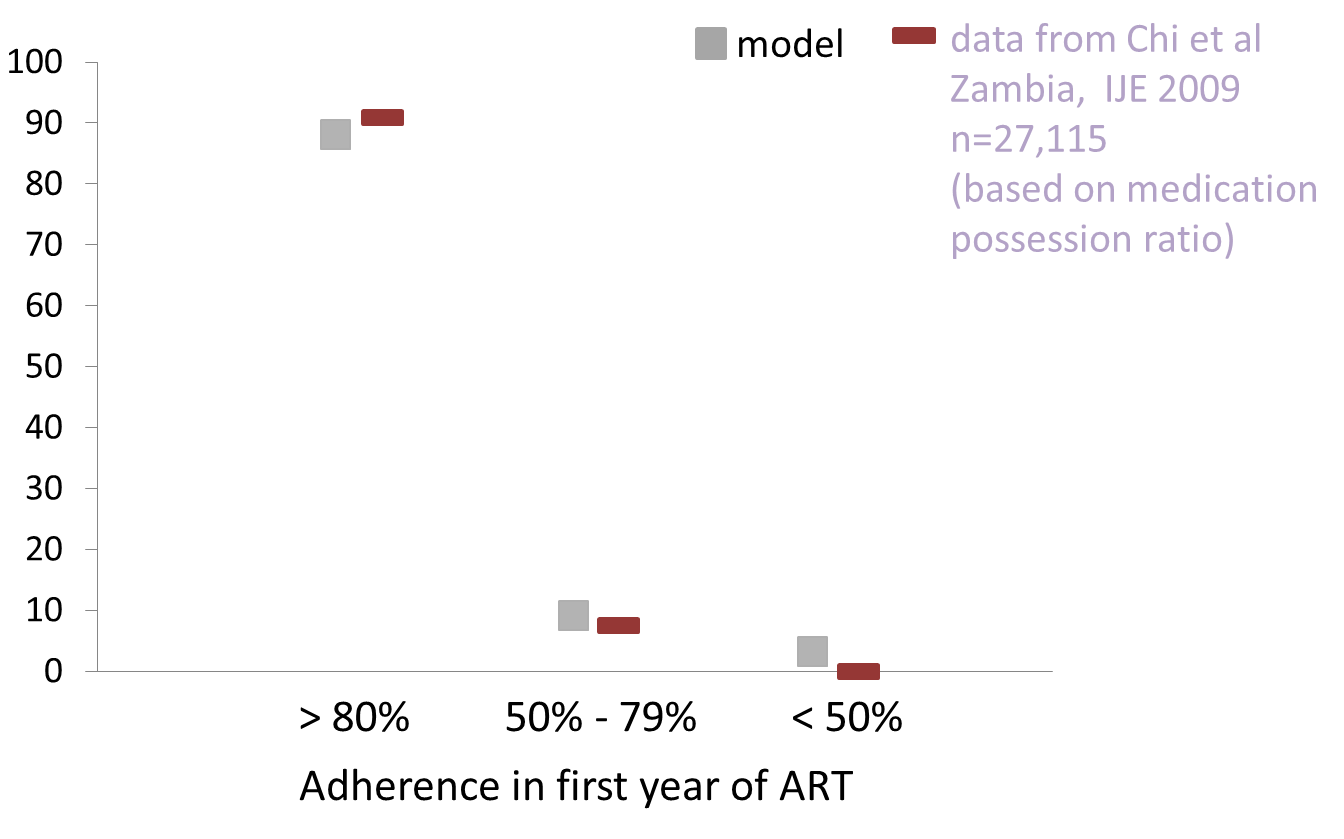


Figure 5.6 (a) Percent of people alive at given time points from start of ART who have viral load suppression and (b) percent of people alive and on ART at given time points .from start of ART who have viral load suppression (WHO Resistance Surveillance Report 2012(WHO 2012)).

(a)

**
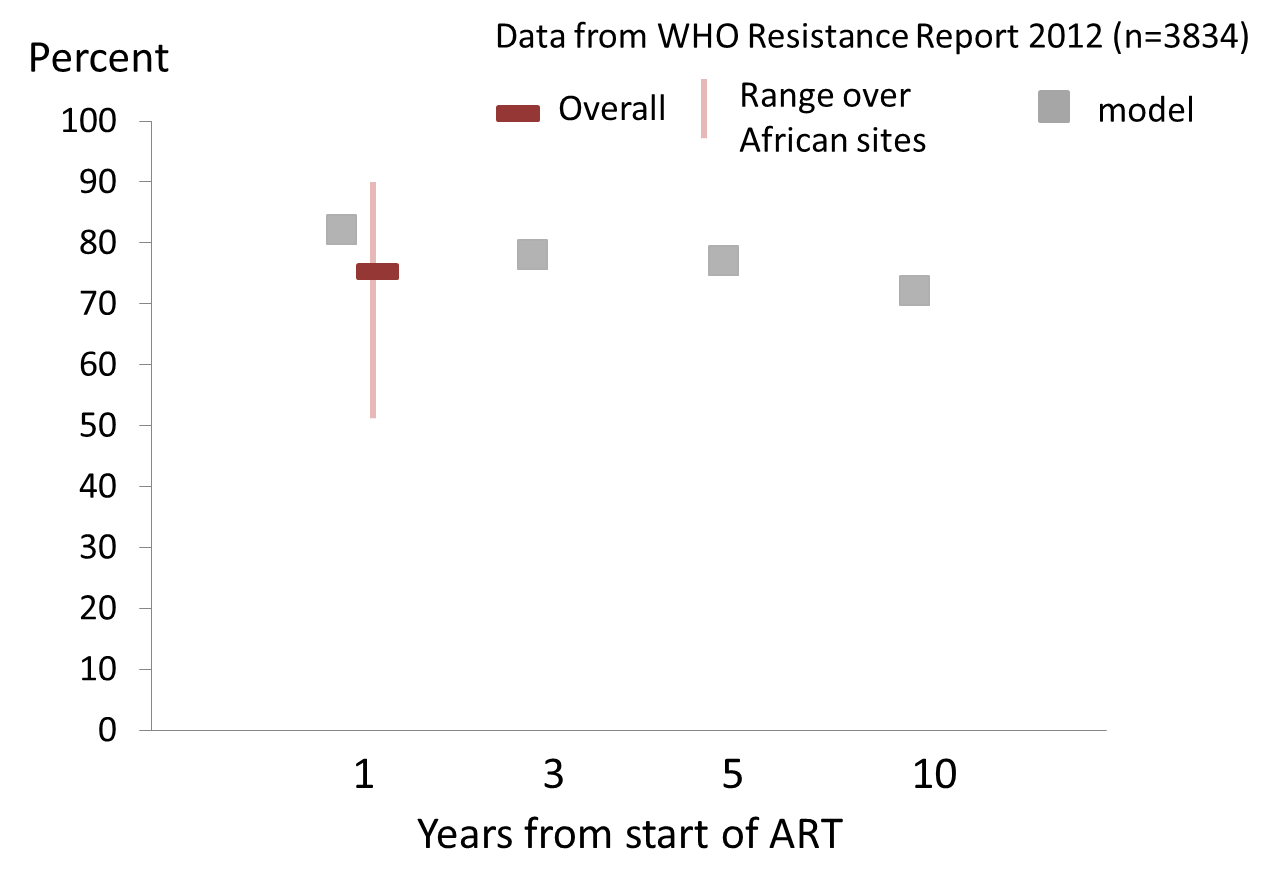
**

(b)

**
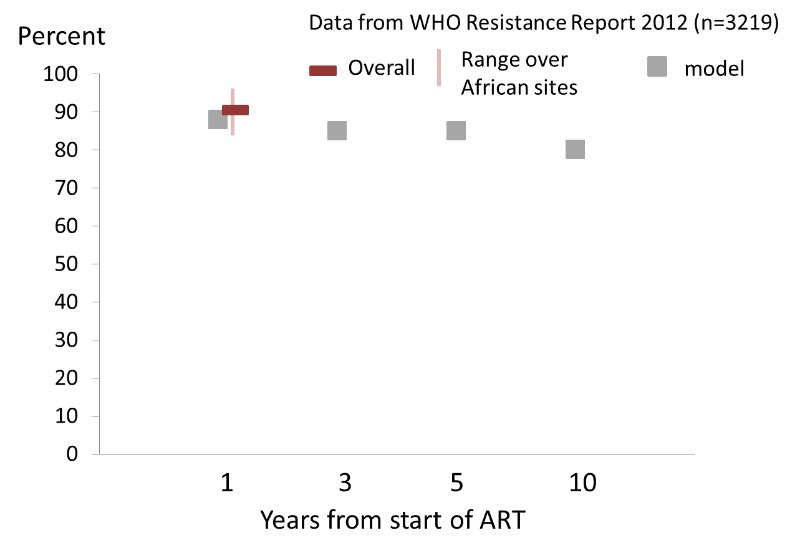
**

**
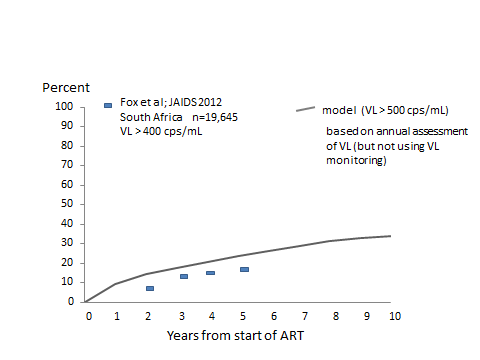
Figure 5.7 Kaplan Meier estimates of risk of virologic failure while on ART, by time from start of ART.**

**
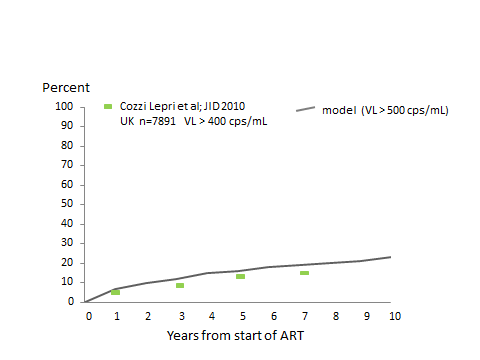
**

Figure 5.8 Kaplan Meier estimates of risk of NNRTI resistance with virologic failure while on ART, by time from start of ART (Cozzi-Lepri 2010).

Figure 5.9 Of people with viral load > 500 at 1 year from start of ART, percent who have NNRTI drug resistance (WHO Resistance Surveillance Report 2012(WHO 2012)).

**
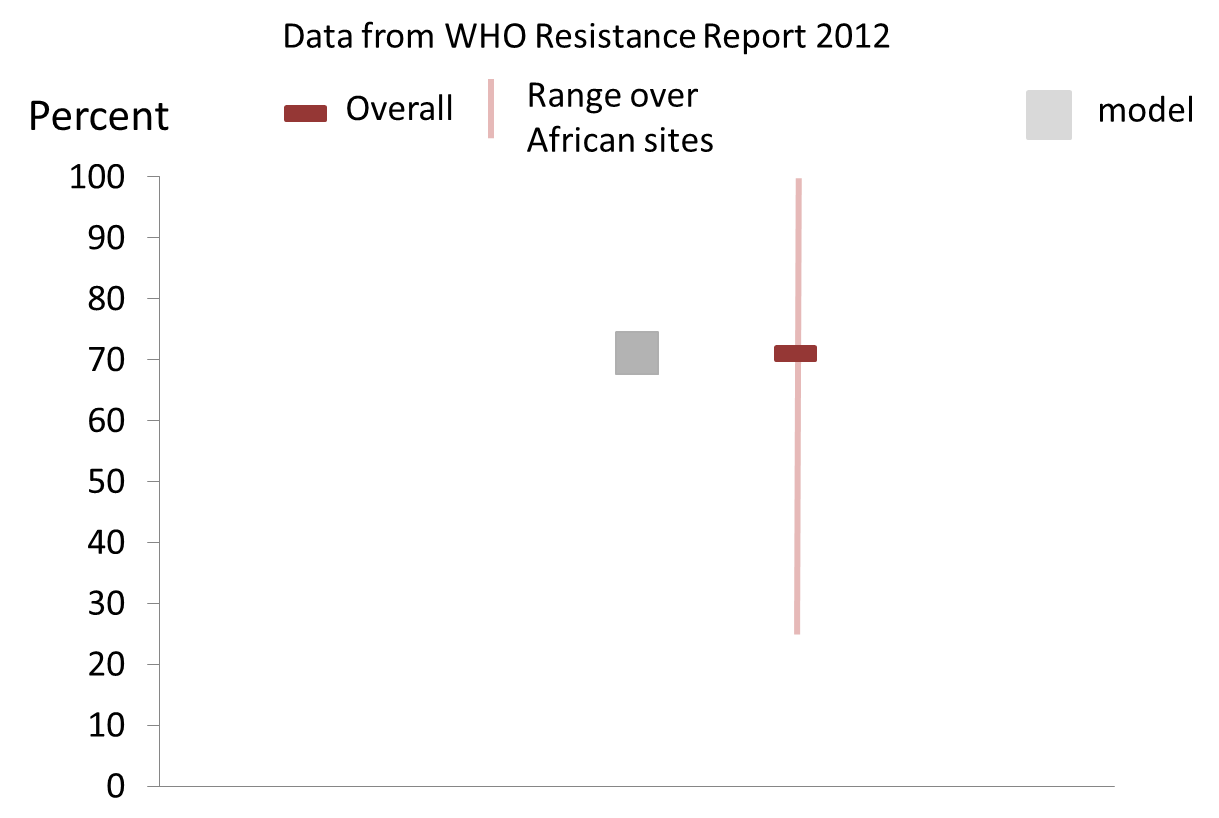
**

**Effective adherence**

We also considered the concept of *effective* adherence, which reflects predicted adequacy of drug levels, whereby for those on regimens that do not include an NNRTI the effective adherence is as the adherence itself, but for those on NNRTI-containing regimens the effective adherence is the adherence + *add_eff_adh_nnrti* (base value Log normal(ln 0.10, 0.30)), reflecting the long half life of NNRTI drugs (Cheeseman 1993) which is an advantage as it means such regimens are more forgiving of periods of poor adherence (Bangsberg 2004;Bangsberg 2006a;Bangsberg 2006b;Gardner 2009;Gross 2008;Kobin 2011;Meresse 2014;Parienti 2007). Additionally, it is assumed that patients on ART are susceptible to occasional (rate 0.02 per 3-months severe temporary drops in drug level (i.e. effective adherence level), leaving them susceptible to viral rebound (but with low risk of resistance as the effective adherence drop is so profound). This phenomenon is assumed to be 100 times more frequent among those on protease inhibitor regimens than in those on other regimens. This latter assumption is the only plausible means (at least within our model framework) to explain why virologic failure occurring on boosted protease inhibitor regimens often occurs in the absence of resistance (Hill 2013).

**Effect of viral load measurement above 1000 cps/mL on adherence**

Various factors can influence adherence, including the initial measurement of viral load > 1000 copies/mL which is assumed to lead to an increase in adherence in 70% of people as a result of targeted adherence intervention; this is consistent with data showing that a high proportion of people with measured viral load > 1000 copies/mL who undergo an adherence intervention subsequently achieve viral suppression without a change in ART (Orrell et al 2007, Hoffman et al 2009, Hoffman et al 2013, Rutstein et al 2015) and broadly consistent with a meta-analysis (Bonner et al 2013). Although the appropriate duration to assume for this effect is uncertain (Hoffman et al 2013), the impact of adherence interventions has often been shown to diminish with time (Bärnighausen et al 2011). Based on this overall body of data, we assume that the adherence intervention is effective only the first time it is performed and that for 40% the effect is permanent (i.e. 70% x 40%= 28% of those with a viral load >1000), but that in the remaining 60% (i.e. 70% x 60% = 42% of those with viral load>1000) it lasts only 6 months.

**Interruption of ART**

People can interrupt ART, and this may be due to not continuing with clinic visits (disengagement, modelled as simultaneous interruption and loss) but ART can be interrupted also in those still attending clinical visits. The basic rate of interruption due to patient choice is *rate_int_choice* (base value: Log normal (ln 0.003, 0.5)) - this rate is greater in people with current toxicity (2-fold) (note that in addition to this increased risk of interruption with current toxicity, there is assumed to be some substitution of drugs causing toxicity with available alternatives and a greater rate of interruption in patients with a greater tendency to be non-adherent (1.5-fold if adherence average *adhav* 50 – 79% and 2-fold if adherence average *adhav* < 50%). In a systematic review, drug toxicity, adverse events and side effects have been found to be the most commonly given reasons for drug discontinuation (Kranzer 2011).

The rate of interruption also reduces with time on ART, decreasing after 2 years. Evidence suggests that rates of discontinuation do decrease over time ((Kranzer 2010;Tassie 2010;Wandeler 2012) although the point at which the risk lowers might be somewhat earlier than 2 years. If adherence average (*adhav*) > 80% then the chance that interruption coincides with interrupting/stopping visits to the clinic is equal to *prob_lost_art* (randomly distributed Beta 4, 4); if 50 <= *adhav* < 80% then *prob_lost_art is multiplied by* 1.5, if *adhav* < 50% then *prob_lost_art* is multiplied by 2. This is due to an assumption that factors leading to poor adherence are also likely to be associated with interruption. The rate of interruption and disengagement with care is likely to vary by setting. Figure 5.10 shows a comparison between modelled and observed (from a study by Kranzer et al. (Kranzer 2010)) Kaplan Meier estimates of the percent of people having interrupted or discontinued ART by time from ART initiation.

Figure 5.10 Percent who have interrupted or discontinued ART by time from initiation.


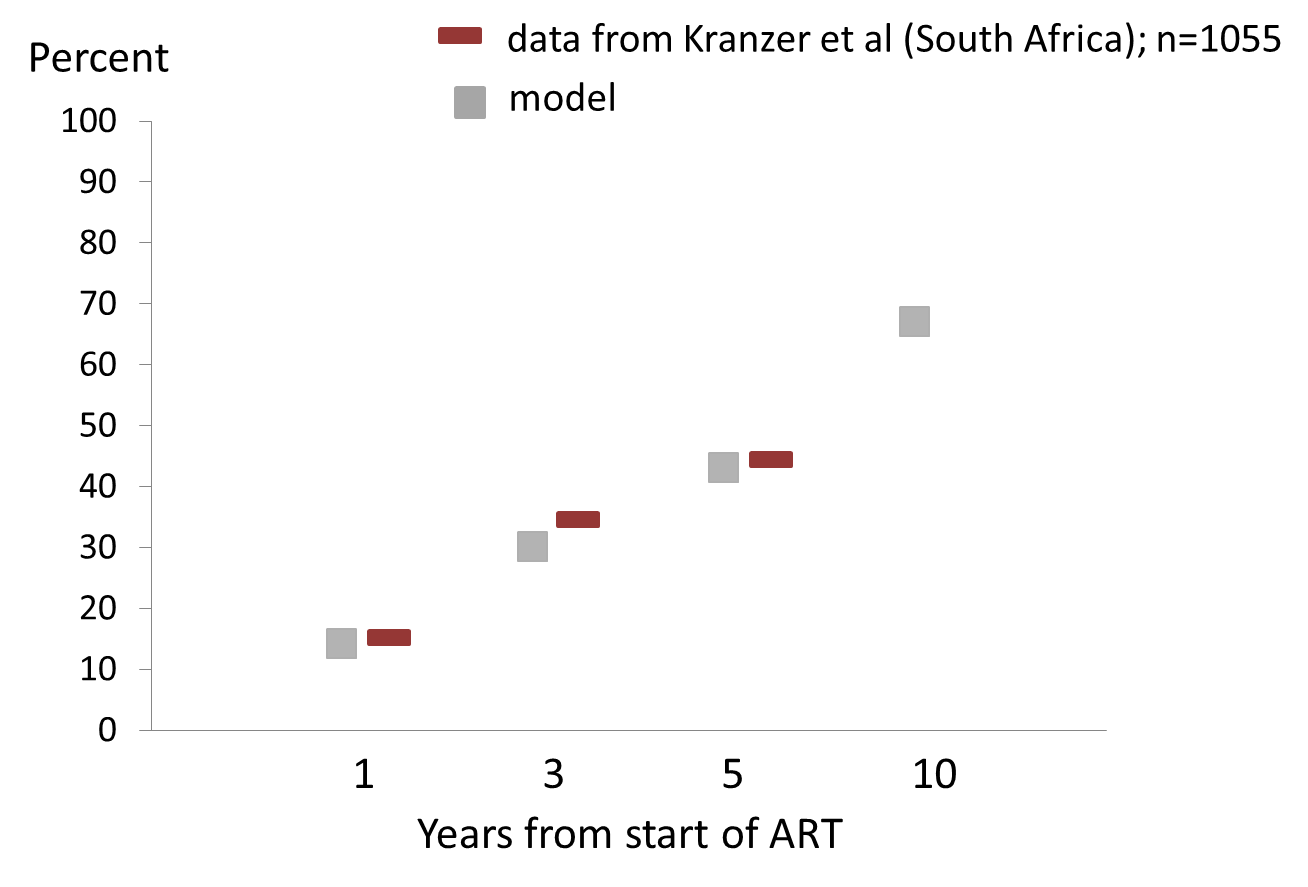


**Interruption of ART without clinic/clinician being aware**

It is known that in some instances people on ART have such poor adherence that they have in fact interrupted or stopped ART entirely but, in the same way that the clinician is not always aware of the true adherence level, they are also not always aware when the person has completely interrupted ART. This means that the clinician (in the absence of a resistance test) may think a patient is virologically failing, because viral load is high, when in fact this is due to interruption rather than resistance. This can be seen from studies on people with virologic failure in which a proportion have no identified resistance mutations (Hamers 2011;Hoffmann 2009;Wallis 2010). Thus, when a person interrupts ART (but remains under care) we introduce a variable that indicates whether the clinician is unaware. *clinic_not_aw_int_frac* (base value Beta (6,4), median=0.61). This distribution was chosen to produce realistic model outputs for the proportion of people with virological failure who have resistance. If a patient has interrupted ART with the clinician unaware then not only is the patient (wrongly) classified (by the clinician) as virologically failing (if viral load has been measured), but a switch to second line can occur. Figure O compares the proportion of people with resistance between our model and WHO survey data.

**Re-initiation of ART after interrupting in patients still under clinic follow-up**

For patients who have interrupted ART due to choice but are still under clinic follow-up, the probability of restarting ART per 3 months in the base model is *rate_restart* (Log normal (ln 0.5, 0.5). This probability is increased 3-fold if a new WHO 3 condition has occurred at t-1, and 5-fold if a new WHO 4 condition has occurred at t-1 since occurrence of clinical disease in a person seen at clinic is likely to prompt ART re-initiation. This will vary by setting but is informed by studies showing that of people who have initiated ART who are still seen at clinic a very high proportion are on ART at 12 months from start of ART (McMahon 2013). Kranzer et al found a rate of restarting ART amongst those that interrupted or discontinued of 21 per 100 person-years but this figure is an overall figure which includes in the denominator those who are not attending the clinic (loss to follow-up and return to care are described below). The equivalent figure, produced as an output from the model is 19 per 100 person-years.

**Interruption due to drug stock-outs**

The basic rate of interruption due to interruption of the drug supply is *prob_supply_interrupted* per 3 months (base value: 0.01). The rate of resupply (*prob_supply_resumed*) has a base value 0.8 per 3 months. This will vary by setting. There have been reported to be significant issues with drug stock-outs in Zimbabwe in the past (<http://www.irinnews.org/report/97224/still-struggling-with-drug-shortages>). For patients who have interrupted ART due to interruption of supply the probability of restarting ART per 3 months is *prob_supply_resumed* (base value 0.8).

**Loss to follow-up while off ART (for reasons apart from drug stock-outs)**

The probability per 3 months of interrupting/stopping clinic visits (i.e. being lost to follow-up) is *rate_lost* (base value Log normal (ln 0.02, 0.4)) if adherence average *adhav* > 80%. This is increased by 1.5 fold if 50% < *adhav* < 80% and by 2-fold if *adhav* < 50%. This high rate is informed by the fact that low numbers of people attending clinics after having been initiated on ART are not still on ART (e.g. WHO 2012 Resistance report). Interruption of ART and loss to follow-up are assumed correlated with the underlying tendency to adhere when on ART because we assume that the same underlying social, practical and economic factors will be an underlying cause of these behaviours.

For people lost to follow-up who are asymptomatic, the probability of returning to clinic per 3 months is *rate_return* (base value Log normal (ln 0.20, 0.50)) if adherence average *adhav* > 80%. This is decreased by 2-fold if 50% < *adhav* < 80% and by 3-fold if *adhav* < 50%. If a person develops a new WHO 3 or 4 event then they are assumed to return to the clinic with probability 1. As mentioned above, this leads to an overall rate of restarting of ART after interruption (including having been loss to follow-up in many cases) consistent with the estimates from South Africa from Kranzer et al, although these will vary by setting (Charurat 2010;Fox 2012;Kranzer 2010).

As output from the model, the retention on ART at 1 year is 94% amongst those still alive. This is difficult to compare with estimates from the literature because few studies are able to know the outcome status of all people initiated on ART, and a high proportion of those lost from a given clinic in fact remain on ART at another clinic or have died. However, taking this into account, this modelled output value of 94% seems consistent with data from the WHO Drug Resistance Surveillance Report (2012) (see Figure 5.11).


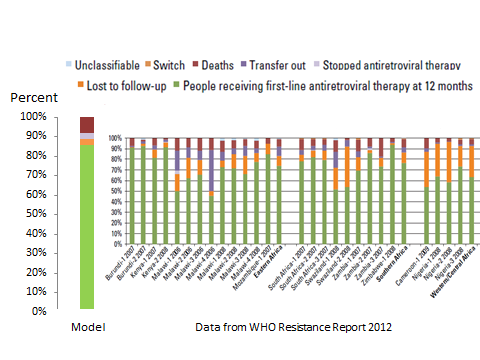


Figure 5.11 Status at 1 year from start of ART. Data is from WHO Drug Resistance Surveillance Report (2012).

**Effect of ART on viral load, CD4 count, resistance development and drug toxicity**

This section describes the determination of updated viral load, CD4 count, and acquisition of new resistance mutations in a given time period for people on ART. The updated viral load, CD4 count and risk of new resistance mutations appearing all depend on the effective adherence in the previous and current period, the number of active drugs (*nactive(t-1)*) and the current viral load, as well as the time period from the last time ART was started or restarted. The values of viral load, CD4 count, and resistance mutation risk for any combination of these factors are given in the tables below. The rationale behind this approach and how the specific values in the table were chosen is explained below. The choice of values is directly informed by studies in this area and by comparison of model outputs with data. For the new resistance mutation risk, the number in the table is multiplied by the viral load (mean of values at t-1 and t) to give a value for the variable *newmut*, which is used when assessing whether a new mutation or mutations have arisen (see below).

**Number of active drugs**

We use the concept of the number of drugs that are active, based on presence of resistance mutations to the drugs being used. The level of resistance is determined by the presence of drug resistance mutations, with a given set of mutations being translated into a level of resistance to a given drug on a scale of 0 to 1 in the same way as is done for common resistance interpretation systems. The activity level of a drug is then calculated as 1 minus the level of resistance to the drug. The ability of the number of active drugs, or the genotypic sensitivity score, to predict the viral load outcome is well established (DeGruttola 2000), and the concept of using a genotypic score to define “optimised background therapy” has been common to the design of several trials in treatment experienced patients (e.g. (Grinsztejn 2007)).

**Classification of adherence levels**

While we model the adherence level for each individual at each three month time period as a value between 0 and 100%, to determine the viral load, CD4 count and resistance risk, we classify adherence into three levels.

This is the simplest approach that allows inclusion of the fact that the relationship between adherence and resistance risk is not linear, since the risk of resistance tends to be lower when the adherence is either low or high, and the risk of resistance is highest when adherence is moderate, allowing enough replication for mutations to be selected for and enough drug present to allow selection of virus with resistance mutations (Bangsberg 2004;Gardner 2009;Rosenbloom 2012).

The cut-offs used to define the three adherence levels are 50% and 80%. Adherence-resistance and adherence-viral load relationships differ by regimen type and even specific regimen within a class and any overall breakdown into groups is necessarily a simplification. A cut off of 80% is chosen as the upper level as (unlike for unboosted PI regimens) at adherence levels of at least 80%, NNRTI and boosted PI regimens are likely to have maximal or close to maximal effects on viral load and minimal risk of resistance selection (Parienti 2007). Actual risk of resistance probably depends on the pattern of adherence, not just the average over a three month period, so that a treatment interruption of over 1 week during the three month period, while maintaining an overall average adherence of 80%, could lead to a higher level of risk of resistance emergence than a situation in which the adherence was more uniform over the period (Genberg 2012), although in people who have ongoing viral suppression NNRTI regimens seem to be generally robust to even relatively low levels of adherence (Cambiano 2010b;Gross 2008;Meresse 2014;Parienti 2007). A level below 50% is one that that has been associated with raised risk of detectable viral load (Arnsten 2001;Genberg 2012)

**Determination of viral load, CD4 count and risk of resistance in people on ART**

*Viral load, CD4 count and risk of resistance in the first 3 months after (re-)starting ART*

Table 5.1 shows how the viral load, CD4 count and risk of resistance is determined for people in the first 3 months after starting ART or re-starting ART after an interruption of at least 3 months. Since in this early period on ART, the viral load will depend on the initial value the updated viral load is given as a reduction from the pre-ART maximum viral load. If the number of active drugs is three or more then at a high adherence level (above 0.8) the mean viral load change from the pre-ART maximum is 3 log copies/mL. To reflect the fact that there is variability in the response (Montaner 1998), the value for a given person is sampled from a Normal distribution with standard deviation 0.5. This viral load response diminishes both with decreasing number of active drugs in the regimen being started (which is informed by data from studies relating GSS to virologic outcome, as well as by studies of mono and dual therapy regimens (DeGruttola 2000;Eron 1995;Havlir 1995;Kuritzkes 1996;Larder 1995;Phillips 1997;Wittkop 2011;Wittkop 2013). The viral load response also diminishes with decreasing level of adherence (see Figure 5.12 and for example Genberg et al). As is well established, the CD4 count response generally mirrors the viral load response, although with very low numbers of active drugs and low adherence there is a mean decrease in CD4 count and still a small decrease in viral load from the maximum.

Figure 5.12 Model output: of people on ART, percent with current VL >500 according to current adherence. Comparison with data from Genberg at el on electronic monitoring-based adherence measures.

**
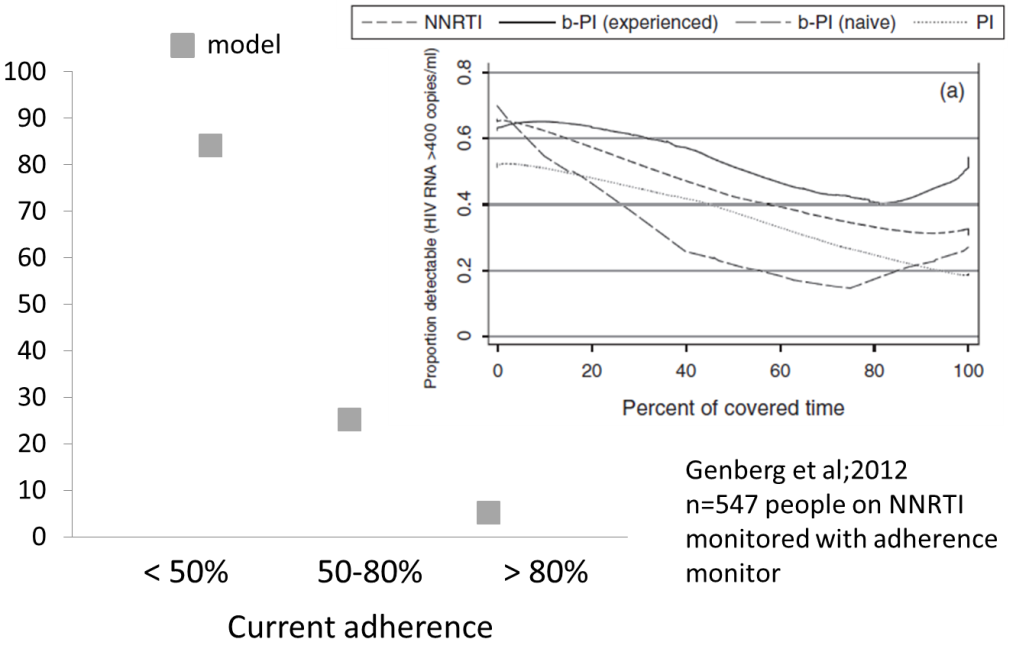
**

Regarding the risk of new drug resistant mutations arising, Tables Na-Nc provide a number for “new mutation risk” that is multiplied by the viral load (mean of values at t-1 and t) to give a probability used when assessing whether a new mutation(s) has/have arisen. Values of the new mutations risk have been chosen in conjunction with the translation of presence of mutations into reduced drug activity to provide estimates of resistance accumulation consistent with those observed in clinical practice (Gallant 2004;Harrigan 2005;Johannessen 2009;Ledergerber 1999;Phillips 2001;Phillips 2005;Staszewski 1999a;Staszewski 1999b;van Leth 2004). Risk of new resistance mutations arising increases with decreasing number of active drugs, reflecting the known greater risk of resistance with regimens less able to suppress viral replication, most clearly seen in the fact that mono and dual therapy regimens are highly susceptible to resistance development (Havlir 1995;Kuritzkes 1996;Larder 1995). At low adherence levels, the risk of resistance development is generally low regardless of the number of active drugs, as drug selection pressure is low. However, for those on NNRTI regimens the new resistance mutation risk is assumed to be that for the effective adherence category of 50 – 80% (i.e. maximal) even if the effective adherence is below 50%, reflecting the fact that NNRTI resistance develops easily, even when drug exposure is very low (Bangsberg 2004;Bangsberg 2006b).

*Viral load, CD4 count and risk of resistance between 3-6 months from (re-)starting ART*

For the period 3-6 months from (re-)start of ART (Table 5.2; to reduce the table content we do not provide the matrices of values for the resistance risk or CD4 count, only for the viral load – available in Cambiano et al 2014) we consider the adherence in both the current and previous 3 month period, since the likelihood of reaching viral suppression by 6 months will depend on adherence throughout the whole 6 month period from start of ART, although the adherence in the current period is assumed to be the stronger factor. By 6 months after starting ART, those on 3 or more active drugs with consistently high adherence generally reach a relatively high level of viral suppression, regardless of pre-ART maximal viral load, so a person’s viral load is no longer given by the change from baseline but the absolute level of viral load which it is likely they have reached. In these optimal conditions of high adherence and maximal active drugs we assume the viral load has a mean value of 0.5 log, again with variability between individuals. Since most viral load assays have a lower limit of quantification of 40 or 50 copies per mL, it is not actually known what the actual viral load level is, although highly sensitive assays suggest that a proportion of patients reach below 5 copies/mL (0.7 log copies/mL) (Doyle 2012). At lower numbers of active drugs and lower adherence, the viral load is still related to the maximal pre-ART viral load rather than being an absolute value, as the person’s viral load has not become so low that the initial value loses relevance. The viral load response decreases with a lower number of active drugs, lower current adherence, and lower adherence in the previous 3 month period. Values for the viral load response between those known from studies (high level of suppression for 3 active drugs and maximal adherence, and only around 0.5 log viral suppression when adherence is < 0.5 even with three active drugs (Gross 2001;Wittkop 2011) are imputed assuming a monotonic relationship. CD4 count responses again mirror the viral load response, as has been extensively studied in patients with ongoing viraemia on ART (Ledergerber 2004). Risk of new resistance mutations again increases with decreasing number of active drugs, if current adherence is in the middle or highest group. The only situation in which risk of new mutations is extremely low is when the number of active drugs is 3 or close to 3 and the current adherence is in the high category.

*Viral load, CD4 count and risk of resistance after 6 months of (re-)starting ART*

Table 5.3 shows how the viral load, CD4 count and risk of resistance is determined for the situation where a person has been on ART for more than 6 months and the viral load is suppressed or partially suppressed (< 4 log copies/mL). These values are similar to those used for the period 3-6 months from start of ART except that there is assumed to dependence on the adherence in the current 3 month period only.

The situation where the viral load is above 4 log copies /mL, 10,000 copies/mL is treated the same as that in the period 3-6 months from start of ART (described above), with adherence in the current and previous period having some influence.

Table 5.4 and Table 5.5 provide estimates from the model on various other outcomes at time points from start of ART.

**Table 5.1 Viral load (mean change from viral load max), CD4 count change (mean change between t-1 and t), and new mutation risk in first 3 months. For 0 active drugs, these are the changes regardless of time from start of ART. For viral load this is the mean of a Normal distribution with standard deviation 0.2, from which the patient's value/change is sampled. For the CD4 count patients vary in their underlying propensity for CD4 rise on ART (given by sampling from lognormal(1,0.5^2^) and the CD4 count change given here is multiplied by this factor. For the new mutation risk, this is a number that is multiplied by the viral load (mean of values at t-1 and t). The resulting probability is used when assessing whether a new mutation or mutations have arisen.**

|  | Effective adherence between t-1 & t | Number of active drugs | | | | | | | | | | | | |
| --- | --- | --- | --- | --- | --- | --- | --- | --- | --- | --- | --- | --- | --- | --- |
|  |  | 3 | 2.75 | 2.5 | 2.25 | 2.0 | 1.75 | 1.5 | 1.25 | 1 | 0.75 | 0.5 | 0.25 | 0 |
| Viral load | > 80% | -3.0 | -2.6 | -2.2 | -1.8 | -1.5 | -1.25 | -0.9 | -0.8 | -0.7 | -0.55 | -0.4 | -0.3 | -0.3 |
| (log change | > 50%, <80% | -2.0 | -1.6 | -1.2 | -1.1 | -0.9 | -0.8 | -0.6 | -0.5 | -0.4 | -0.25 | -0.1 | -0.05 | -0.1 |
| from vmax) | < 50% | -0.5 | -0.4 | -0.3 | -0.25 | -0.2 | -0.15 | 0.0 | +0.05 | +0.1 | +0.1 | +0.1 | +0.1 | 0.0 |
| CD4 count | > 80% | +50 | +45 | +40 | +35 | +30 | +25 | +20 | +17 | +13 | +10 | +5 | -2 | -15 |
| change | > 50%, <80% | +30 | +30 | +23 | +20 | +15 | +13 | +10 | +8 | +5 | +3 | 0 | -7 | -17 |
| (t-1 to t) | < 50% | +5 | +4 | +3 | +2 | +1 | -1 | -3 | -6 | -10 | -11 | -12 | -13 | -18 |
| New mutation | > 80% | 0.002 | 0.01 | 0.03 | 0.05 | 0.1 | 0.15 | 0.2 | 0.3 | 0.4 | 0.45 | 0.5 | 0.5 | 0.5 |
| Risk | > 50%, <80% | 0.15 | 0.15 | 0.2 | 0.25 | 0.3 | 0.3 | 0.3 | 0.35 | 0.4 | 0.45 | 0.5 | 0.5 | 0.5 |
| (x log viral load) | < 50%* | 0.15 | 0.15 | 0.2 | 0.25 | 0.3 | 0.3 | 0.3 | 0.35 | 0.4 | 0.45 | 0.5 | 0.5 | 0.5 |
|  | < 50%** | 0.05 | 0.05 | 0.05 | 0.05 | 0.05 | 0.05 | 0.05 | 0.05 | 0.05 | 0.05 | 0.05 | 0.05 | 0.05 |

* for NNRTI containing regimen, ** for boosted PI containing regimen.

Table 5.2 Summary of viral load (mean absolute value or mean change from viral load max) between 3-6 months, and after 6 months if viral load at t-1 > 4 logs. This is the mean of a Normal distribution with standard deviation 0.2, from which the patient's value/change is sampled.

| Effective adherence between t-2 & t-1 | Effective adherence between t-1 & t | Number of active drugs | | | | | | | | | | | |
| --- | --- | --- | --- | --- | --- | --- | --- | --- | --- | --- | --- | --- | --- |
|  |  | 3 | 2.75 | 2.5 | 2.25 | 2.0 | 1.75 | 1.5 | 1.25 | 1 | 0.75 | 0.5 | 0.25 |
| > 80% | > 80% | 0.5 | 0.8 | 1.2 | 1.4 | 2.0 | 2.7 | -1.7 | -1.15 | -0.9 | -0.75 | -0.6 | -0.4 |
| > 50%, <80% | > 80% | 1.2 | 1.2 | 1.2 | 1.4 | -2.0 | -1.6 | -1.2 | -1.05 | -0.9 | -0.7 | -0.5 | -0.35 |
| < 50% | > 80% | 1.2 | 1.2 | 1.2 | 1.4 | -2.0 | -1.6 | -1.2 | -1.0 | -0.9 | -0.7 | -0.5 | -0.2 |
| > 80% | > 50%, <80% | 1.2 | 1.6 | 1.8 | 2.2 | 2.4 | -2.4 | -1.5 | -0.9 | -0.7 | -0.55 | -0.4 | -0.3 |
| > 50%, <80% | > 50%, <80% | 2.5 | 2.5 | 2.5 | 2.5 | -1.2 | -1.1 | -0.8 | -0.65 | -0.5 | -0.35 | -0.2 | -0.05 |
| < 50% | > 50%, <80% | -2.0 | -1.8 | -1.5 | -1.35 | -1.2 | -1.1 | -0.8 | -0.65 | -0.5 | -0.2 | -0.2 | -0.05 |
| > 80% | < 50% | -0.5 | -0.4 | -0.3 | -0.25 | -0.2 | -0.15 | -0.10 | -0.05 | +0.0 | +0.0 | +0.0 | +0.0 |
| > 50%, <80% | < 50% | -0.5 | -0.4 | -0.3 | -0.25 | -0.2 | -0.15 | -0.10 | -0.05 | +0.0 | +0.0 | +0.0 | +0.0 |
| < 50% | < 50% | -0.5 | -0.4 | -0.3 | -0.25 | -0.2 | -0.15 | -0.10 | -0.05 | +0.0 | +0.0 | +0.0 | +0.0 |

Table 5.3 Summary of viral load (mean change from viral load max), CD4 count change (mean change between t-1 and t), and new mutation risk after 6 months, where viral load at t-1 < 4 logs. For viral load this is the mean of a Normal distribution with standard deviation 0.2, from which the patient's value/change is sampled. For the CD4 count patients vary in their underlying propensity for CD4 rise on ART (given by sampling from lognormal(1,0.5^2^) and the CD4 count change given here is multiplied by this factor. For the new mutation number, this is a number that is multiplied by the viral load (mean of values at t-1 and t). The resulting probability is used when assessing whether a new mutation or mutations have arisen.

|  | Effective adherence between t-1 & t | Number of active drugs | | | | | | | | | | | |
| --- | --- | --- | --- | --- | --- | --- | --- | --- | --- | --- | --- | --- | --- |
|  |  | 3 | 2.75 | 2.5 | 2.25 | 2.0 | 1.75 | 1.5 | 1.25 | 1 | 0.75 | 0.5 | 0.25 |
| Viral load | > 80% | 0.5 | 0.0 | 1.2 | 1.6 | -2.5 | -2.0 | -1.4 | -1.15 | -0.9 | -0.75 | -0.6 | -0.3 |
| (absolute value | > 50%, <80% | 1.2 | 1.2 | 1.2 | 1.4 | -1.2 | -1.0 | -0.7 | -0.6 | -0.5 | -0.4 | -0.3 | -0.1 |
| or log change | < 50% | -0.5 | -0.4 | -0.3 | -0.25 | -0.2 | -0.2 | -0.1 | -0.1 | -0.1 | -0.1 | -0.1 | -0.0 |
| from vmax) |  |  |  |  |  |  |  |  |  |  |  |  |  |
| CD4 count | > 80% | +30 | +28 | +25 | +23 | +21 | +19 | +3 | -5 | -9 | -10.5 | -12 | -12 |
| Change | > 50%, <80% | +15 | +13 | +10 | +8 | -4.5 | -7.5 | -10 | -12 | -13 | -14 | -15 | -15 |
| (t-1 to t) | < 50% | -13 | -14 | -15 | -15.5 | -16 | -16.5 | -17 | -17 | -18 | -17 | -17 | -17 |
| New mutation | > 80% | 0.002 | 0.01 | 0.03 | 0.08 | 0.10 | 0.15 | 0.2 | 0.3 | 0.4 | 0.45 | 0.5 | 0.5 |
| risk | > 50%, <80% | 0.15 | 0.18 | 0.2 | 0.25 | 0.3 | 0.3 | 0.3 | 0.35 | 0.4 | 0.45 | 0.5 | 0.5 |
| (x log viral load) | < 50%* | 0.15 | 0.18 | 0.2 | 0.25 | 0.3 | 0.3 | 0.3 | 0.35 | 0.4 | 0.45 | 0.5 | 0.5 |
|  | < 50%** | 0.05 | 0.05 | 0.05 | 0.05 | 0.05 | 0.05 | 0.05 | 0.05 | 0.05 | 0.05 | 0.05 | 0.05 |

* for NNRTI containing regimen, ** for boosted PI containing regimen.

Table 5.4 Model outputs of Status of people who started ART, according to time since initiation.

| Status | Number of years since ART | | | |
| --- | --- | --- | --- | --- |
|  | 1 | 3 | 5 | 10 |
| On ART VL < 500 | 76% | 65% | 56% | 39% |
| On ART VL < 500 no resistance | 3% | 3% | 2% | 1% |
| On ART VL > 500 with 1 or more resistance mutation | 7% | 8% | 6% | 5% |
| Off ART but under care | 3% | 3% | 3% | 2% |
| Off ART but not under care | 2% | 4% | 4% | 4% |
| Dead AIDS | 5% | 10% | 16% | 27% |
| Dead non-AIDS | 3% | 8% | 12% | 21% |

Table 5.5 Model-derived Kaplan-Meier estimates of proportion experiencing various outcomes by years from initiation of ART.

| Status | Years from start of ART | | | |
| --- | --- | --- | --- | --- |
|  | 1 | 3 | 5 | 10 |
| Viral load failure* | 9 | 17 | 25 | 38 |
| Resistance mutation (with virologic failure) | 8 | 14 | 18 | 25 |
| CD4 count rise of > 100/mm^3^ | 59 | 84 | 90 | 95 |
| CD4 count rise of > 200/mm^3^ | 17 | 63 | 77 | 88 |
| Interruption | 14 | 31 | 44 | 68 |
| Loss to follow-up | 4 | 10 | 15 | 28 |
| Death | 8 | 17 | 25 | 44 |

* considering viral load annually only, for consistency with countries where virologic failure monitored

**Variable patient-specific tendency for CD4 count rise on ART**

There is variability in the tendency for the CD4 count to rise on ART, for a given level of viral load suppression. For scenarios in the above tables (5.1 – 5.3) in which the CD4 count change is positive the CD4 count change is multiplied by this patient-specific factor (i.e. it is fixed for each patient), which is given by sampling for each patient from

Exp ( N(0, (*sd_patient_cd4_rise_art*)^2^)

sd_patient_cd4_rise_art = 0.2

To reflect the fact that the rate of CD4 count increase on ART tends to diminish with time, for those with patient-specific factor determining the CD4 count rise on ART > 1, this factor is modified by a factor 0.25 after 2 years of continuous treatment.

**Accelerated rate of CD4 count loss if PI not present in regimen**

The rate of change in CD4 count in people on failing regimens is largely based on data from the PLATO collaboration, for which patients were mainly on regimens containing a PI. If the regimen does not contain a PI the change in CD4 count per 3 months is modified (in the base model) by *poorer_cd4_rise_on_failing_nnrti* (= -6 /mm^3^). This applies regardless of viral load level, so PIs are assumed to lead to a more beneficial CD4 count change than NNRTIs (Ledergerber 2004).

**Variability in individual (underlying) CD4 counts for people on ART**

Once the mean of the underlying CD4 count is obtained as described above for people on ART, to obtain the CD4 count, variability (*sd_cd4* = 1.2) is added on the square root scale. The estimate was based on unpublished analyses

**Viral load and CD4 count changes during ART interruption**

Viral load returns to previous maximum viral load (vmax) in 3 months and adopts natural history changes thereafter.

CD4 rate of decline returns to natural history changes (ie those in ART naïve patients) after 9 months, unless the count remains > 200 above the CD4 nadir

Rate of CD4 count decline depends on current viral load. c(t) is the CD4 count at time t, cmin(t) is the CD4 count nadir measured by time t and cc(t-1) is the change in CD4 count from t-1 to t. v

if time off ART = 3 months or if time off ART > 3 months and CD4 in previous period is > 300 above the minimum CD4 count to date

v(t) = vmax(t-1)

if v(t) > 5 then cc(t-1) = Normal (-200,10^2^)

if 4.5 <= v(t) < 5 then cc(t-1) = Normal (-160,10^2^)

if v(t) < 4.5 then cc(t-1) = Normal (-120,10^2^)

If this leads to c(t) < cmin(t) (CD4 nadir) then c(t) is set to cmin(t)

if time off ART = 6 months:-

if v(t) > 5 then cc(t-1) = Normal (-100,10^2^)

if 4.5 <= v(t) < 5 then cc(t-1) = Normal (-90,10^2^)

if v(t) < 4.5 then cc(t-1) = Normal (-80,10^2^)

if time off ART = 9 months:-

if v(t) > 5 then cc(t-1) = Normal (-80,10^2^)

if 4.5 <= v(t) < 5 then cc(t-1) = Normal (-70,10^2^)

if v(t) < 4.5 then cc(t-1) = Normal (-60,10^2^)

This is broadly based on evidence from a number of analyses of the effects of ART interruption (e.g. d'Arminio Monforte 2005, Li X 2005, Mocroft 2001, Wit 2005)

**Incidence of new current toxicity and continuation of existing toxicity**

Toxicities including gastrointestinal symptoms, rash, hepatoxicity, CNS toxicity, lipodystrophy, hypersensitvity reaction, peripheral neuropathy and nephrolithiasis can occur with certain probability on certain specific drugs. These probabilities are based broadly on evidence from trials and cohort studies, although there are no common definitions for some conditions which complicates this.

Table 5.6 Risk of development of specific drug toxicities.

| **Toxicity** | **Drug** | **Risk of development per 3 months** | **Probability of continuation if pre-existing** |
| --- | --- | --- | --- |
| Nausea | Atazanavir, darunavir | 1% (5-fold higher in 1^st^ year) | 50% |
|  | Zidovudine, ddI, lopinavir | 3% (5-fold higher in 1^st^ year) | 50% |
| Diarrhoea | ddI | 5% (2.5-fold higher in 1^st^ year) | 50% |
|  | Lopinavir | 2% (2.5-fold higher in 1^st^ year) | 50% |
|  | Atazanavir, darunavir | 1% (2.5-fold higher in 1^st^ year) | 50% |
| Rash | efavirenz | 3% (in first 6 months on efavirenz) |  |
|  | nevirapine | 10% (in first 6 months on nevirapine) |  |
| CNS toxicity | efavirenz | 10% (if been on efavirenz <1 year) | 80% if been on efavirenz <1 year. 90% if been on efavirenz ≥1 year |
|  | dolutegravir | 5% (if been on dolutegravir <1 year) | 40% if been on dolutegravir <1 year. 90% if been on dolutegravir ≥1 year |
| Lipodystrophy | d4T | 5% | 100% |
|  | Zidovudine | 1.5% | 100% |
| Peripheral neuropathy | d4T | 2% (1.5-fold higher in 1^st^ year) | 100% (if remain on d4T) |
|  | ddI | 1% (1.5-fold higher in 1^st^ year) | 100% (if remain on ddI) |
| Acute hepatitis | nevirapine | 2% (one off risk in 1^st^ and 2^nd^ 3 month periods) |  |
| Anaemia | zidovudine | 3% (1.5-fold higher in 1^st^ year) | 20% |
| Headache | ZDV | 10% (1.5-fold higher in 1^st^ year) | 40% |
| Pancreatitis | D4T, DDI | 0.02% (1.5-fold higher in 1^st^ year) | 100% |
| Lactic acidosis | Zidovudine, d4T, ddI | 0.02% |  |
| Renal dysfunction | tenofovir | 0.35% | 100% |

**Switching of drugs due to toxicity**

If toxicity is present then individual drugs may be switched due to toxicity (nevirapine for efavirenz, zidovdine for tenofovir). ddI is only used if neither zidovudine and tenofovir are available due to toxicity.

# Emergence of specific resistance mutations and their effect on drug activity

**Accumulation of resistance mutations**

*newmut* (see Table 5.3 - Table 5.5 above) is a probability used to indicate the level of risk of new mutations arising in a given 3 month period. If this chance comes up in a given 3 month period (determined by sampling from the binomial distribution) then the following criteria operate.

Table 6.1 Risk of acquiring new resistance mutations.

| **Resistance mutation** | **Probability**  **of arising** | **Conditions** |
| --- | --- | --- |
| M184 | 80% | if (on 3TC) |
| # TAMS increases by 1 | 20% | if (on ZDV or d4T) and (not on 3TC nor FTC) |
|  | 12% | if (on ZDV or d4T) and (on 3TC or FTC) |
| # TAMS increases by 2 | 1% | if (on ZDV or d4T) and (not on 3TC nor FTC) |
|  | 1% | if (on ZDV or d4T) and (on 3TC or FTC) |
| K65 | 2% | if (on tenofovir or ddI) and (on zidovudine or d4T) |
|  | 10% | If (on tenofovir or ddI) and (not on zidovudine nor d4T) |
| L74 | 1% | if (on ddI) |
| Q151 | 2% | if (on ddI or d4T or zidovudine) |
| K103 | 20% | If on nevirapine |
|  | 60% | If on efavirenz |
| Y181 | 40% | If on nevirapine |
|  | 10% | If on efavirenz |
| G190 | 20% | If on nevirapine |
|  | 10% | If on efavirenz |
| V32 | 1% | if on lopinavir |
| M46 | 2% | if on lopinavir |
| I47 | 1% | If on lopinavir |
| I50L | 3% | If on atazanavir |
| I50V | 1% | If on darunavir |
| I54 | 2% | If on lopinavir |
|  | 1% | If on darunavir |
| L76 | 2% | If on lopinavir |
|  | 1% | If on darunavir |
| V82 | 2% | If on lopinavir |
| I84 | 1% | If on darunavir |
|  | 3% | If on atazanavir |
| N88 | 3% | If on atazanavir |

These values are chosen, in conjunction with values of *newmut{t}*, to provide estimates of accumulation of specific classes of mutation consistent with those observed in clinical practice (UK Drug Resistance Database 2005, Harrigan 2005, Sigaloff 2012). They reflect a greater propensity for some mutations to arise than others. This probably relates to the ability of the virus to replicate without the mutations (e.g. probably very low in the presence of 3TC for virus without M184V) as well as the replicative capacity of virus with the mutations. Over time as more data accumulate it may be possible improve these estimates of rates of accumulation of specific mutations.

**New resistance to NNRTI arising as a result of ART interruption**

It is assumed that due to the long half life of NNRTIs nevirapine and efavirenz, stopping of a regimen containing one of these drugs is associated with a specific probability of an NNRTI resistance mutation arising (see, for example, Fox et al, 2008). The respective probabilities for K103, Y181 and G190 are 1.8%, 0.06% and 0.6%.

**Loss of acquired mutations from majority virus**

It is assumed that mutations tend to be lost from majority virus with a certain probability from 3 months after stopping to take a drug that selects for that mutation. The probability of losing mutations per 3 months (from 3 months after stopping) is as follows (Devereux 1999, Devereux 2001, Deeks 2003, Birk 2001, Walter 2002, Hance 2001, Tarwater PM 2003)

Table 6.2 Probability of loss of acquired mutations from majority virus per 3 months after stopping drugs selecting for mutation.

----------------------------------------

M184V 0.8

L74V 0.6

Q151M 0.6

K65R 0.6

TAMS (lose all) 0.4

NNRTI mutations 0.05

Protease mutations 0.2

---------------------------------------

Mutations are regained in majority virus if a drug selecting for the mutation is again started.

**Determination of level of resistance to each drug**

Table 6.3 shows the level of resistance to each drug according to presence of specific resistance mutations.

Table 6.3 Level of resistance to each drug according to presence of specific resistance mutations.

| **Resistance mutation** | **Drug** | **Level of resistance**  **(1=full resistance)** | **Condition** |
| --- | --- | --- | --- |
| M184 | 3TC or FTC | 0.75 |  |
| 1-2 TAMS | zidovudine or d4T | 0.5 | No 3TC or FTC in regimen |
|  | zidovudine or d4T | 0.25 | 3TC or FTC in regimen and ever had M184V |
|  | zidovudine or d4T | 0.5 | 3TC or FTC in regimen and never had M184V |
| 2-3 TAMS | tenofovir | 0.5 | No 3TC or FTC in regimen, or 3TC in the regimen and never had M184V |
|  | tenofovir | 0.5 | 3TC or FTC in regimen and ever had M184V |
| 3-4 TAMS | zidovudine or d4T | 0.75 | No 3TC or FTC in regimen |
|  | zidovudine or d4T | 0.5 | 3TC or FTC in regimen and ever had M184V |
|  | zidovudine or d4T | 0.75 | 3TC or FTC in regimen and never had M184V |
| 3 or more TAMS | ddI | 0.5 |  |
| 4 or more TAMS | tenofovir | 0.75 | No 3TC or FTC in regimen, or 3TC in the regimen and never had M184V |
|  | tenofovir | 0.5 | 3TC or FTC in regimen and ever had M184V |
|  | tenofovir | 0.75 | 3TC or FTC in regimen and never had M184V |
| 5 or more TAMS | zidovudine or d4T | 1.0 | No 3TC or FTC in regimen |
|  | zidovudine or d4T | 0.75 | 3TC or FTC in regimen and ever had M184V |
|  | zidovudine or d4T | 0.75 | 3TC or FTC in regimen and never had M184V |
| Q151 | 3TC or FTC | 0.25 |  |
|  | Zidovudine, d4T ddI | 0.75 |  |
| K65 | 3TC or FTC | 0.25 |  |
|  | d4T | 0.5 |  |
|  | tenofovir or ddI | 0.75 |  |
| K103 | nevirapine or efavirenz | 1.0 |  |
| Y181 | Nevirapine | 1.0 |  |
|  | efavirenz | 0.75 |  |
| G190 | Nevirapine | 1.0 |  |
|  | Efavirenz | 0.75 |  |
| I47 | Lopinavir | 0.75 |  |
| I501 | Atazanavir | 1.0 |  |
| N88 | Atazanavir | 1.0 |  |
| I84 | Atazanavir | 1.0 |  |
| 1 or 2 or 3 of (V32, M46, I54, V82, L90) | Atazanavir | 0.5 |  |
| At least 4 of (V32, M46, I54, V82, L90) | Atazanavir | 1.0 |  |
| 1 of (V32, L76, V82) | Lopinavir | 0.25 | Never had I47 |
| 2 of (V32, L76, V82) | Lopinavir | 0.5 | Never had I47 |
| 3 of (V32, L76, V82) | Lopinavir | 0.75 | Never had I47 |
| All of (V32, I47, L76, V82) | Lopinavir | 1.0 |  |
| 4 of (M46, V82, I84, L90) | Lopinavir | Max(level of resistance as above in this table, 0.5) |  |
| 2 or 3 of (M46, V82, I84, L90) | lopinavir | Max(level of resistance as above in this table, 0.25) |  |
| 2 of (V32, I47, I50, I54, L76, I84) | darunavir | 0.25 |  |
| 3 of (V32, I47, I50, I54, L76, I84) | darunavir | 0.50 |  |
| At least 4 of (V32, I47, I50, I54, L76, I84) | darunavir | 0.75 |  |

These rules approximately follow the interpretation systems for conversion of mutations present on genotypic resistance test into a predicted level of drug activity (or, equivalently, of resistance; <http://www.rega.kuleuven.be>, <http://hivdb.stanford.edu>, <http://www.hivfrenchresistance.org/>

**Calculation of activity level of each drug**

This is given by 1-level of resistance. For ritonavir boosted PIs it is given by 2 – (2 x level of resistance); i.e. assumed higher potency due to ability to induce sustained viral suppression alone. Activity levels of each drug in the regimen are summed to give the total number of active drugs.

# Interventions and populations

**Circumcision**

There are four instances in which circumcision can occur within the model:

It is assumed 5% of men of any age, including those under 15 at t=1 (1989) have previously been circumcised, defined by variable *prev_circ*.

It is further assumed 5% of the male population get circumcised at birth from 1989 onwards, defined by *birth_circ*

Uncircumcised boys aged 11-15 have 0.3% probability of being circumcised per 3 months prior to 2013; after 2013 this probability increases to 4%, reflecting the circumcision program rolled out in schools (GARCPR 2015)

From 2008 onwards, circumcision can occur amongst those aged >15, reflecting circumcision intervention programmes. If the chance of getting circumcised is realised and there is no previous HIV diagnosis, a HIV test will be performed and if negative, circumcision will occur. This is defined by age and time varying probabilities as described below.

The baseline probability of being circumcised (*prob_circ*) for those aged >15 after 2008 is defined as follows:

If 2008 < year < 2017 then:

If 14.75 < age{t} <20 then *prob_circ* = (*caldate{t}* -mc_int)^2^ * circ_inc_rate * 0.8

If 20 < age{t} <25 then *prob_circ* = (*caldate{t}*-mc_int)^2^ * circ_inc_rate * 0.6

If 25 < age{t} <30 then *prob_circ* = (*caldate{t}*-mc_int)^2^ * circ_inc_rate * 0.4

If 30 < age{t} <40 then *prob_circ* = (*caldate{t}*-mc_int)^2^ * circ_inc_rate * 0.2

If 40 < age{t} <50 then *prob_circ* = (*caldate{t}*-mc_int)^2^ * circ_inc_rate * 0.2

Where *circ_inc_rate =0.0007,* and is an increase in the rate of circumcision, *caldate* is the current 3 month time period and *mc_int* refers to the year of introduction of voluntary medical male circumcision (*2008*). Circumcision is determined by sampling from a Uniform (0,1) distribution and ascertaining whether this is below *prob_circ*. The number of circumcisions per year is given in the *Description of Model Calibration* document.

**Female sex workers**

In the model we distinguish between female sex workers (FSW) having condomless sex (CLS) and female sex workers who consistently use a condom (CCU). The term ‘female sex workers’ is debatable; these women are sometimes referred to ‘women having transactional sex’, amongst other varying terms. The definition of FSW is also debatable; for the purposes of the model, we first determine the number of condomless sex partners each person has in each three month period and define FSW as women with more than 3 condomless sex partners in a 3 month period in the last year, and this is notated by *whr{t}* in the model.

It is assumed that there is a certain rate of becoming CCU FSW (from 1995, before it is assumed condoms are not widely used) and a certain rate of stopping being a CCU FSW. The rate (per three months) of becoming a CCU FSW is sampled from a Uniform distribution from 0 to 0.002, and is multiplied by 0.33 in women aged 30 to 39 and by 0.20 in women 40 to 64. The rate of interruption applies to women who are CCUFSW and is assumed to be 0.025 per 3 months. The concept of CCU FSW is introduced so that we can mimic the introduction of interventions aimed at FSW as a whole (those having condomless sex and those not).

**Pregnant women**

It is assumed that 5% of all women are unable to ever be defined as being pregnant. Of the remaining 95% of women, the base rate of pregnancy relating to women aged 35-45 who had condomless sex in the previous 3 month period, *prob_pregnancy_base* is 0.085. This is multiplied by age specific probabilities, *fold_preg* to reflect lower likelihood of pregnancy in older women. The multiplicative factors for women ages 15-25, 25-35, 45-55, and 55-65 are 2, 1.9, 0.2 and 0 respectively. If women had condomless sex with a short-term partner, the probability of pregnancy is multiplied by the factor *fold_tr_newp (=0.3)*, to take into account the lower number of sex acts per short term partner than per long term partner. Pregnancy is defined as occurring only in one 3-month period (the period at the end of pregnancy) and hence is dependent on condomless sex in the period t-3.

# Risk of clinical disease and death in HIV infected people

**Occurrence of WHO 4 diseases**

The rate of WHO 4 diseases according to CD4 count per 3 months is given below.

**Table 7.1 Rate of WHO stage 4 disease according to CD4 count and viral load.**

| If cd4 > 650 | rate=0.002 | if 500 < cd4 < 650 | rate=0.010 |
| --- | --- | --- | --- |
| if 450 < cd4 < 500 | rate=0.013 | if 400 < cd4 < 450 | rate=0.016 |
| if 375 < cd4 < 400 | rate=0.020 | if 350 < cd4 < 375 | rate=0.022 |
| if 325 < cd4 < 350 | rate=0.025 | if 300 < cd4 < 325 | rate=0.030 |
| if 275 < cd4 < 300 | rate=0.037 | if 250 < cd4 < 275 | rate=0.045 |
| if 225 < cd4 < 250 | rate=0.055 | if 200 < cd4 < 225 | rate=0.065 |
| if 175 < cd4 < 200 | rate=0.080 | if 150 < cd4 < 175 | rate=0.10 |
| if 125 < cd4 < 150 | rate=0.13 | if 100 < cd4 < 125 | rate=0.17 |
| if 90 < cd4 < 100 | rate=0.20 | if 80 < cd4 < 90 | rate=0.23 |
| if 70 < cd4 < 80 | rate=0.28 | if 60 < cd4 < 70 | rate=0.32 |
| if 50 < cd4 < 60 | rate=0.40 | if 40 < cd4 < 50 | rate=0.50 |
| if 30 < cd4 < 40 | rate=0.80 | if 20 < cd4 < 30 | rate=1.10 |
| if 10 < cd4 < 20 | rate=1.80 | if 0 < cd4 < 10 | rate=2.50 |
|  |  |  |  |
| Independent effect of viral load | |  |  |
| if v < 3 | rate = rate x 0.2 |  |  |
| if 3 <= v < 4 | rate = rate x 0.3 |  |  |
| if 4 <= v < 4.5 | rate = rate x 0.6 |  |  |
| if 4.5 <= v < 5 | rate = rate x 0.9 |  |  |
| if 5 <= v < 5.5 | rate = rate x 1.2 |  |  |
| if 5.5 <= v | rate = rate x 1.6 |  |  |

This is informed by Phillips AIDS 2004.

**Independent effect of age**

rate = rate x (age / 38)^1.2^

**Independent effect of PJP prophylaxis**

If patient on PJP prophylaxis then this rate is multiplied by 0.8.

If CD4 count is measured and current value < 350 /mm3 then patient assumed to have 80% chance of starting PJP prophylaxis after 1996

If patient has current WHO stage 3 or 4 condition they are assumed to have an 80% chance of starting PJP prophylaxis

If CD4 count is measured then PJP prophylaxis assumed to stop if current value > 350/mm3.

If the patient has been continuously on ART for 2 years with no WHO 3 or 4 condition in previous 6 months then it is assumed that PJP prophylaxis is stopped.

**Independent effect of being on ART**

For patients on a single drug regimen this risk is multiplied by 0.9, for patients on a two drug regimen it is multiplied by 0.85 and for patients on a 3 drug regimen it is multiplied by 0.6, to reflect that being on ART has a positive effect on risk of AIDS and death independent of latest CD4 count and viral load.

**Occurrence of WHO 3 diseases**

As for WHO 4 except risk is fold_incr_who3 (= 5) higher.

**Risk of HIV-related death**

As for WHO 4 except risk *fold_decr_hivdeath* - fold lower (= 0.25).

CD4-, viral load- age-specific death rate raised *incr_death_rate_tb*-fold (= 10) if current TB and *incr_death_rate_adc*-fold (= 10) if current WHO 4 disease. We assume 15% of HIV-related deaths (ie not including deaths that arise due to background mortality rates) are classified as non-HIV-related.

References

Phillips AN, Pillay D, Garnett G, et al. Effect on transmission of HIV-1 resistance of timing of implementation of viral load monitoring to determine switches from first to second-line regimens in resource-limited settings. AIDS 2011; 25: 843–50.

Cambiano V, Bertagnolio S, Jordan M, et al. Transmission of Drug Resistant HIV and Its Potential Impact on Mortality and Treatment Outcomes in Resource-Limited Settings. J Infect Dis 2013; 207: S57-62

Cambiano V, Bertagnolio S, Jordan M, Pillay D, Perriens J, Venter F, et al. Predicted levels of HIV drug resistance: potential impact of expanding diagnosis, retention, and eligibility criteria for antiretroviral therapy initiation. AIDS 2014, 28 (Suppl 1):S15–S23.

CIA world factbook (2014) <https://www.cia.gov/library/publications/the-world-factbook/geos/zi.html>

Fonner VA, Denison J, Kennedy CE, O’Reilly K, Sweat M. Voluntary counseling and testing (VCT) for changing HIVrelated risk behavior in developing countries. *Cochrane Database of Systematic Reviews* 2012, Issue 9. Art. No.: CD001224. DOI:10.1002/14651858.CD001224.pub4.

DHS Zimbabwe. <http://dhsprogram.com/Publications/Publication-Search.cfm?ctry_id=48&c=Zimbabwe&Country=Zimbabwe&cn=Zimbabwe>

Mbizvo MT, Kasule J, Mahomed K, Nathoo K. HIV-1 seroconversion incidence following pregnancy and delivery among women seronegative at recruitment in Harare, Zimbabwe. Cent Afr J Med 2001 May; 47(5):115-8.

Humphrey JH, Hargrove JW, Malaba LC, et al. HIV incidence among post-partum women in Zimbabwe: risk factors and the effect of vitamin A supplementation. AIDS 2006 Jun 26; 20(10):1437-46.

Mbizvo MT, Machekano R, McFarland W, et al. HIV seroincidence and correlates of seroconversion in a cohort of male factory workers in Harare, Zimbabwe. AIDS 1996 Jul; 10(8):895-901.

Corbett EL, Makamure B, Cheung YB, et al. HIV incidence during a cluster-randomized trial of two strategies providing voluntary counselling and testing at the workplace, Zimbabwe. AIDS 2007 Feb 19; 21(4):483-9.

Johnson LF, et al. Sexual behaviour patterns in South Africa and their association with the spread of HIV: Insights from a mathematical model. Demographic Research 2009; 21:289-340.

Gregson S et al. Methods to reduce social desirability bias in sex surveys in low-development settings - Experience in Zimbabwe. Sexually Transmitted Diseases 2002; 29: 568-575.

Gregson S, Gonese E, Hallett TB, et al. HIV decline in Zimbabwe due to reductions in risky sex? Evidence from a comprehensive epidemiological review. Int J Epid 2010;39:1311–1323.

Halperin DT, Mugurungi O, Hallett TB, Muchini B, Campbell B, et al. (2011) A Surprising Prevention

Success: Why Did the HIV Epidemic Decline in Zimbabwe? PLoS Med 8(2): e1000414. doi:10.1371/

journal.pmed.1000414

Hollingsworth TD, Anderson RM, Fraser C. HIV-1 transmission, by stage of infection. J Infect Dis 2008; 198:687–93

Cohen MS. Sexually transmitted diseases enhance HIV transmission: no longer a hypothesis

Lancet 1998; 351 : 5.

Nicolosia A. The efficiency of male-to-female and female-to-male sexual transmission of the human-immunodeficiency-virus -a study of 730 stabke couples. Epidemiology 1994; 5 : 570 1994

Castro H, Pillay D, Cane P, Asboe A, Cambiano V, Phillips AN, Dunn DT. Persistence of Transmitted HIV-1 Drug Resistance Mutations. JID 2013; DOI: 10.1093/infdis/jit345

Smith DM, Wong JK, Hightower GK, et al. HIV drug resistance acquired through superinfection. AIDS 2005; 19: 1251–56.

Phillips AN, Cambiano V, Nakagawa F, Brown AE, Lampe F, et al. (2013) Increased HIV Incidence in Men Who Have Sex with Men Despite High Levels of ART-Induced Viral Suppression: Analysis of an Extensively Documented Epidemic. PLoS ONE 8(2): e55312. doi:10.1371/journal.pone.0055312

Corvasce et al. Evidence of differential selection of HIV-1 variants carrying drug-resistant mutations in seroconverters. Antiviral Therapy 2006; 11:329 -334.

Turner et al. Diminished Representation of HIV-1 Variants Containing Select Drug Resistance–Conferring mutations in Primary HIV-1 Infection. JAIDS 2004; 37: 1627-1631)

Dunkle KL, et al. New heterosexually transmitted HIV infections in married or cohabiting couples in urban Zambia and Rwanda: an analysis of survey and clinical data. Lancet 2008; 371:2183-2191.

Phillips AN, Pillay D, Miners AH, Bennett DE, Gilks CF, Lundgren JD. Outcomes from monitoring of patients on antiretroviral therapy in resource-limited settings with viral load, CD4 cell count, or clinical observation alone: a computer simulation model. *Lancet* 2008; 371: 1443–51.

Nakagawa F, Lodwick RK, Smith CJ, Smith R, Cambiano V, Lundgren JD, et al. Projected life expectancy of people with HIV according to timing of diagnosis. AIDS 2012;26(3):335-43.

Nakagawa F, Miners A, Smith CJ, Simmons R, Lodwick RK, Cambiano V, et al. (2015) Projected

Lifetime Healthcare Costs Associated with HIV Infection. PLoS ONE 10(4): e0125018. doi:10.1371/

journal.pone.0125018

Pantazis N, Touloumi G. Bivariate modelling of longitudinal measurements of two human immunodeficiency type 1 disease progression markers in the presence of informative drop-outs. JRSS C 2005; 54: 405-423.

Sabin CA, Devereux H, Phillips AN, et al. Course of viral load throughout HIV-1 infection. JAIDS 2000; 23:172-177.

Hubert J-B, Burgard M, Dussaix E, et al. Natural history of serum HIV-1 RNA levels in 330 patients with known date of infection. AIDS 2000; 14:123-131.

O'Brien TR, Rosenberg PS, Yellin F, et al. Longitudinal HIV-1 RNA levels in a cohort of homosexual men. JAIDS 1998; 18:155-161.

Henrard DR, Phillips JF, Muenz LR et al. Natural history of HIV-1 cell-free viraemia. JAMA 1995; 274: 554-558.

Lyles RH, Munoz A, Yamashita TE, et al. Natural history of human immunodeficiency virus type 1 viraemia after seroconversion and proximal to AIDS in a large cohort of homosexual men. J Infect Dis 2000; 181 (3): 872-880.

Touloumi G, Pantazis N, Babiker AG, et al. Differences in HIV RNA levels before the initiation of antiretroviral therapy among 1864 individuals with known HIV-1 seroconversion dates. AIDS 2004; 18 (12): 1697-1705.

Mellors JW, Munoz A, Giorgi JV, et al. Plasma viral load and CD4(+) lymphocytes as prognostic markers of HIV-1 infection. Ann Intern Med 1997; 126 (12): 946-954.

Koot M, Keet IPM, Vos AHV, et al. Prognostic value of human HIV-1 biological phenotype for the rate of CD4+ cell depletion and progression to AIDS. Ann Intern Med 1993; 118: 681-688.

Darby SC, Ewart DW, Giangrande PLF, et al. Importance of age at infection with HIV-1 for survival and development of AIDS in UK haemophilia population. Lancet 1996; 347: 1573–79.

Wanyenze RK, Hahn JA, Liechty CA, Ragland K, Ronald A, Mayanja-Kizza H, et al. Linkage to HIV care and survival following inpatient HIV counseling and testing. AIDS Behav 2011; 15(4):751-760.

Bassett IV, Giddy J, Nkera J, Wang B, Losina E, Lu Z, et al. Routine voluntary HIV testing in Durban, South Africa - The experience from an outpatient department. Jaids-Journal of Acquired Immune Deficiency Syndromes 2007; 46(2):181-186.

Hensen B, Baggaley R, Wong VJ, Grabbe KL, Shaffer N, Lo YR, et al. Universal voluntary HIV testing in antenatal care settings: a review of the contribution of provider-initiated testing & counselling. Trop Med Int Health 2012; 17(1):59-70.

Sabapathy K, Van den Bergh R, Fidler S, Hayes R, Ford N. Uptake of Home-Based Voluntary HIV Testing in Sub-Saharan Africa: A Systematic Review and Meta-Analysis. Plos Medicine 2012; 9(12)

Cambiano V, Ford D, Mabugu T, et al. Assessment of the potential impact and cost-effectiveness of self-testing for HIV in low-income countries. J Infect Dis 2015 DOI: 10.1093/infdis/jiv040

Fox, M.P., Cutsem, G.V., Giddy, J., Maskew, M., Keiser, O., Prozesky, H., Wood, R., Hernan, M.A., Sterne, J.A., Egger, M., & Boulle, A. 2012. Rates and predictors of failure of first-line antiretroviral therapy and switch to second-line ART in South Africa. J.Acquir.Immune.Defic.Syndr., 60, (4) 428-437 available from: PM:22433846

Johnston, V., Fielding, K.L., Charalambous, S., Churchyard, G., Phillips, A., & Grant, A.D. 2012. Outcomes following virological failure and predictors of switching to second-line antiretroviral therapy in a South African treatment program. J.Acquir.Immune.Defic.Syndr., 61, (3) 370-380 available from: PM:22820803

Cambiano, V., Lampe, F.C., Rodger, A.J., Smith, C.J., Geretti, A.M., Lodwick, R.K., Puradiredja, D.I., Johnson, M., Swaden, L., & Phillips, A.N. 2010a. Long-term trends in adherence to antiretroviral therapy from start of HAART. *AIDS*, 24, (8) 1153-1162 available from: PM:20299959

Carrieri, P., Cailleton, V., Le, M., V, Spire, B., Dellamonica, P., Bouvet, E., Raffi, F., Journot, V., & Moatti, J.P. 2001. The dynamic of adherence to highly active antiretroviral therapy: results from the French National APROCO cohort. *J.Acquir.Immune.Defic.Syndr.*, 28, (3) 232-239 available from: PM:11694829

El-Khatib, Z., Ekstrom, A.M., Coovadia, A., Abrams, E.J., Petzold, M., Katzenstein, D., Morris, L., & Kuhn, L. 2011. Adherence and virologic suppression during the first 24 weeks on antiretroviral therapy among women in Johannesburg, South Africa - a prospective cohort study. *BMC.Public Health*, 11, 88 available from: PM:21303548

Genberg, B.L., Wilson, I.B., Bangsberg, D.R., Arnsten, J., Goggin, K., Remien, R.H., Simoni, J., Gross, R., Reynolds, N., Rosen, M., & Liu, H. 2012. Patterns of antiretroviral therapy adherence and impact on HIV RNA among patients in North America. *AIDS*, 26, (11) 1415-1423 available from: PM:22767342

Glass, T.R., Battegay, M., Cavassini, M., De, G.S., Furrer, H., Vernazza, P.L., Hirschel, B., Bernasconi, E., Rickenbach, M., Gunthard, H.F., & Bucher, H.C. 2010. Longitudinal analysis of patterns and predictors of changes in self-reported adherence to antiretroviral therapy: Swiss HIV Cohort Study. *J.Acquir.Immune.Defic.Syndr.*, 54, (2) 197-203 available from: PM:20035231

Kleeberger, C.A., Buechner, J., Palella, F., Detels, R., Riddler, S., Godfrey, R., & Jacobson, L.P. 2004. Changes in adherence to highly active antiretroviral therapy medications in the Multicenter AIDS Cohort Study. *AIDS*, 18, (4) 683-688 available from: PM:15090774

Lazo, M., Gange, S.J., Wilson, T.E., Anastos, K., Ostrow, D.G., Witt, M.D., & Jacobson, L.P. 2007. Patterns and predictors of changes in adherence to highly active antiretroviral therapy: longitudinal study of men and women. *Clin.Infect.Dis.*, 45, (10) 1377-1385 available from: PM:17968839

Levine, A.J., Hinkin, C.H., Castellon, S.A., Mason, K.I., Lam, M.N., Perkins, A., Robinet, M., Longshore, D., Newton, T., Myers, H., Durvasula, R.S., & Hardy, D.J. 2005. Variations in patterns of highly active antiretroviral therapy (HAART) adherence. *AIDS Behav.*, 9, (3) 355-362 available from: PM:16088365

Mannheimer, S., Friedland, G., Matts, J., Child, C., & Chesney, M. 2002. The consistency of adherence to antiretroviral therapy predicts biologic outcomes for human immunodeficiency virus-infected persons in clinical trials. *Clin.Infect.Dis.*, 34, (8) 1115-1121 available from: PM:11915001

Meresse, M., March, L., Kouanfack, C., Bonono, R.C., Boyer, S., Laborde-Balen, G., Aghokeng, A., Suzan-Monti, M., Delaporte, E., Spire, B., Carrieri, M.P., & Laurent, C. 2014. Patterns of adherence to antiretroviral therapy and HIV drug resistance over time in the Stratall ANRS 12110/ESTHER trial in Cameroon. *HIV.Med.* available from: PM:24589279

Osterberg, L. & Blaschke, T. 2005. Adherence to Medication. *NEJM*, 353, 487-497

Muyingo, S.K., Walker, A.S., Reid, A., Munderi, P., Gibb, D.M., Ssali, F., Levin, J., Katabira, E., Gilks, C., & Todd, J. 2008. Patterns of individual and population-level adherence to antiretroviral therapy and risk factors for poor adherence in the first year of the DART trial in Uganda and Zimbabwe. *J.Acquir.Immune.Defic.Syndr.*, 48, (4) 468-475 available from: PM:18614918

Bangsberg DR, Moss AR, Deeks SG et al. Paradoxes of adherence and drug resistance to HIV antiretroviral therapy. J Antimicrob Chem 2004; 53 (5): 696-699.

Bangsberg, D.R., Acosta, E.P., Gupta, R., Guzman, D., Riley, E.D., Harrigan, P.R., Parkin, N., & Deeks, S.G. 2006b. Adherence-resistance relationships for protease and non-nucleoside reverse transcriptase inhibitors explained by virological fitness. *AIDS*, 20, (2) 223-231 available from: PM:16511415

Hamers, R.L., Wallis, C.L., Kityo, C., Siwale, M., Mandaliya, K., Conradie, F., Botes, M.E., Wellington, M., Osibogun, A., Sigaloff, K.C., Nankya, I., Schuurman, R., Wit, F.W., Stevens, W.S., van, V.M., & de Wit, T.F. 2011. HIV-1 drug resistance in antiretroviral-naive individuals in sub-Saharan Africa after rollout of antiretroviral therapy: a multicentre observational study. *Lancet Infect.Dis.*, 11, (10) 750-759 available from: PM:21802367

Hassan, A.S., Nabwera, H.M., Mwaringa, S.M., Obonyo, C.A., Sanders, E.J., Rinke de Wit, T.F., Cane, P.A., & Berkley, J.A. 2014. HIV-1 virologic failure and acquired drug resistance among first-line antiretroviral experienced adults at a rural HIV clinic in coastal Kenya: a cross-sectional study. *AIDS Res.Ther.*, 11, (1) 9 available from: PM:24456757

Hoffmann, C.J., Charalambous, S., Grant, A.D., Morris, L., Churchyard, G.J., & Chaisson, R.E. 2014. Durable HIV RNA resuppression after virologic failure while remaining on a first-line regimen: a cohort study. *Trop.Med.Int.Health*, 19, (2) 236-239 available from: PM:24588012

Kobin, A.B. & Sheth, N.U. 2011. Levels of adherence required for virologic suppression among newer antiretroviral medications. *Ann.Pharmacother.*, 45, (3) 372-379 available from: PM:21386024

Li, J.Z., Gallien, S., Ribaudo, H., Heisey, A., Bangsberg, D.R., & Kuritzkes, D.R. 2014. Incomplete adherence to antiretroviral therapy is associated with higher levels of residual HIV-1 viremia. *AIDS*, 28, (2) 181-186 available from: PM:24361679

Mackie, N.E., Phillips, A.N., Kaye, S., Booth, C., & Geretti, A.M. 2010. Antiretroviral drug resistance in HIV-1-infected patients with low-level viremia. *J.Infect.Dis.*, 201, (9) 1303-1307 available from: PM:20350161

Rosenblum, M., Deeks, S.G., van der Laan, M., & Bangsberg, D.R. 2009. The risk of virologic failure decreases with duration of HIV suppression, at greater than 50% adherence to antiretroviral therapy. *PLoS.One.*, 4, (9) e7196 available from: PM:19787058

Tran, D.A., Wilson, D.P., Shakeshaft, A., Ngo, A.D., Doran, C., & Zhang, L. 2014. Determinants of virological failure after 1 year's antiretroviral therapy in Vietnamese people with HIV: findings from a retrospective cohort of 13 outpatient clinics in six provinces. *Sex Transm.Infect.* available from: PM:24619575

Usitalo, A., Leister, E., Tassiopoulos, K., Allison, S., Malee, K., Paul, M.E., Smith, R., Van Dyke, R.B., Seage, G.R., III, & Mellins, C.A. 2014. Relationship between viral load and self-report measures of medication adherence among youth with perinatal HIV infection. *AIDS Care*, 26, (1) 107-115 available from: PM:23800360

von Wyl., V, Klimkait, T., Yerly, S., Nicca, D., Furrer, H., Cavassini, M., Calmy, A., Bernasconi, E., Boni, J., Aubert, V., Gunthard, H.F., Bucher, H.C., & Glass, T.R. 2013. Adherence as a predictor of the development of class-specific resistance mutations: the Swiss HIV Cohort Study. *PLoS.One.*, 8, (10) e77691 available from: PM:24147057

Johannessen, A., Naman, E., Kivuyo, S.L., Kasubi, M.J., Holberg-Petersen, M., Matee, M.I., Gundersen, S.G., & Bruun, J.N. 2009. Virological efficacy and emergence of drug resistance in adults on antiretroviral treatment in rural Tanzania. *BMC.Infect.Dis.*, 9, 108 available from: PM:19583845

Chi, B.H., Cantrell, R.A., Zulu, I., Mulenga, L.B., Levy, J.W., Tambatamba, B.C., Reid, S., Mwango, A., Mwinga, A., Bulterys, M., Saag, M.S., & Stringer, J.S. 2009. Adherence to first-line antiretroviral therapy affects non-virologic outcomes among patients on treatment for more than 12 months in Lusaka, Zambia. *Int.J.Epidemiol.*, 38, (3) 746-756 available from: PM:19223334

WHO Resistance Surveillance Report 2012(WHO 2012)

Cozzi-Lepri, A., UK HIV Drug Resistance, & UK CHIC 2010. Long-term probability of detecting drug-resistant HIV in treatment-naive patients initiating combination antiretroviral therapy. *Clin.Infect.Dis.*, 50, (9) 1275-1285 available from: PM:20353366

Cheeseman, S.H., Hattox, S.E., McLaughlin, M.M., Koup, R.A., Andrews, C., Bova, C.A., Pav, J.W., Roy, T., Sullivan, J.L., & Keirns, J.J. 1993. Pharmacokinetics of nevirapine: initial single-rising-dose study in humans. *Antimicrob.Agents Chemother.*, 37, (2) 178-182 available from: PM:8452345

Bangsberg DR, Moss AR, Deeks SG et al. Paradoxes of adherence and drug resistance to HIV antiretroviral therapy. J Antimicrob Chem 2004; 53 (5): 696-699.

Bangsberg, D.R. 2006a. Less than 95% adherence to nonnucleoside reverse-transcriptase inhibitor therapy can lead to viral suppression. *Clin.Infect.Dis.*, 43, (7) 939-941 available from: PM:16941380

Gardner, E.M., Burman, W.J., Steiner, J.F., Anderson, P.L., & Bangsberg, D.R. 2009. Antiretroviral medication adherence and the development of class-specific antiretroviral resistance. *AIDS*, 23, (9) 1035-1046 available from: PM:19381075

Gross, R., Bilker, W.B., Wang, H., & Chapman, J. 2008. How long is the window of opportunity between adherence failure and virologic failure on efavirenz-based HAART? *HIV.Clin.Trials*, 9, (3) 202-206 available from: PM:18547907

Parienti, J.J., Massari, V., Reliquet, V., Chaillot, F., Le, M.G., Arvieux, C., Vabret, A., & Verdon, R. 2007. Effect of twice-daily nevirapine on adherence in HIV-1-infected patients: a randomized controlled study. *AIDS*, 21, (16) 2217-2222 available from: PM:18090049

Hill, A., McBride, A., Sawyer, A.W., Clumeck, N., & Gupta, R.K. 2013. Resistance at virological failure using boosted protease inhibitors versus nonnucleoside reverse transcriptase inhibitors as first-line antiretroviral therapy--implications for sustained efficacy of ART in resource-limited settings. *J.Infect.Dis.*, 207 Suppl 2, S78-S84 available from: PM:23687293

Orrell, C., Harling, G., Lawn, S.D., Kaplan, R., McNally, M., Bekker, L.G., & Wood, R. 2007. Conservation of first-line antiretroviral treatment regimen where therapeutic options are limited. *Antivir.Ther.*, 12, (1) 83-88 available from: PM:17503751

Hoffmann CJ, Charalambous S, Sim J, et al. Viremia, Resuppression, and Time to Resistance in Human Immunodeficiency Virus (HIV) Subtype C during First-Line Antiretroviral Therapy in South Africa. Clin Infect Dis 2009; 49:1928–35.

Hoffmann CJ, Charalambous S, Grant AD, Morris L, Churchyard GJ, Chaisson RE. Durable HIV RNA resuppression after virologic failure while remaining on a first-line regimen: a cohort study. Trop Med & Int Health 2013

Bärnighausen T, Chaiyachati K, Chimbindi N, et al. Interventions to increase antiretroviral adherence in sub-Saharan Africa: a systematic review of evaluation studies. Lancet Infect Dis 2011; 11: 942–51.

Rutstein SE, Hosseinipour MC, Kamwendo D, Soko A, Mkandawire M, Biddle AK, et al. (2015) Dried Blood Spots for Viral Load Monitoring in Malawi: Feasible and Effective. PLoS ONE 10(4): e0124748. doi:10.1371/journal.pone.0124748

Bonner, K., Mezochow, A., Roberts, T., Ford, N., & Cohn, J. 2013. Viral load monitoring as a tool to reinforce adherence: a systematic review. *J.Acquir.Immune.Defic.Syndr.*, 64, (1) 74-78 available from: PM:23774877

Kranzer, K. & Ford, N. 2011. Unstructured treatment interruption of antiretroviral therapy in clinical practice: a systematic review. *Trop.Med.Int.Health*, 16, (10) 1297-1313 available from: PM:21718394

Kranzer, K., Lewis, J.J., Ford, N., Zeinecker, J., Orrell, C., Lawn, S.D., Bekker, L.G., & Wood, R. 2010. Treatment interruption in a primary care antiretroviral therapy program in South Africa: cohort analysis of trends and risk factors. *J.Acquir.Immune.Defic.Syndr.*, 55, (3) e17-e23 available from: PM:20827216

Tassie, J.M., Baijal, P., Vitoria, M.A., Alisalad, A., Crowley, S.P., & Souteyrand, Y. 2010. Trends in retention on antiretroviral therapy in national programs in low-income and middle-income countries. *J.Acquir.Immune.Defic.Syndr.*, 54, (4) 437-441 available from: PM:20351559

Wandeler, G., Keiser, O., Pfeiffer, K., Pestilli, S., Fritz, C., Labhardt, N.D., Mbofana, F., Mudyiradima, R., Emmel, J., Egger, M., & Ehmer, J. 2012. Outcomes of antiretroviral treatment programs in rural Southern Africa. *J.Acquir.Immune.Defic.Syndr.*, 59, (2) e9-16 available from: PM:22067665

Wallis, C.L., Mellors, J.W., Venter, W.D., Sanne, I., & Stevens, W. 2010. Varied patterns of HIV-1 drug resistance on failing first-line antiretroviral therapy in South Africa. *J.Acquir.Immune.Defic.Syndr.*, 53, (4) 480-484 available from: PM:19801944

McMahon, J.H., Elliott, J.H., Bertagnolio, S., Kubiak, R., & Jordan, M.R. 2013. Viral suppression after 12 months of antiretroviral therapy in low- and middle-income countries: a systematic review. *Bull.World Health Organ*, 91, (5) 377-385E available from: PM:23678201

Charurat, M., Oyegunle, M., Benjamin, R., Habib, A., Eze, E., Ele, P., Ibanga, I., Ajayi, S., Eng, M., Mondal, P., Gebi, U., Iwu, E., Etiebet, M.A., Abimiku, A., Dakum, P., Farley, J., & Blattner, W. 2010. Patient retention and adherence to antiretrovirals in a large antiretroviral therapy program in Nigeria: a longitudinal analysis for risk factors. *PLoS.One.*, 5, (5) e10584 available from: PM:20485670

Fox MP et al. Rates and Predictors of Failure of First-line Antiretroviral Therapy and Switch to Second-line ART in South Africa JAIDS 2012; 60:428–437

DeGruttola, V., Dix, L., D'Aquila, R., Holder, D., Phillips, A., Ait-Khaled, M., Baxter, J., Clevenbergh, P., Hammer, S., Harrigan, R., Katzenstein, D., Lanier, R., Miller, M., Para, M., Yerly, S., Zolopa, A., Murray, J., Patick, A., Miller, V., Castillo, S., Pedneault, L., & Mellors, J. 2000. The relation between baseline HIV drug resistance and response to antiretroviral therapy: re-analysis of retrospective and prospective studies using a standardized data analysis plan. *Antivir.Ther.*, 5, (1) 41-48 available from: PM:10846592

Grinsztejn, B., Nguyen, B.Y., Katlama, C., Gatell, J.M., Lazzarin, A., Vittecoq, D., Gonzalez, C.J., Chen, J., Harvey, C.M., & Isaacs, R.D. 2007. Safety and efficacy of the HIV-1 integrase inhibitor raltegravir (MK-0518) in treatment-experienced patients with multidrug-resistant virus: a phase II randomised controlled trial. *Lancet*, 369, (9569) 1261-1269 available from: PM:17434401

Rosenbloom, D.I., Hill, A.L., Rabi, S.A., Siliciano, R.F., & Nowak, M.A. 2012. Antiretroviral dynamics determines HIV evolution and predicts therapy outcome. *Nat.Med.*, 18, (9) 1378-1385 available from: PM:22941277

Cambiano, V., Lampe, F.C., Rodger, A.J., Smith, C.J., Geretti, A.M., Lodwick, R.K., Holloway, J., Johnson, M., & Phillips, A.N. 2010b. Use of a prescription-based measure of antiretroviral therapy adherence to predict viral rebound in HIV-infected individuals with viral suppression. *HIV.Med.*, 11, (3) 216-224 available from: PM:20002781

Arnsten, J.H., Demas, P.A., Farzadegan, H., Grant, R.W., Gourevitch, M.N., Chang, C.J., Buono, D., Eckholdt, H., Howard, A.A., & Schoenbaum, E.E. 2001. Antiretroviral therapy adherence and viral suppression in HIV-infected drug users: comparison of self-report and electronic monitoring. *Clin.Infect.Dis.*, 33, (8) 1417-1423 available from: PM:11550118

Montaner, J.S., Reiss, P., Cooper, D., Vella, S., Harris, M., Conway, B., Wainberg, M.A., Smith, D., Robinson, P., Hall, D., Myers, M., & Lange, J.M. 1998. A randomized, double-blind trial comparing combinations of nevirapine, didanosine, and zidovudine for HIV-infected patients: the INCAS Trial. Italy, The Netherlands, Canada and Australia Study. *JAMA*, 279, (12) 930-937 available from: PM:9544767

Eron, J.J., Benoit, S.L., Jemsek, J., MacArthur, R.D., Santana, J., Quinn, J.B., Kuritzkes, D.R., Fallon, M.A., & Rubin, M. 1995. Treatment with lamivudine, zidovudine, or both in HIV-positive patients with 200 to 500 CD4+ cells per cubic millimeter. North American HIV Working Party. *N.Engl.J.Med.*, 333, (25) 1662-1669 available from: PM:7477218

Havlir, D., McLaughlin, M.M., & Richman, D.D. 1995. A pilot study to evaluate the development of resistance to nevirapine in asymptomatic human immunodeficiency virus-infected patients with CD4 cell counts of > 500/mm3: AIDS Clinical Trials Group Protocol 208. *J.Infect.Dis.*, 172, (5) 1379-1383 available from: PM:7594683

Kuritzkes, D.R., Quinn, J.B., Benoit, S.L., Shugarts, D.L., Griffin, A., Bakhtiari, M., Poticha, D., Eron, J.J., Fallon, M.A., & Rubin, M. 1996. Drug resistance and virologic response in NUCA 3001, a randomized trial of lamivudine (3TC) versus zidovudine (ZDV) versus ZDV plus 3TC in previously untreated patients. *AIDS*, 10, (9) 975-981 available from: PM:8853730

Larder, B.A. 1995. Viral resistance and the selection of antiretroviral combinations. *J.Acquir.Immune.Defic.Syndr.Hum.Retrovirol.*, 10 Suppl 1, S28-S33 available from: PM:8595505

Doyle, T. & Geretti, A.M. 2012. Low-level viraemia on HAART: significance and management. *Curr.Opin.Infect.Dis.*, 25, (1) 17-25 available from: PM:22156900

Phillips, A.N., Eron, J., Bartlett, J., Kuritzkes, D.R., Johnson, V.A., Gilbert, C., Johnson, J., Keller, A., & Hill, A.M. 1997. Correspondence between the effect of zidovudine plus lamivudine on plasma HIV level/CD4 lymphocyte count and the incidence of clinical disease in infected individuals. North American Lamivudine HIV Working Group. *AIDS*, 11, (2) 169-175 available from: PM:9030363

Wittkop, L., Gunthard, H.F., de, W.F., Dunn, D., Cozzi-Lepri, A., De, L.A., Kucherer, C., Obel, N., von, W., V, Masquelier, B., Stephan, C., Torti, C., Antinori, A., Garcia, F., Judd, A., Porter, K., Thiebaut, R., Castro, H., van Sighem, A.I., Colin, C., Kjaer, J., Lundgren, J.D., Paredes, R., Pozniak, A., Clotet, B., Phillips, A., Pillay, D., & Chene, G. 2011. Effect of transmitted drug resistance on virological and immunological response to initial combination antiretroviral therapy for HIV (EuroCoord-CHAIN joint project): a European multicohort study. *Lancet Infect.Dis.*, 11, (5) 363-371 available from: PM:21354861

Wittkop, L., Bitard, J., Lazaro, E., Neau, D., Bonnet, F., Mercie, P., Dupon, M., Hessamfar, M., Ventura, M., Malvy, D., Dabis, F., Pellegrin, J.L., Moreau, J.F., Thiebaut, R., & Pellegrin, I. 2013. Effect of cytomegalovirus-induced immune response, self antigen-induced immune response, and microbial translocation on chronic immune activation in successfully treated HIV type 1-infected patients: the ANRS CO3 Aquitaine Cohort. *J.Infect.Dis.*, 207, (4) 622-627 available from: PM:23204178

Gallant, J.E., Staszewski, S., Pozniak, A.L., DeJesus, E., Suleiman, J.M., Miller, M.D., Coakley, D.F., Lu, B., Toole, J.J., & Cheng, A.K. 2004. Efficacy and safety of tenofovir DF vs stavudine in combination therapy in antiretroviral-naive patients: a 3-year randomized trial. *JAMA*, 292, (2) 191-201 available from: PM:15249568

Harrigan PR, Hogg RS, Dong WWY, et al. Predictors of HIV drug resistance mutations in a large antiretroviral naïve cohort initiating triple antiretroviral therapy. J Infect Dis 2005; 191: 339:347.

Ledergerber, B., Egger, M., Opravil, M., Telenti, A., Hirschel, B., Battegay, M., Vernazza, P., Sudre, P., Flepp, M., Furrer, H., Francioli, P., & Weber, R. 1999. Clinical progression and virological failure on highly active antiretroviral therapy in HIV-1 patients: a prospective cohort study. Swiss HIV Cohort Study. *Lancet*, 353, (9156) 863-868 available from: PM:10093977

Phillips, A.N., Staszewski, S., Weber, R., Kirk, O., Francioli, P., Miller, V., Vernazza, P., Lundgren, J.D., & Ledergerber, B. 2001. HIV viral load response to antiretroviral therapy according to the baseline CD4 cell count and viral load. *JAMA*, 286, (20) 2560-2567 available from: PM:11722270

Phillips, A.N., Dunn, D., Sabin, C., Pozniak, A., Matthias, R., Geretti, A.M., Clarke, J., Churchill, D., Williams, I., Hill, T., Green, H., Porter, K., Scullard, G., Johnson, M., Easterbrook, P., Gilson, R., Fisher, M., Loveday, C., Gazzard, B., & Pillay, D. 2005. Long term probability of detection of HIV-1 drug resistance after starting antiretroviral therapy in routine clinical practice. *AIDS*, 19, (5) 487-494 available from: PM:15764854

Staszewski, S., Miller, V., Sabin, C., Carlebach, A., Berger, A.M., Weidmann, E., Helm, E.B., Hill, A., & Phillips, A. 1999a. Virological response to protease inhibitor therapy in an HIV clinic cohort. *AIDS*, 13, (3) 367-373 available from: PM:10199227

Staszewski, S., Miller, V., Sabin, C., Schlecht, C., Gute, P., Stamm, S., Leder, T., Berger, A., Weidemann, E., Hill, A., & Phillips, A. 1999b. Determinants of sustainable CD4 lymphocyte count increases in response to antiretroviral therapy. *AIDS*, 13, (8) 951-956 available from: PM:10371176

Van Leth, L.F., Phanuphak, P., Ruxrungtham, K., Baraldi, E., Miller, S., Gazzard, B., Cahn, P., Lalloo, U.G., van der Westhuizen, I.P., Malan, D.R., Johnson, M.A., Santos, B.R., Mulcahy, F., Wood, R., Levi, G.C., Reboredo, G., Squires, K., Cassetti, I., Petit, D., Raffi, F., Katlama, C., Murphy, R.L., Horban, A., Dam, J.P., Hassink, E., van, L.R., Robinson, P., Wit, F.W., & Lange, J.M. 2004. Comparison of first-line antiretroviral therapy with regimens including nevirapine, efavirenz, or both drugs, plus stavudine and lamivudine: a randomised open-label trial, the 2NN Study. *Lancet*, 363, (9417) 1253-1263 available from: PM:15094269

Bangsberg, D.R., Acosta, E.P., Gupta, R., Guzman, D., Riley, E.D., Harrigan, P.R., Parkin, N., & Deeks, S.G. 2006b. Adherence-resistance relationships for protease and non-nucleoside reverse transcriptase inhibitors explained by virological fitness. AIDS, 20, (2) 223-231 available from: PM:16511415

Gross, R., Bilker, W.B., Friedman, H.M., & Strom, B.L. 2001. Effect of adherence to newly initiated antiretroviral therapy on plasma viral load. *AIDS*, 15, (16) 2109-2117 available from: PM:11684930

Ledergerber, B., Lundgren, J.D., Walker, A.S., Sabin, C., Justice, A., Reiss, P., Mussini, C., Wit, F., d'Arminio, M.A., Weber, R., Fusco, G., Staszewski, S., Law, M., Hogg, R., Lampe, F., Gill, M.J., Castelli, F., & Phillips, A.N. 2004. Predictors of trend in CD4-positive T-cell count and mortality among HIV-1-infected individuals with virological failure to all three antiretroviral-drug classes. *Lancet*, 364, (9428) 51-62 available from: PM:15234856

d'Arminio Monforte A, Cozzi Lepri A, Phillips AN, et al. Interruption of HAART in HIV clinical practice. Results from the ICONA study. JAIDS 2005; 38: 407-416.

Li X, Margolick JB, Conover CS, et al. Interruption and discontinuation of HART in the MACS. JAIDS 2005; 38: 3:320-328.

Mocroft A, Youle M, Moore A, et al. Reasons for modification and discontinuation of antiretrovirals: results from a single treatment centre. AIDS 2001; 15 (2): 185-194.

Wit FWNM, Blanckenberg DH, Brinkman K, et al. Safety of long-term interruption of successful antiretroviral therapy: the ATHENA cohort study. AIDS 2005; 19: 345-348.

UK HIV Drug Resistance Database and UK CHIC. Long term probability of detection of HIV-1 drug resistance after starting antiretroviral therapy in routine clinical practice. AIDS 2005; 19 (5): 487-494.

Harrigan, P.R., Hogg, R.S., Dong, W.W., Yip, B., Wynhoven, B., Woodward, J., Brumme, C.J., Brumme, Z.L., Mo, T., Alexander, C.S., & Montaner, J.S. 2005. Predictors of HIV drug-resistance mutations in a large antiretroviral-naive cohort initiating triple antiretroviral therapy. *J.Infect.Dis.*, 191, (3) 339-347 available from: PM:15633092

Sigaloff K, et a. Accumulation of HIV Drug Resistance Mutations in Patients Failing First-Line Antiretroviral Treatment in South Africa. AIDS Res Hum Retr 2012; 28:171-175.

Fox Z, Phillips AN, Cohen C, et al. Viral resuppression and detection of drug resistance following interruption of a suppressive non-nucleoside reverse transcriptase inhibitor-based regimen. AIDS 2008; 22:2279-2289.

Devereux HL, Youle M, Johnson MA, et al Rapid decline in detectability of HIV-1 drug resistance mutations after stopping therapy. AIDS  1999; 13:F123-F127.

Devereux HL, Emery VC, Johnson MA, et al. Replicative fitness in vivo of HIV-1 variants with multiple drug resistance associated mutations. J Med Virol 2001:; 65:218-224.

Deeks SG, Grant RM, Wrin T, et al. Persistence of drug-resistant HIV-1 after a structured treatment interruption and its impact on treatment response. AIDS 2003; 17:361-370.

Birk M, Svedhem V, Sonnerborg A. Kinetics of HIV-1 RNA and resistance-associated mutations after cessation of antiretroviral combination therapy. AIDS 2001; 15:1359-1368.

Walter H, Low P, Harrer T, et al. No evidence for persistence of multidrug resistant viral strains after a 7-month treatment interruption in an HIV-1 infected individual. JAIDS 2002; 31:137-146.

Hance AJ, Lemiale V, Izopet J, et al. Changes in HIV-1 populations after treatment interruption in patients failing antiretroviral therapy. J Virol 2001; 75:6410-6417.

Tarwater PM, Parish M, Gallant JE. Prolonged treatment interruption after immunologic response to HAART. Clin Infect Dis 2003; 37:1541-1548.

Phillips A, CASCADE Collaboration. Short-term risk of AIDS according to current CD4 cell count and viral load in antiretroviral drug-naïve individuals and those treated in the monotherapy era. *AIDS*, 2004. 18(1):51-8.

Brambilla D et al. The contribution of assay variation and biological variation to the total variability of plasma HIV-1 RNA measurements. AIDS 1999;13(16):2269-79.

Raboud JM, Montaner JSG, Conway B, Haley L, Sherlock C, O’Shaughnessy MV, Schechter MT. Variation in plasma RNA levels, CD4 cell counts and p24 antigen levels in clinically stable men with human immunodeficiency virus infection. J Infect Dis 1996;174:191-4

Andreotti M et al. Correlation between HIV-1 viral load quantification in plasma, dried blood spots, and dried plasma spots using the Roche COBAS Taqman assay. J Clin Virol. 2010;47:4–7.

Arredondo M et al. Comparison of HIV-1 RNA measurements using plasma and dried blood spots (DBS) in the Automated Abbott Real Time Viral Load Assay. J Clin Microbiol. 2011 (published ahead of printing).

Marconi A et al. Evaluation of the Abbott Real-Time HIV-1 quantitative assay with dried blood spot speci­mens. Clin Microbiol Infect*.* 2009;15:93–7.

Pirillo MF-Pinson P et al. Quantification of HIV-RNA from dried blood spots using Siemens VERSANT® HIV-1 RNA (kPCR) assay. J Antimicrob Chemother. 2011;66:2823–6.

Fajardo E, Metcalf C, Chaillet P, Aleixo L, Pannus P, Panunzi I, et al. Prospective Evaluation of Diagnostic Accuracy of Dried Blood Spots from Finger Prick Samples for Determination of HIV-1 Load with the NucliSENS Easy-Q HIV-1 Version 2.0 Assay in Malawi. 2014

Smit PW, Sollis KA, Fiscus S, Ford N, Vitoria M, et al. (2014) Systematic Review of the Use of Dried Blood Spots for Monitoring HIV Viral Load and for Early Infant Diagnosis. PLoS ONE 9(3): e86461. doi:10.1371/journal.pone.0086461

Ondoa P, Shamu T, Bronze M, Wellington M, Sonia Boender T, Manting C, et al. Performance and Logistical Challenges of Alternative HIV-1 Virological Monitoring Options in a Clinical Setting of Harare, Zimbabwe. BioMed Research International Volume 2014. http://dx.doi.org/10.1155/2014/102598

Mavedzenge SN, Davey C, Chirenje T, Mushati P, Mtetwa S, Dirawo J, et al. (2015) Finger Prick Dried Blood Spots for HIV Viral Load Measurement in Field Conditions in Zimbabwe. PloS ONE 10(5): e0126878. doi:10.1371/journal.pone.0126878

World Health Organisation. Technical and operational considerations for implementing HIV viral load testing. Access to HIV diagnostics. July 2014. [www.who.int](http://www.who.int)

Stinnett, A.A., Mullahy, J., Net health benefits: a new framework for the analysis of uncertainty in cost-effectiveness analysis, Medical Decision Making, 1998; 18:2, S68-80.

Drummond, M., et al., Methods for the Economic Evaluation of Health Care Programmes. 3rd Edn. 2005: Oxford Medical Publications.

Woods E, Revill P, Sculpher M, Claxton K. Country-Level Cost- Effectiveness Thresholds: Initial Estimates and the Need for Further Research, Centre for Health Economics Research Paper 109, University of York. 2015.

Eaton J et al. Health benefits, costs, and cost-effectiveness of earlier eligibility for adult antiretroviral therapy and expanded treatment coverage: a combined analysis of 12 mathematical models. Lancet Global Health 2014: E23-E34

Hyle, E. P., Jani, I. V, Lehe, J., Su, A. E., Wood, R., Quevedo, J., … Walensky, R. P. (2014). The Clinical and Economic Impact of Point-of-Care CD4 Testing in Mozambique and Other Resource-Limited Settings: A Cost-Effectiveness Analysis. PLoS Med, 11(9), e1001725. doi:10.1371/journal.pmed.1001725.

Keebler D, Revill P, et al. How Should HIV Programmes Monitor Adults on ART? A Combined Analysis of Three Mathematical Models. Lancet Global Health 2014. E35-E43.

Salomon JA, Vos T, Hogan DR, et al. Common values in assessing health outcomes from disease and injury: disability weights measurement study for the Global Burden of Disease Study 2010. Lancet 2012; 380: 2129–43.
