# Supplementary material for: 90‐90‐90 by 2020? Estimation and projection of the adult HIV epidemic and ART programme in Zimbabwe – 2017 to 2020
Source: J Int AIDS Soc. 2018 Nov 22;21(11):e25205. doi: 10.1002/jia2.25205 (PMC6250855; doi:10.1002/jia2.25205)
Supplement: Supplementary file 2 — Data S2. Description of model calibration. [file JIA2-21-e25205-s002.docx]

**Description of Model Calibration**

This document explains the process undertaken to calibrate the HIV Synthesis model to country data from Zimbabwe. The model is a detailed individual-based model including sexual behaviour, HIV transmission, HIV progression and effects of ART. Being “individual-based” means that each time the model (in the form of a computer program) is run it generates a data set of the simulated lifetime adult experience of a representative subset of the population of a given country or region. In each three- month period, data for each simulated person in the dataset on variables such as age and condomless sex, HIV testing, and, if infected with HIV, on HIV diagnosis, CD4 count, viral load, use of specific ART drugs, adherence, resistance are updated.

The data items used for calibration are extracted from DHS (2006, 2011, 2015) (1-3), ZIMPHIA surveys (2016) (4), Zimbabwe Ministry of Health Report in 2014 (5), a respondent driven sampling survey amongst female sex workers (FSW) (2013) (6) (number of FSW is deduced from data provided by Cowan et al (2017) (8)), the WHO Resistance Report in 2012 (9) and the GARPCR 2016 (number on ART were confirmed by the MoH (10). The extracted data point values are shown below and on the graphs below describing the model calibration.

The process of calibration is to find values for model parameters that result in a model that is able to mimic the real life epidemic and ART programme.

The approach to model calibration uses Approximate Bayesian Computation (11). This involves sampling model parameter values from various possible values, represented by prior distributions, then running the model and assessing how closely the outputs concur with observed data.

Once we have a number of sets of parameter values that result in model outputs that are close to observed data then these sets are used when running the model to make projections and consider the effects of potential interventions.

The model contains many parameters but most of these are held fixed, and hence form part of the model structure. The parameters for which we sample from prior distributions are described below. Most of these parameter values are not directly informed by observed data, rather it is the above process of comparing model outputs with observed data that allows the model parameters to be informed by data and the uncertainty in their values can be propagated into the uncertainty expressed around final model outputs.

**Prior distributions for parameters**

|  | **Description** | **Distribution (prior)** | **Median (90% range)** |
| --- | --- | --- | --- |
| ***Parameters relating to sexual behaviour*** | |  |  |
| *Swn* | Value of multiplicative factor determining numbers of partners for those in highest new partner group | Uniform (4, 25) | 14.5 (5.07, 23.9) |
| *Eprate* | Rate of new long term partners in youngest age group (15-24) | Log normal (ln 0.10, 0.25) | 0.10 (0.07, 0.15) |
| *Highsa* | Multiplicative factor determining numbers of partners for those in second highest new partner group | Uniform (3, 10) | 6.49 (3.34, 9.65) |
| *p_rred_p* | Proportion of the population in whom the sexual risk behaviour is very low | Uniform (0.1, 0.5) | 0.30 (0.12, 0.48) |
| *p_hsb_p* | Proportion of the population in whom the sexual risk behaviour has a tendency to be higher than average | Uniform (0, 0.05) | 0.02 (0.002, 0.05) |
| *newp_factor* | Overall average level of sexual risk behaviour. The correlation with the above parameters induced by the sampling of this parameter is to provide a focus on parameter space most likely to give low values of the overall fit. For example, if the sampling of swn and highsa gives values at the high end of the distribution and sampling of p_rred_p produces a value at the low end then the model simulation run will produce an epidemic which is too large, unless there is some compensation when selecting the value of this parameter. | 3 x (6.5/highsa)x(14.5/swn) x (p_rred_p/0.3) x (0.025/p_hsb_p) x exp(Normal(0, 0.5^2^) | 5.03 (0.87, 64.3) |
| *P_ccufsw_base* | Proportion of women aged 15 to 29 who become sex workers who consistently use condoms per 3 months. | Uniform (0, 0.002) | 0.001 (0.0001, 0.002) |
| *conc_ep* | Degree to which those with a long term condomless sex partner have a lower or higher probability of short term condomless sex partners than those without a long term condomless sex partner. | Lognormal (0,0.6) | 0.99 (0.37, 2.66) |
| *ych_risk_beh_newp* | Degree of reduction in condomless sex with short term partners per year from 1995 – 2000 | Uniform (0,0.20) | 0.10 (0.01, 0.19) |
| *ych_risk_beh_ep* | Degree of reduction in condomless sex per year with long term partners from 1995-2000 | Uniform (0,0.20) | 0.10 (0.01, 0.19) |
| *ch_risk_diag_newp* | Degree of reduction in condomless sex with short term partners in a person diagnosed with HIV | Beta (12, 2) | 0.87 (0.69, 0.97) |
| *ch_risk_diag* | Degree of reduction in condomless sex with long term partner in a person diagnosed with HIV | Beta (12, 2) | 0.87 (0.69, 0.97) |
| *ych2_risk_beh_newp* | Degree of change in condomless sex with short term partners per year from 2010 – 2015 | Uniform(-0.04,0.04) | 0.0002 (-0.036, 0.036) |
| **Parameters relating to transmission** | |  |  |
| *fold_tr* | Multiplicative factor determining the uncertainty in transmission rate in different viral load groups | Lognormal (ln 1, 0.3) | 1 (0.61, 1.64) |
| *tr_rate_undetec_vl* | Multiplicative factor to determine uncertainty in transmission rate when VL undetectable | Lognormal (ln 0.001, 1.5) | 0.001 (0.00008, 0.003) |
| *Fold_change_w* | Multiplicative factor accounting for higher rate of acquisition for women compared to men | Lognormal (ln 1.5, 0.3) | 1.50 (0.92, 2.46) |
| *fold_change_sti* | Multiplicative factor accounting for higher rate of acquisition in people with STI | Lognormal (ln 3.0, 0.3) | 3.00 (1.84, 4.91) |
| *res_trans_factor~* | parameter determining the probability that if resistance mutation present in source partner that this is not present/detectable in virus new host | 0.50: 33%  0.75: 33%  1.00: 33% |  |
| rate_loss_persistence | loss of persistence of transmitted mutations from majority virus to minority virus per 3 months | Uniform (0.005, 0.02) | 0.013 (0.006, 0.02) |
| **Parameters relating to HIV testing** | |  |  |
| *an_lin_incr_test* | increase in age and gender specific HIV testing rate per 3 month during the linear increase, i.e. when quadratic increase ends in 2012 | Uniform (0, 0.03) | 0.01 (0.002, 0.03) |
| base_test_incr | Rate of increase in baseline HIV testing | Lognormal (0, 0.6) | 1.00 (0.37, 2.67) |
| *rate_testanc_inc* | Factor multiplied by the square of the years since the introduction of testing in ANC to obtain the probability of attending ANC for pregnant women (this happens until the end of 2016) | Beta (3, 900) | 0.003 (0.0009, 0.007) |
| *incr_test_rate_sympt* | Relative increase each 3 months in the probability of a person with a WHO stage 3 or 4 disease to be tested for HIV | Uniform (1.05, 1.075) | 1.06 (1.05, 1.07) |
| *p_hard_reach_m* | Proportion of the male population considered hard to reach and that cannot be tested unless they develop symptoms | Uniform (0.25, 0.45) | 0.35 (0.26, 0.40) |
| *p_hard_reach_w* | Proportion of the female population considered hard to reach and that cannot be tested unless they develop symptoms | Uniform (0.15, 0.35) | 0.25 (0.16, 0.34) |
| **Parameters relating to pre-ART care and progression of HIV** | | |  |
| *fx* | Multiplicative factor to alter the average rate of CD4 count decline in natural HIV progression (which thus alters the incubation period distribution). | Lognormal (ln 1.0, 0.2) | 1.00 (0.72, 1.39) |
| *prob_loss_at_diag* | Probability that a person is immediately lost after HIV diagnosis. | Beta (2, 14) | 0.11 (0.02, 0.28) |
| *prob_lossdiag_adctb* | Probability that a person with AIDS or acute TB is immediately lost after HIV diagnosis. | Beta (5,95) | 0.05 (0.02, 0.09) |
| *prob_lossdiag_who3e* | Probability that a person with WHO stage 3 condition is immediately lost after HIV diagnosis. | Beta (15, 85) | 0.15 (0.10, 0.21) |
| fold_change_ac_death_rate | Fold change in all cause death rate compared to base rate | Lognormal (ln2.0, 0.25) | 2.00 (1.32, 3.01) |
| *rate_lost* | For people under care yet to start ART or previously have taken ART, the rate of being lost to care per 3 months. | Lognormal (ln 0.02, 0.4) | 0.02 (0.01, 0.04) |
| *rate_return* | Probability of return to care for a person who has been diagnosed with HIV (and may have started ART) but is now lost and not on ART, without current WHO stage 3 or 4 disease, per 3 months. | Lognormal (ln 0.15, 0.5) | 0.15 (0.07, 0.34) |
| *prob_return_adc* | Probability of return to care for a person who has been diagnosed with HIV (and may have started ART) but is now lost and not on ART and has a WHO stage 4 condition. This is a probability that operates just for the 3 month period that the events occurs. | Beta (12,8) | 0.60 (0.42, 0.77) |
| **Parameters relating to people on ART** | |  |  |
| *adh_pattern* | Population adherence profile; described in terms of the proportion having a given average adherence and period-to-period variability in adherence. | Three alternative population adherence profiles, 50% probability of falling into either one |  |
| red_adh_tb_adc | Factor subtracted from the adherence level in 3 month periods with acute TB or when AIDS develops | Lognormal (ln 0.1, 0.5) | 0.10 (0.04, 0.23) |
| red_adh_tox_pop | Amount by which toxicity is assumed to affect adherence (it is assumed that this is the case in 30% of periods in which toxicity is present | Lognormal (ln 0.05, 0.5) | 0.05 (0.02, 0.11) |
| add_eff_adh_nnrti | Factor added to the adherence level in people on NNRTI | Lognormal (ln 0.10, 0.30) | 0.10 (0.06, 0.16) |
| an_incr_pr_art_init | Factor multiplied by the square of the years since ART introduction to obtain the probability of ART initiation per 3 months (it applies to people diagnosed with HIV ART-naïve). It is truncated at 0.8 | Beta (1.2, 100) | 0.01 (0.0009, 0.03) |
| altered_adh_sec_line_pop | Altered adherence for those on second line | Lognormal(ln 0.05, 0.05) | 0.05 (-0.03, 0.13) |
| *prob_lost_art* | For a person who interrupts / stops ART the probability that they are simultaneously lost from care. | Beta (4, 4) | 0.50 (0.23, 0.77) |
| *rate_restart* | Rate of restart of ART for people who previously have been on ART and have returned to care, per 3 months. | Lognormal (ln 0.5, 0.5) | 0.50 (0.22, 1.14) |
| *rate_int_choice* | Rate of interruption / stopping of ART per 3 months. This is the base rate – actual rate at any 3 month period also influenced by current drug toxicity and underlying tendency to adhere. | Lognormal (ln 0.005, 0.5) | 0.01 (0.002, 0.01) |
| *incr_rate_int_low_adh* | Parameter indicating the extent to which people with a long term average adherence in the lowest group have a multiplicatively increased risk of ART interruption. | Uniform (1,2) | 1.50 (1.05, 1.95) |
| *pr_switch_line* | Probability of switch to second line per 3 months in a person who has fulfilled the failure criteria for first line failure. | Beta (2, 100) | 0.02 (0.004, 0.05) |
| *clinic_not_aw_int_frac* | If a person interrupts ART, the probability that this is not disclosed to the clinic and they are classified as being on ART | Beta (6, 4) | 0.61 (0.34, 0.83) |

**Calibration score**

Our approach was to derive a calibration score that conveys the mean deviance of the modelled outputs from the observed data over a range of data items. Each model run produces model outputs that can be compared with observed data and the calibration score calculated. **This is calculated as the weighted mean** for each data item component. The weights (indicated in the table below) are determined according to the perceived importance of a close calibration to the data item and on the judgement of the accuracy and robustness of the data source. Note that the score represents the average amount by which the model outputs differ proportionately from the observed data; e.g. a score of 0.25 means that on average the model outputs are 25% away from the observed data. The lower the calibration score the more closely the model outputs can re-produce observed data estimates. The calibration to each data item for any one year is given by:

|observed data – model output|/observed data

For example, the component describing the prevalence of HIV in males ages 15-49 is calculated as –

$$c\_prev1549m=(\left( \frac{\sqrt{\left( {o\_prev1549m}_{06}-prev1549m_{06} \right)^{2}}}{{o\_prev1549m}_{06}} \right)$$

$$+\left( \frac{\sqrt{\left( {o\_prev1549m}_{11}-prev1549m_{11} \right)^{2}}}{{o\_prev1549m}_{11}} \right) +\left( \frac{\sqrt{\left( {o\_prev1549m}_{15}-prev1549m_{15} \right)^{2}}}{{o\_prev1549m}_{15}} \right) +\left( \frac{\sqrt{\left( {o\_prev1549m}_{16}-prev1549m_{16} \right)^{2}}}{{o\_prev1549m}_{16}} \right) )/4$$

Where ${o\_prev1549m}_{06}$ refers to the observed HIV prevalence in 2006 in men aged 15 to 49, $prev1549m_{06}$ refers to the modelled HIV prevalence among men aged 15 to 49 in 2006 and so on for the prevalence in 2011, 2015, and 2016.

The weighted sum of these components together form the overall calibration score.

The observed data used for the calibration score is shown in Table 1 below.

Table 1: Observed data used for each component of the calibration score

| Data item | Source | Year | Observed value | Weight |
| --- | --- | --- | --- | --- |
| Prevalence of HIV | DHS 2005-6 (1) | 2006 | 0.145 | 1 |
| Men 15-49 | DHS 2010-11 (3) | 2011 | 0.123 |  |
|  | DHS 2015 (2) | 2015 | 0.105 |  |
|  | Zimphia (4) | 2016 | 0.112 |  |
| Prevalence of HIV | DHS 2005-6 (1) | 2006 | 0.211 | 1 |
| Women 15-49 | DHS 2010-11 (3) | 2011 | 0.177 |  |
|  | DHS 2015 (2) | 2015 | 0.167 |  |
|  | Zimphia (4) | 2016 | 0.166 |  |
| Prevalence of HIV | DHS 2005-6 (1) | 2006 | 0.042 | 0.5 |
| Men 15-24 | DHS 2010-11 (3) | 2011 | 0.036 |  |
|  | DHS 2015 (2) | 2015 | 0.029 |  |
| Prevalence of HIV | DHS 2005-6 (1) | 2006 | 0.110 | 0.5 |
| Women 15-24 | DHS 2010-11 (3) | 2011 | 0.073 |  |
|  | DHS 2015 (2) | 2015 | 0.067 |  |
| Prevalence of HIV in FSW | RDS 2013 (6) | 2013 | 0.575 | 0.5 |
| Women 18-64 |  |  |  |  |
| Incidence of HIV | Zimphia (4) | 2016 | 0.28 | 0.5 |
| Men 15-49 |  |  |  |  |
| Incidence of HIV | Zimphia (4) | 2016 | 0.67 | 0.5 |
| Women 15-49 |  |  |  |  |
| Number of HIV tests done per year^a^ | GARCPR 2016 (12) | 2007 | 579767 | 1 |
| All 15-49 |  | 2009 | 1108264 |  |
|  |  | 2012 | 2240344 |  |
|  |  | 2013 | 2274328 |  |
|  |  | 2014 | 1755179 |  |
|  |  | 2015 | 2201246 |  |
| PLHIV with known status | Zimphia (4) | 2016 | 0.697 | 1 |
| Men 15-64 |  |  |  |  |
| PLHIV with known status | Zimphia (4) | 2016 | 0.771 | 1 |
| Women 15-64 |  |  |  |  |
|  |  |  |  |  |
| Number receiving ART^a^ | GARCPR 2016 (12) | 2004 | 4857 | 1 |
| Men 15-64 | Tsitsi Apollo (10) | 2005 | 11305 |  |
|  |  | 2006 | 25184 |  |
|  |  | 2007 | 33235 |  |
|  |  | 2008 | 47210 |  |
|  |  | 2009 | 72571 |  |
|  |  | 2010 | 117261 |  |
|  |  | 2011 | 161627 |  |
|  |  | 2012 | 188595 |  |
|  |  | 2013 | 226291 |  |
|  |  | 2014 | 264614 |  |
|  |  | 2015 | 301650 |  |
| Number receiving ART^a^ | GARCPR 2016 (12) | 2004 | 6143 | 1 |
| Women 15-64 | Tsitsi Apollo (10) | 2005 | 14562 |  |
|  |  | 2006 | 28448 |  |
|  |  | 2007 | 56854 |  |
|  |  | 2008 | 87513 |  |
|  |  | 2009 | 127059 |  |
|  |  | 2010 | 213118 |  |
|  |  | 2011 | 283036 |  |
|  |  | 2012 | 330206 |  |
|  |  | 2013 | 392589 |  |
|  |  | 2014 | 468305 |  |
|  |  | 2015 | 516557 |  |
| Number on 2^nd^ line | MoH (5) | 2014 | 10580 | 0.5 |
| All 15-64 |  |  |  |  |
| Proportion with viral suppression of PLHIV | Zimphia (4) | 2016 | 0.525 | 1 |
| Men 15-64 |  |  |  |  |
| Proportion with viral suppression of PLHIV | Zimphia (4) | 2016 | 0.635 | 1 |
| Women 15-64 |  |  |  |  |
| Number of FSW^b^ | Cowan (8) | 2017 | 73270^c^ | 0.5 |
| Proportion with RT resistance | WHO Resistance (9) | 2008 | 0.021 | 0.33 |
| at ART initiation | Report 2012 | 2009 | 0.037 |  |
| All 15-64 |  | 2010 | 0.061 |  |
| Number of pregnant women | MoH (13) | 2011 | 412122 | 0.75 |
| 15-64 | WHO (14) | 2013 | 398474 |  |
|  | Mhangara (15) | 2016.5 | 433914 |  |
|  |  |  |  |  |

**^a^**A higher emphasis was given to the observed data in later years (2014 and 2015) as these are considered to be more reliable data sources than earlier ones and we considered it more important to fit closely to these data than those in earlier years. Hence the c-score components for data from 2014 and 2015 were multiplied by 5 before being added to the overall score.

**^b^**A higher penalty (a value of 5 is added on to the calibration score) is given if the modelled number of condomless sex FSW in 2016.25 falls below 27 or above 291, corresponding to 9,874 and 106,149 condomless sex FSW in Zimbabwe. The rationale for penalizing these simulations is as follows –

It has been estimated that the minimum and maximum percentage of women who are FSW in sub-Saharan Africa is between 0.4% and 4.3% respectively(16). These percentages were applied to the overall female population in Zimbabwe as stated in the Census 2012(17) (N=3,291,432 women aged 15-49), giving minimum and maximum estimates of 13,166 and 141,532 FSW in Zimbabwe. Of these, we derived the number of condomless FSW. An estimate of 0.59 (0.45, 0.73) is given in the Sapphire Trial Analysis (paper awaiting submission). This estimate refers to the proportion of women reporting condomless sex within the last month.

In the model, women are defined as condomless FSW if they have 3 or more condomless partners within a three month period in the last *year*. Given this, coupled with the general underreporting of condomless sex, and the upper end of the confidence interval provided in the SAPPH-IRe Trial, we felt 75% would be a more realistic estimate of the proportion of FSW having condomless sex with their clients. Hence 75% was applied to the estimates of the number of FSW, giving minimum and maximum estimates of the number of condomless FSW of 9,874 and 106,149 respectively.

^c^ **FSW population size**

Size estimation from Harare, Bulawayo, Shamva and Mazowe were used to estimate a national estimate of 44,586, 1.35% of all women aged 15-49, assuming Sisters Clinics cover 95% of the country (18).

In sensitivity analyses it is assumed that Sisters Clinics cover only 75% of the country. This results in a FSW population of **48,358**, 1.47% all women aged 15-49. This latter figure is used as the initial FSW population estimate.

Of high risk women recruited to DREAMS, 66% of women self-identified as FSW (AIDSImpact_outline_20171106, Slide 6). The remaining referred to as ‘young women selling sex, YWSS’. This implies 48,358 is equal to only 66% of the total sex worker population.

Therefore multiply initial FSW population by (1/0.66=1.51) to get **73270 FSW** in Zimbabwe.

**Aborting of model runs for which calibration to prevalence is inadequate**

Model runs were aborted in 1997 if the modelled value for HIV prevalence for women was below 0.15 or above 0.35 and in 2006 if the overall HIV prevalence was below 0.11 or above 0.25 as we considered these outside a plausible range, based on estimates from sentinel ANC sites(18).

**Results from calibration**

The aim of the calibration process was to find 500 parameter sets that yielded a calibration score of <0.30. Over 70,000 runs were performed in order to achieve this. Approximately 80% of runs were aborted due to the prevalence being outside of the above ranges in 1997 or 2006.

The aim of identifying these 500 parameter sets was to use them to replace the original random probability distributions for these model parameters when making future projections. Hence, for future projections, the model was run 1500 times, to again find runs that successfully met the prevalence criteria described above (i.e. runs aborted if the prevalence was perceived to be too low or too high) and had a calibration score of <0.3. To reduce stochastic variation, each time the model was run, we ensured that simulations for future years were repeated 10 times per run and the mean across these simulations was used when describing outputs.

**Comparison of model outputs with observed data**

Below we show the median and 90% range of model outputs from runs which are selected based on the calibration score being below 0.30. *Where available, modelled data are compared to observed data – of note, this may include observed data that were not used in the calibration score*. The median values across all runs of the modelled data are shown by the solid line in the graphs and are labelled ‘Model (median)’, whilst the 90% range of these runs are shown by the shaded area and are labelled ‘Model 90% range’.

**Model outputs included in the calibration score**


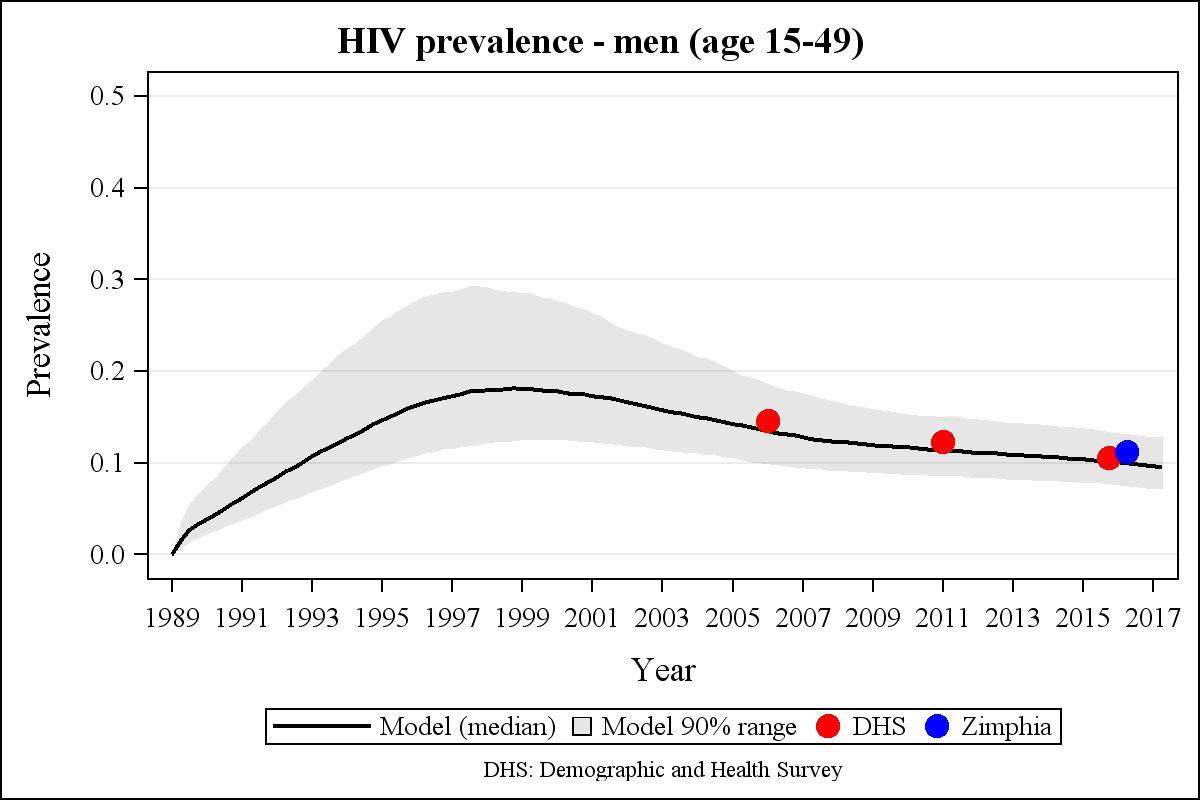


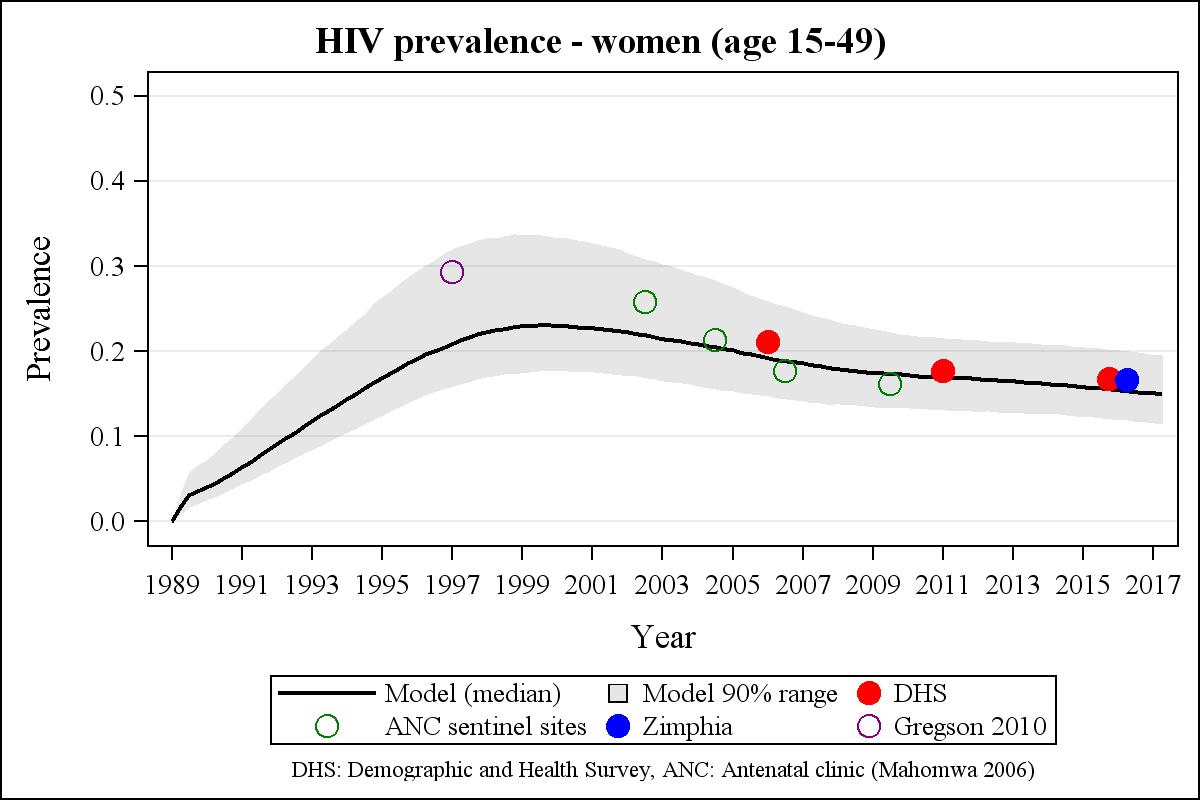


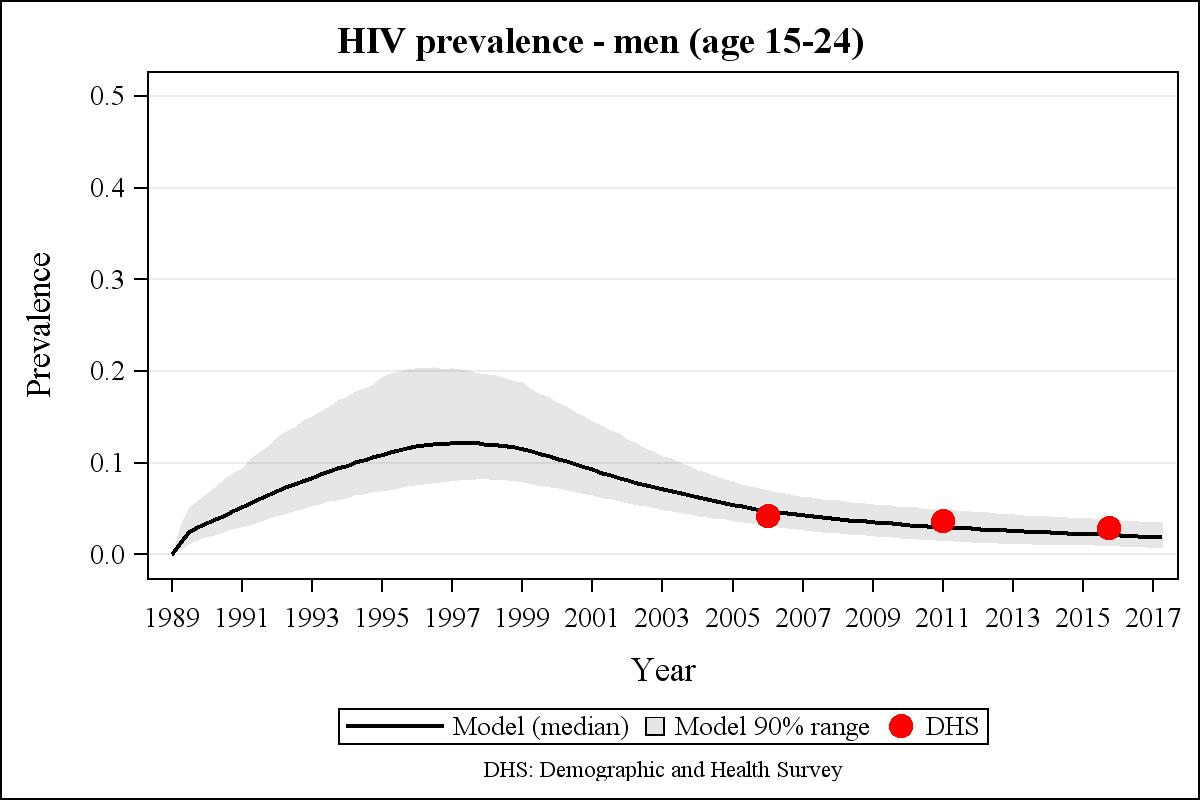


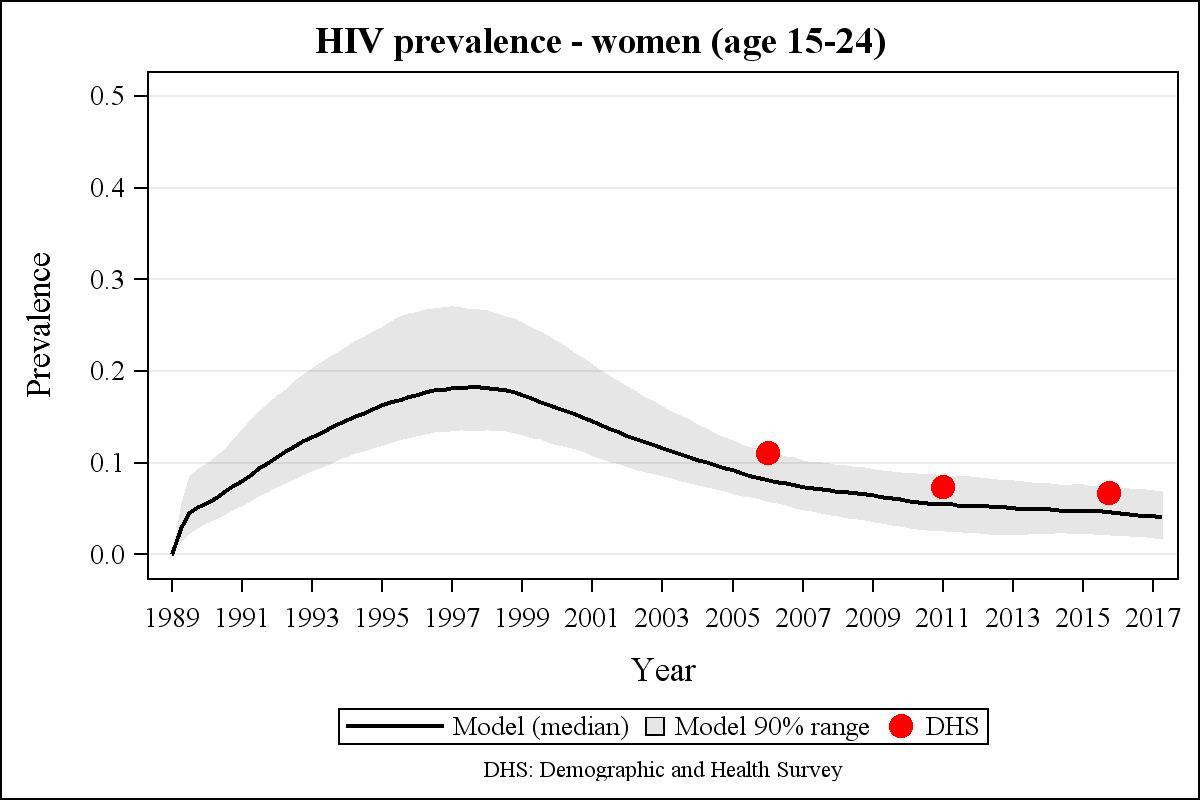


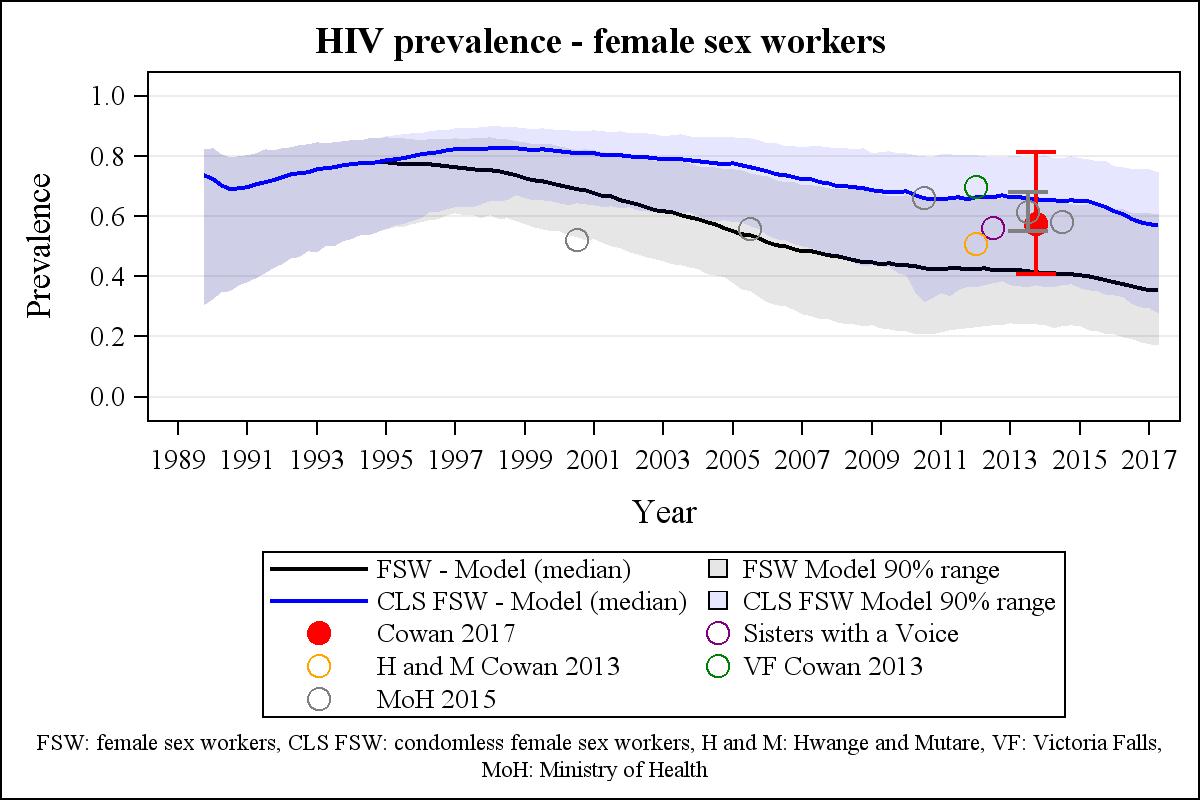


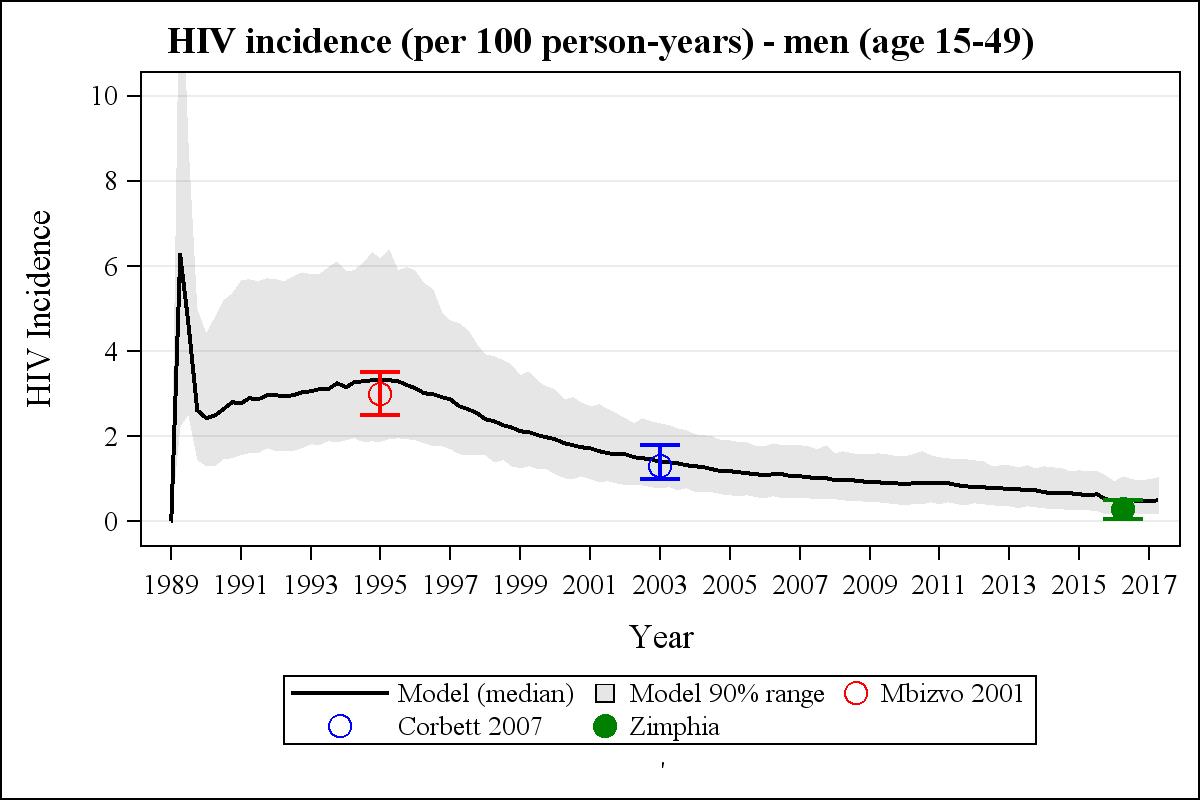


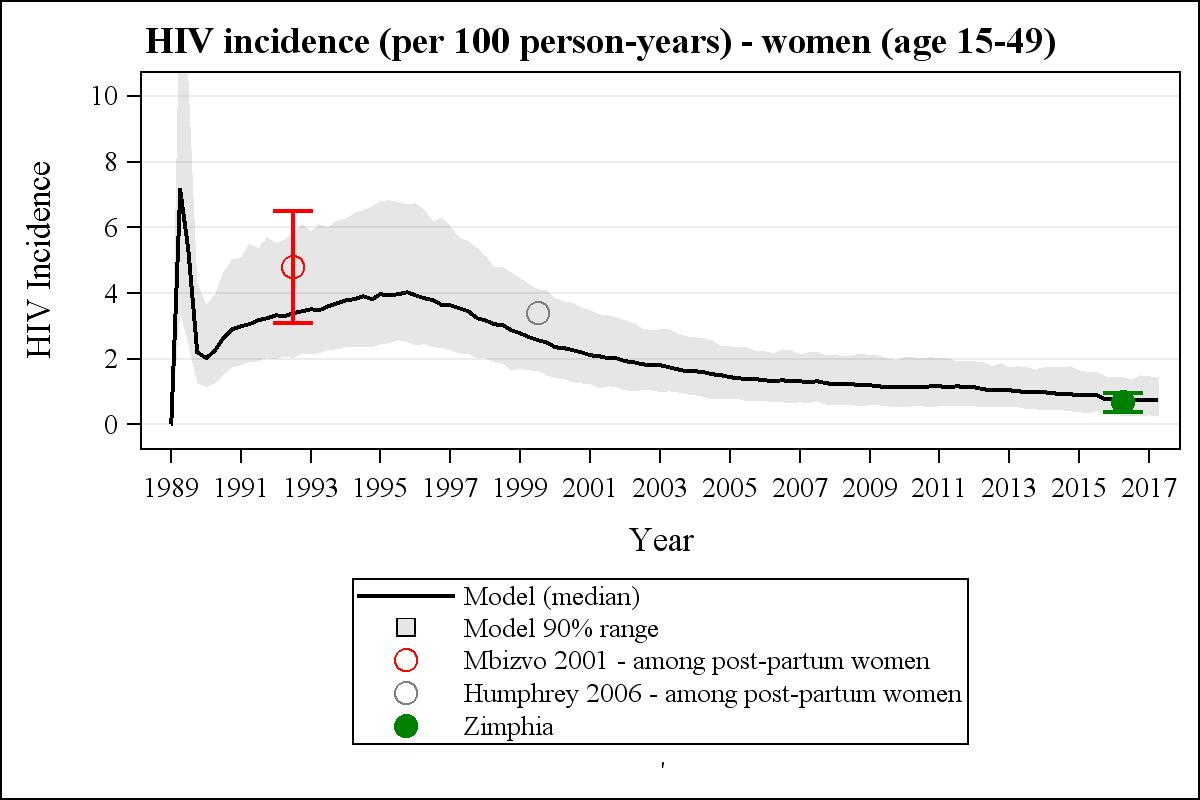


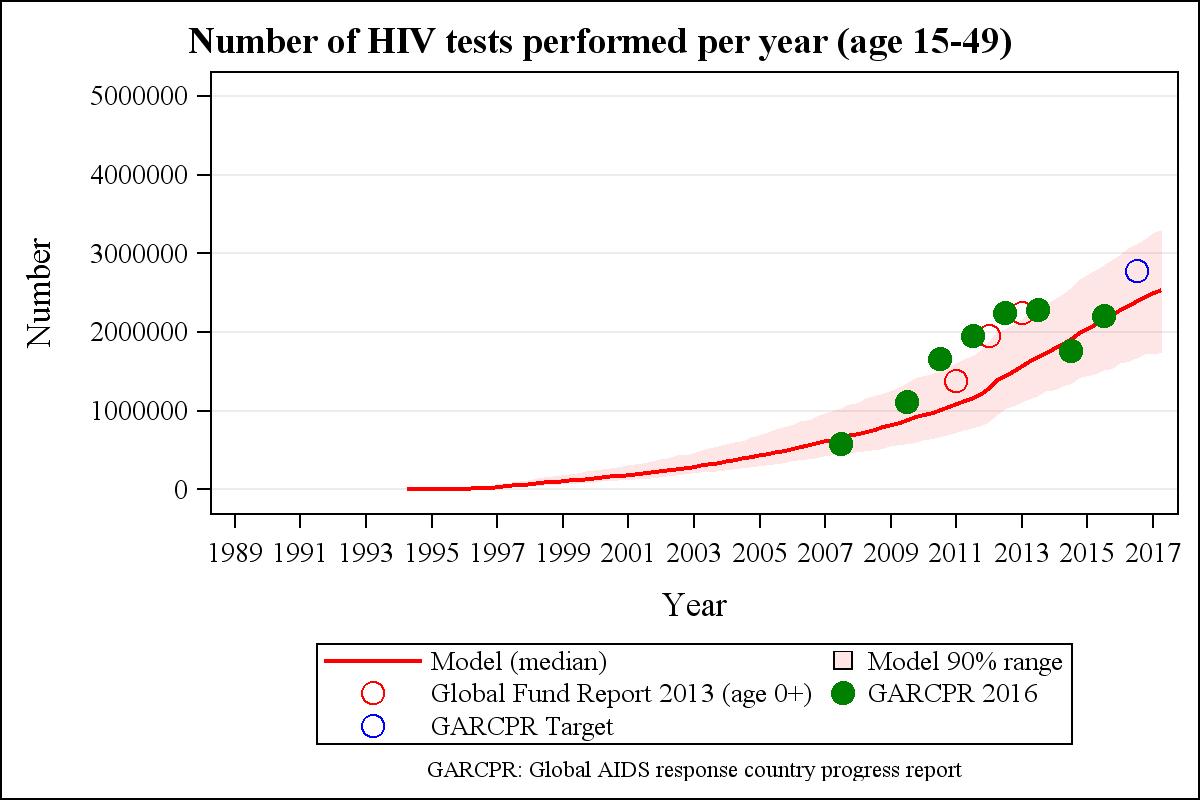


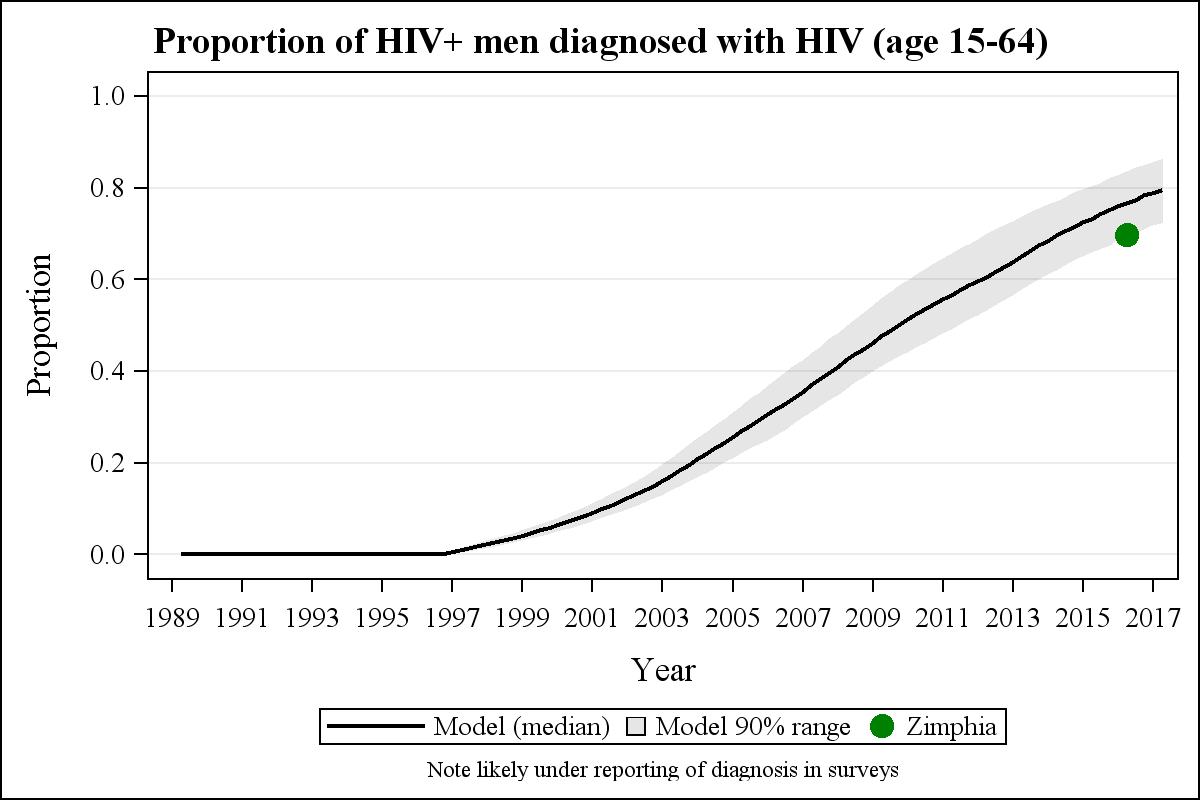


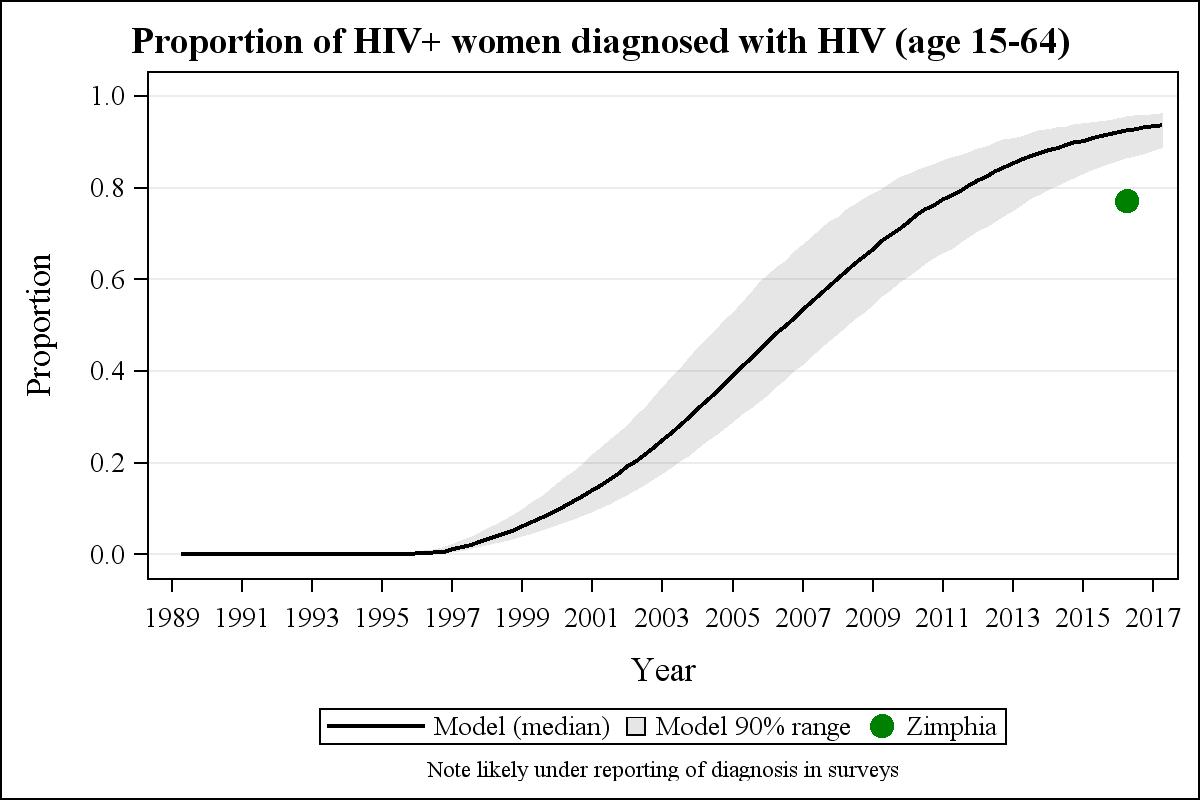


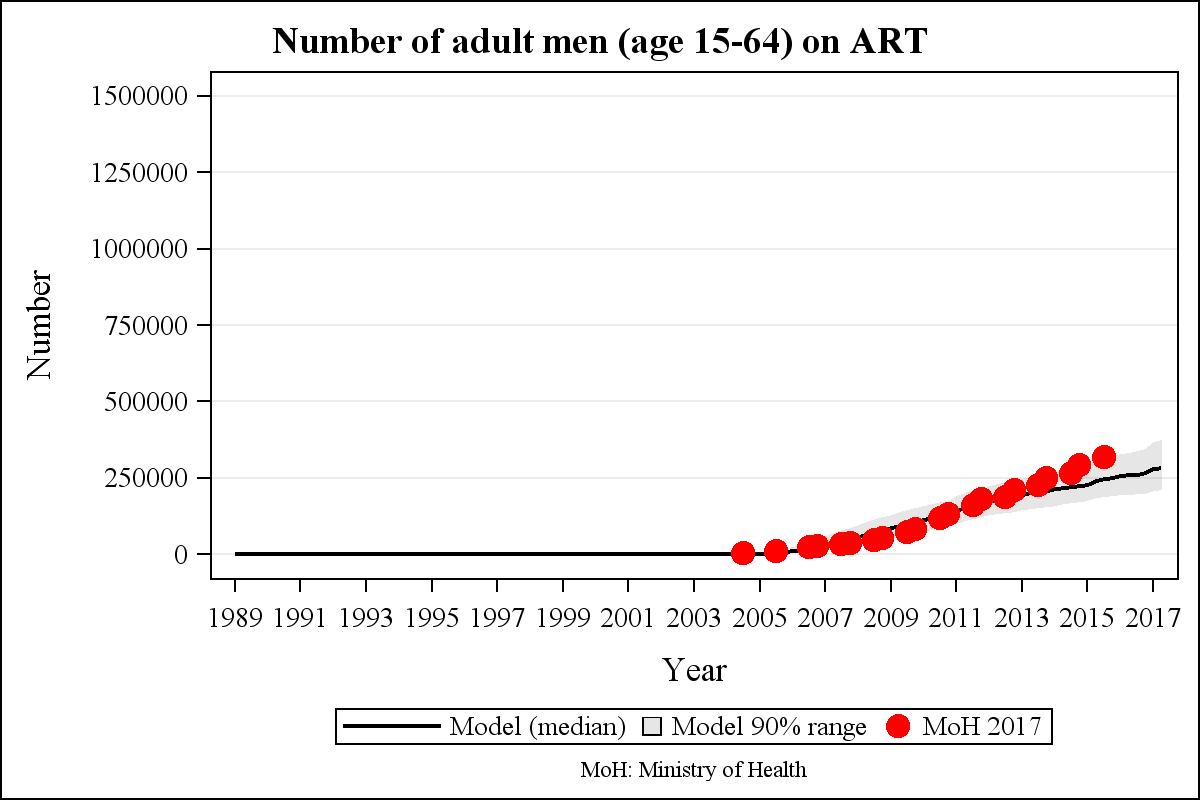


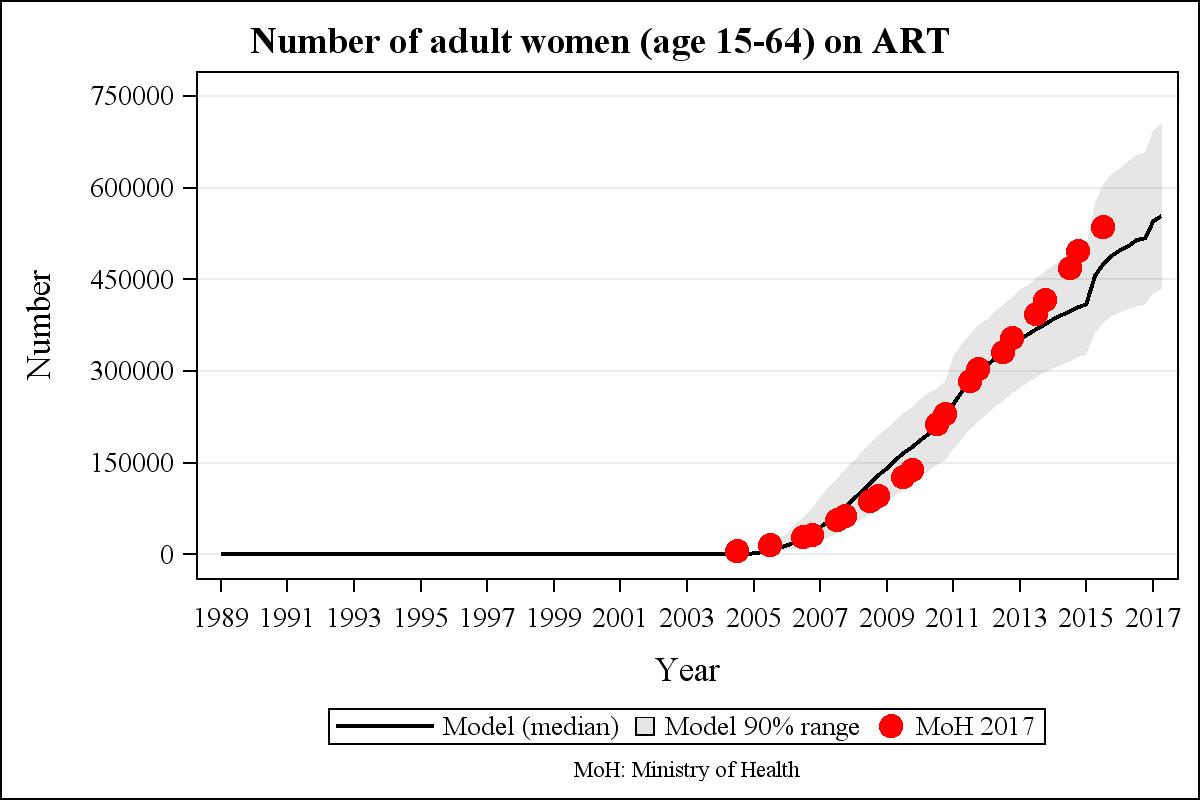


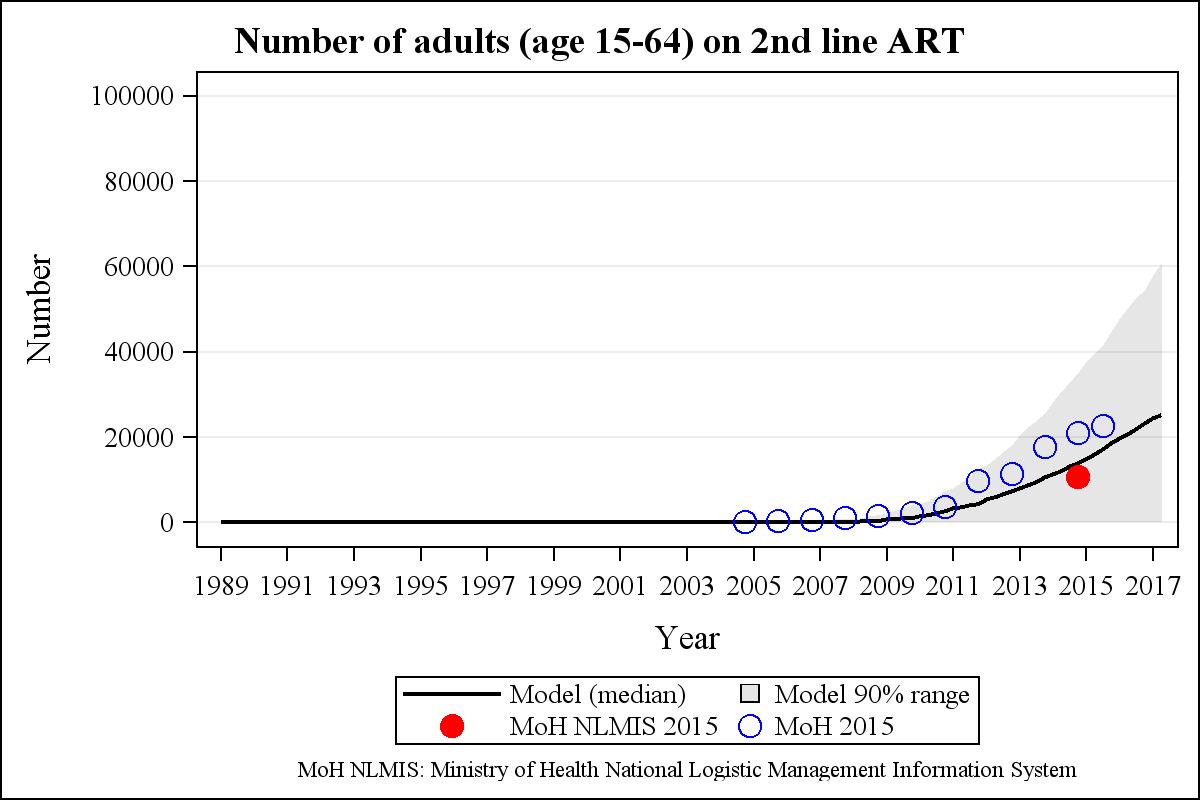


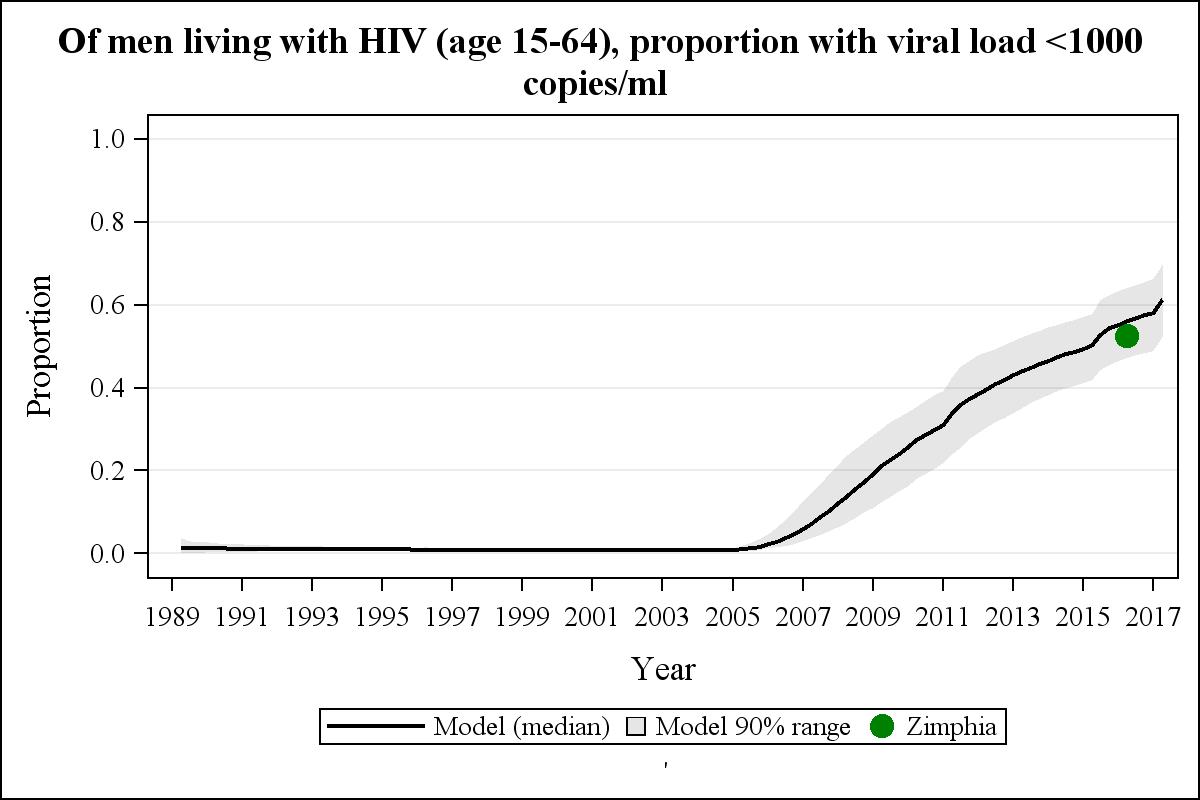


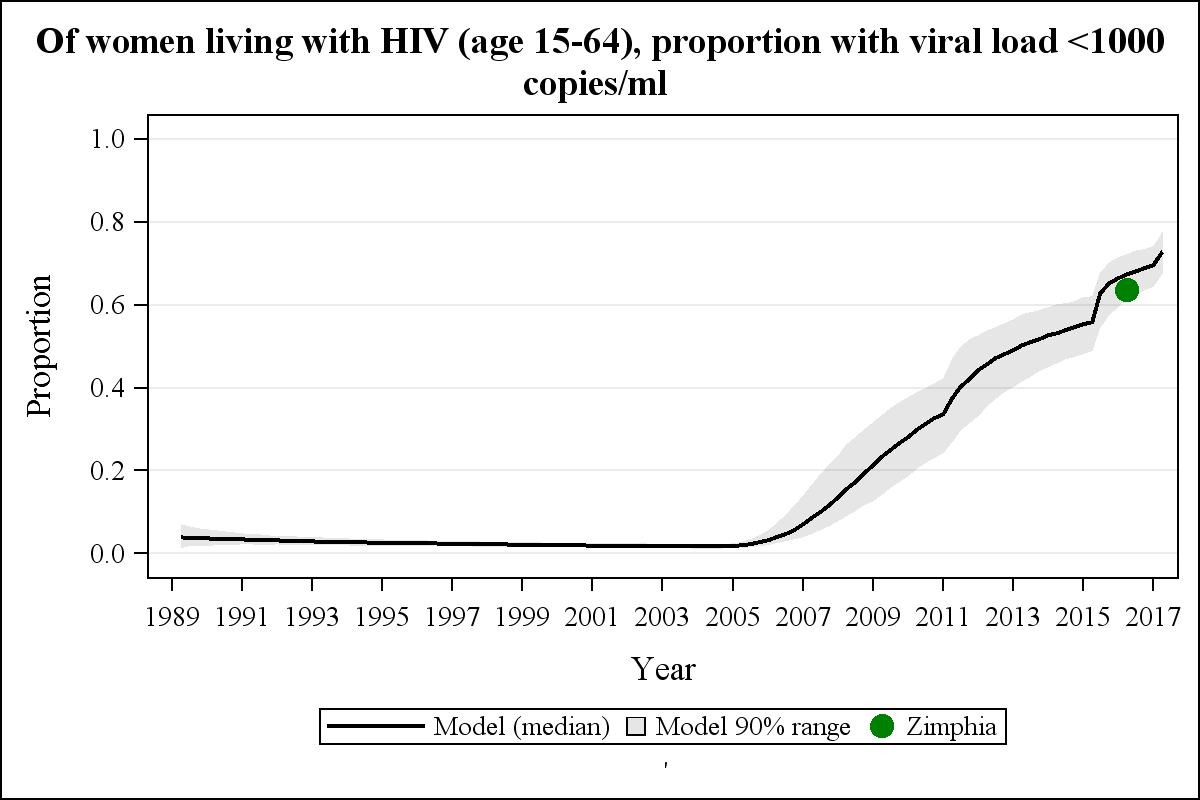

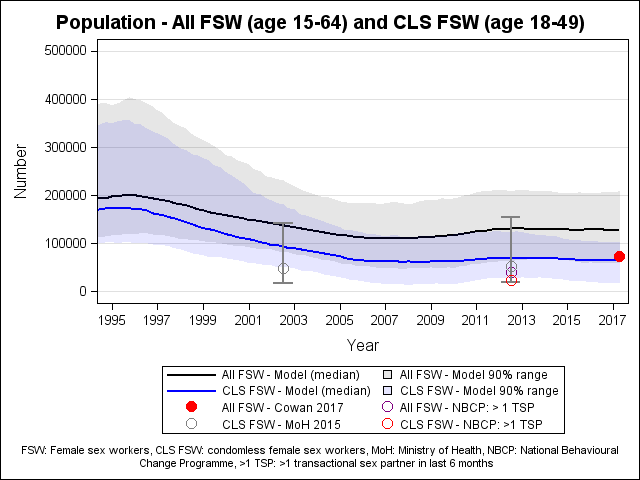


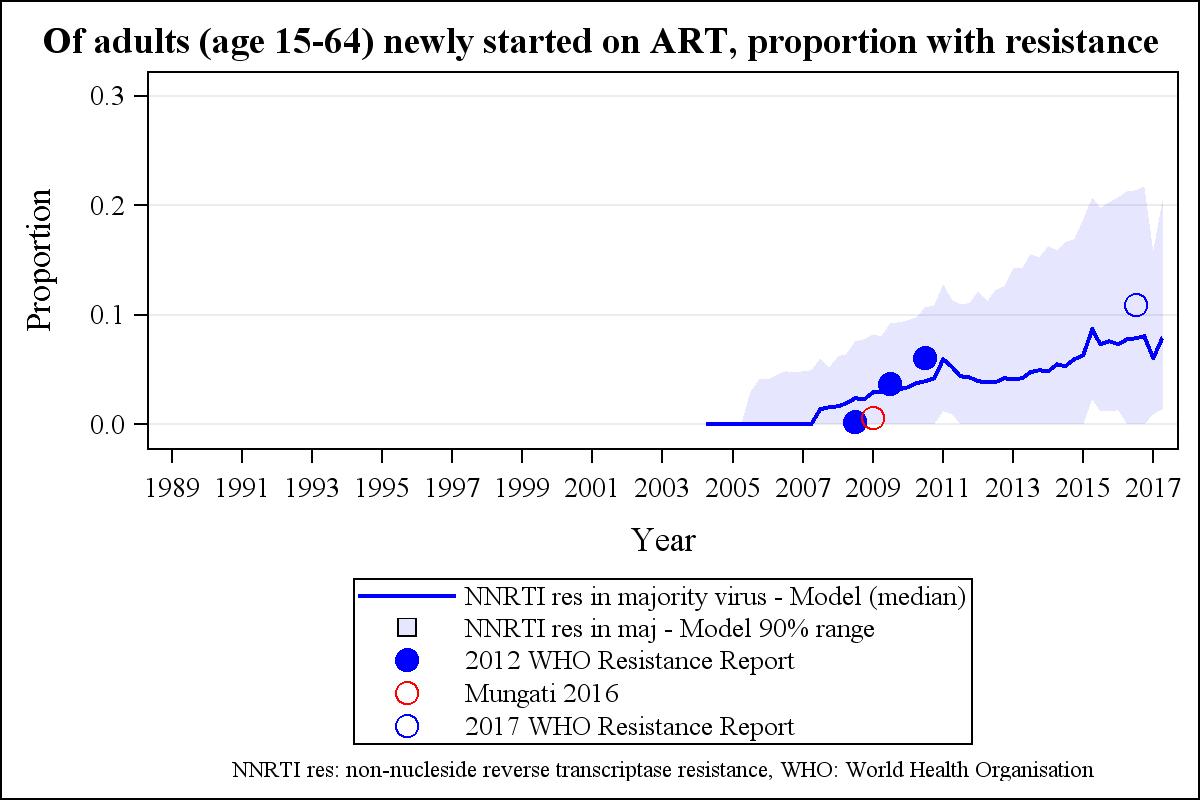


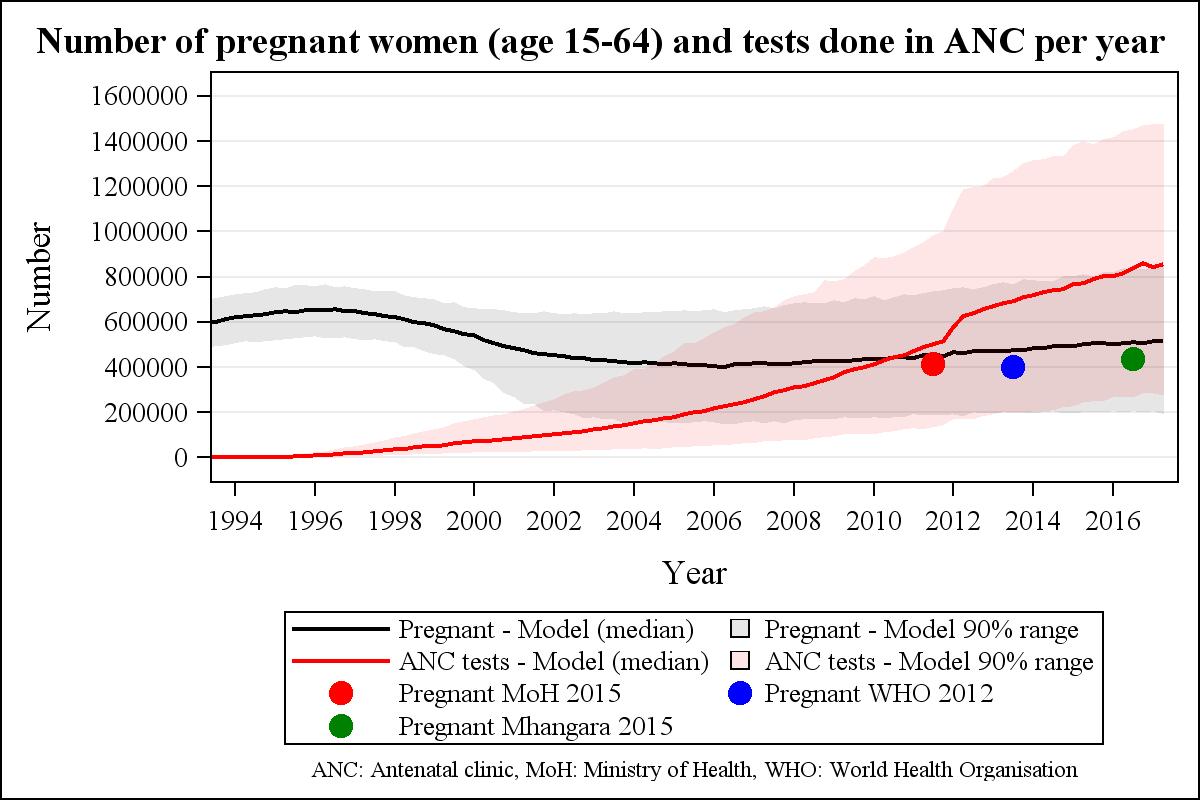


**Other model outputs**

***Demographics***


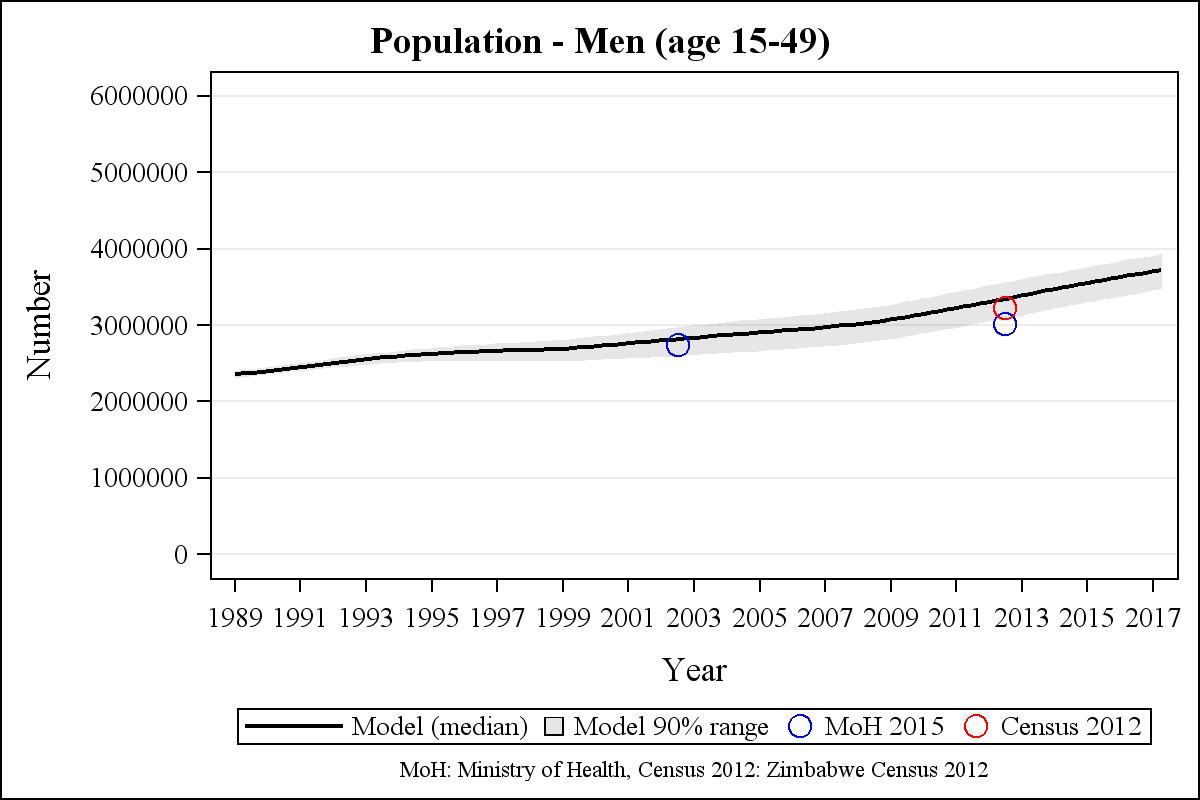


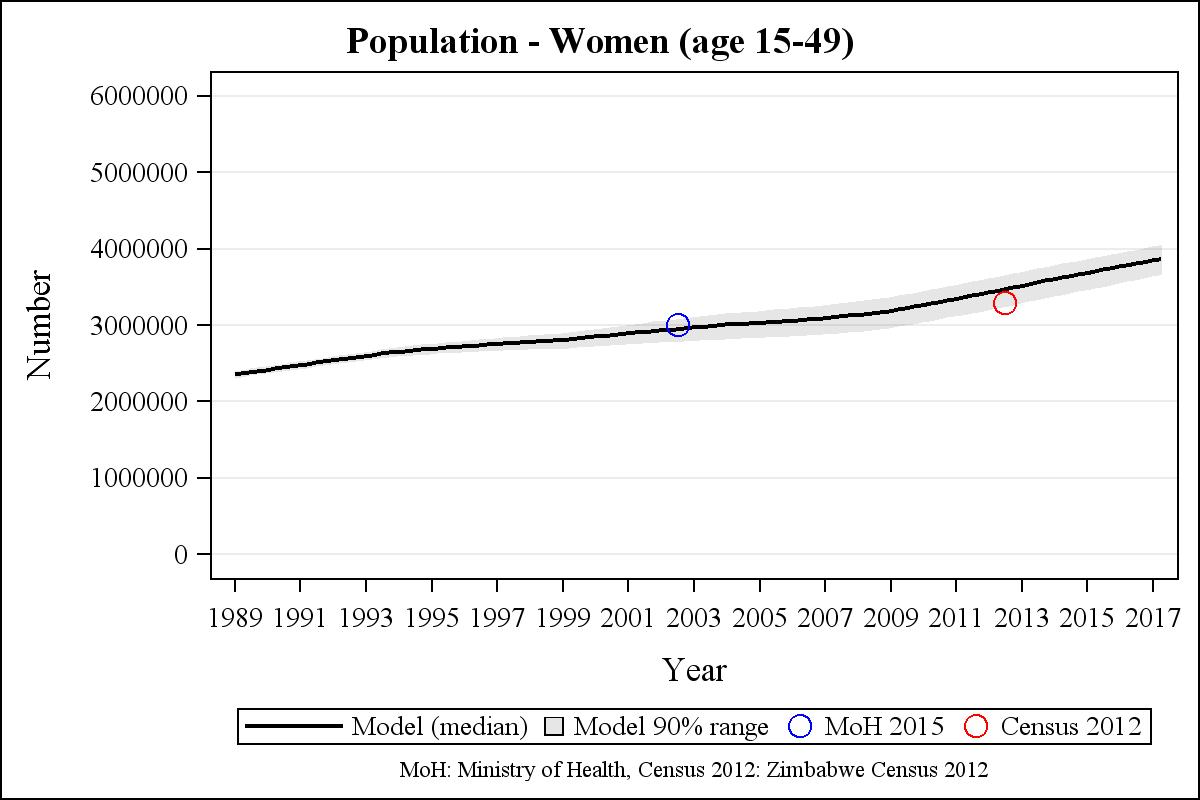


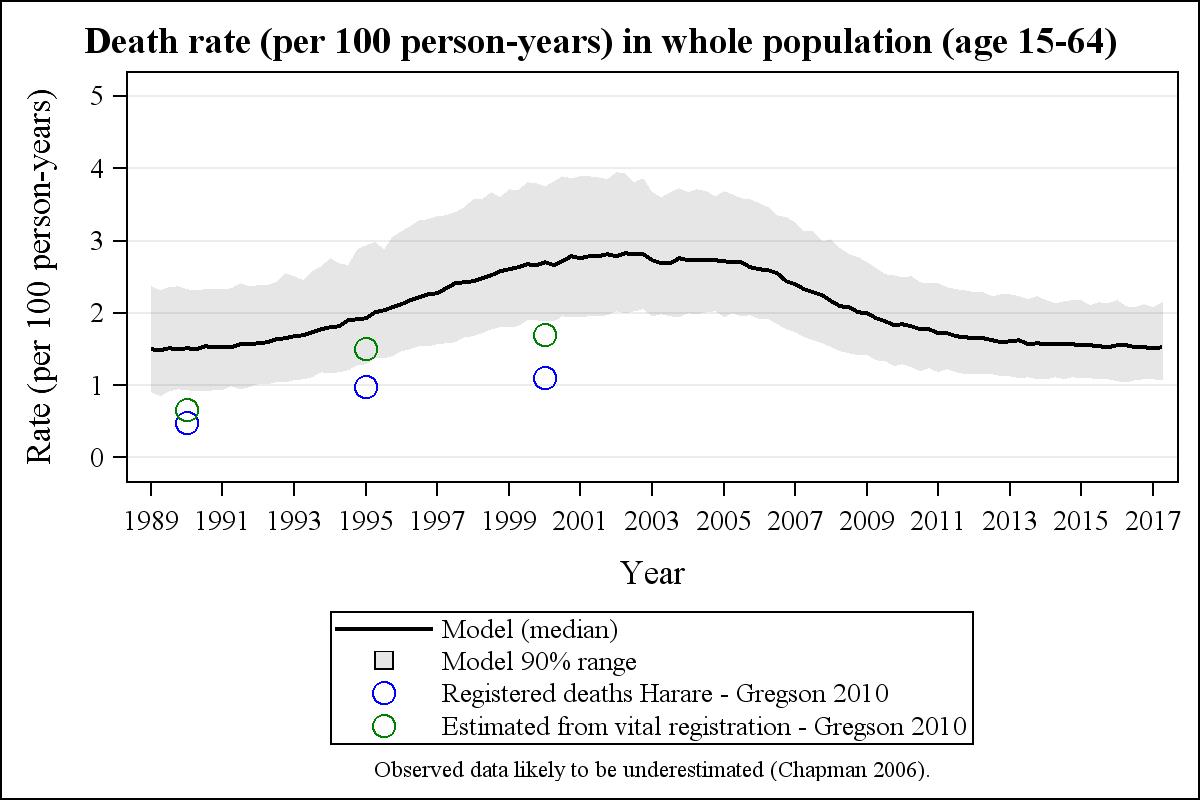


**Modelled and observed (Demographic and Health Survey [DHS])^1^ death rates, stratified by age**

|  | Year 🡪 | 1994.5 | 1999.5 | 2006 | 2011 | 2015 |
| --- | --- | --- | --- | --- | --- | --- |
|  | Age | Rate (90% range) | | | | |
| Death | 15-19 Model | 0.49 (0, 1.02) | 0.47 (0, 1.01) | 0.40 (0, 1.02) | 0.38 (0, 0.97) | 0.36 (0, 0.92) |
| rate | *15-19 Observed* | *0.05* | *0.05* | *0.07* | *0.23* | *0.17* |
| (males) | 20-24 Model | 0.98 (0, 2.25) | 1.27 (0.27, 2.56) | 0.92 (0.23, 1.70) | 0.64 (0.20, 1.61) | 0.62 (0.20, 1.45) |
|  | *20-24 Observed* | *0.20* | *0.50* | *0.34* | *0.34* | *0.27* |
|  | 25-29 Model | 1.78 (0.37, 3.48) | 2.81 (1.33, 5.50) | 2.45 (1.09, 4.45) | 1.45 (0.49, 2.83) | 1.29 (0.43, 2.71) |
|  | *25-29 Observed* | *0.35* | *0.95* | *0.90* | *0.64* | *0.43* |
|  | 30-34 Model | 2.44 (0.83, 4.44) | 3.94 (1.82, 7.16) | 3.93 (1.69, 6.87) | 2.26 (0.92, 4.38) | 1.29 (0.81, 2.71) |
|  | *30-34 Observed* | *0.50* | *2.00* | *2.01* | *1.40* | *0.86* |
|  | 35-39 Model | 2.52 (0.85, 4.92) | 4.32 (1.94, 7.75) | 4.48 (2.02, 8.41) | 2.65 (0.98, 5.12) | 2.32 (0.73, 4.57) |
|  | *35-39 Observed* | *0.60* | *2.20* | *2.77* | *2.30* | *1.22* |
|  | 40-44 Model | 2.74 (0.59, 5.46) | 4.34 (1.98, 8.12) | 4.86 (2.03, 8.83) | 3.04 (1.08, 5.96) | 2.87 (1.09, 5.48) |
|  | *40-44 Observed* | *0.95* | *2.35* | *3.71* | *3.00* | *1.57* |
|  | 45-49 Model | 3.15 (0.62, 6.12) | 4.25 (1.35, 8.10) | 4.55 (1.81, 8.68) | 3.17 (0.84, 6.83) | 3.16 (0.78, 6.57) |
|  | *45-49 Observed* | *1.15* | *2.95* | *3.65* | *3.20* | *2.62* |
| Death | 15-19 Model | 0.25 (0, 0.99) | 0.24 (0, 0.97) | 0.21 (0, 0.82) | 0.20 (0, 0.77) | 0.18 (0, 0.72) |
| rate | *15-19 Observed* | *0.15* | *0.25* | *0.27* | *0.25* | *0.19* |
| (females) | 20-24 Model | 0.96 (0, 2.00) | 1.26 (0.26, 2.41) | 0.91 (0.23, 1.86) | 0.62 (0, 1.44) | 0.60 (0, 1.39) |
|  | *20-24 Observed* | *0.20* | *0.50* | *0.55* | *0.52* | *0.25* |
|  | 25-29 Model | 1.35 (0.34, 2.75) | 2.54 (1.06, 4.85) | 2.43 (0.89, 4.07) | 1.18 (0.25, 2.30) | 0.90 (0.22, 1.96) |
|  | *25-29 Observed* | *0.35* | *1.10* | *1.23* | *0.96* | *0.50* |
|  | 30-34 Model | 1.24 (0.39, 2.78) | 2.82 (1.15, 5.34) | 3.49 (1.53, 6.05) | 1.51 (0.57, 3.08) | 1.13 (0.28, 2.37) |
|  | *30-34 Observed* | *0.40* | *1.50* | *2.04* | *1.64* | *1.08* |
|  | 35-39 Model | 1.31 (0, 2.99) | 2.99 (0.96, 5.42) | 3.81 (1.77, 6.95) | 1.88 (0.42, 3.99) | 1.55 (0.60, 3.29) |
|  | *35-39 Observed* | *0.50* | *1.65* | *2.50* | *2.07* | *1.36* |
|  | 40-44 Model | 1.60 (0, 3.71) | 2.91 (0.95, 5.73) | 4.07 (1.73, 7.89) | 2.19 (0.53, 4.86) | 1.94 (0.50, 4.31) |
|  | *40-44 Observed* | *0.55* | *1.20* | *2.52* | *2.33* | *1.52* |
|  | 45-49 Model | 1.79 (0.53, 4.12) | 3.03 (0.68, 5.82) | 3.92 (1.42, 7.26) | 2.44 (0.60, 5.08) | 2.26 (0.58, 5.31) |
|  | *45-49 Observed* | *0.60* | *1.25* | *2.65* | *2.26* | *1.71* |

***^1^*** *DHS:* Zimbabwe Demographic and Health Survey 2005-2006, 2010-2011, 2015-2016 Central Statistical Office

*There is a substantial lack of information on death rates. The lower observed death rates are likely to be due to underreporting of deaths, which were based on report of deaths in siblings.*

***Female sex work (FSW)***

(These graphs start from 1995 as it is assumed condoms were introduced at this point, resulting in a decline in condomless sex in the general population)


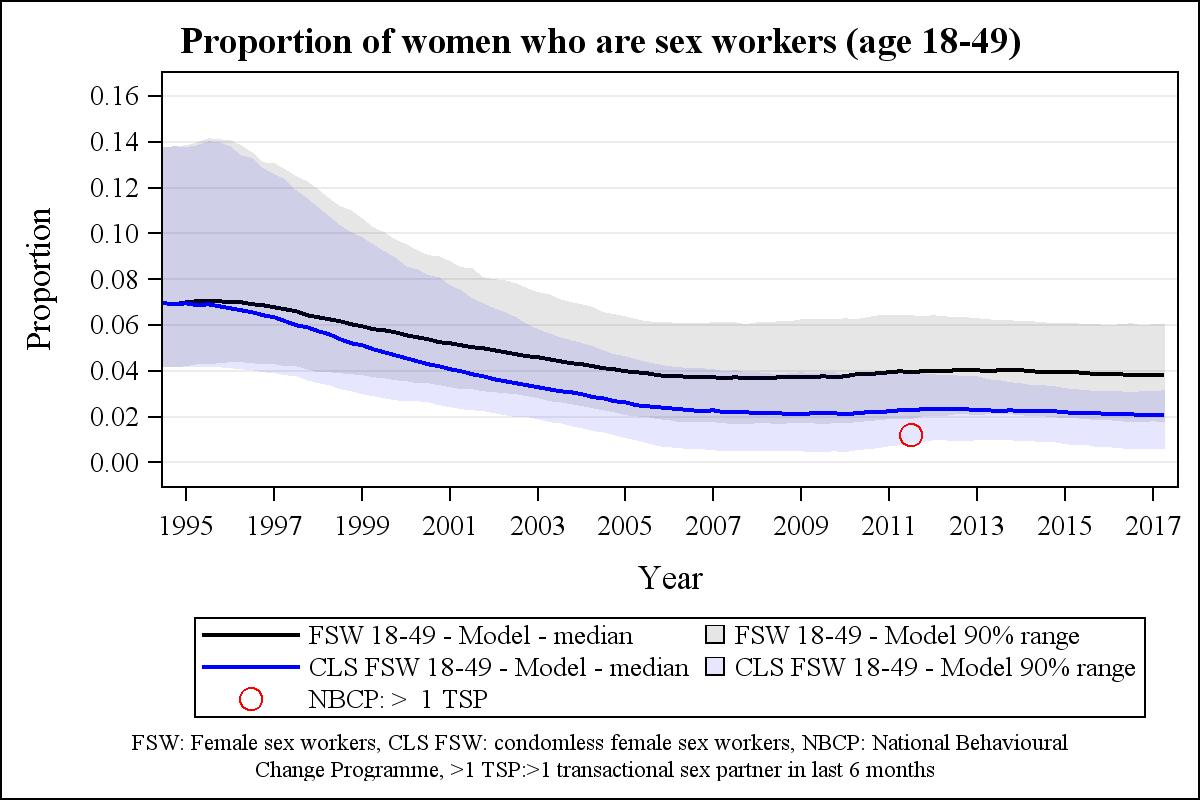

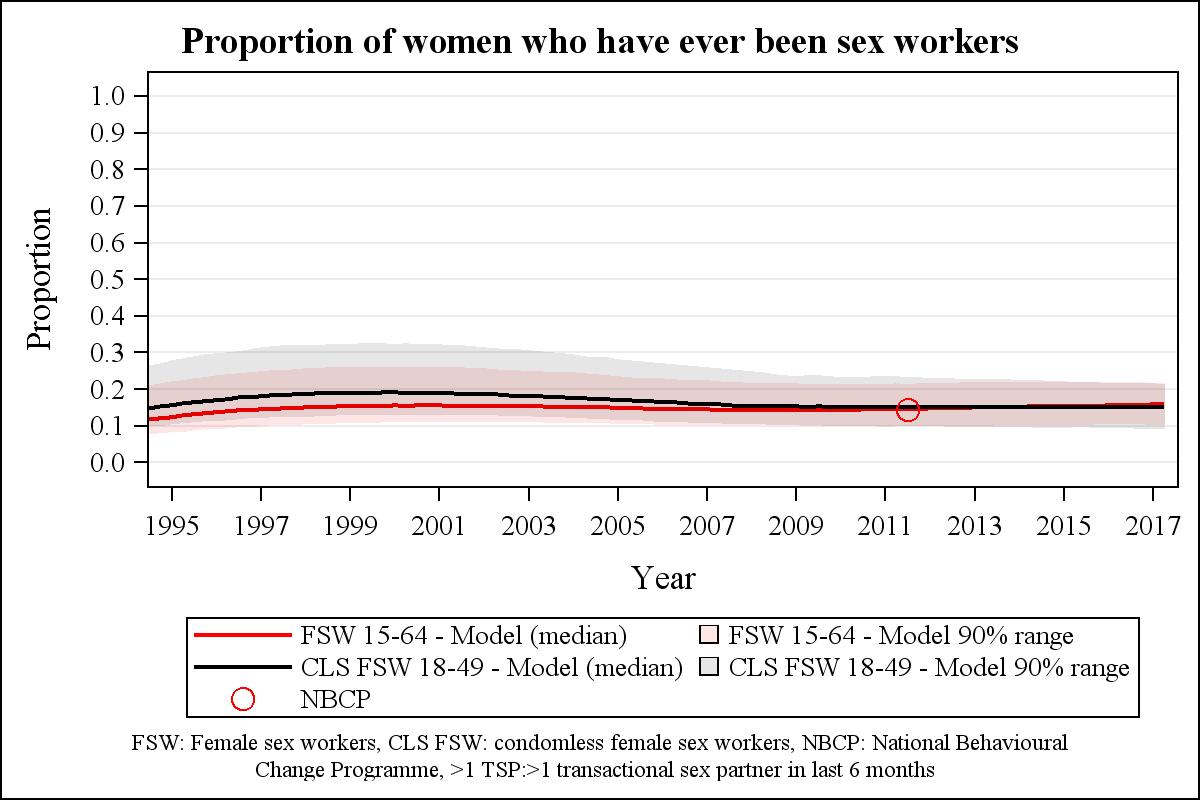


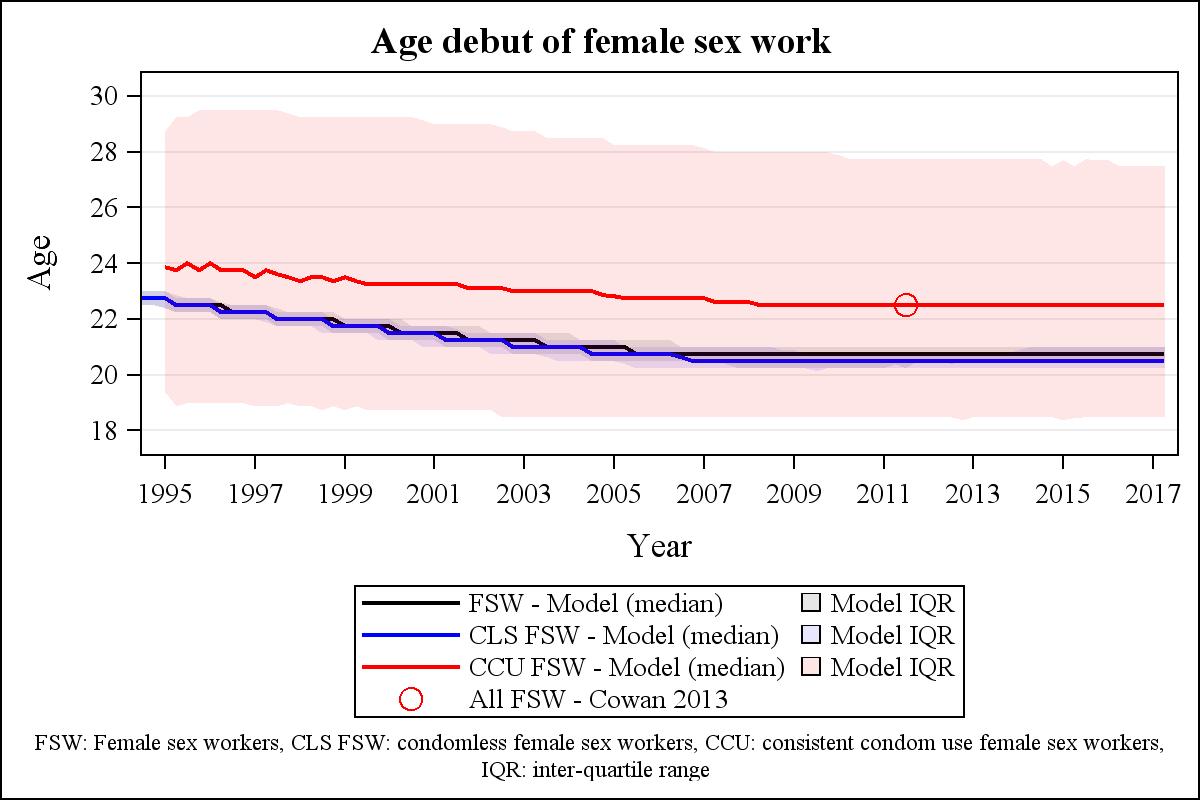


**Modelled and observed (Cowan 2017^1^) proportion of female sex workers in each age band**

| Age | 18-24 | 25-29 | 30-39 | >=40 |
| --- | --- | --- | --- | --- |
| Model (All FSW) | 0.42 (0.34, 0.52) | 0.25 (0.21, 0.29) | 0.25 (0.18, 0.30) | 0.08 (0.04, 0.13) |
| *Observed (All FSW)* | *0.27 (0.10, 0.38)* | *0.24 (0.18, 0.33)* | *0.34 (0.23, 0.45)* | *0.16 (0.07, 0.22)* |

^1^ The HIV Care Cascade Among Female Sex Workers in Zimbabwe: Results of a Population-Based Survey From the Sisters Antiretroviral Therapy Programme for Prevention of HIV, an Integrated Response (SAPPH-IRe) Trial (Cowan 2017 [under-sampling of younger FSW considered]).

**Modelled and observed (Cowan 2013^1^) proportion of all current female sex workers who have been doing sex work for over 9 years**

| **Proportion of current FSW who have been doing sex work for over 9 years** |  |
| --- | --- |
| Model (median [90% range]) | 0.16 (0.09, 0.23) |
| Observed (>10 years) | 0.21 |

1 Cowan 2013: Engagement with HIV Prevention Treatment and Care among Female Sex Workers in Zimbabwe: a Respondent Driven Sampling Survey (Cowan 2013)

***HIV Prevalence and Incidence***


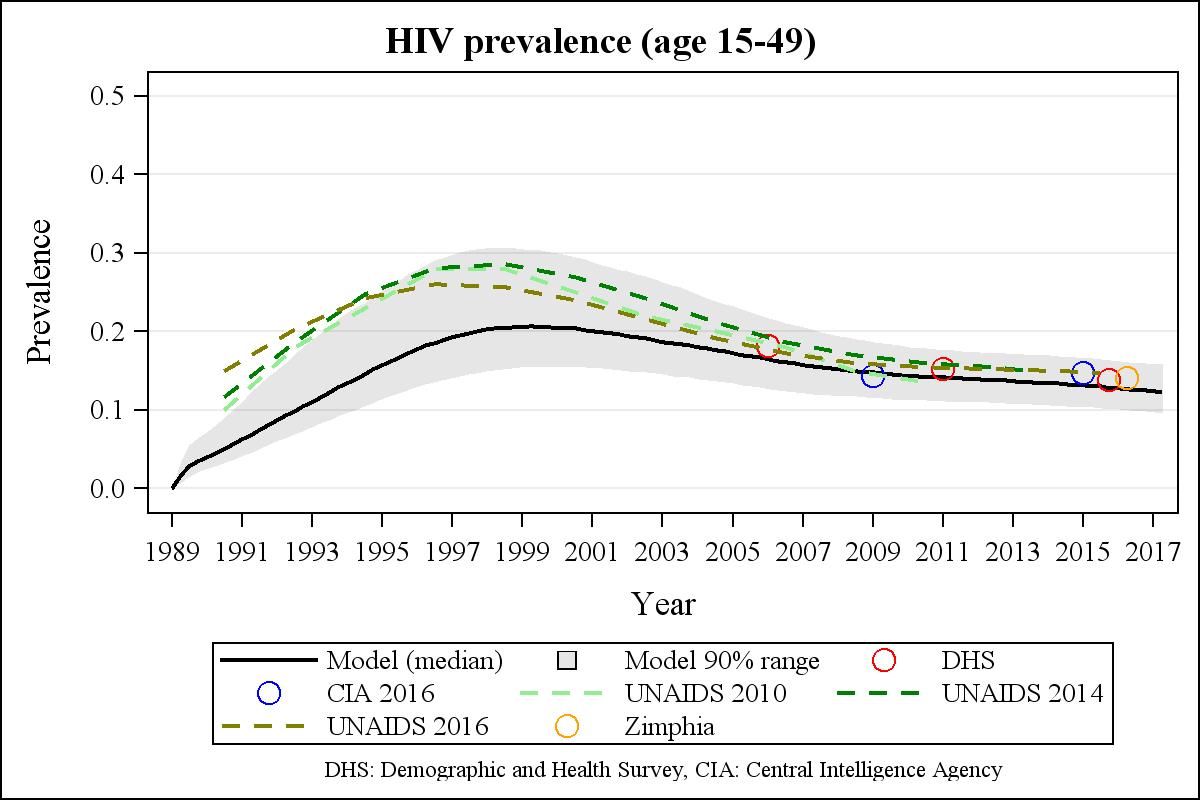


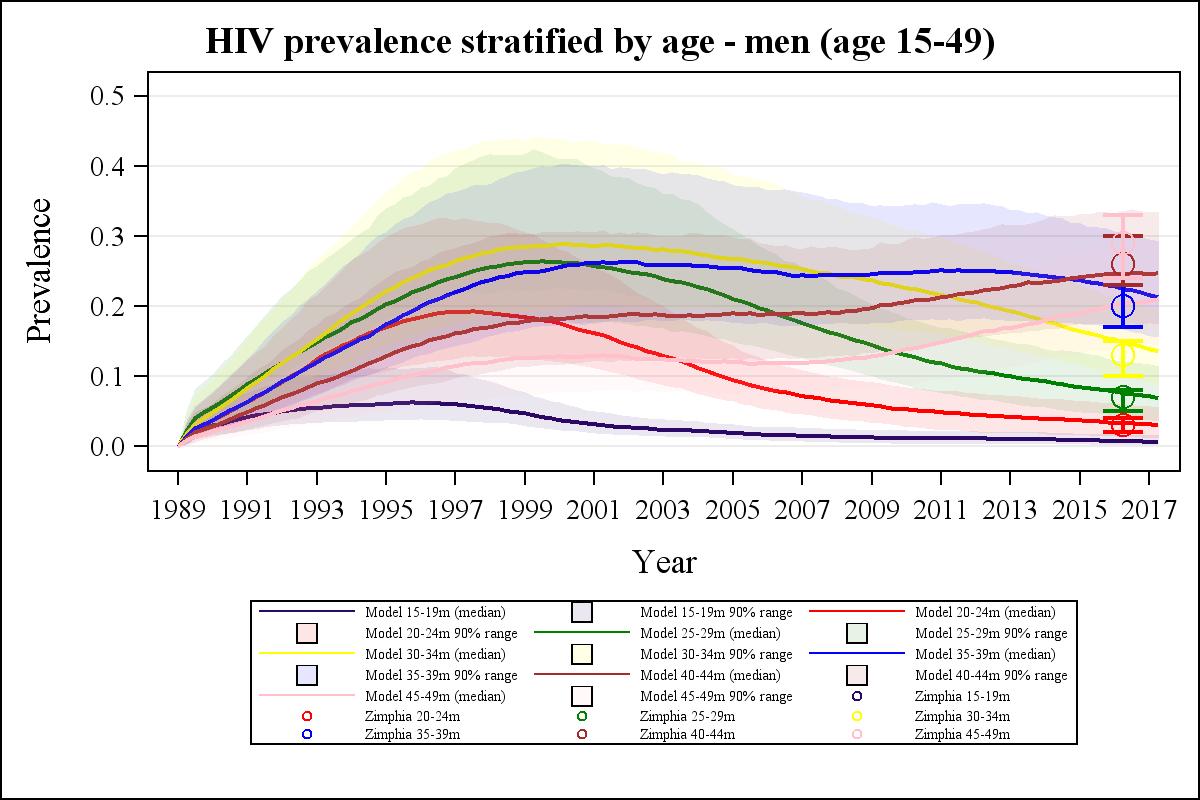


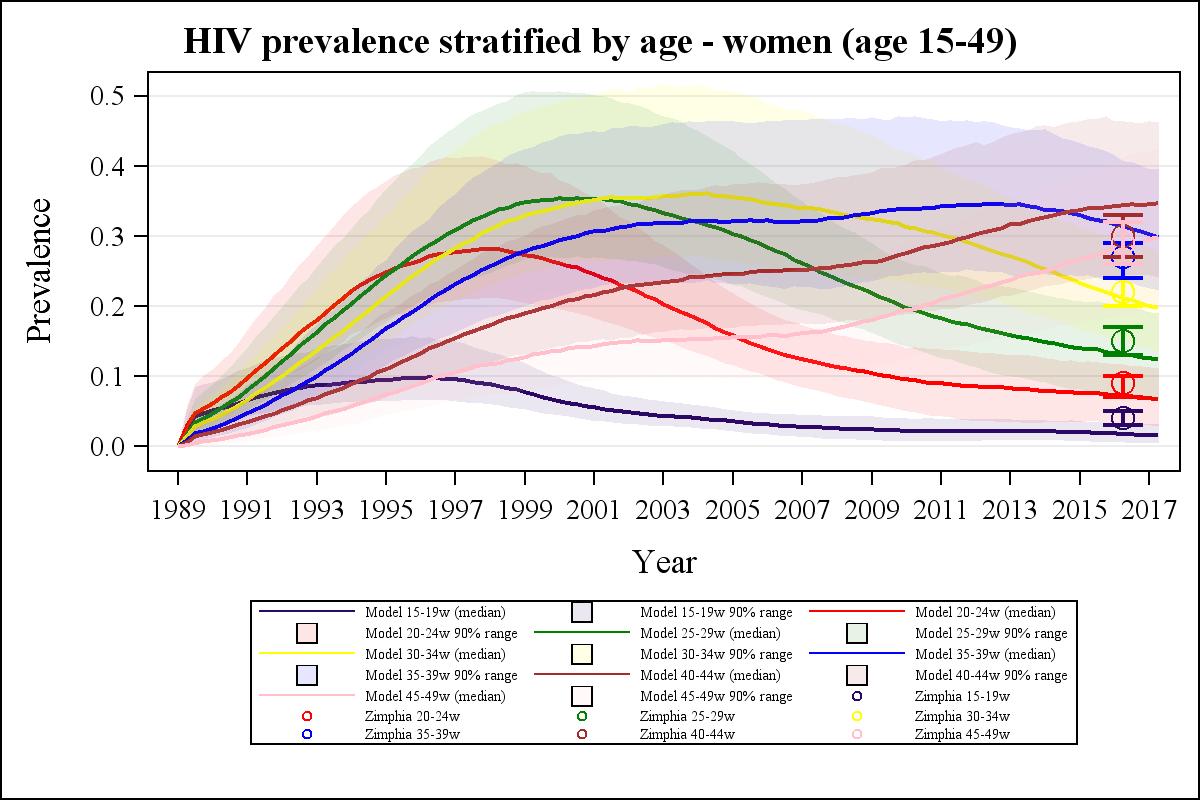


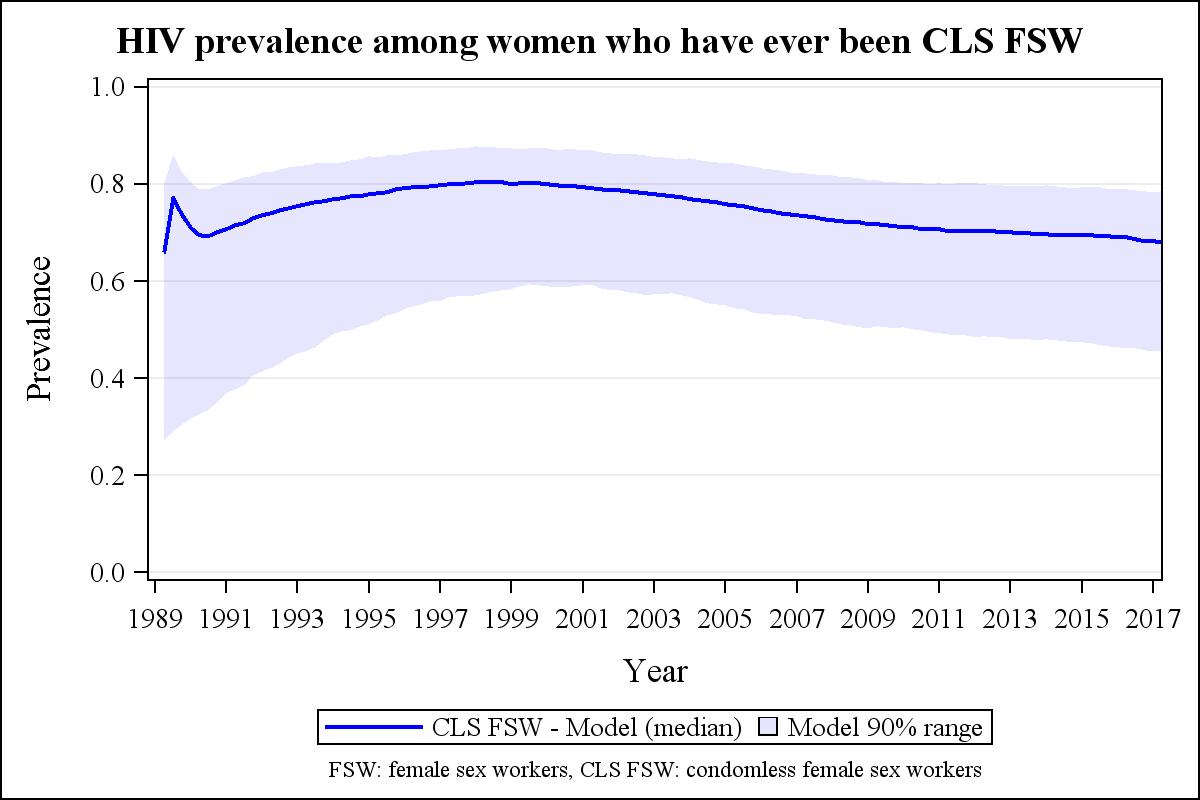


**Modelled and observed (Cowan 2017^1^) prevalence for female sex workers (FSW) in 2013**

| Age | 18-24 | 25-29 | 30-39 | >=40 |
| --- | --- | --- | --- | --- |
| Model (All FSW) | 0.45 (0.18, 0.68) | 0.44 (0.19, 0.73) | 0.45 (0.29, 0.73) | 0.29(0.14, 0.60) |
| Model (Condomless FSW) | 0.64 (0.29, 0.80) | 0.74 (0.42, 0.90) | 0.75 (0.52, 0.90) | 0.67 (0.17, 1.00) |
| *Observed (All FSW)* | *0.39* | *0.62* | *0.76* | *0.78* |

^1^ Draft paper: How can programmes better support female sex workers to avoid HIV infection in Zimbabwe? A prevention cascade analysis (Cowan 2017)

**Modelled and observed (Cowan 2017^1^) prevalence for female sex workers by duration of sex work (FSW) in 2013**

| Age | 18-24 | 25-29 | 30-39 | >=40 |
| --- | --- | --- | --- | --- |
| **Duration 0-2 years** |  |  |  |  |
| Model (All FSW) | 0.42(0.16, 0.65) | 0.39 (0.18, 0.68) | 0.44 (0.20, 0.70) | 0.25 (0, 0.67) |
| Model (Condomless FSW) | 0.56 (0.22, 0.75) | 0.58 (0.24, 0.83) | 0.57 (0.27, 0.82) | 0.50 (0, 1.00) |
| *Observed (All FSW)* | *0.31* | *0.53* | *0.63* | *0.80* |
| **Duration 3-4 years** |  |  |  |  |
| Model (All FSW) | *0.54 (0.17, 0.89)* | *0.50 (0.11, 0.95)* | *0.40 (0, 1.00)* | *0.00 (0, 1.00)* |
| Model (Condomless FSW) | *0.95 (0.50, 1.00)* | *1.00 (0.50, 1.00)* | *1.00 (0.33, 1.00)* | *1.00 (0, 1.00)* |
| *Observed (All FSW)* | *0.43* | *0.54* | *0.63* | *0.67* |
| **Duration 5-8 years** |  |  |  |  |
| Model (All FSW) | *0.44(0.05, 0.89)* | *0.40 (0.09, 0.89)* | *0.32 (0.07, 0.80)* | *0.14 (0, 0.67)* |
| Model (Condomless FSW) | *1.00 (0.50, 1.00)* | *1.00 (0.50, 1.00)* | *1.00 (0.50, 1.00)* | *1.00 (0, 1.00)* |
| *Observed (All FSW)* | *0.35* | *0.55* | *0.66* | *0.76* |

^1^ Draft paper: How can programmes better support female sex workers to avoid HIV infection in Zimbabwe? A prevention cascade analysis (Cowan 2017)


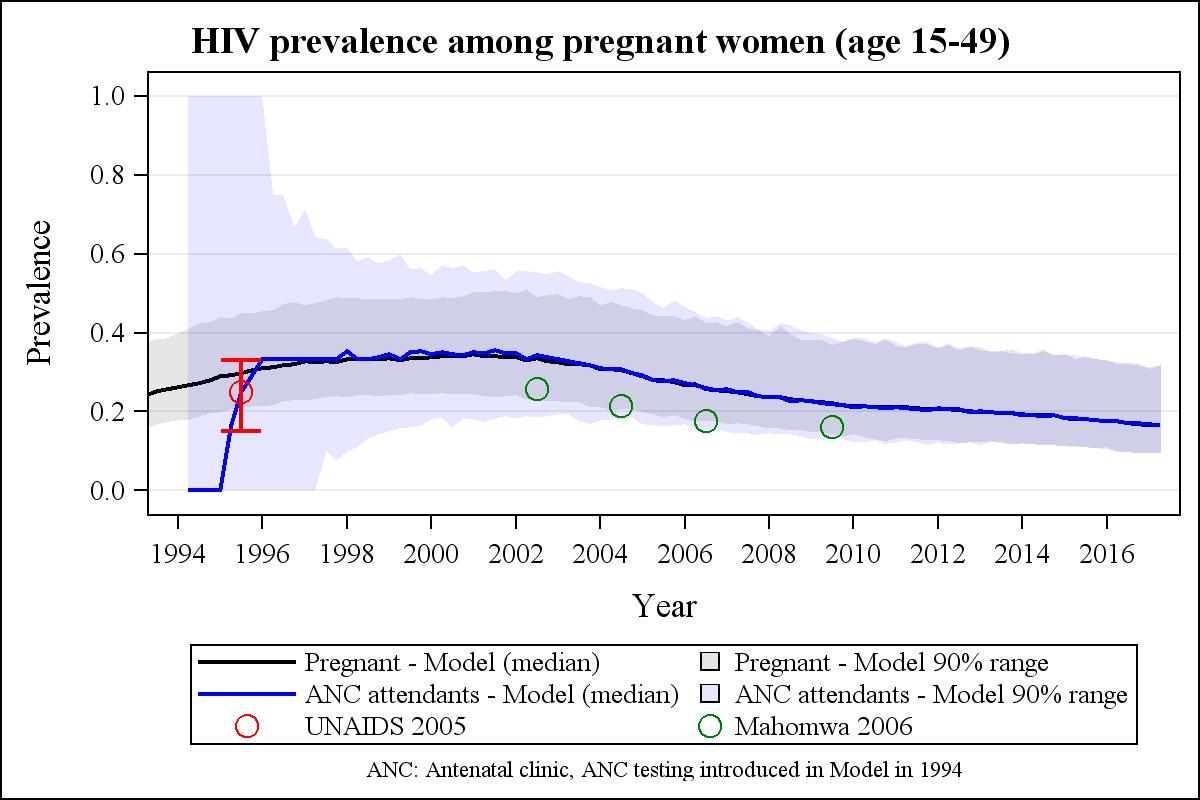


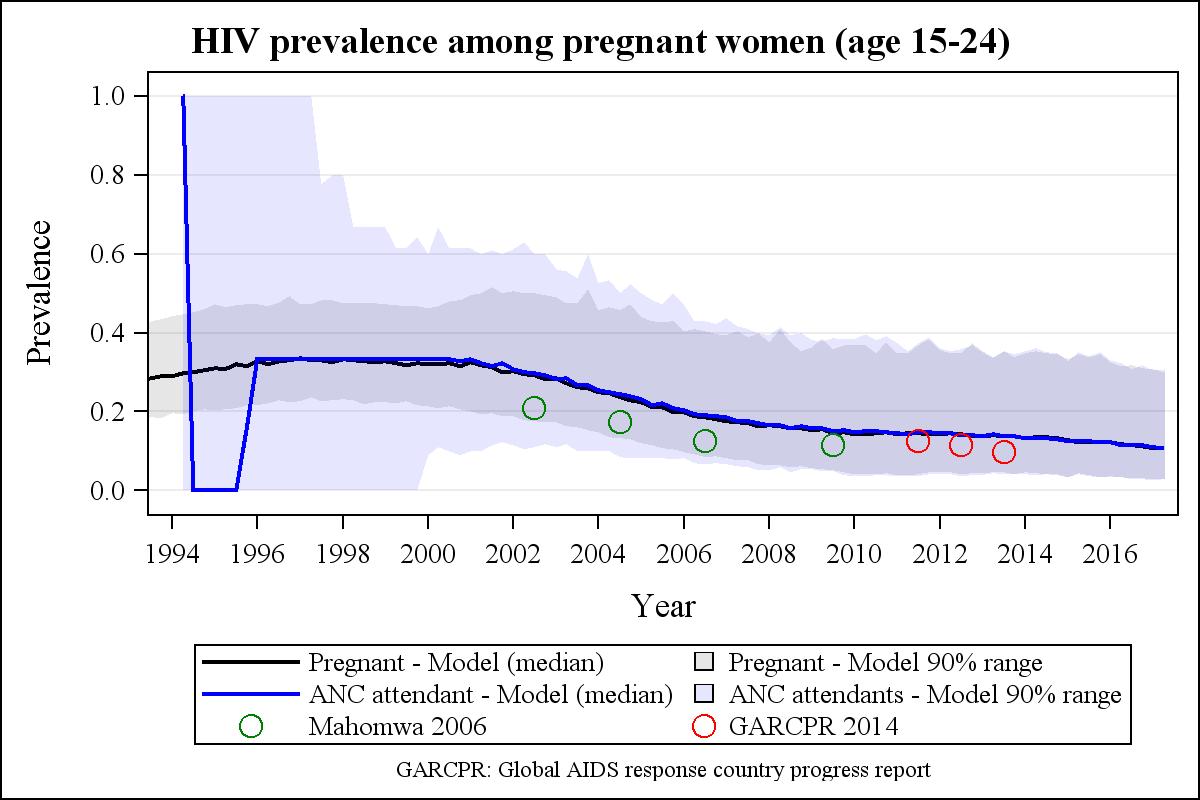


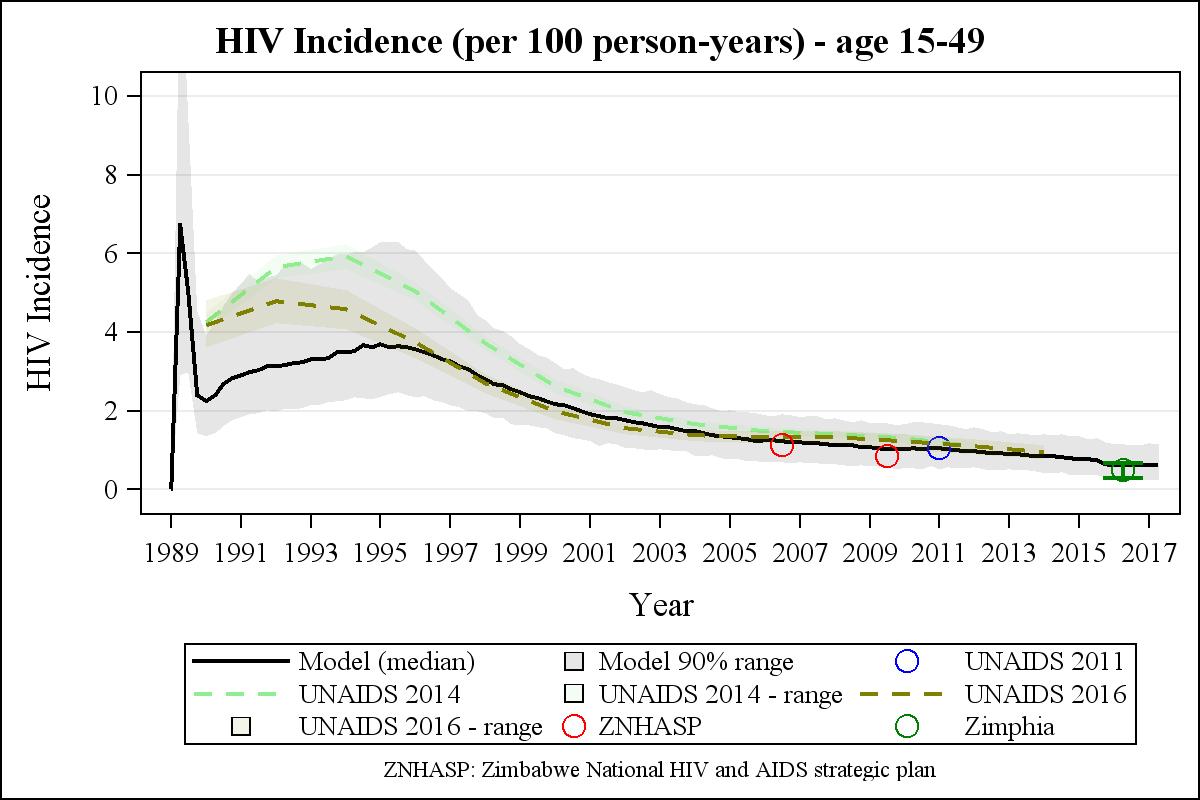


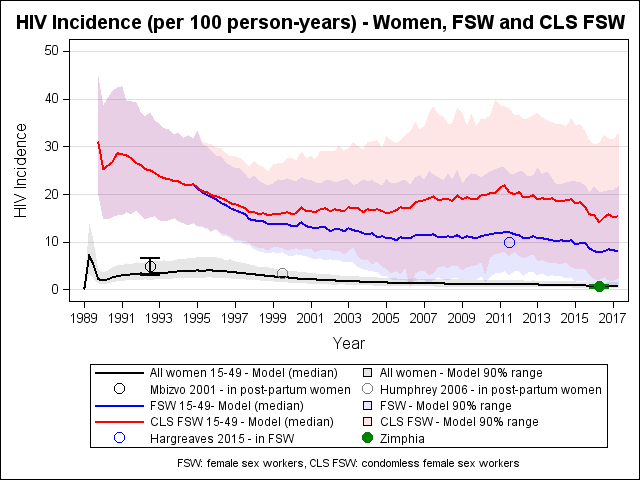


***HIV symptoms and testing***


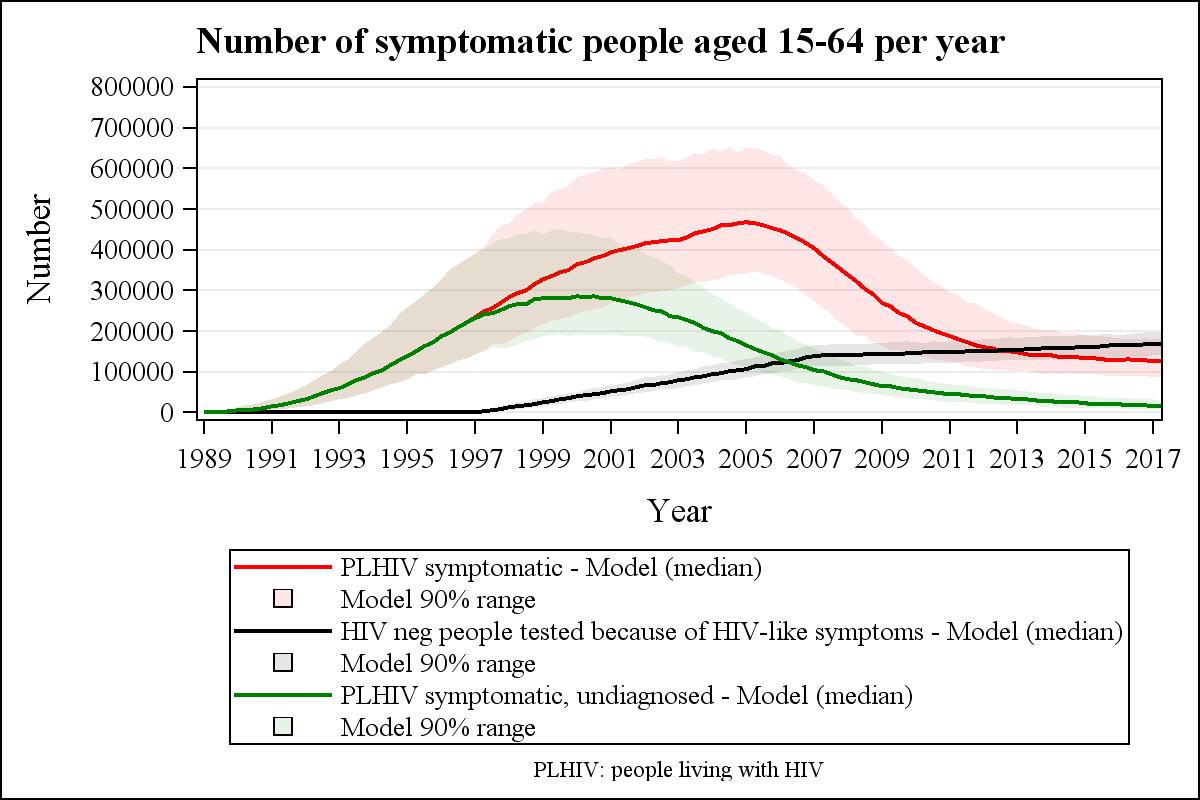


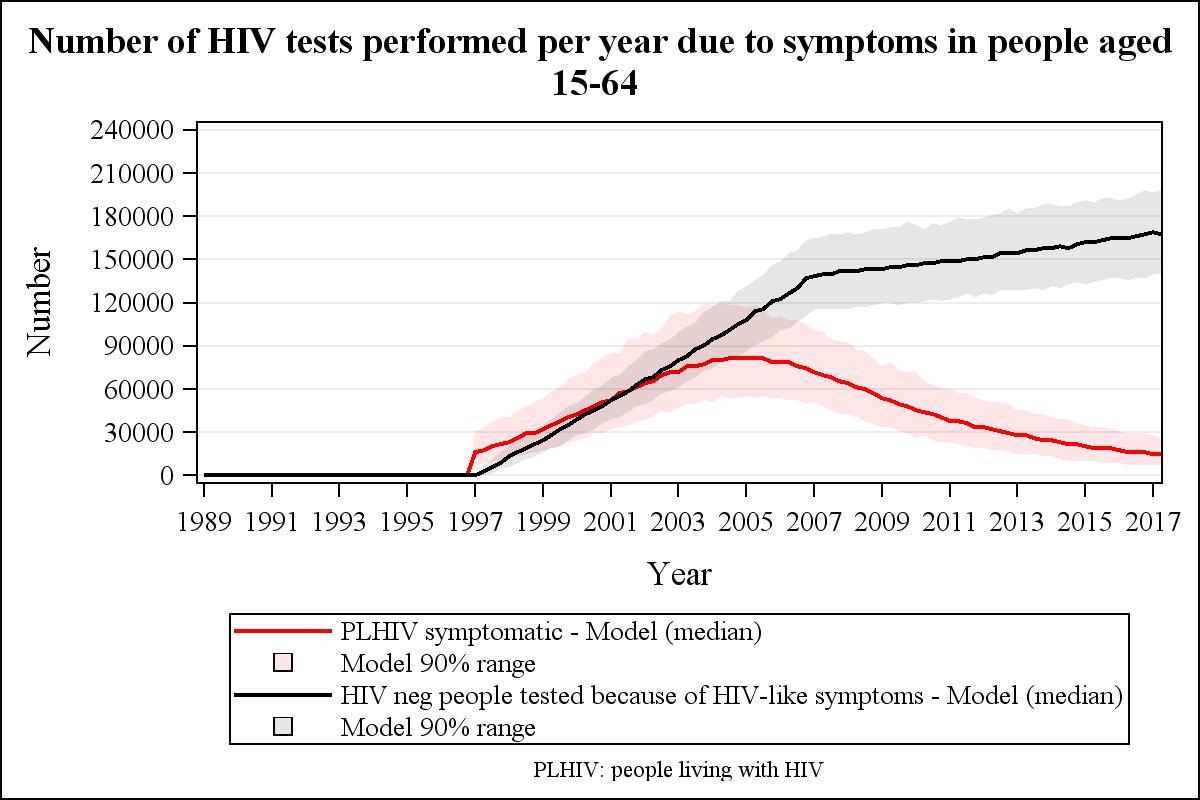


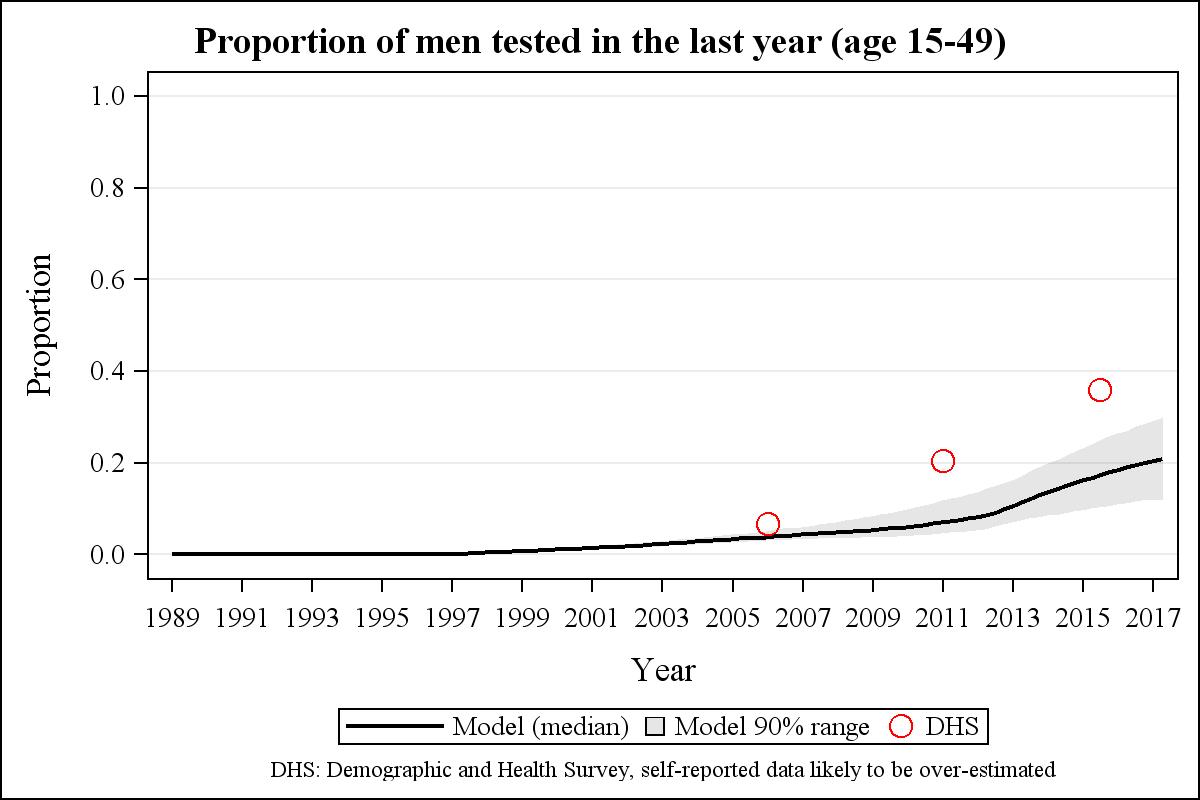


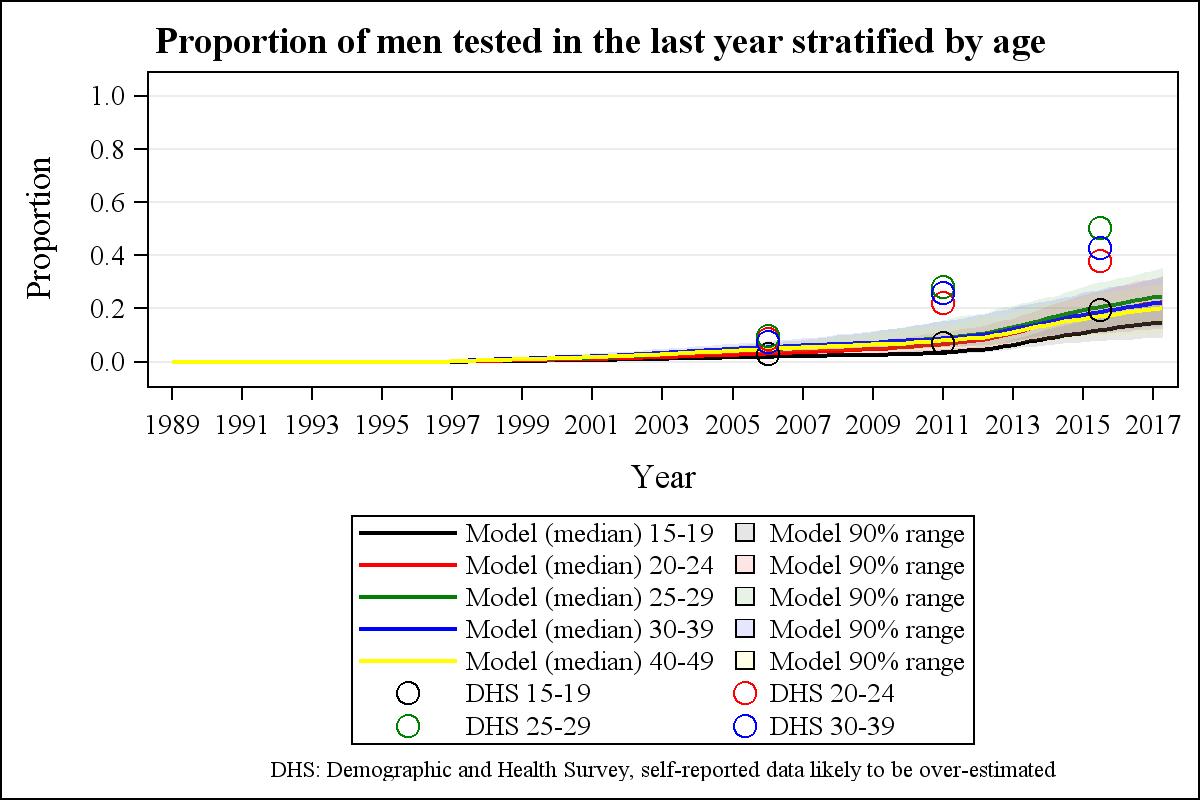


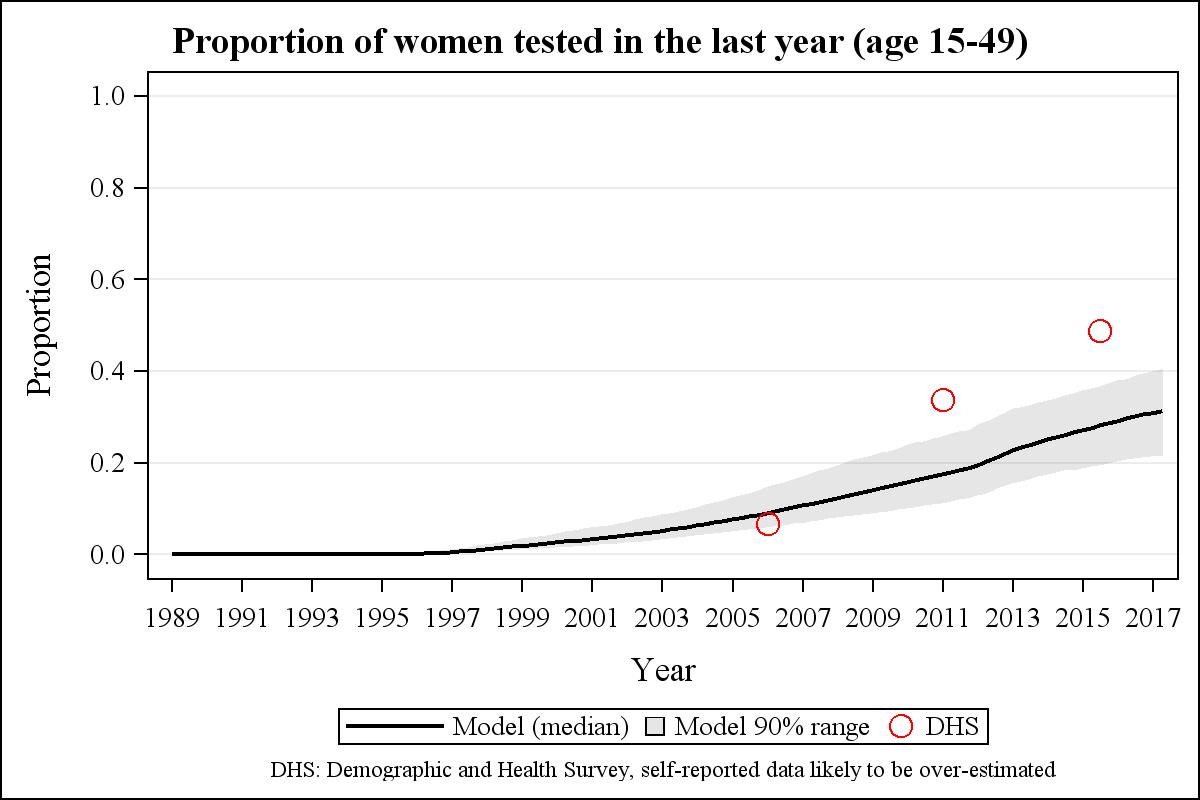


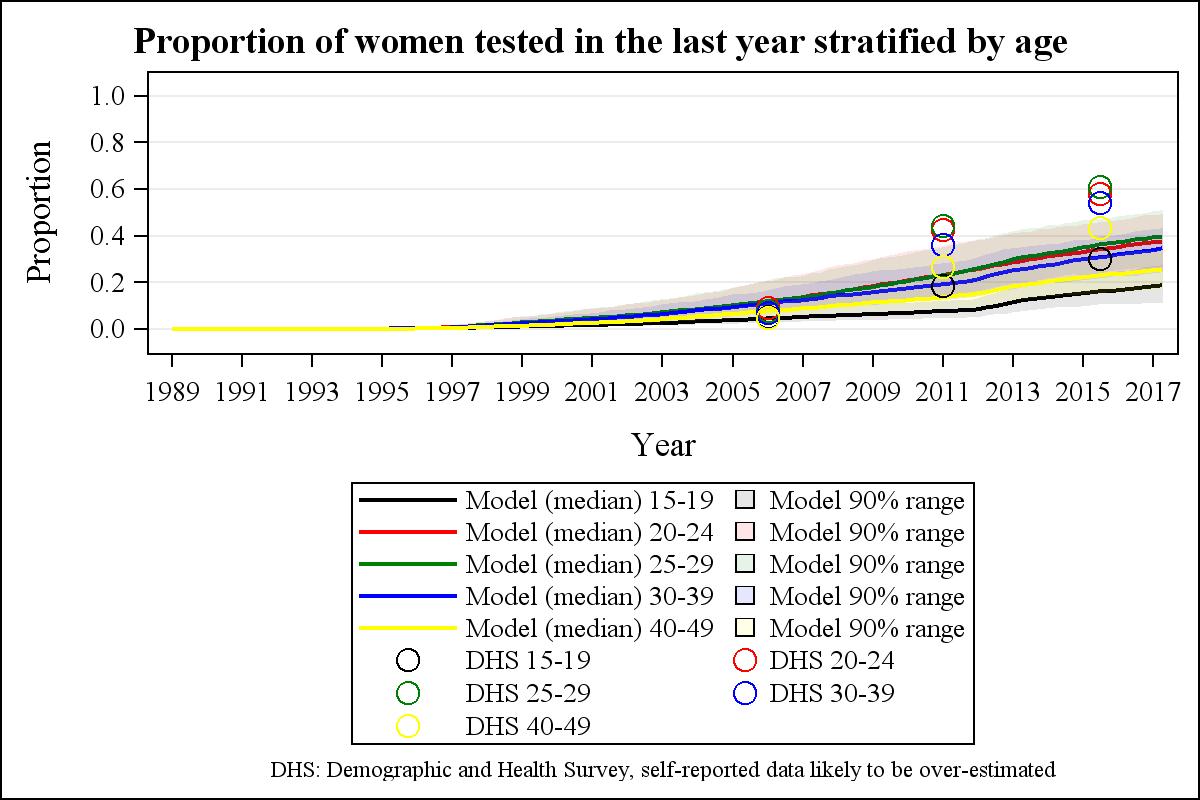


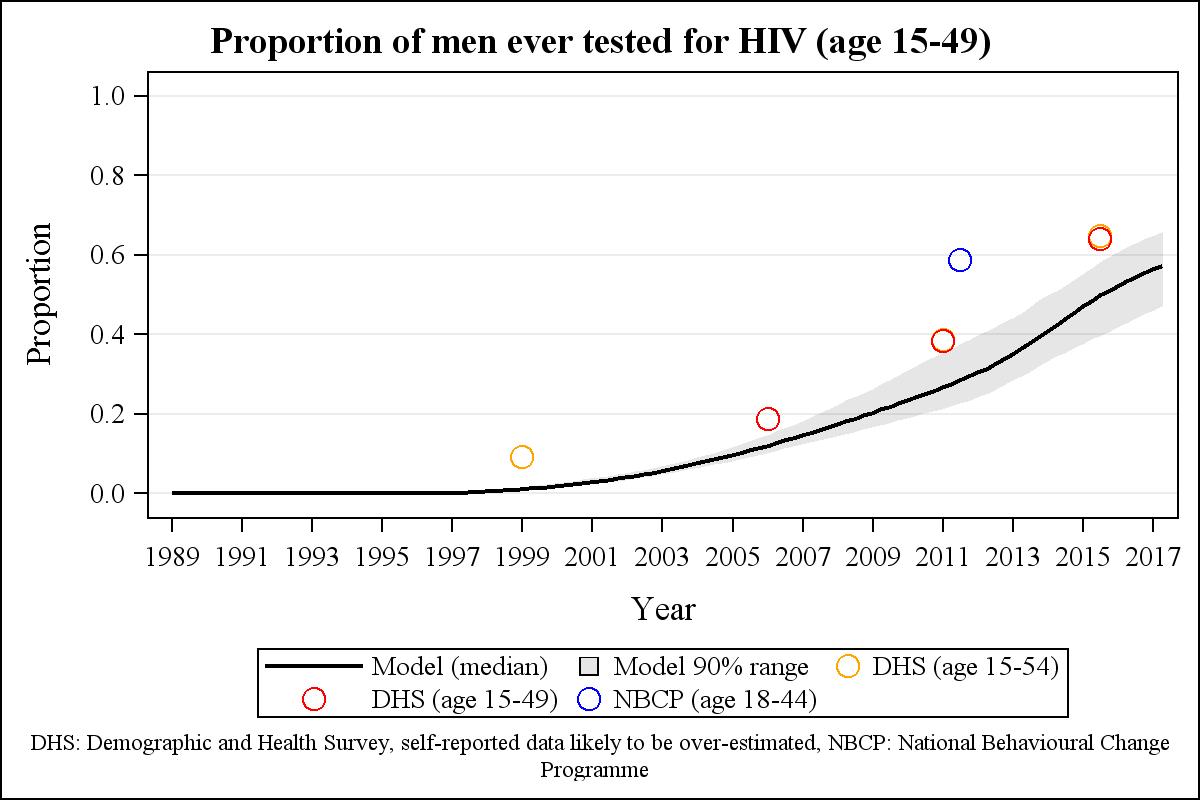


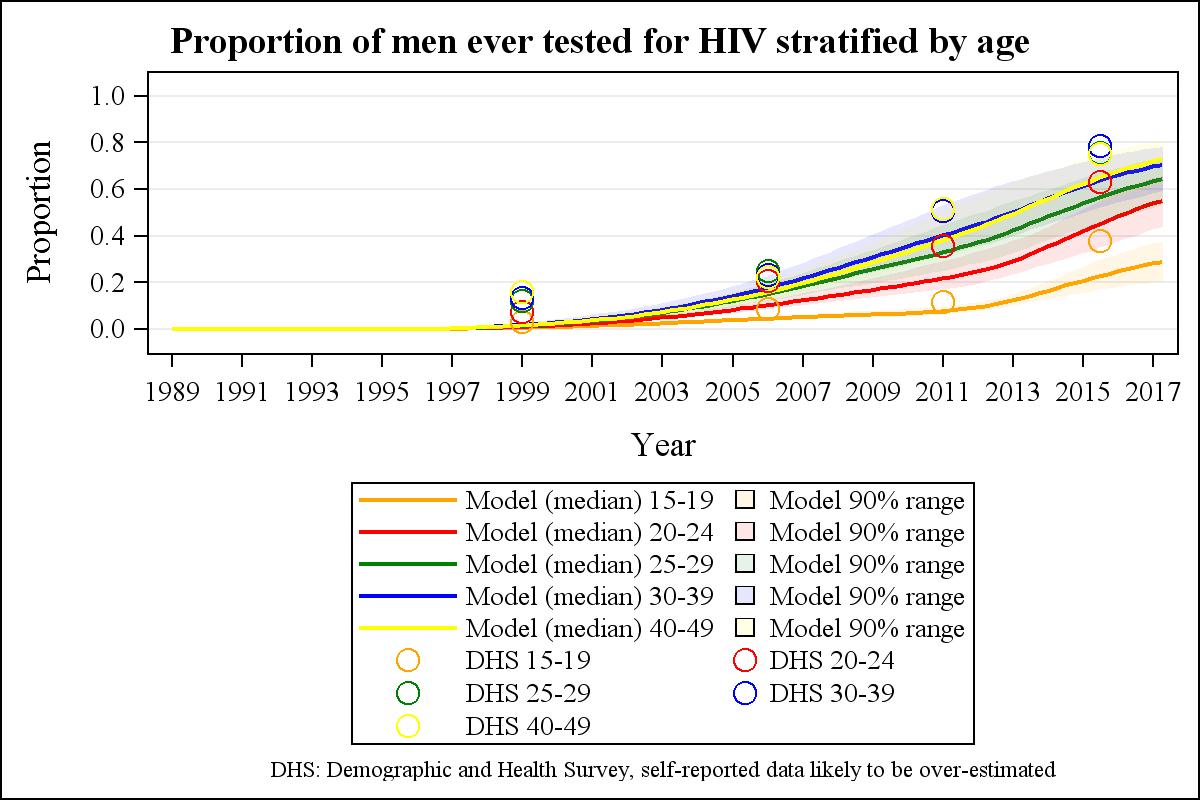


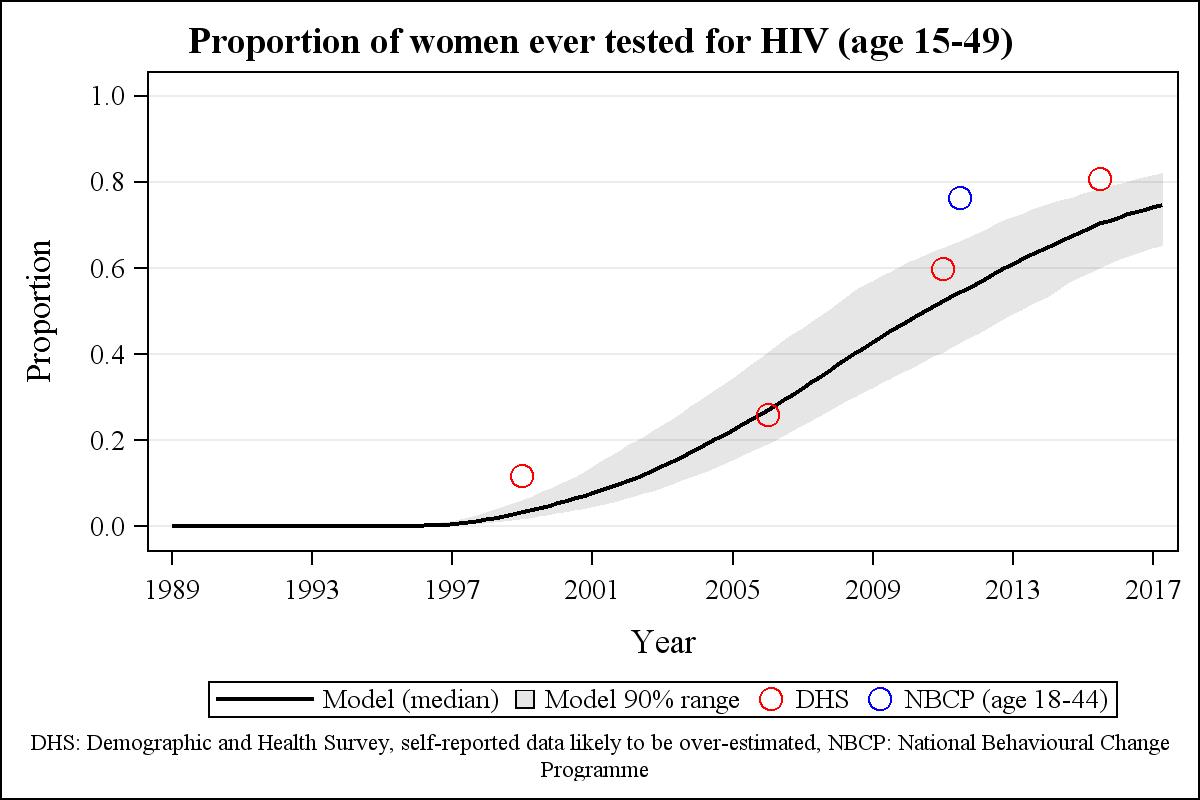


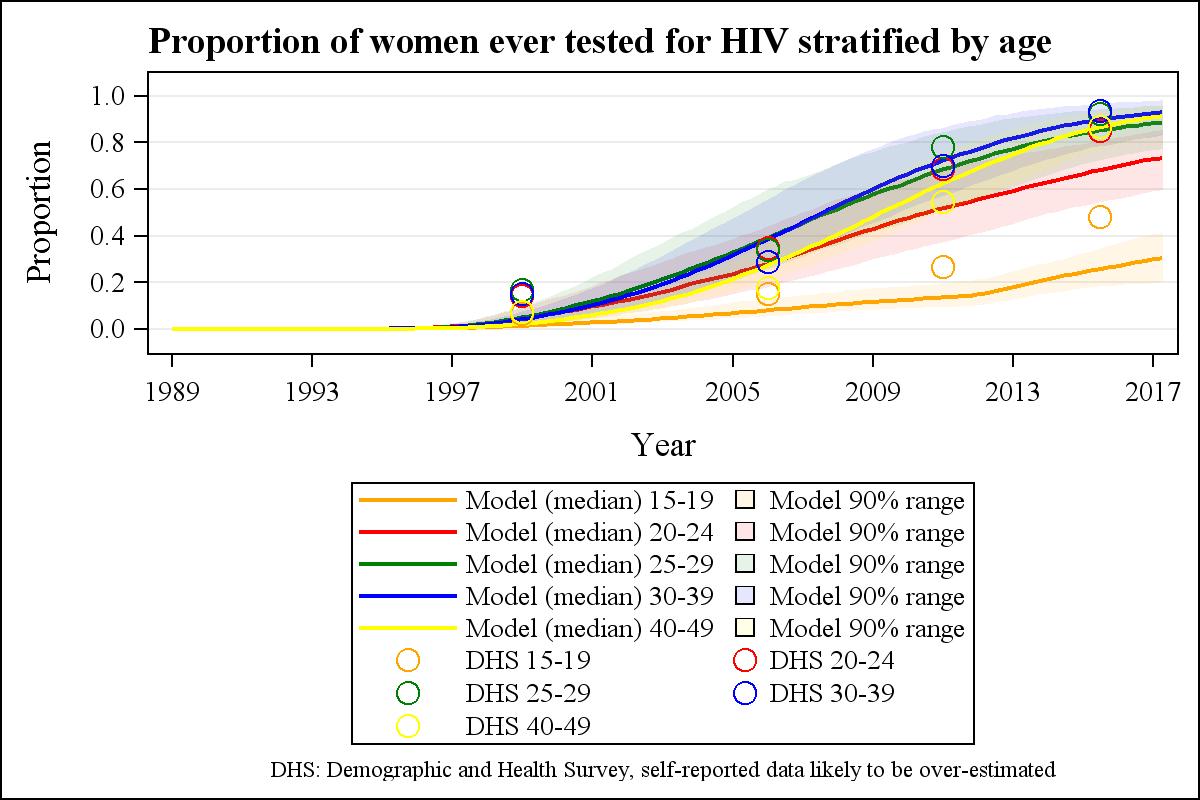


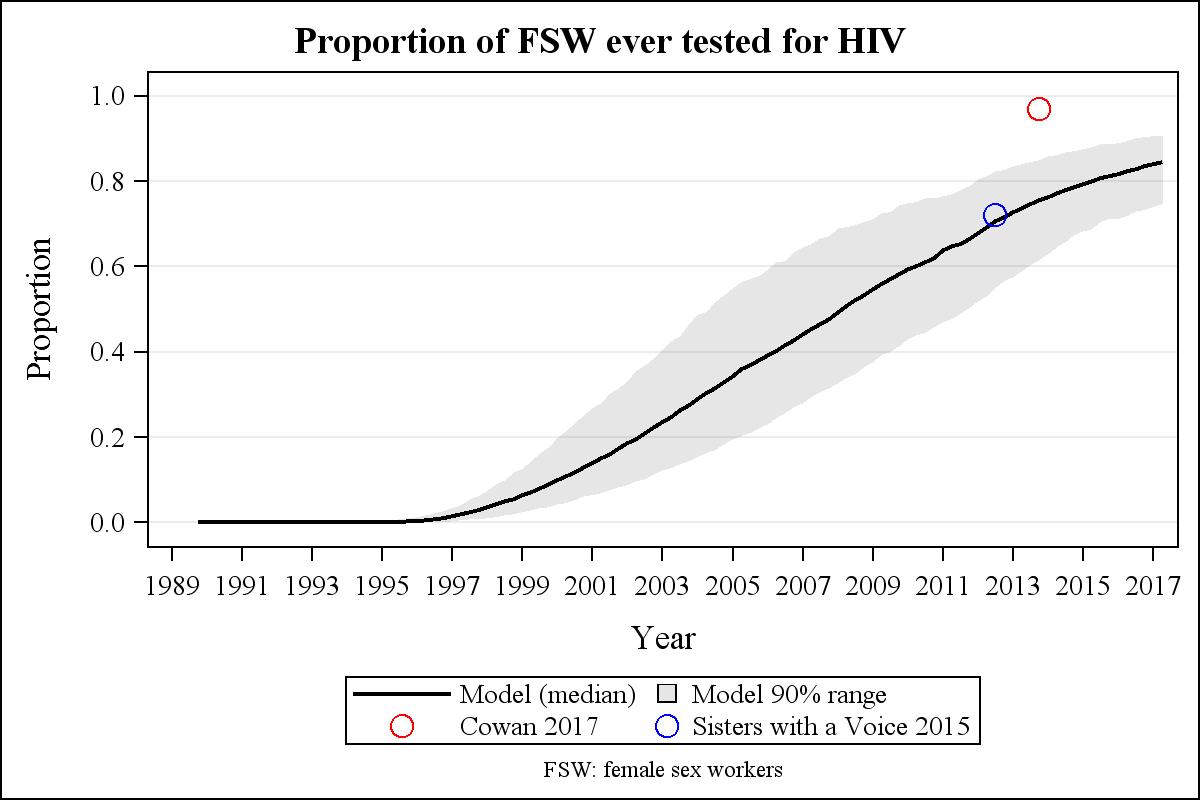


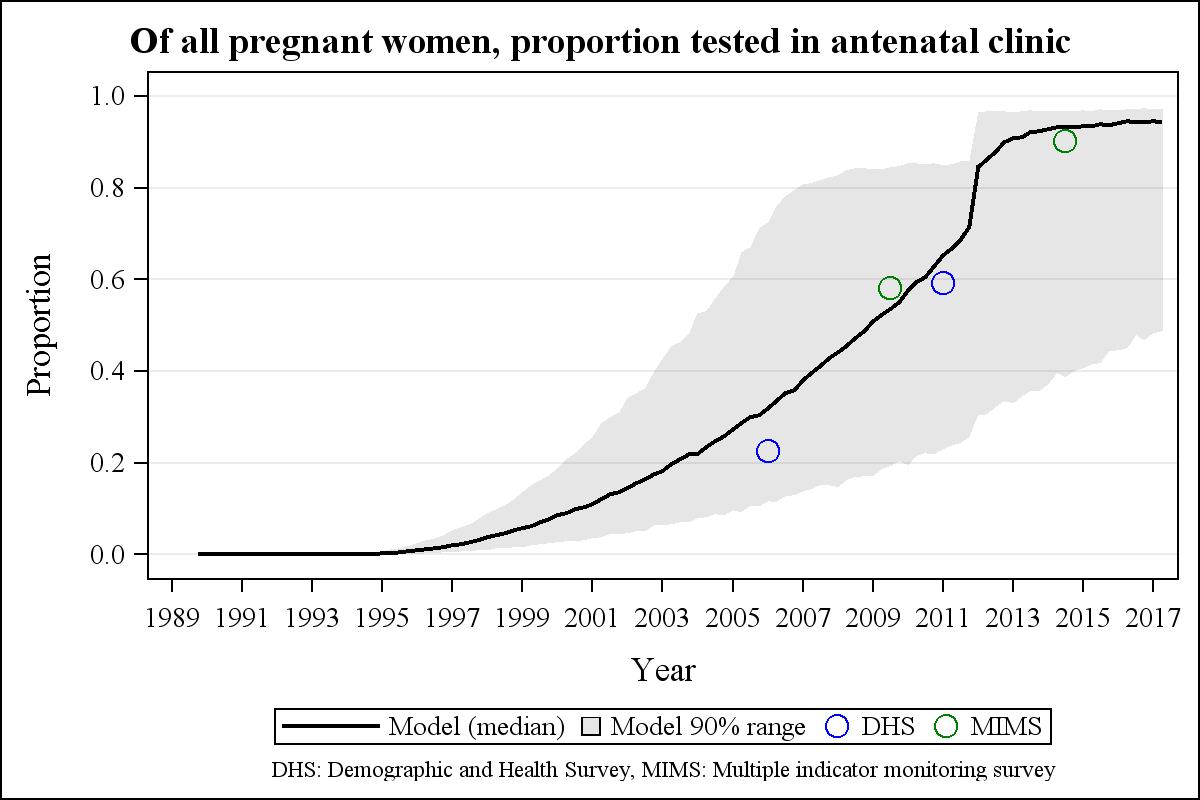


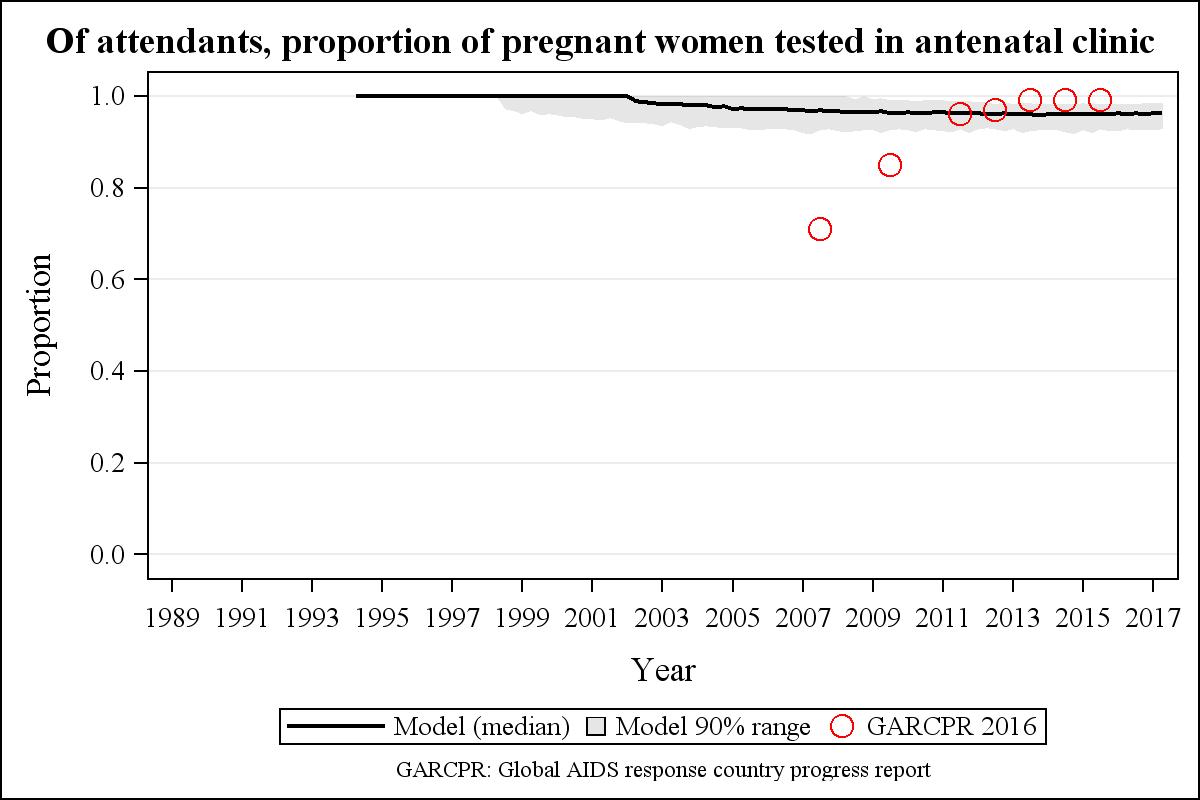


***HIV care, including diagnosis***


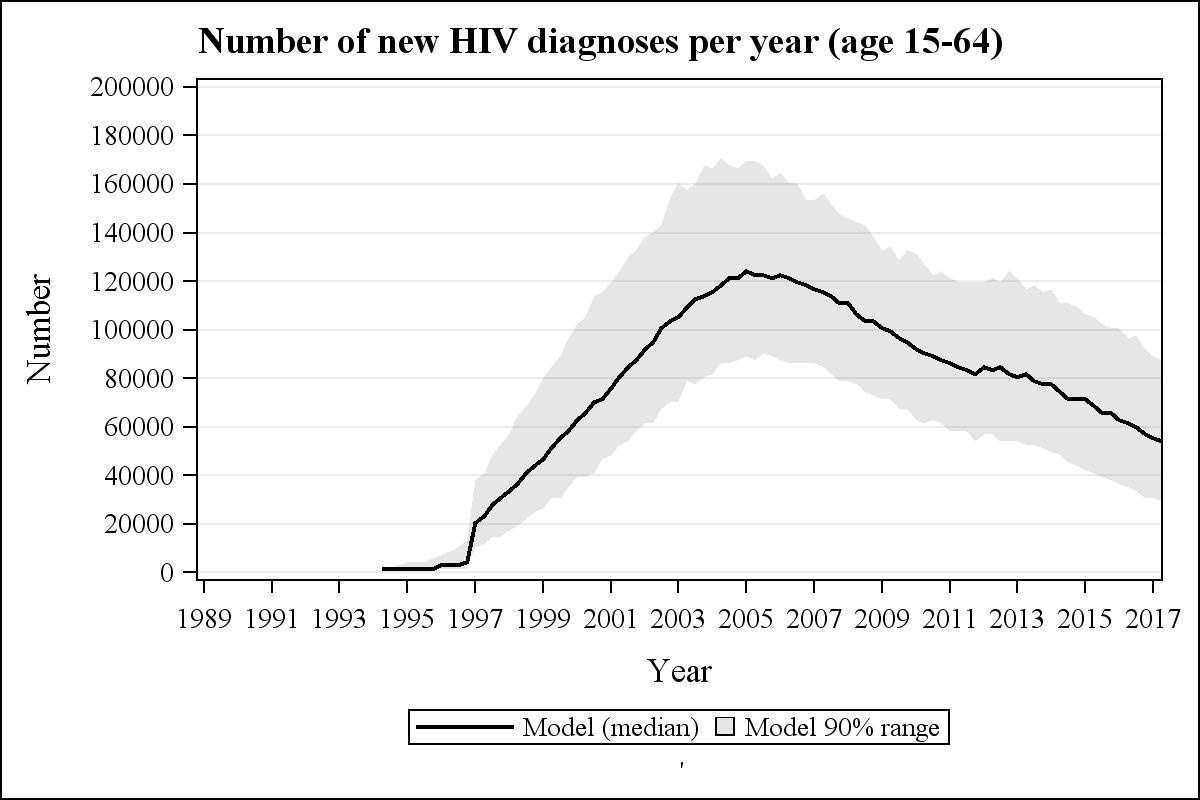


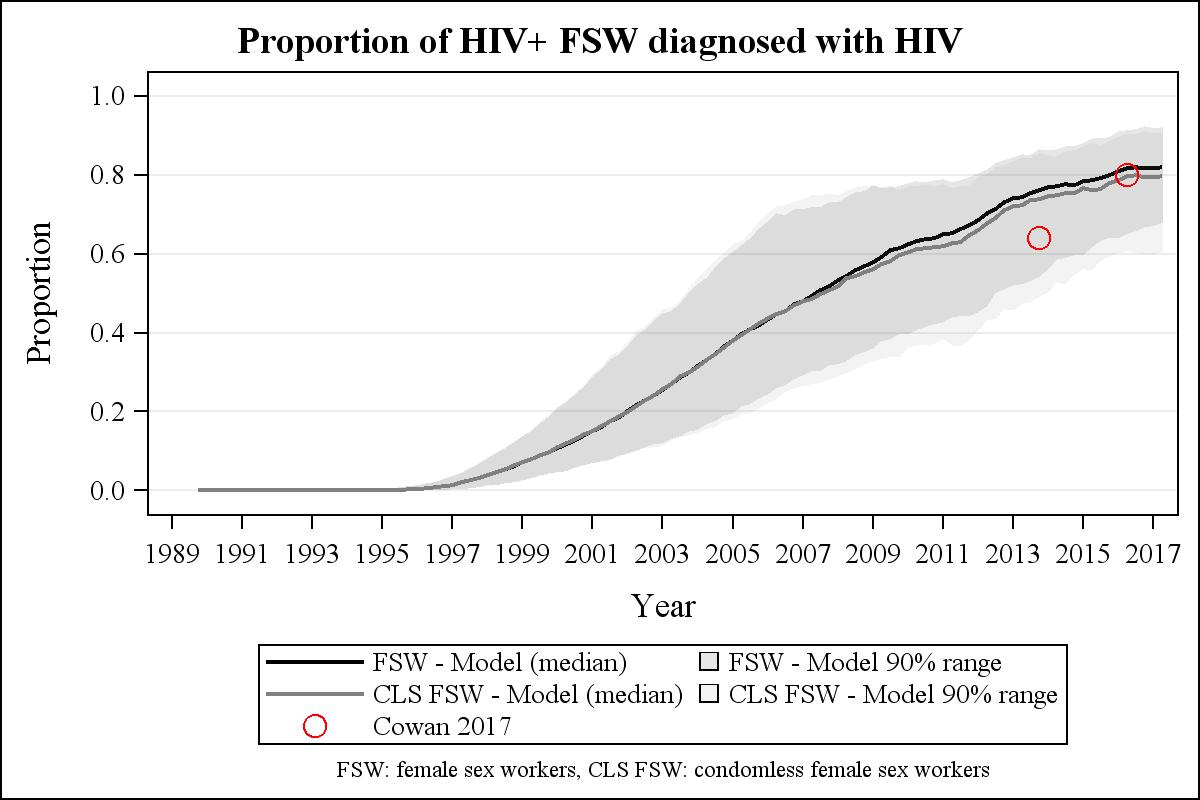


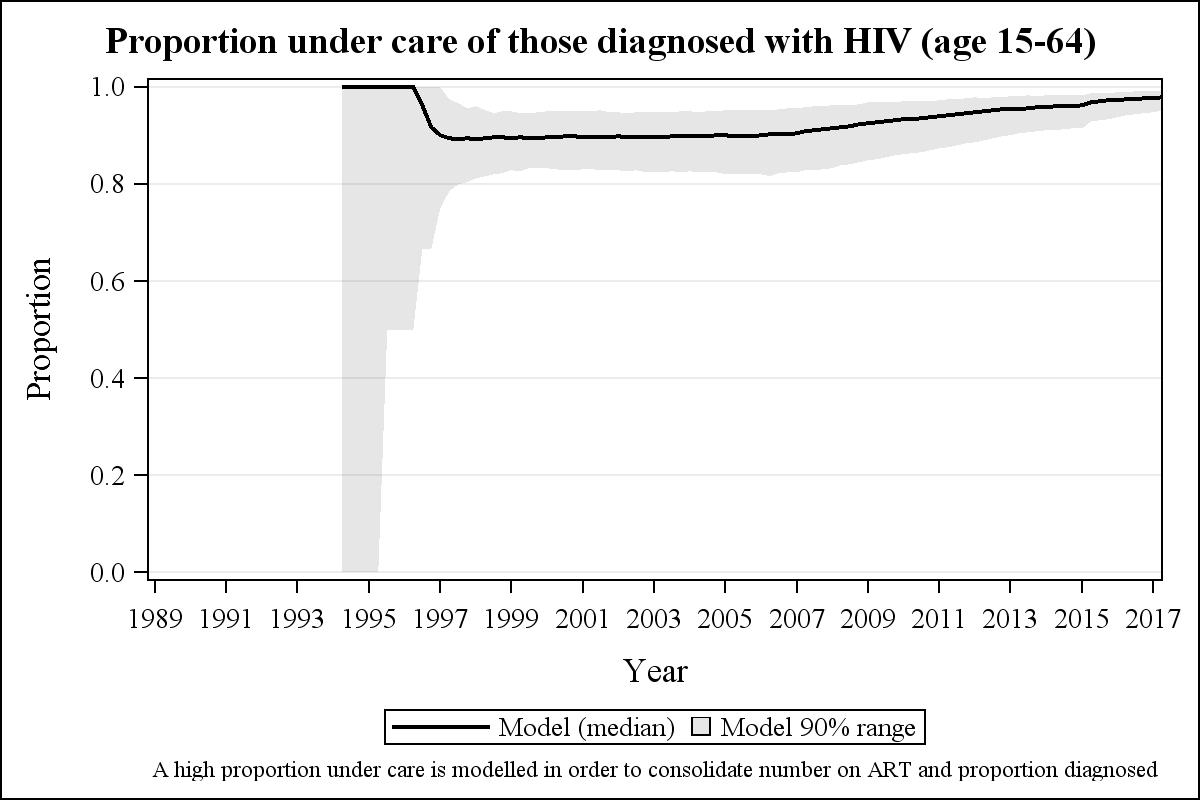


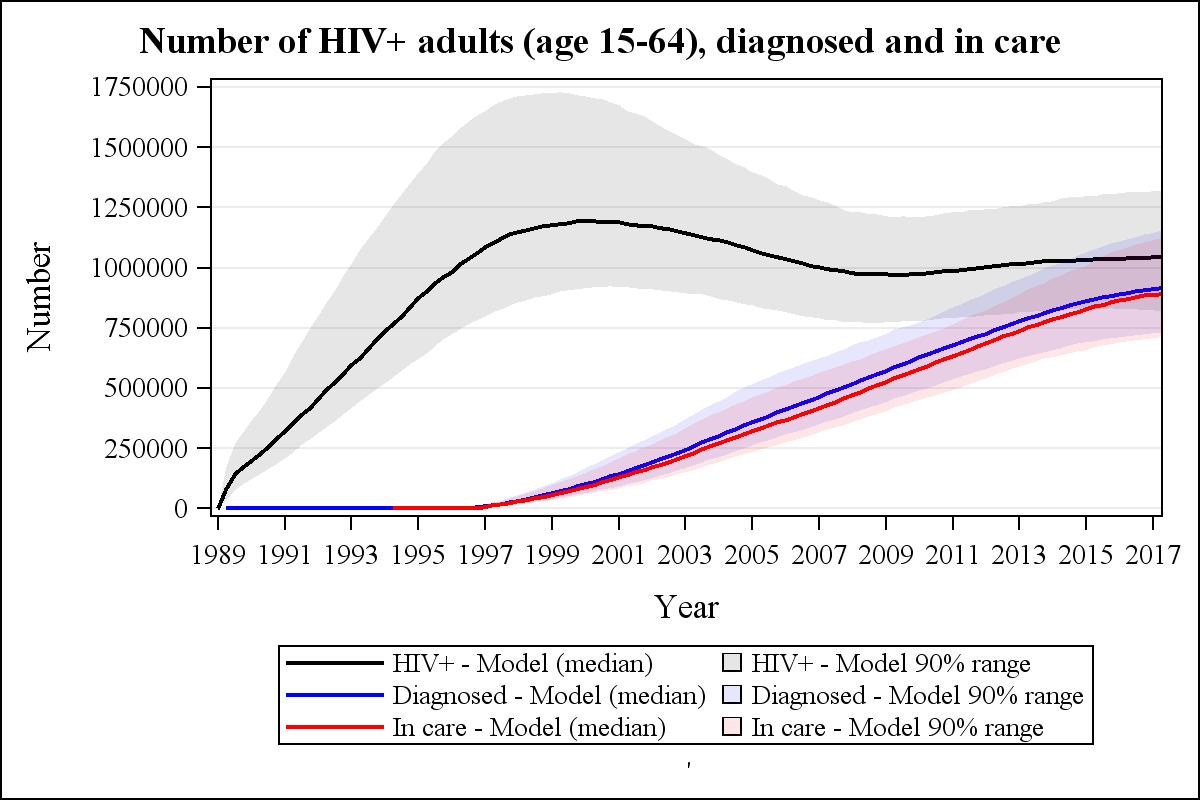


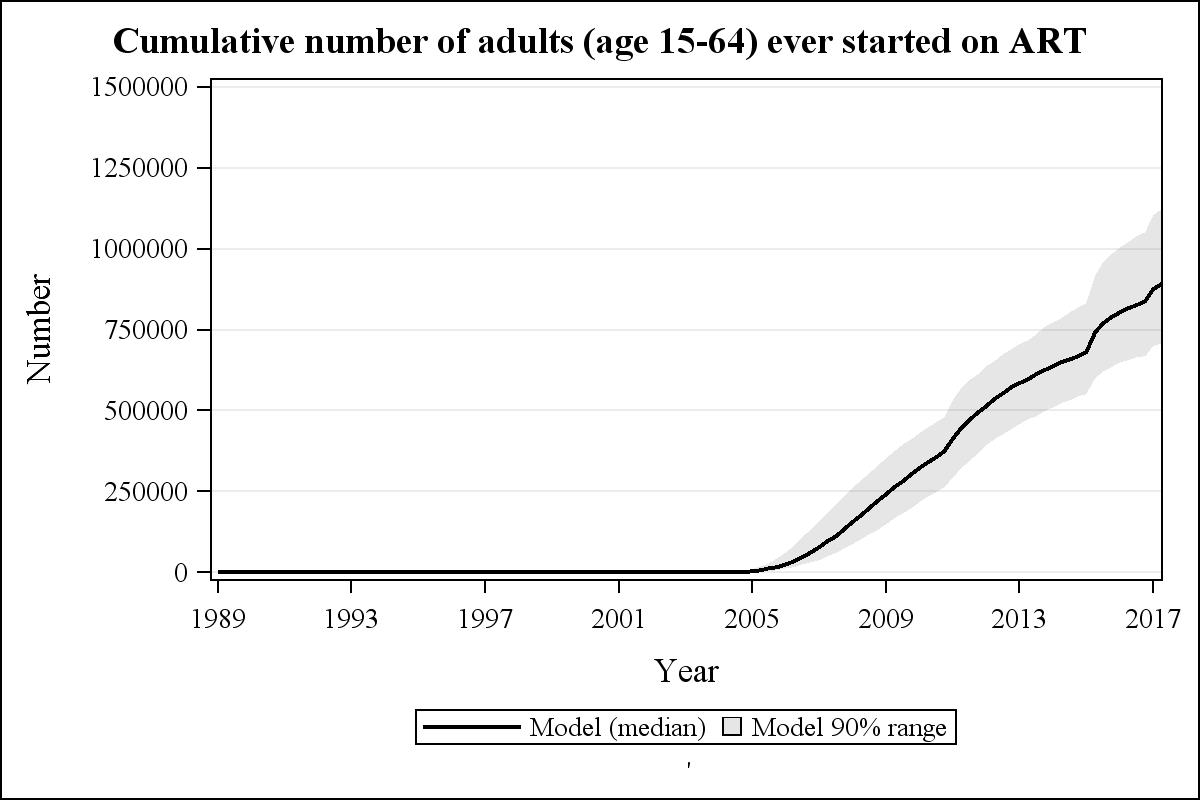


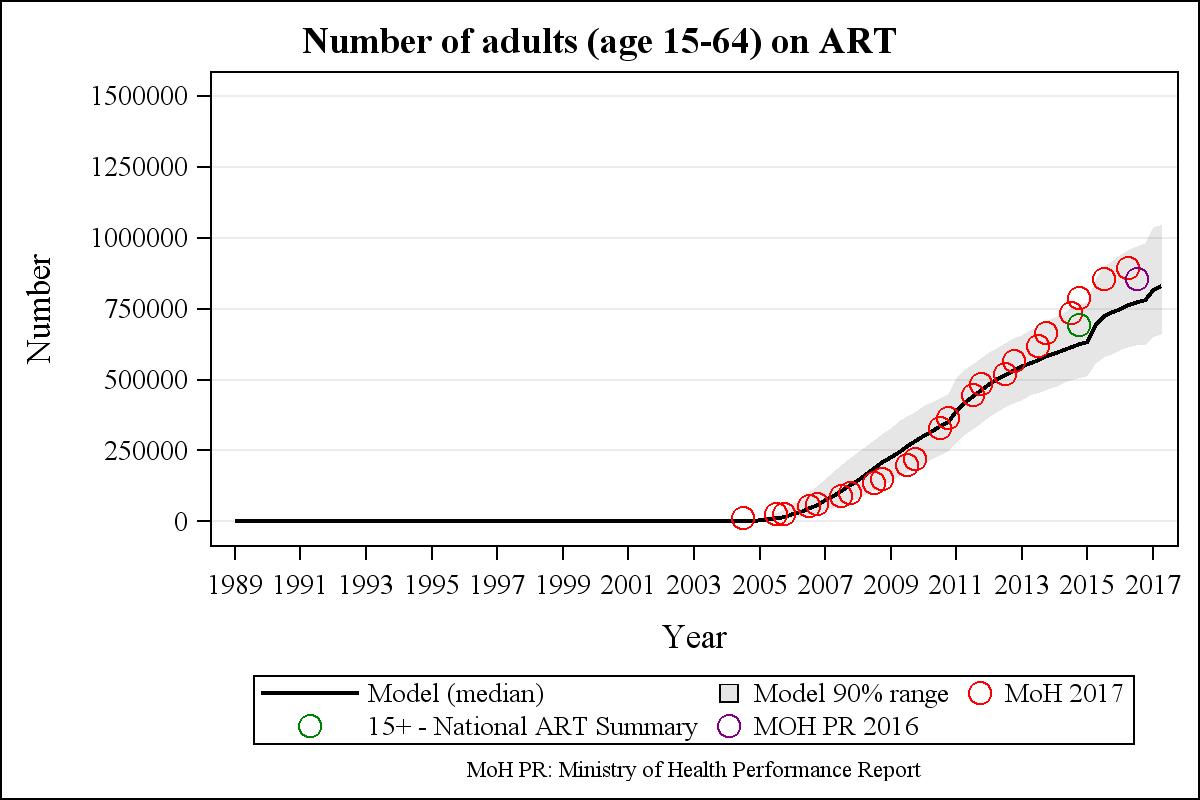


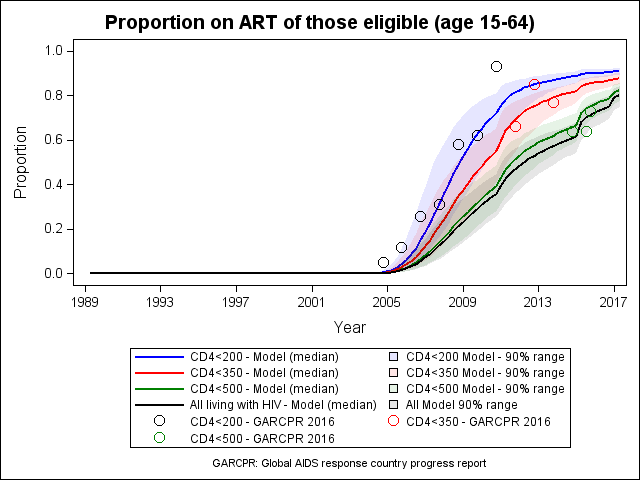


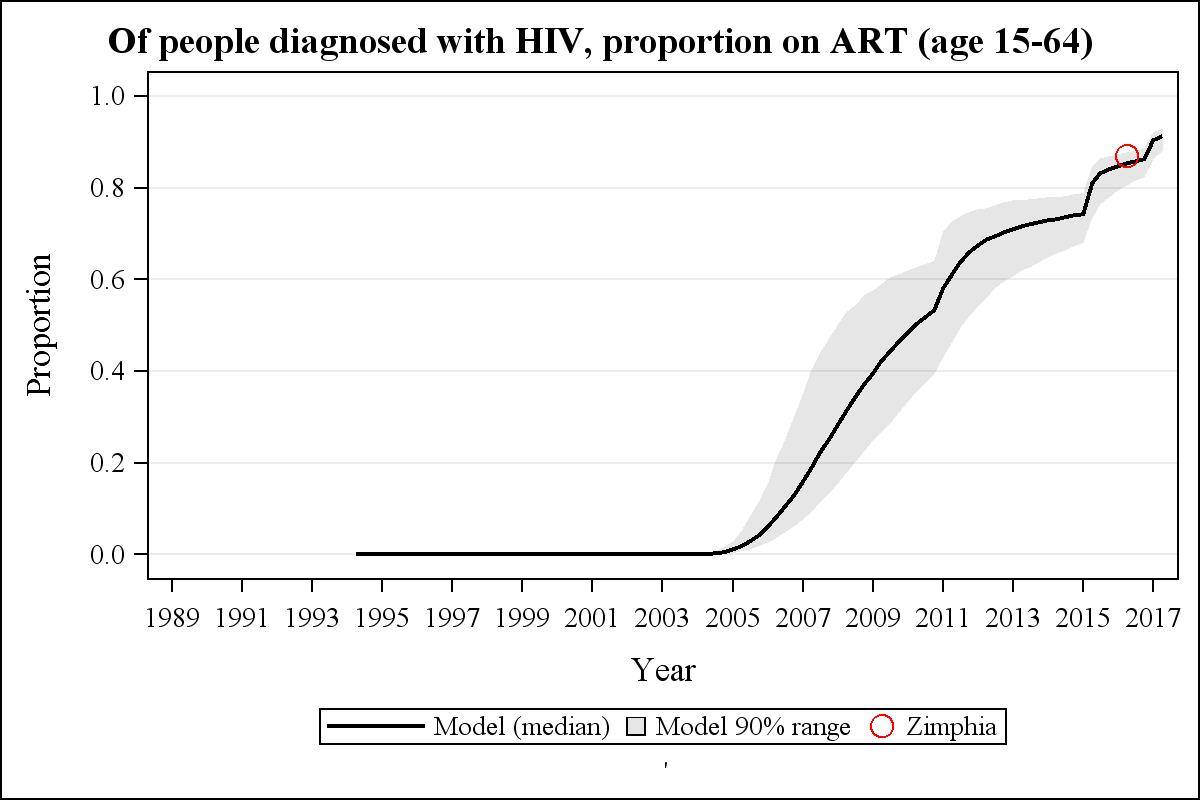


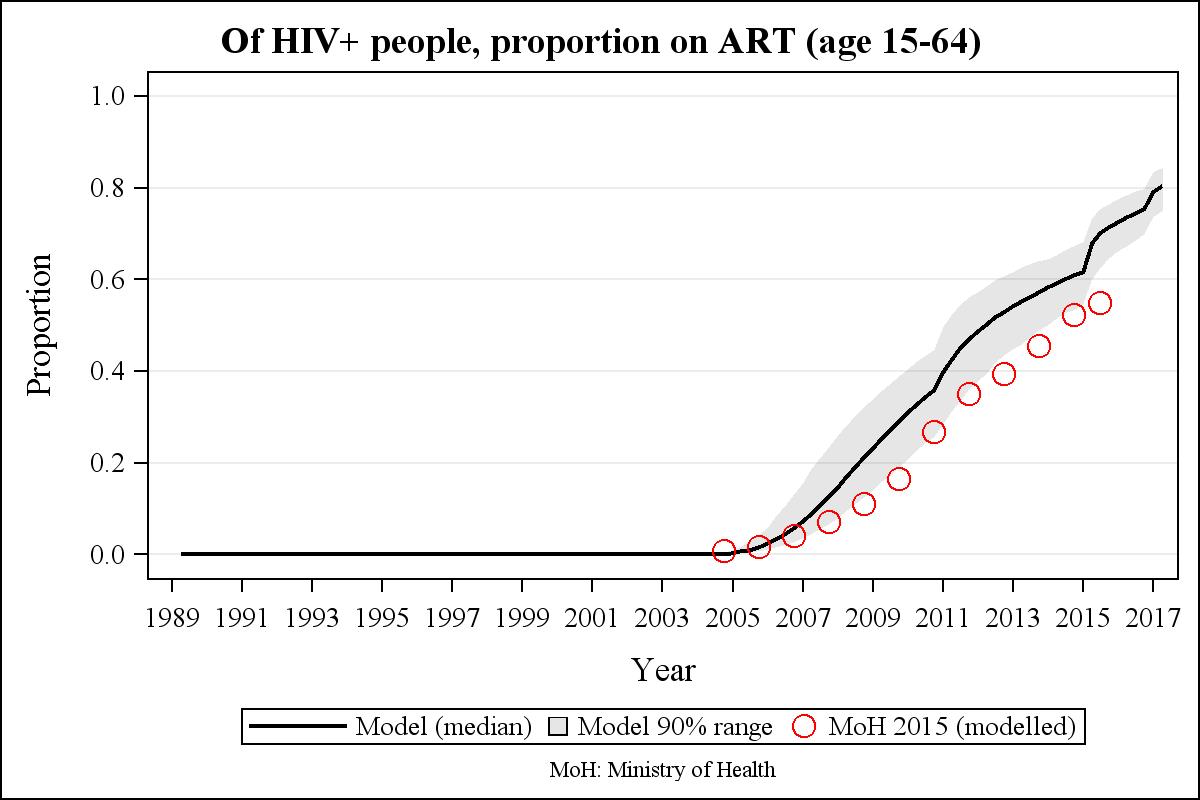


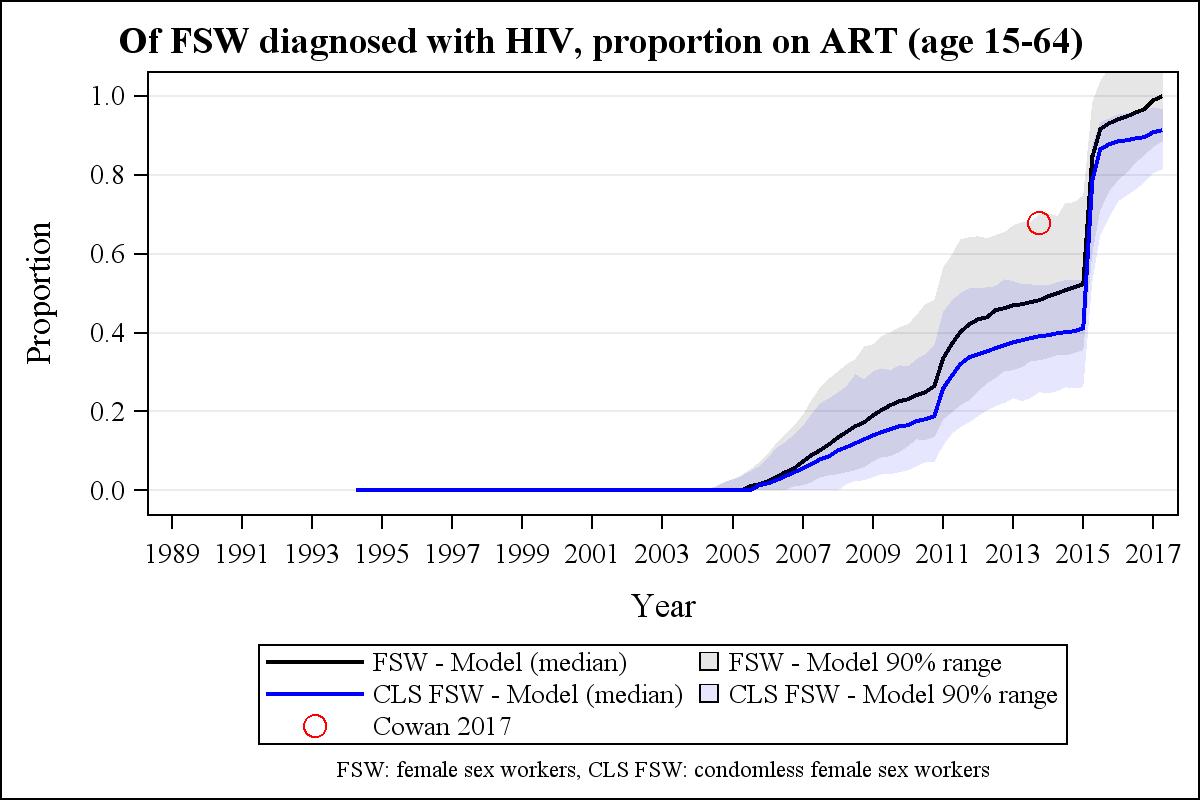


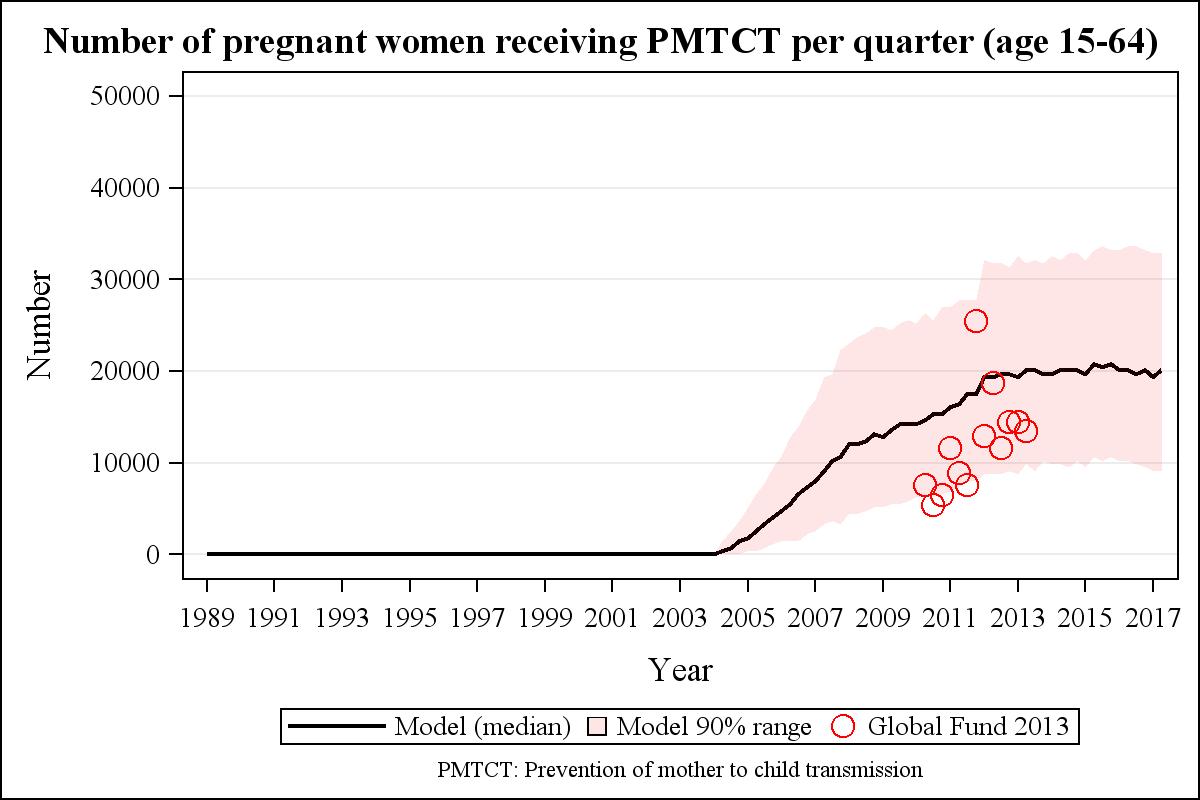


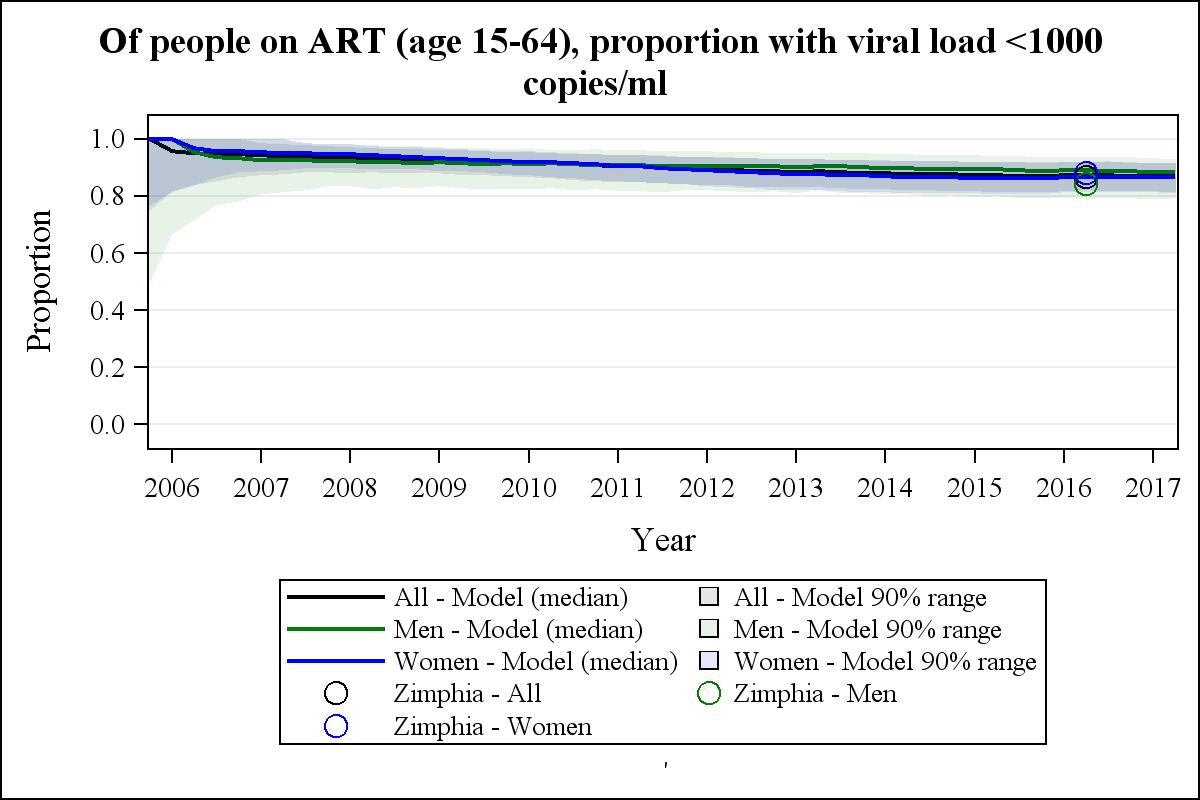


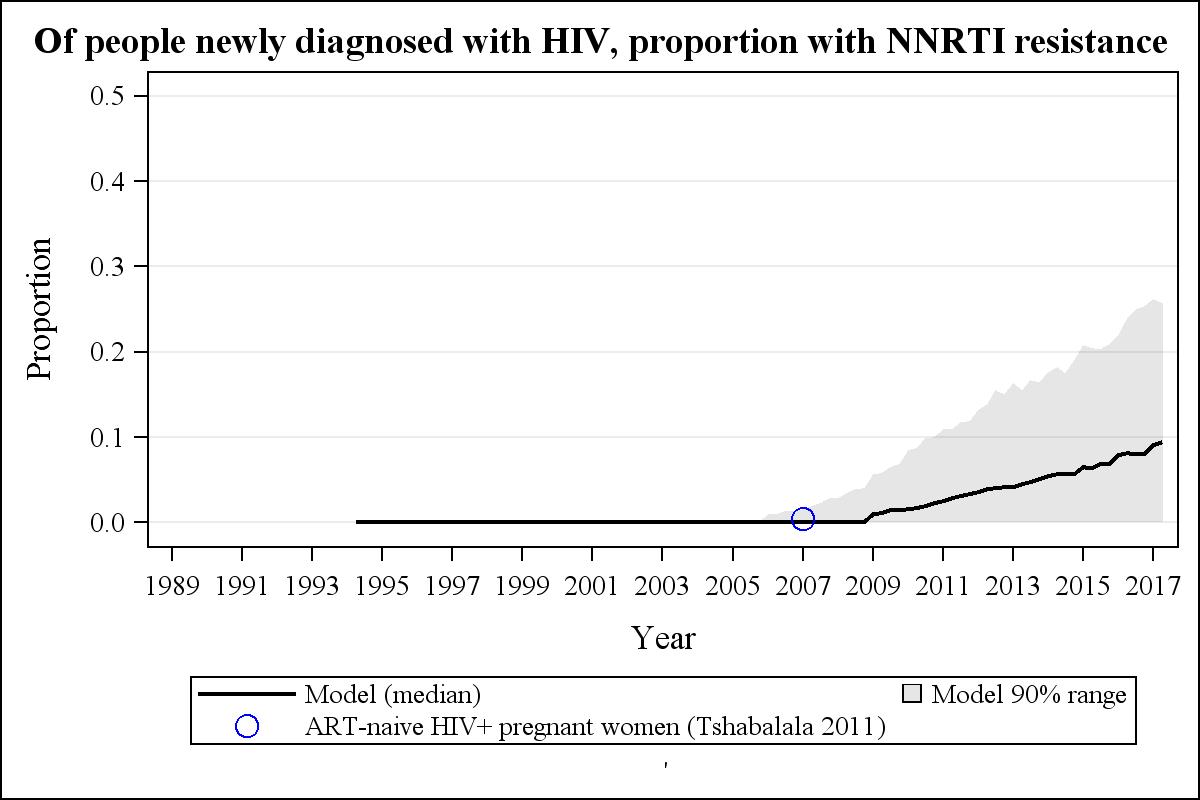


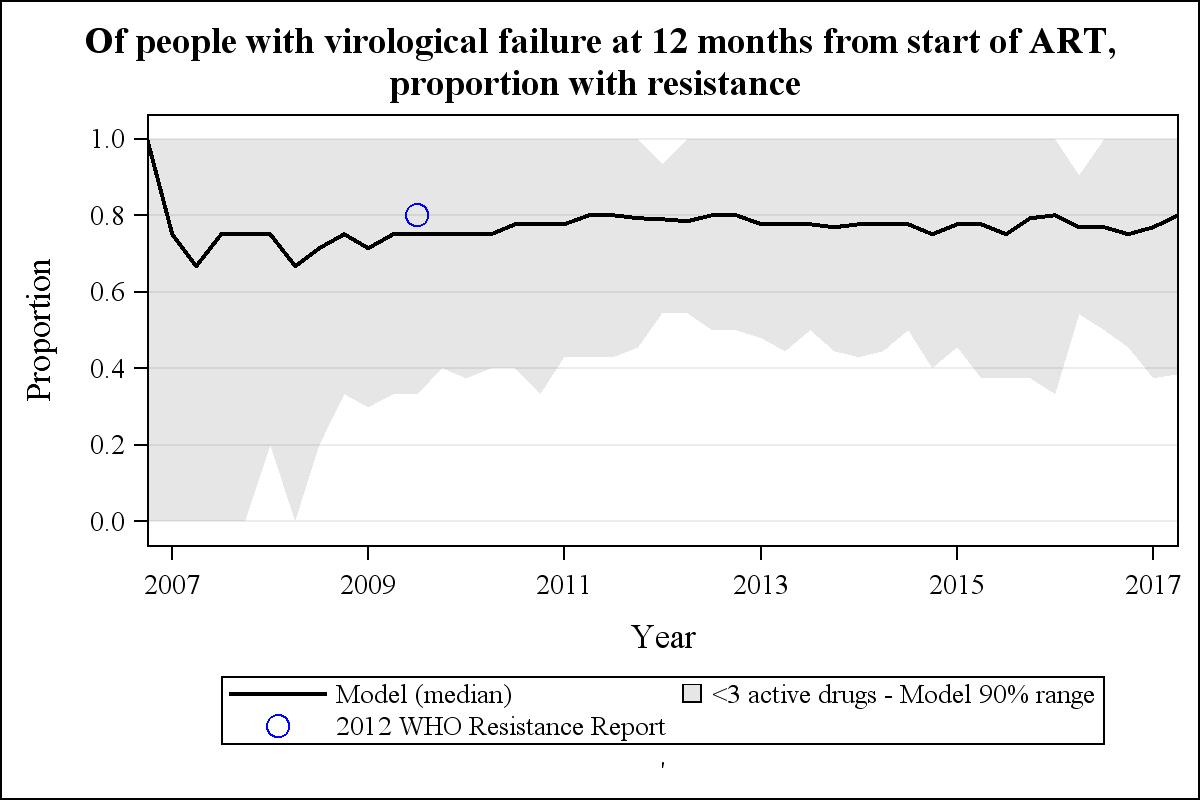


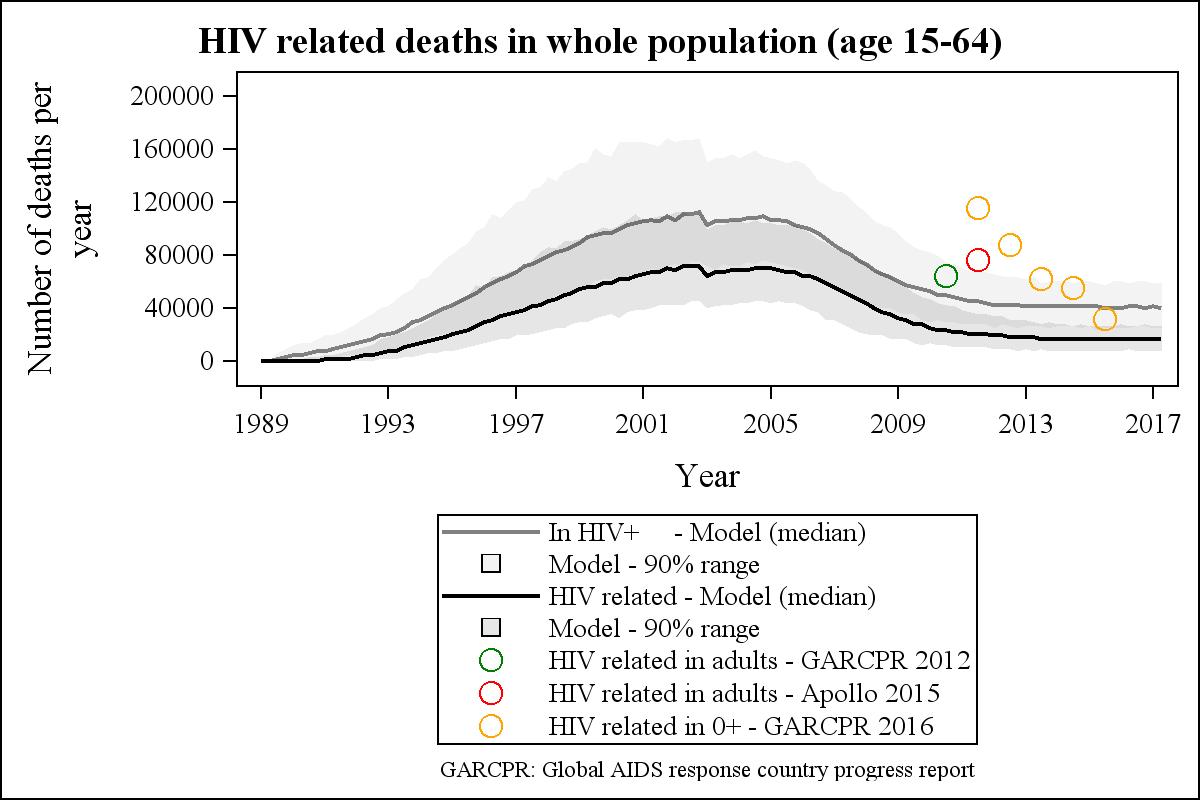


***Sexual behaviour***


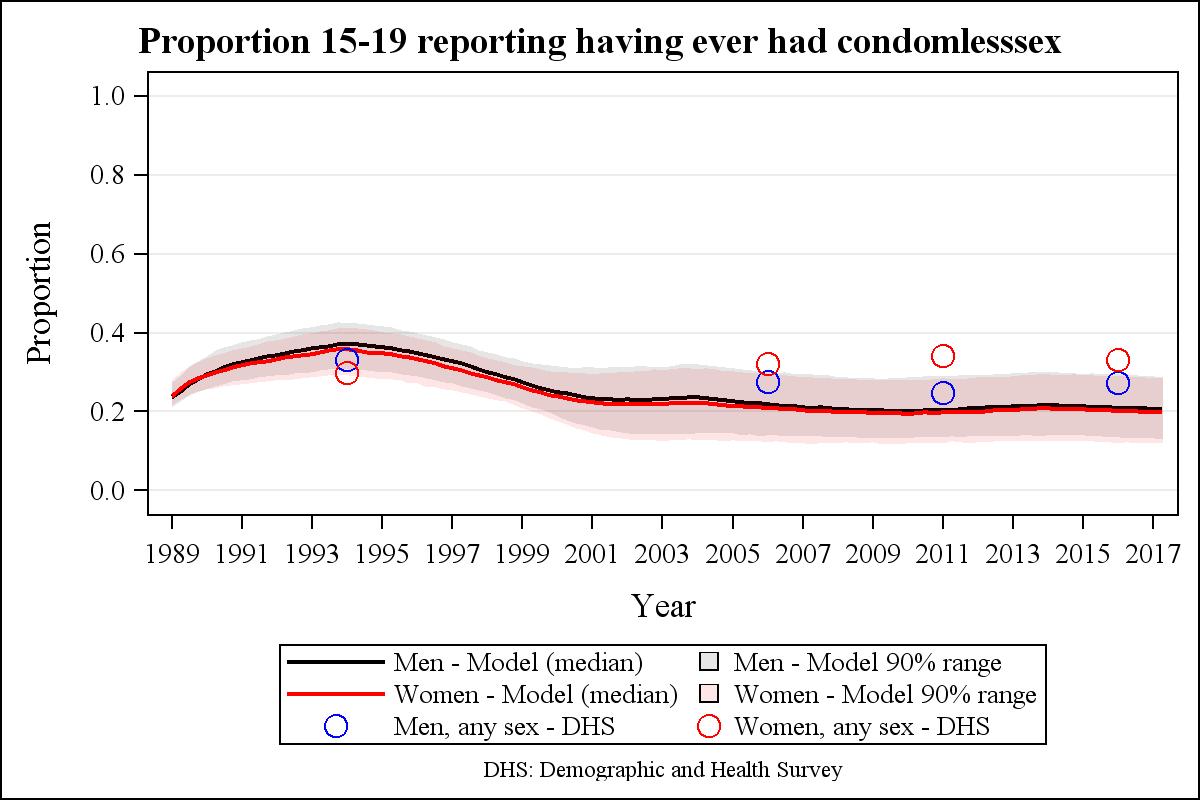


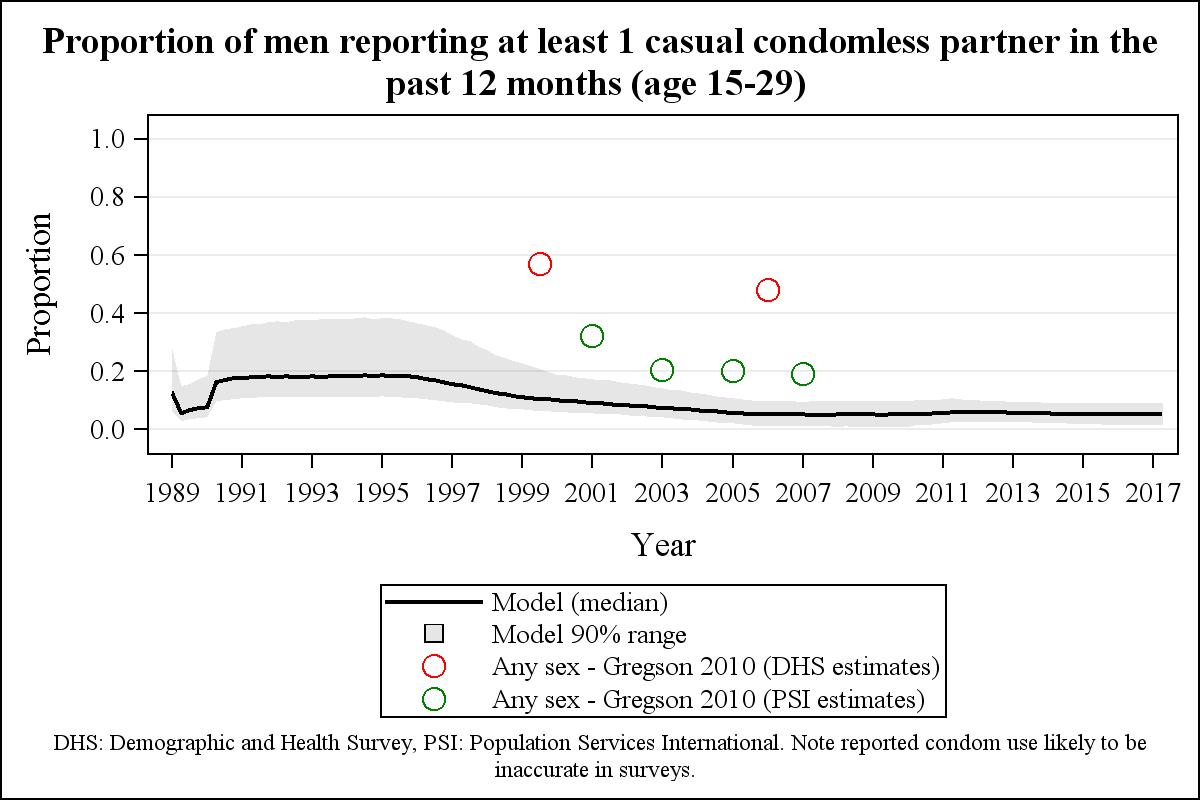


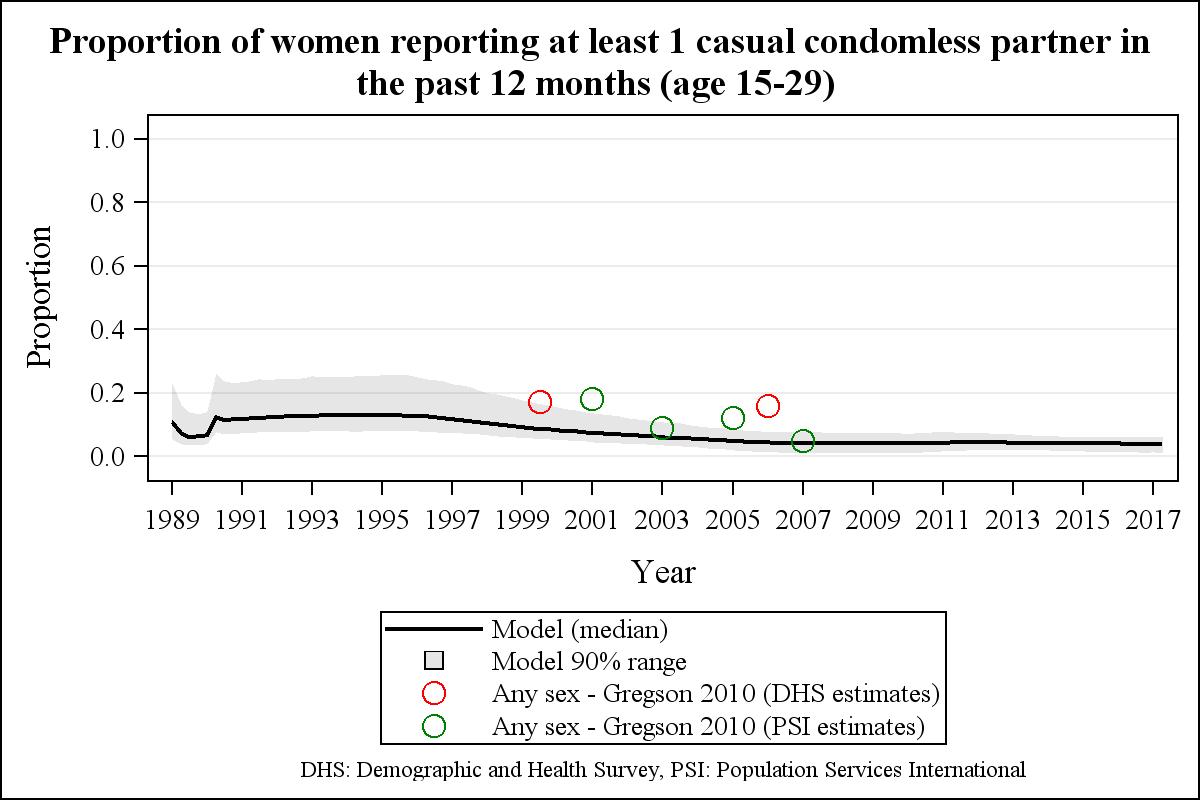


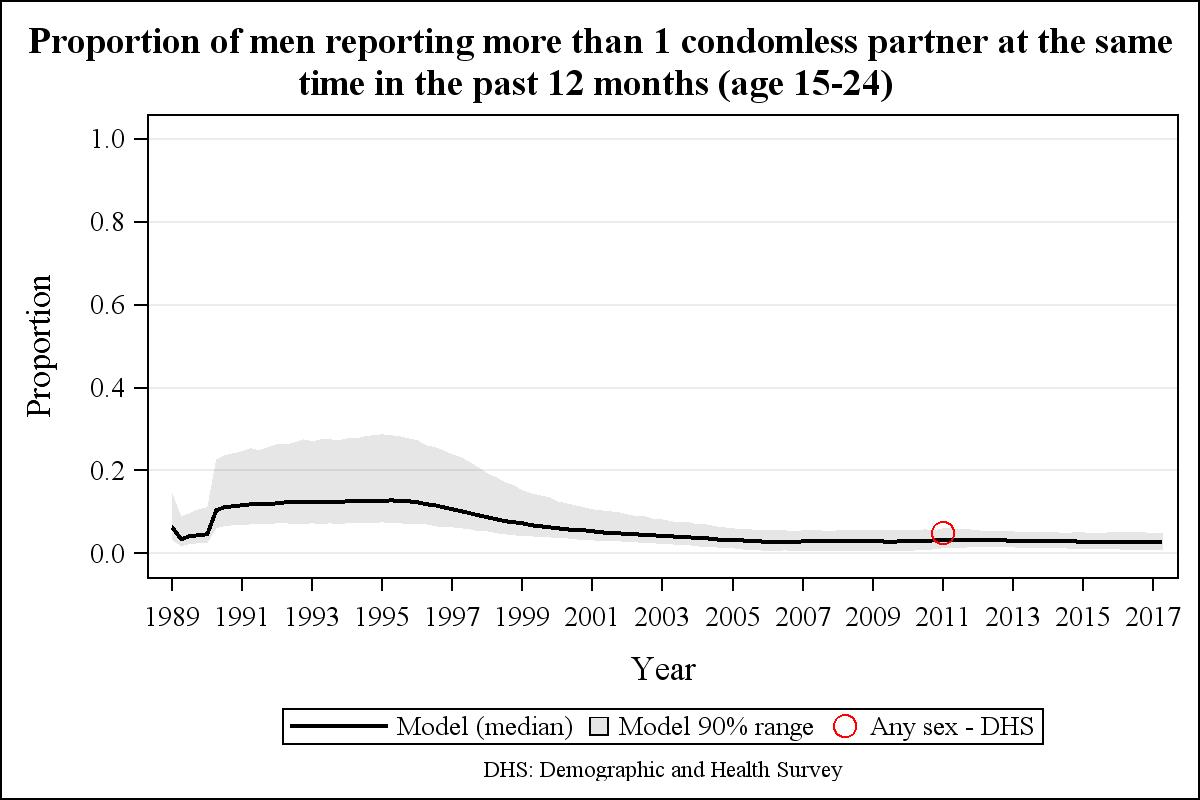


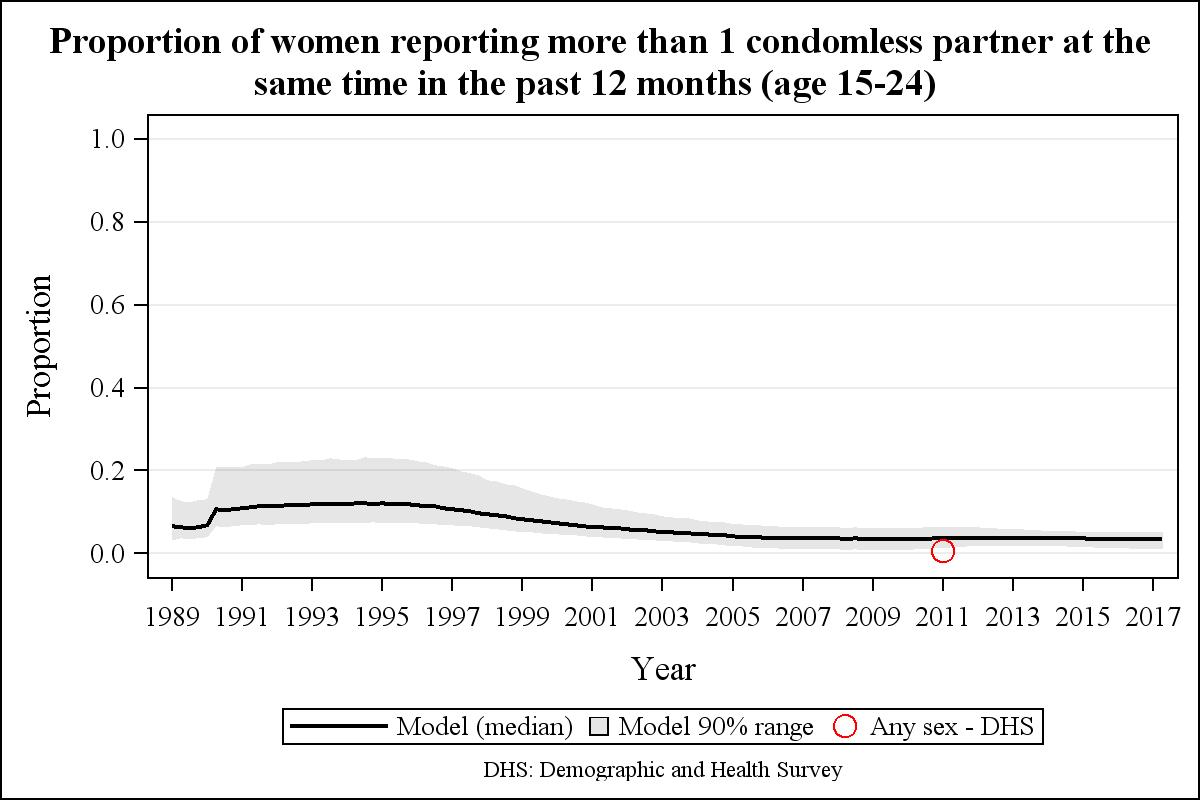


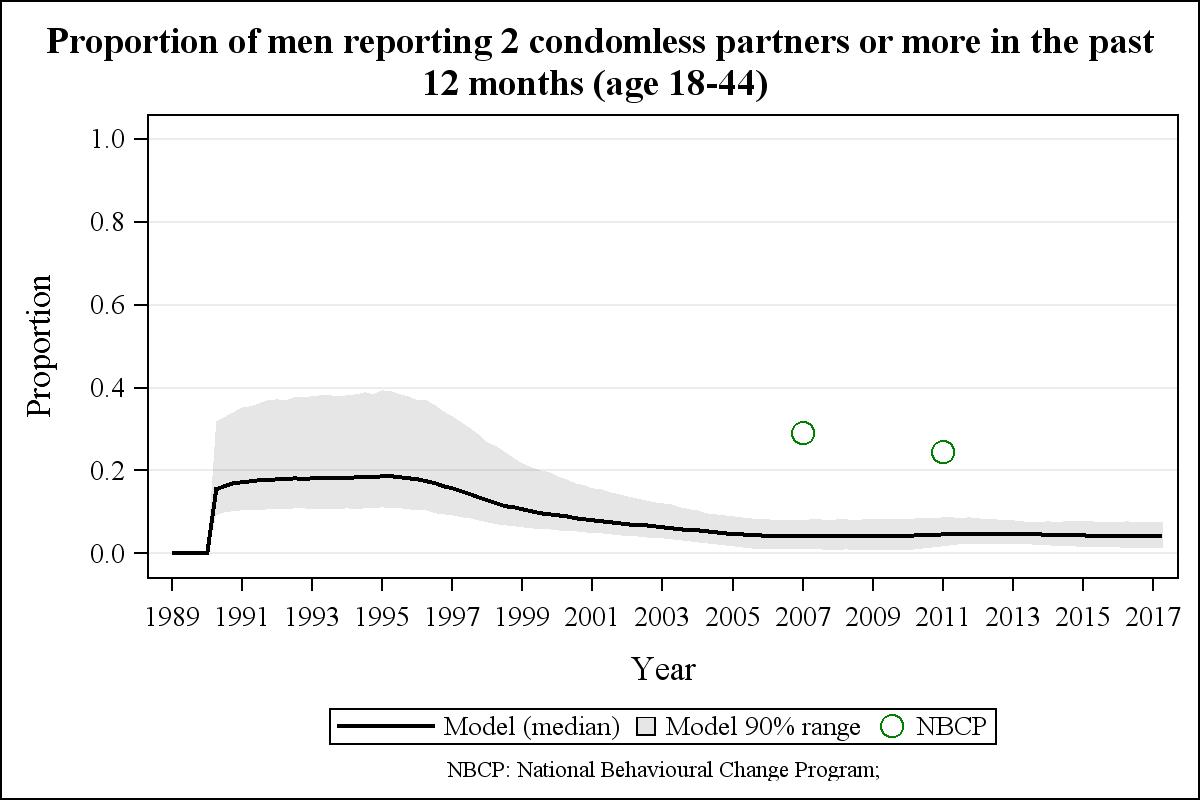


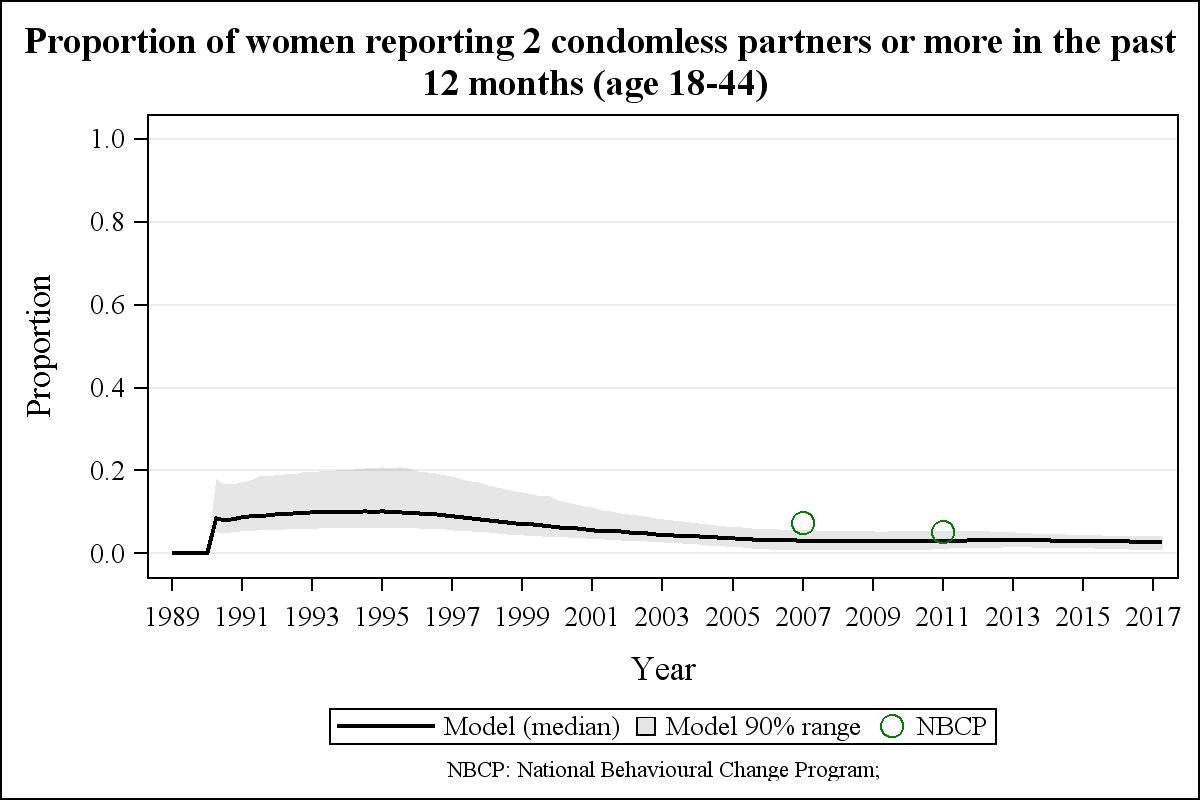


***Source of infection***


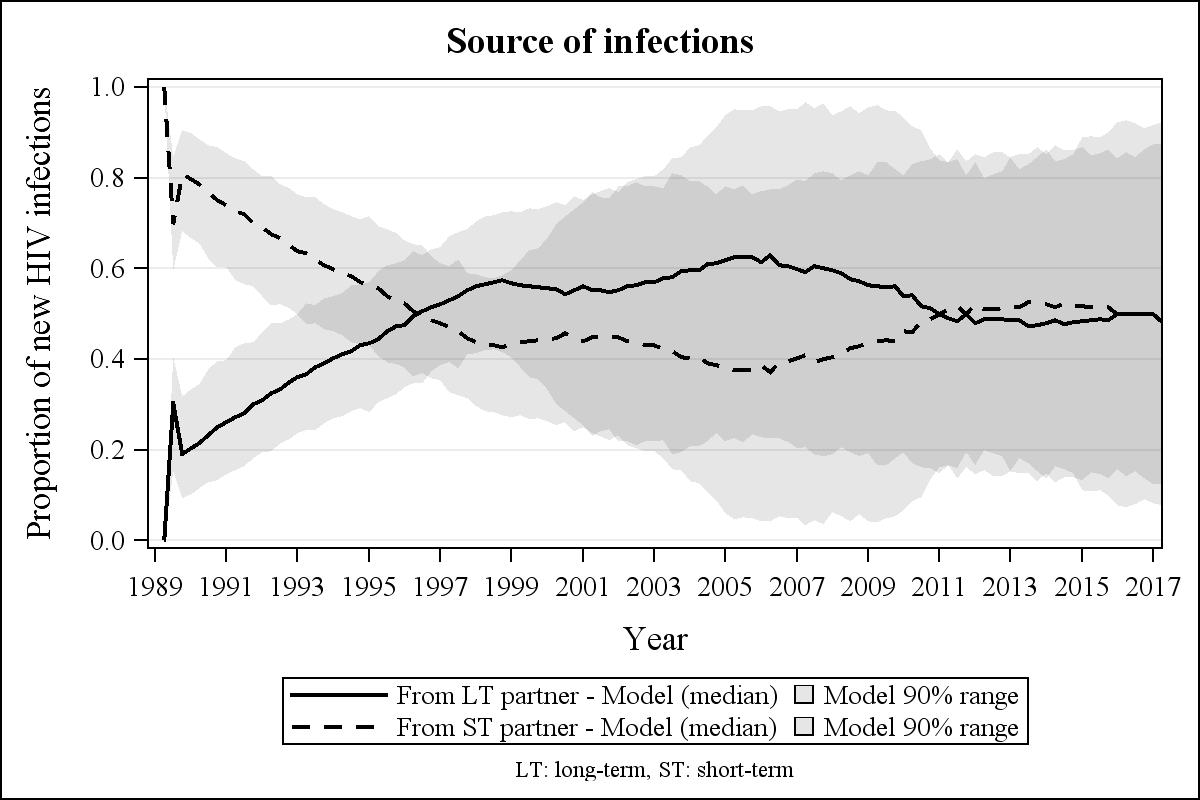


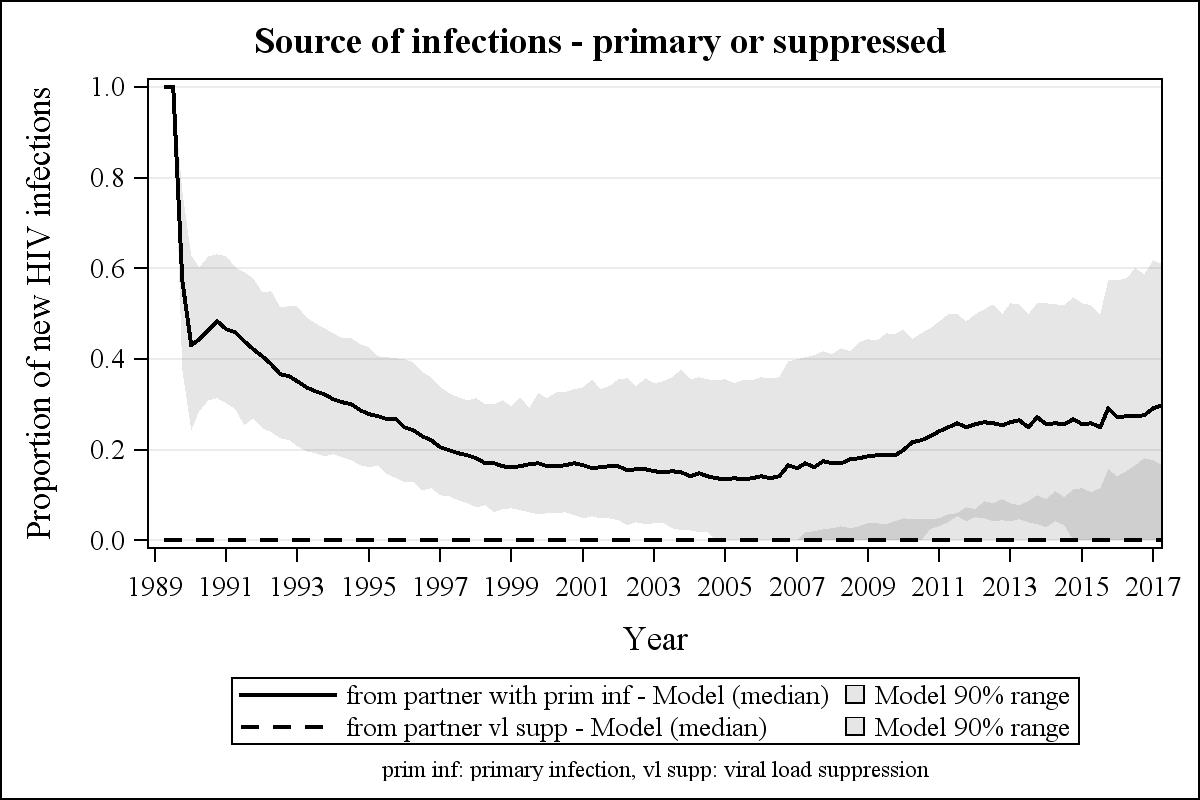


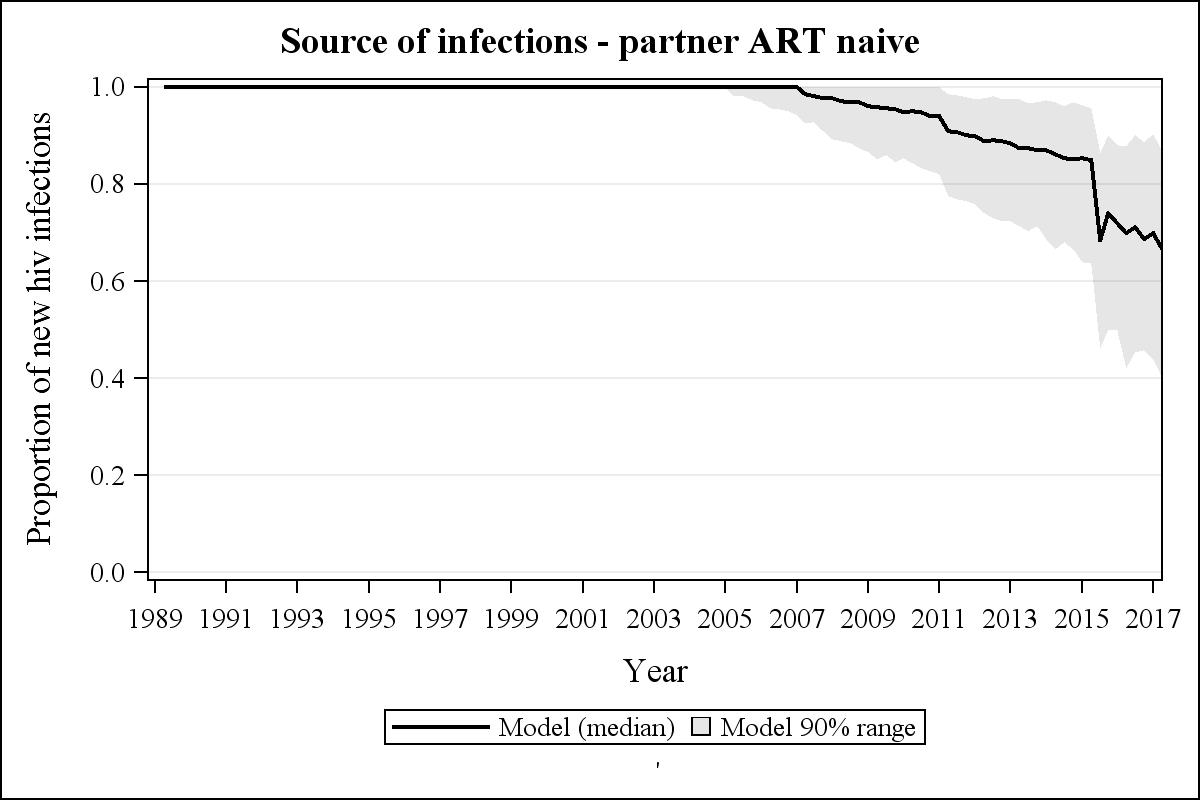


***Circumcision***


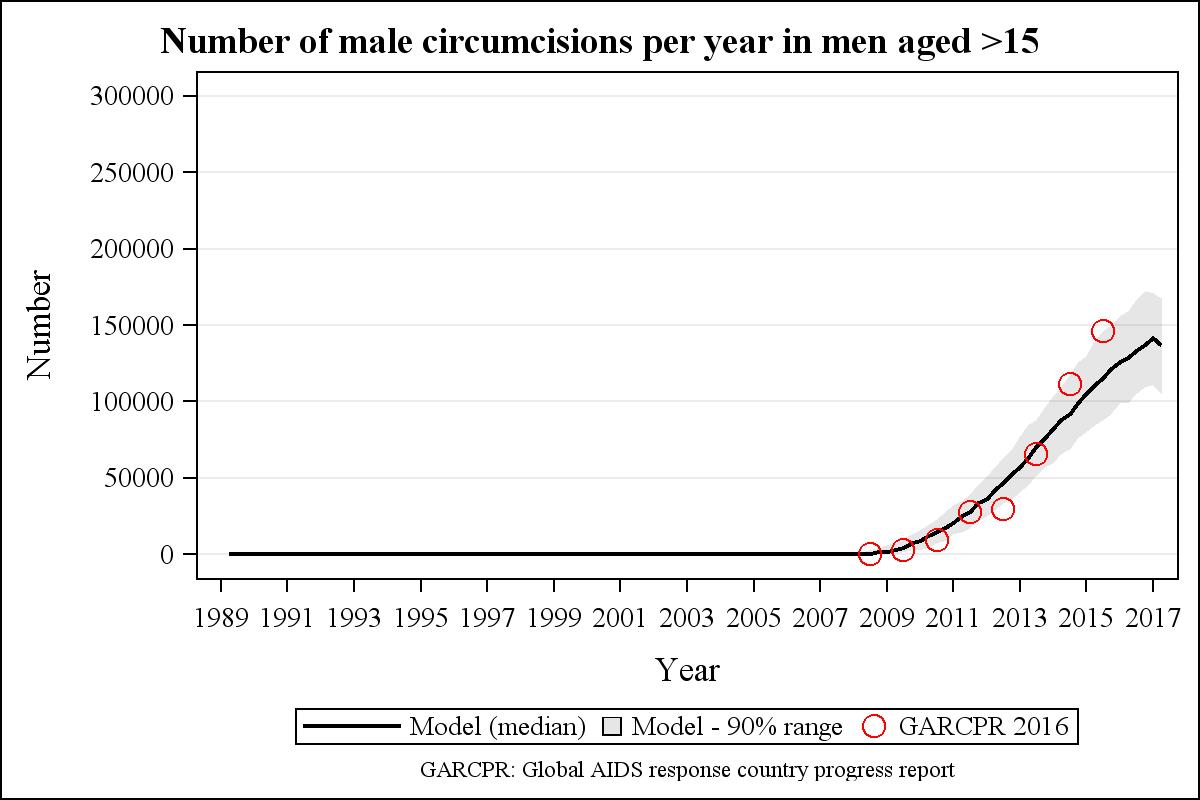


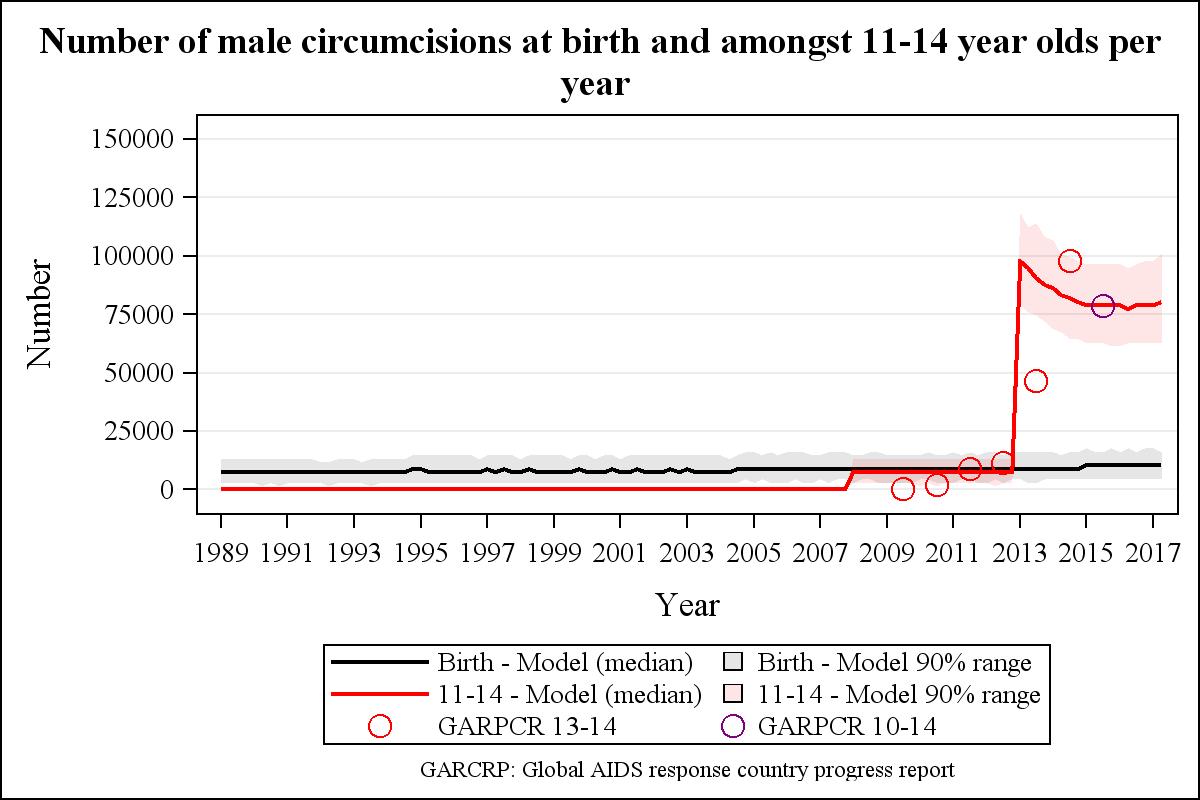


Reference List

(1) Central Statistical Office HZ. Zimbabwe Demographic and Health Survey 2005-2006. ZImbabwe; 2005.

(2) Central Statistical Office HZ. Zimbabwe Demographic and Health Survey 2015-2016. Zimbabwe; 2016.

(3) Zimbabwe National Statistics Agency, ICF International I. Zimbabwe Demographic and Health Survey 2010-2011. Zimbabwe; 2012 Mar.

(4) Zimbabwe Ministry of Health. Zimbabwe Population-Based HIV Impact Assessment ZIMPHIA 2015–2016. 2016.

(5) Joseph Murungu. Numbers of adults on 2nd line ART. 19-6-2015.

Ref Type: Personal Communication

(6) Cowan FM, Davey CB, Fearon E, Mushati P, Dirawo J, Cambiano V, et al. The HIV Care Cascade Among Female Sex Workers in Zimbabwe: Results of a Population-Based Survey From the Sisters Antiretroviral Therapy Programme for Prevention of HIV, an Integrated Response (SAPPH-IRe) Trial. J Acquir Immune Defic Syndr 2017 Apr 1;74(4):375-82.

(7) Wilson D, National AIDS Council of Zimbabwe. Zimbabwe Data. 2015.

Ref Type: Personal Communication

(8) Cowan FM. Cowan_Feedback from Sisters Programme 29 Sep 2017. 2017.

Ref Type: Personal Communication

(9) WHO. WHO HIV Drug Resistance Report 2012. 2012 Jul 1.

(10) Apollo T. Number of adults on 1st line ART by gender. 2017.

Ref Type: Personal Communication

(11) Beaumont MA, Zhang W, Balding DJ. Approximate Bayesian computation in population genetics. Genetics 2002 Dec;162(4):2025-35.

(12) UNAIDS. Global AIDS Response Progress Report 2016. 2016.

(13) Ministry of Health Z. **HIV antenatal clinic surveillance using PMTCT program data with additional quality monitoring and strengthening in Zimbabwe**. 2015.

(14) Population Division DoEaSAUN. World Population Prospects: The 2012 Revision. Zimbabwe. 2012.

(15) Mhangara M. **Maternal PMTCT Cascade Jan-Jun 2016**. 2016.

Ref Type: Personal Communication

(16) Vandepitte J, Lyerla R, Dallabetta G, Crabbe F, Alary M, Buve A. Estimates of the number of female sex workers in different regions of the world. Sex Transm Infect 2006 Jun;82 Suppl 3:iii18-iii25.

(17) Zimbabwe National Statistics Agency. Zimbabwe Census 2012 National Report. 2012.

(18) Gregson S, Gonese E, Hallett TB, Taruberekera N, Hargrove JW, Lopman B, et al. HIV decline in Zimbabwe due to reductions in risky sex? Evidence from a comprehensive epidemiological review. Int J Epidemiol 2010 Oct;39(5):1311-23.
