# Supplementary material for: 90‐90‐90 by 2020? Estimation and projection of the adult HIV epidemic and ART programme in Zimbabwe – 2017 to 2020
Source: J Int AIDS Soc. 2018 Nov 22;21(11):e25205. doi: 10.1002/jia2.25205 (PMC6250855; doi:10.1002/jia2.25205)

Alternative methods of calculating the calibration score

We considered the impact of excluding HIV prevalence data from both DHS and ZIMPHIA, given it is believed prevalence estimates could be underestimates (Option 2). We also calculated the score by favouring fit to the ZIMPHIA survey over all other sources of data (Option 3). Finally, we considered the impact of excluding HIV incidence estimates from ZIMPHIA as there is uncertainty about its accuracy (Option 4).

We identified parameter sets for each of these alternative scores and used these to project forward to 2020 to describe trends as stated in the manuscript

Table S1: Data items and weights included in original and alternative calibration scores

	Source	Weight	**Original C-score**	Option 2[1]	Option 3	Option 4
				Exclude prevalence	ZIMPHIA only	Exclude incidence
Prevalence - men 15-49	DHS/ZIMPHIA (2;6;11;12)	1	✓		✓ (not DHS)	✓
Prevalence - women 15-49	DHS/ ZIMPHIA (2;6;11;12)	1	✓		✓ (not DHS)	✓
Prevalence - men 15-24	DHS (6;11;12)	0.5	✓			✓
Prevalence - women 15-24	DHS (6;11;12)	0.5	✓			✓
Prevalence – FSW 15-65	Cowan 2017 (13)	0.5	✓	✓		✓
Incidence – men 15-49	ZIMPHIA (2)	0.5	✓	✓	✓ (W[2]=1)	
Incidence – women 15-49	ZIMPHIA (2)	0.5	✓	✓	✓ (W=1)	
Number of tests – all 15-49	GARCPR 2016 (14)	1	✓	✓		✓
Proportion diagnosed – men 15-65	ZIMPHIA (2)	1	✓	✓	✓	✓
Proportion diagnosed – women 15-65	ZIMPHIA (2)	1	✓	✓	✓	✓
Number on ART[3] – men 15-65	MoH (15)	1	✓	✓		✓
Number on ART[3] – women 15-65	MoH (15)	1	✓	✓		✓
Number on 2nd line – all 15-65	MoH (16)	0.5	✓	✓		✓
Proportion suppressed of PLHIV – men 15-65	ZIMPHIA (2)	1	✓	✓	✓	✓
Proportion suppressed of PLHIV– women 15-65	ZIMPHIA (2)	1	✓	✓	✓	✓
Proportion with resistance at ART initiation – all 15-65	WHO Resistance report 2012 (17)	0.33	✓	✓		✓
Number of FSW 18-49	Cowan (18)	0.5	✓	✓		✓
Number of pregnancies 15-49	MoH/Mhangara/United Nations (UN) (19-21)	0.75	✓	✓		✓
Proportion tested in last year – men 15-49	DHS (6;11;12)	1				
Proportion tested in last year – women 15-49	DHS (6;11;12)	1				

DHS: Zimbabwe Demographic and Health Survey; ZIMPHIA: Zimbabwe population based HIV impact assessment: GARCPR: Global AIDS Response Country Progress Report; MoH: Ministry of Health, Zimbabwe; WHO: World Health Organisation

[1] Runs with prevalence >0.35 or <0.15 amongst women aged 15-49 in 1997 and prevalence >0.25 or <0.11 amongst all 15-49 year olds in 2006 were aborted when calculating the c-score. For option 2, these criteria were loosened to prevalence >0.45 or <0.15 amongst women aged 15-49 in 1997 and prevalence >0.35 or <0.11 amongst all 15-49 year olds in 2006

[2] W: Weight

[3] A higher emphasis was given to the observed data in later years (2014 and 2015) as these are known to be reliable data sources. Hence the c-score components for data from 2014 and 2015 were multiplied by 5 before being added to the overall score

Figure S1: Median (90% range) modelled projections using the alternative calibration scores at 2017 and 2020

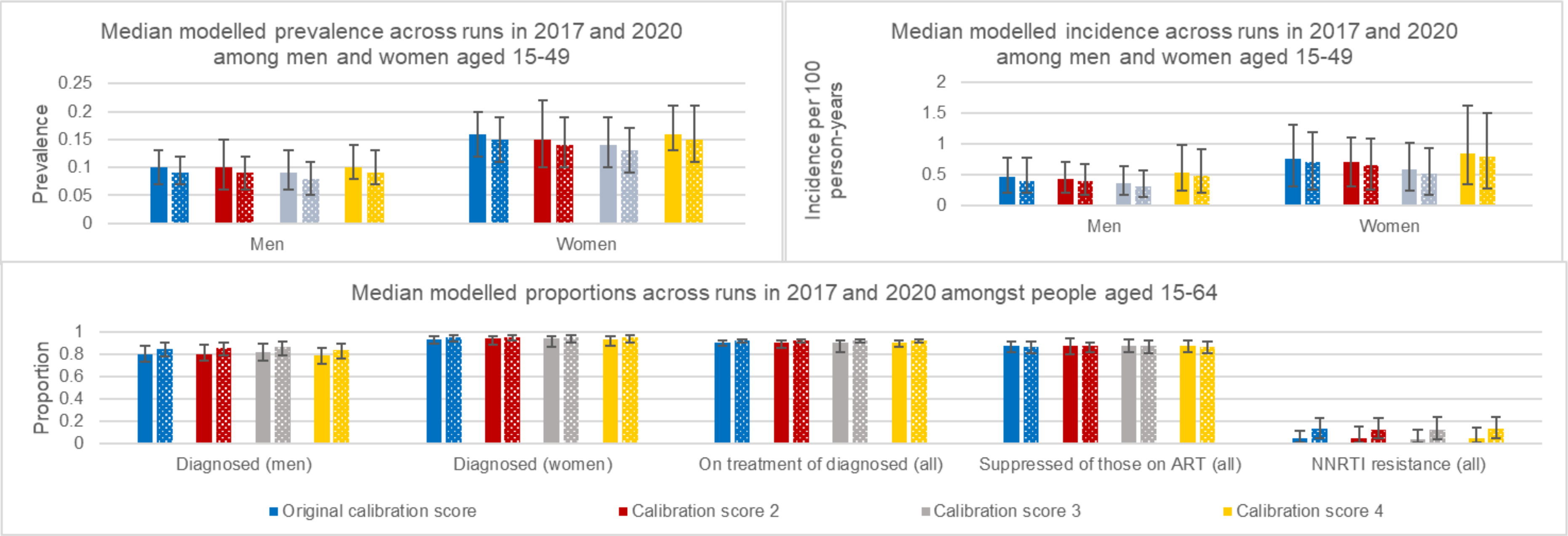

Table 3: Projections of absolute change between 2017 and 2020 estimates (median over 1500 runs, with 90% range)

	**Original C-score**	Option 2	Option 3	Option 4
		Exclude prevalence	ZIMPHIA only	Exclude incidence
Prevalence - men 15-49	-0.01 (-0.02, -0.00)	-0.01 (-0.02, -0.01)	-0.01 (-0.02, 0.00)	-0.01 (-0.02, -0.00)
Prevalence - women 15-49	-0.01 (-0.02, -0.00)	-0.01 (-0.02, -0.00)	-0.01 (-0.03, 0.00)	-0.01 (-0.02, 0.00)
Incidence per 100py men 15-49	-0.05 (-0.13, 0.02)	-0.06 (-0.39, 0.18)	-0.06 (-0.35, 0.18)	-0.05 (-0.38, 0.22)
Incidence per 100py – women 15-49	-0.07 (-0.22, 0.06)	-0.07 (-0.51, 0.29)	-0.08 (-0.45, 0.28)	-0.08 (-0.54, 0.33)
Proportion diagnosed – men 15-64	0.05 (0.02, 0.07)	0.04 (0.02, 0.07)	0.04 (0.01, 0.07)	0.04 (0.02, 0.07)
Proportion diagnosed – women 15-64	0.02 (0.00, 0.04)	0.02 (0.00, 0.03)	0.02 (0.00, 0.04)	0.02 (0.00, 0.04)
Proportion on treatment of those diagnosed –all 15-64	0.02 (0.01, 0.06)	0.02 (0.01, 0.05)	0.02 (0.00, 0.09)	0.02 (0.01, 0.06)
Proportion suppressed of those on ART – all 15-64	-0.01 (-0.02, 0.01)	-0.01 (-0.03, 0.01)	-0.01 (-0.02, 0.01)	-0.01 (-0.02, 0.01)
Proportion with resistance – all 15-64	0.07 (0.01, 0.16)	0.07 (0.02, 0.17)	0.07 (0.01, 0.16)	0.07 (0.02, 0.18)

S2 Figure: Median (90% range) modelled projections at 2017 and 2020 with *no* restriction on c-score

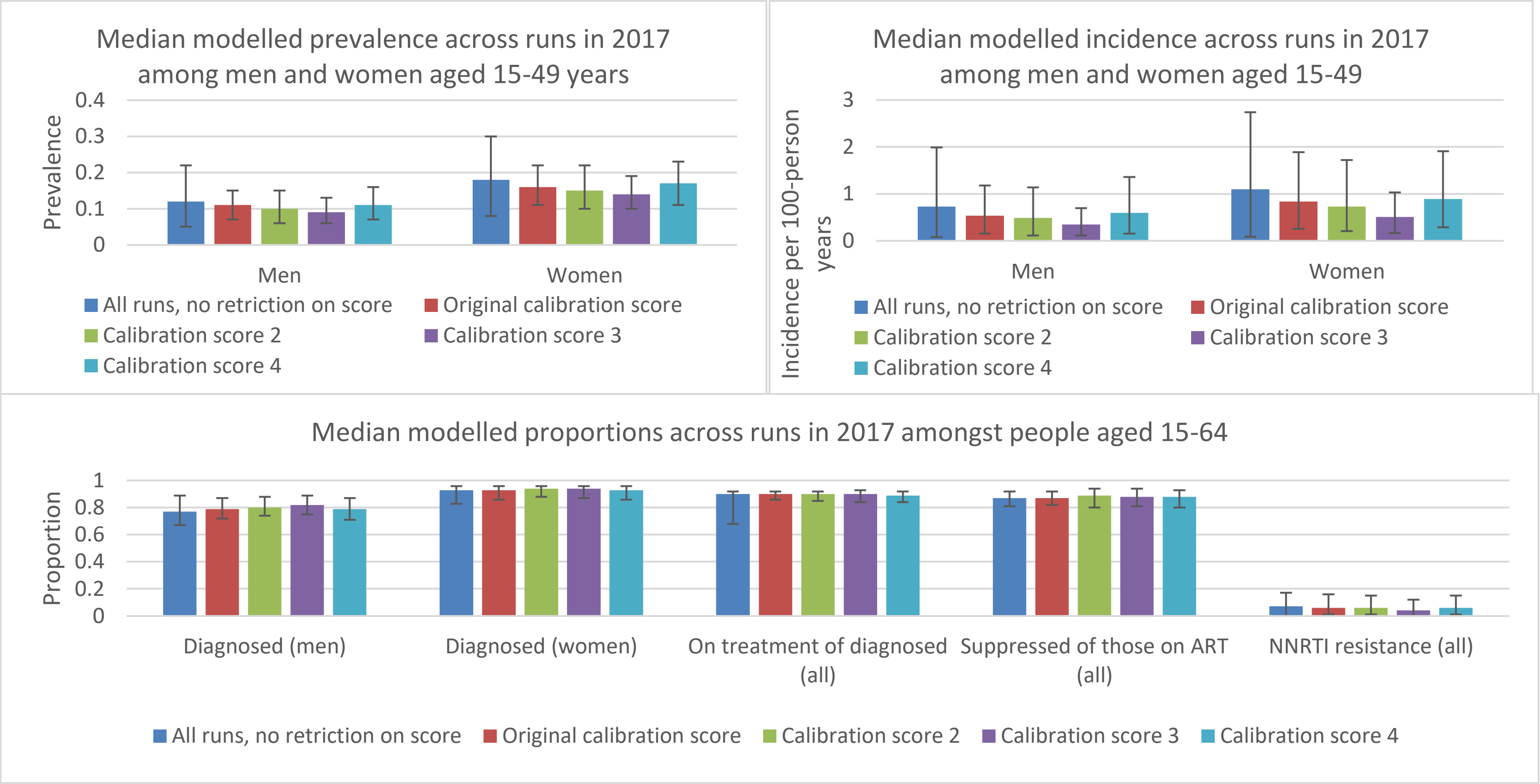

Supplement: Supplementary file 3 — Data S3. Alternative methods of calculating the calibration score. [file JIA2-21-e25205-s003.pdf]
